# Corrigendum: CD317-Positive Immune Stromal Cells in Human “Mesenchymal Stem Cell” Populations

**DOI:** 10.3389/fimmu.2022.1111042

**Published:** 2022-12-13

**Authors:** Alasdair G. Kay, James M. Fox, James P. Hewitson, Andrew P. Stone, Sophie Robertson, Sally James, Xiao-nong Wang, Elizabeth Kapasa, Xuebin B. Yang, Paul G. Genever

**Affiliations:** ^1^ York Biomedical Research Institute and Department of Biology, University of York, York, United Kingdom; ^2^ Translational and Clinical Research Institute, Newcastle University, Newcastle, United Kingdom; ^3^ Department of Oral Biology, School of Dentistry, University of Leeds, St James’s University Hospital, Leeds, United Kingdom

**Keywords:** mesenchymal stromal cells, MSC subtypes, heterogeneity, immunomodulation, CD317, BST2, tetherin

In the published article, there was an error in the **Acknowledgments** section. An acknowledgment that would have been included at the time of submission was not included due to inability to obtain the written approval required which has become available now.

A correction has been made to **Acknowledgments**. The section previously stated:

“The authors thank the staff and patients of Clifton Park Hospital for samples. We are grateful to the University of York Technology Facility for support with flow cytometry, cell sorting, confocal microscopy bioinformatics and proteomic analysis. We also thank the York Centre of Excellence in Mass Spectrometry. We thank Emily Taylor for assistance in isolating primary T cells”.

The corrected **Acknowledgments** appears below:

“The authors thank the staff and patients of Clifton Park Hospital for samples and Emily Taylor for assistance isolating primary T cells. We thank the University of York Technology Facility for support with flow cytometry, cell sorting, confocal microscopy and proteomic analysis as well as the York Centre of Excellence in Mass Spectrometry. The authors thank Gillian Gough, MRes student at Newcastle University, for contributions to acquisition, analysis, and interpretation of skin explant data.”

The authors apologize for this error and state that this does not change the scientific conclusions of the article in any way. The original article has been updated.

## Publisher’s note

All claims expressed in this article are solely those of the authors and do not necessarily represent those of their affiliated organizations, or those of the publisher, the editors and the reviewers. Any product that may be evaluated in this article, or claim that may be made by its manufacturer, is not guaranteed or endorsed by the publisher.

