# Supplementary material for: CD317-Positive Immune Stromal Cells in Human “Mesenchymal Stem Cell” Populations
Source: Front Immunol. 2022 Jun 6;13:903796. doi: 10.3389/fimmu.2022.903796 (PMC9207511; doi:10.3389/fimmu.2022.903796)
Supplement: Supplementary file 1 [file DataSheet_1.docx]

**Supplementary Data**

**Index:**

**Table S1** – Gene Expression Omnibus (GEO) summary data for 01 and 02 cell lines

**Table S2** – Antibodies used

**Table S3** – Primer Sequences

**Table S4** – Rohart Scores for individual cell lines

**Table S5** – Surfaceomic MS data for hTERT cell lines

**Table S6** – Differentially Expressed Gene (DEG) data for hTERT cell lines

**Table S7** – Gene ontology (GO) term associations between CD317^pos^ cell lines and autoimmune disease states

**Table S8** – Upregulated signalling pathway associations between CD317^pos^ cell lines and autoimmune disease states

**Figure S1**

**Figure S2**

**Figure S3**

**Figure S4**

# **Table S1** Antibodies used (h denotes anti-human, m denotes anti-mouse antibody).

| **Antibody** | **Conjugate** | **Clone/Cat. number** | **Source** |
| --- | --- | --- | --- |
| Mouse anti-human CD317 | PE/AF488 | 26F8 | eBioscience |
| Goat anti-human CXCL10 |  | AF-266-NA | Bio-techne |
| Goat anti-human CXCL11 |  | AF260 | Bio-techne |
| Mouse anti-human CD54/ICAM-1 (staining) |  | HA58 | eBioscience |
| Mouse anti-human CD54/ICAM-1 (neutralising) |  | 15.2 | Santa Cruz Biotechnology Inc. |
| Goat anti-human IFN-γ-R2 |  | AF773 | Bio-techne |
| Mouse anti-human CD119/IFNγR1 | PE | GIR-208 | eBioscience |
| Mouse anti-human CD54/ICAM-1 | APC | HA58 | eBioscience |
| Mouse IgG1 1 Κ isotype control | PE/APC/AF488 | P3.6.2.8.1 | eBioscience |
| Mouse IgG2a isotype control |  | MOPC-173 | BioLegend |
| Rabbit anti-mouse CD317 |  | NBP2-27154 | Bio-techne |
| Goat anti-mouse Leptin Receptor |  | BAF497 | Bio-techne |
| Goat Anti-mouse-RPE | RPE | M32204, 10557603 | Invitrogen |
| Goat Anti-mouse-AF647 (IgG H&L) | AF647 | A-21235 | Invitrogen |
| Goat Anti-mouse-AF488 (IgG H&L) | AF488 | A11001 | Invitrogen |
| Rat Anti-mouse-Ly6C | APC | HK1.4, 128016 | Biolegend |
| Rat Anti-mouse-Ly6G | FITC | 1A8, 551460 | BD |
| Rat Anti-mouse-F4/80 | PE-Cy7 | BM8, 123114 | Biolegend |
| Rat Anti-mouse-CD11b | BUV395 | M1/70, 563553 | BD |
| Rat Anti-mouse-CD45 | PerCP-Cy5.5 | 30-F11, 103132 | Biolegend |
| Rat Anti-mouse-SiglecF | BV421 | E50-2440, 562681 | BD |
| Live/Dead | Zombie Aqua | 423101 | Biolegend |
| Rat Anti-mouse-CD8a | PerCP-Cy5.5 | 53-6.7, 100734 | Biolegend |
| Rat Anti-mouse-CD4 | FITC | GK1.5, 100406 | Biolegend |
| Rat Anti-mouse-IL4 | PE | 11B11, 504104 | Biolegend |
| Rat Anti-mouse-IFN-g | APC | XMG1.2, 505810 | Biolegend |
| Rat Anti-mouse-IL17a | BV421 | TC11-18H10, 563354 | BD |
| Rat Anti-mouse-CD8a | PerCP-Cy5.5 | 53-6.7, 100734 | Biolegend |
| Rat Anti-mouse-CD8b | BV421 | YTS156.7.7, 126629 | Biolegend |
| Rat Anti-mouse-FOXP3 | APC | FJK-15s, 17-5773-82 | eBioscience |
| Rat Anti-mouse-CD25 | PE | PC61, 102008 | Biolegend |
| Hamster Anti-mouse-TCRb | Alexa-fluor 488 | H57-597, 109215 | Biolegend |
| Rat Anti-mouse-CD3 | APC-Cy7 | 17a2, 100222 | Biolegend |
| Rat Anti-mouse-CD4 | PerCP-Cy5.5 | RM4-5, 100540 | Biolegend |
| Rat Anti-mouse-CD62L | APC | MEL-14, 104412 | Biolegend |
| Rat Anti-mouse-CD44 | PE | IM7, 103008 | Biolegend |
| Mouse IgG1 1 Κ isotype control | PE/APC/AF488 | P3.6.2.8.1, 16-4714-82 | eBioscience |
| Mouse IgG2a isotype control |  | MOPC-173 | BioLegend |
| Rat IgG2c k isotype control | APC/PE-Cy7/ PerCP-Cy5.5 | RTK4174, 400713 / RTK2758, 400522 / RTK2758 / 400532 | Biolegend |
| Rat IgG2a, κ isotype control | FITC / BV421 / PerCP-Cy5.5 / APC / PE / APC (2) / | R35-95, 553929 / R35-95, 562602 / RTK2758, 400532 / eBR2a, 17-4321-81 / RTK4530, 400608 / RTK2758, 400512 | BD |
| Rat IgG2b, κ isotype control | PerCP-Cy5.5 / FITC / BUV395 / BV421 / APC-Cy7 | RTK4530, 400632 / RTK4530, 400606 / R35-38, 563560 / RTK4530, 400655 / RTK4530 / 400624 | Biolegend |
| Rat IgG1, κ isotype control | PE / APC / BV421 | RTK2758, 400408 / RTK2071, 400412 / R3-34, 562868 | Biolegend |
| Rat IgG1, λ isotype control | PE | G0114F7, 401906 | Biolegend |
| Armenian Hamster IgG isotype control | Alexa-fluor 488 | HTK888, 400923 | Biolegend |
| Mouse anti-human CD317 | PE-Cy7 | RS38E, 348416 | Biolegend |
| Mouse anti-human CD271 | Alexa-fluor 647 | ME20.4, 345114 | Biolegend |
| Mouse anti-human CD164 | PE | 67D2, 324808 | Biolegend |
| Mouse anti-human CD146 | FITC | P1H12, 361012 | Biolegend |
| Mouse anti-human CD4 | FITC | SK3, 11-0047-42 | eBioscience |
| Mouse anti-human IL-4 | APC | 8D4-8, 17-7049-82 | eBioscience |
| Mouse anti-human IFN-g | PE | 4S.B3, 12-7319-82 | eBioscience |
| Mouse anti-human IL-17a | PE-Cy7 | eBio64Dec17, 25-7179-42 | eBioscience |
| Mouse anti-human CD25 | APC (2) | CD25-4E3, 17-0257-42 | eBioscience |
| Rat anti-human FOXP3 | PE | PCH101, 12-4776-42 | eBioscience |
| Mouse IgG2b κ isotype control | APC / PerCP-Cy5.5 / APC (2) | eBMG2b, 17-4732-42 / 27-35, 558304 / 27-35, 555745 | eBioscience |
| Mouse IgG1 κ isotype control | APC / PE-CF594 / BV421 / FITC / PE / PE-Cy7 / AF647 | MOPC-21, 555751 / X40, 562292 / X40, 562438 / P3.6.2.8.1, 11-4714-81 / P3.6.2.8.1, 12-4714-42 / P3.6.2.8.1, 25-4714-41 / MOP-21, 400130 | BD |

# **Table S2** Primer sequences

| **Target** | **Forward primer sequence** | **Reverse primer sequence** |
| --- | --- | --- |
| RPS27a | Tgg at gaga atg gca aaa tta gtc | CAC CCC AGC ACC ACA TTC A |
| CD318/CDCP1 | TGG TTC CAC CCC AGA AAT GT | AAT CTA CGT GGT TGA CTT GAG TAA |
| CXCL10/IP10 | AAG CAG TTA GCA AGG AAA GGT CTA | GCA TCG ATT TTG CTC CCC TC |
| CXCL11 | CCT TGG CTG TGA TAT TGT GTG C | TGA ACA TGG GGA AGC CTT GAA |
| EPSTI1 | TGC ATA CAC CTT GAT AGC ACC AA | TCC TGC TCC GCA ATT CTT TG |
| HERC5 | GAG CTA AGA CCC TGT TTG G | CCA CCT TCC ACA TGC TAT C |
| IFI44L | TGC TCC TTC TGC CCC ATC TA | TGC TCC TTC TGC CCC ATC TA |
| ISG15 | ATG TCG GTG TCA GAG CTG AAG | GTT ATT CCT CAC CAG GAT GCT C |
| LY6E | CCT GGA GTC TTA CGG TCC AA | GTA CAC AGC CAG GCA CAC AT |
| MX1 | TTC AGC ACC TGA TGG CCT ATC | GTA CGT CTG GAG CAT GAA GAA CTG |
| MX2 | CAG AGG CAG CGG AAT CGT AA | TGA AGC TCT AGC TCG GTG TTC |
| RSAD2 | GTG GTT CCA GAA TTA TGG TGA GTA TTT | CCA CGG CCA ATA AGG ACA TT |
| CX3CL1/Fractalkine | CGG CAA ACG CGC AAT CAT C | TTC TCG AAG GTG CCG CCA TTT |
| SAA1 | ACT CTG TTT TCT CTT TCC CAA CAA | GAA GCT TCA TGG TGC TCT CTA |
| SAA2 | TAA ATA GCA GCC ACC TCT CCC T | TTC ATG GTG CTG ATC TGT GC |
| SAA4 | GGA GAA AGG TCC ACA GCA CAA T | ATG TCC CAA TAG GCT CTG CC |
| CCL5/RANTES | GAG TAT TTC TAC ACC AGT GGC AAG | TCC CGA ACC CAT TTC TTC TCT |
| CD54/ICAM1 | GCC AGG AGA CAC TGC AGA CA | TGG CTT CGT CAG AAT CAC GTT |
| CCL2/MCP1 | CAT AGC AGC CAC CTT CAT TCC | TCT CCT TGG CCA CAA TGG TC |

# **Table S3** Rohart scores for parental cell line FH181 and hTERT immortalised cells

| **Replicate Group ID** | **Prediction** | **Lower Bound** | **Upper Bound** | **MSC Calls** | **Total Sub-samplings** | **Percentage** |
| --- | --- | --- | --- | --- | --- | --- |
| FH181, replicate 1 | 0.85 | 0.82 | 0.89 | 200 | 200 | 100 |
| FH181, replicate 2 | 0.86 | 0.82 | 0.89 | 200 | 200 | 100 |
| FH181, replicate 3 | 0.97 | 0.92 | 1.01 | 200 | 200 | 100 |
| Y101, replicate 1 | 1.26 | 1.2 | 1.31 | 200 | 200 | 100 |
| Y101, replicate 2 | 1.01 | 0.97 | 1.05 | 200 | 200 | 100 |
| Y101, replicate 3 | 0.98 | 0.94 | 1.02 | 200 | 200 | 100 |
| Y102, replicate 1 | 0.6 | 0.55 | 0.65 | 200 | 200 | 100 |
| Y102, replicate 2 | 0.55 | 0.5 | 0.6 | 179 | 200 | 89.5 |
| Y102, replicate 3 | 0.81 | 0.74 | 0.87 | 200 | 200 | 100 |
| Y201, replicate 1 | 0.94 | 0.9 | 0.97 | 200 | 200 | 100 |
| Y201, replicate 2 | 1 | 0.95 | 1.03 | 200 | 200 | 100 |
| Y201, replicate 3 | 1 | 0.95 | 1.03 | 200 | 200 | 100 |
| Y202, replicate 1 | 0.63 | 0.57 | 0.68 | 200 | 200 | 100 |
| Y202, replicate 2 | 0.62 | 0.57 | 0.67 | 200 | 200 | 100 |
| Y202, replicate 3 | 0.66 | 0.6 | 0.72 | 200 | 200 | 100 |

# **Table S4** Surfaceomic data for MSC lines

|  | C431_Fox |  |  |  | Y101 | Y101 | Y101 | Y102 | Y102 | Y102 | Y201 | Y201 | Y201 | Y202 | Y202 | Y202 |
| --- | --- | --- | --- | --- | --- | --- | --- | --- | --- | --- | --- | --- | --- | --- | --- | --- |
| # | Identified Proteins (2630/2678) | Accession Number | Molecular Weight /kDA | ANOVA Test (p-value): | Y101_A | Y101_B | Y101_C | Y102_A | Y102_B | Y102_C | Y201_A | Y201_B | Y201_C | Y202_A | Y202_B | Y202_C |
| 1 | Contactin-associated protein 1 OS=Homo sapiens GN=CNTNAP1 PE=1 SV=1 | CNTP1 | 156 | 0.032 | 100% | 100% | 100% | 100% | 100% | 100% | 100% | 100% | 100% | 100% | 100% | 100% |
| 2 | Symplekin OS=Homo sapiens GN=SYMPK PE=1 SV=2 | SYMPK | 141 | 0.035 | 0 | 100% | 98% | 95% | 95% | 100% | 100% | 100% | 100% | 100% | 100% | 100% |
| 3 | Cluster of Ras-related protein Rap-2b OS=Homo sapiens GN=RAP2B PE=1 SV=1 (RAP2B_HUMAN) | RAP2B [2] | 21 | 0.45 | 95% | 100% | 100% | 10% | 100% | 100% | 0 | 100% | 95% | 100% | 100% | 100% |
| 4 | Y-box-binding protein 3 OS=Homo sapiens GN=YBX3 PE=1 SV=4 | YBOX3 | 40 | 0.036 | 0 | 0 | 100% | 0 | 97% | 100% | 100% | 99% | 100% | 99% | 90% | 100% |
| 5 | Striatin OS=Homo sapiens GN=STRN PE=1 SV=4 | STRN | 86 | 0.054 | 0 | 0 | 0 | 99% | 98% | 100% | 0 | 0 | 95% | 0 | 80% | 0 |
| 6 | CXADR-like membrane protein OS=Homo sapiens GN=CLMP PE=1 SV=1 | CLMP | 41 | 0.018 | 93% | 100% | 100% | 0 | 0 | 0 | 0 | 0 | 0 | 0 | 95% | 77% |
| 7 | Collagen alpha-3(VI) chain OS=Homo sapiens GN=COL6A3 PE=1 SV=5 | CO6A3 | 344 | < 0.00010 | 100% | 100% | 100% | 100% | 100% | 100% | 100% | 100% | 100% | 100% | 100% | 100% |
| 8 | Cluster of Keratin, type I cytoskeletal 10 OS=Homo sapiens GN=KRT10 PE=1 SV=6 (K1C10_HUMAN) | K1C10 [10] | 59 | < 0.00010 | 100% | 100% | 100% | 100% | 100% | 100% | 100% | 100% | 100% | 100% | 100% | 100% |
| 9 | Collagen alpha-1(VI) chain OS=Homo sapiens GN=COL6A1 PE=1 SV=3 | CO6A1 | 109 | < 0.00010 | 100% | 100% | 100% | 100% | 100% | 100% | 100% | 100% | 100% | 100% | 100% | 100% |
| 10 | Collagen alpha-2(VI) chain OS=Homo sapiens GN=COL6A2 PE=1 SV=4 | CO6A2 | 109 | 0.0013 | 100% | 100% | 100% | 99% | 100% | 0 | 100% | 100% | 100% | 100% | 100% | 100% |
| 11 | Biglycan OS=Homo sapiens GN=BGN PE=1 SV=2 | PGS1 | 42 | < 0.00010 | 0 | 100% | 100% | 0 | 39% | 0 | 100% | 100% | 100% | 100% | 100% | 100% |
| 12 | Beta-1-syntrophin OS=Homo sapiens GN=SNTB1 PE=1 SV=3 | SNTB1 | 58 | 0.0045 | 100% | 100% | 100% | 100% | 100% | 100% | 0 | 0 | 0 | 100% | 100% | 100% |
| 13 | Sarcolemmal membrane-associated protein OS=Homo sapiens GN=SLMAP PE=1 SV=1 | SLMAP | 95 | < 0.00010 | 0 | 0 | 35% | 100% | 100% | 100% | 98% | 100% | 84% | 100% | 100% | 100% |
| 14 | Delta-sarcoglycan OS=Homo sapiens GN=SGCD PE=1 SV=2 | SGCD | 32 | 0.0099 | 100% | 100% | 100% | 100% | 100% | 100% | 100% | 95% | 95% | 100% | 100% | 100% |
| 15 | Fukutin-related protein OS=Homo sapiens GN=FKRP PE=1 SV=1 | FKRP | 55 | 0.057 | 0 | 14% | 95% | 19% | 100% | 100% | 100% | 100% | 100% | 95% | 95% | 100% |
| 16 | Synemin OS=Homo sapiens GN=SYNM PE=1 SV=2 | SYNEM | 173 | < 0.00010 | 0 | 0 | 0 | 0 | 30% | 32% | 100% | 100% | 100% | 0 | 0 | 0 |
| 17 | Beta-sarcoglycan OS=Homo sapiens GN=SGCB PE=1 SV=1 | SGCB | 35 | < 0.00010 | 0 | 0 | 0 | 99% | 98% | 95% | 0 | 0 | 0 | 0 | 0 | 0 |
| 18 | Elongation factor 1-alpha 1 OS=Homo sapiens GN=EEF1A1 PE=1 SV=1 | EF1A1 (+1) | 50 | 0.028 | 100% | 100% | 100% | 100% | 100% | 100% | 100% | 100% | 100% | 100% | 100% | 100% |
| 19 | Ras-related protein Rab-34 OS=Homo sapiens GN=RAB34 PE=1 SV=1 | RAB34 | 29 | 0.57 | 99% | 100% | 99% | 99% | 0 | 72% | 0 | 97% | 0 | 99% | 98% | 95% |
| 20 | NADH dehydrogenase [ubiquinone] flavoprotein 2, mitochondrial OS=Homo sapiens GN=NDUFV2 PE=1 SV=2 | NDUV2 | 27 | 0.0073 | 95% | 95% | 95% | 95% | 95% | 95% | 0 | 0 | 0 | 95% | 98% | 12% |
| 21 | Cluster of Vimentin OS=Homo sapiens GN=VIM PE=1 SV=4 (VIME_HUMAN) | VIME [21] | 54 | 0.058 | 100% | 100% | 100% | 100% | 100% | 100% | 100% | 100% | 100% | 100% | 100% | 100% |
| 22 | Cluster of Actin, cytoplasmic 1 OS=Homo sapiens GN=ACTB PE=1 SV=1 (ACTB_HUMAN) | ACTB [8] | 42 | 0.47 | 100% | 100% | 100% | 100% | 100% | 100% | 100% | 100% | 100% | 100% | 100% | 100% |
| 23 | Cluster of Plectin OS=Homo sapiens GN=PLEC PE=1 SV=3 (PLEC_HUMAN) | PLEC [2] | 532 | 0.00021 | 100% | 100% | 100% | 100% | 100% | 100% | 100% | 100% | 100% | 100% | 100% | 100% |
| 24 | Neuroblast differentiation-associated protein AHNAK OS=Homo sapiens GN=AHNAK PE=1 SV=2 | AHNK | 629 | < 0.00010 | 100% | 100% | 100% | 100% | 100% | 100% | 100% | 100% | 100% | 100% | 100% | 100% |
| 25 | Annexin A2 OS=Homo sapiens GN=ANXA2 PE=1 SV=2 | ANXA2 | 39 | 0.12 | 100% | 100% | 100% | 100% | 100% | 100% | 100% | 100% | 100% | 100% | 100% | 100% |
| 26 | Myoferlin OS=Homo sapiens GN=MYOF PE=1 SV=1 | MYOF | 235 | 0.0023 | 100% | 100% | 100% | 100% | 100% | 100% | 100% | 100% | 100% | 100% | 100% | 100% |
| 27 | Cluster of Myosin-9 OS=Homo sapiens GN=MYH9 PE=1 SV=4 (MYH9_HUMAN) | MYH9 [3] | 227 | 0.0026 | 100% | 100% | 100% | 100% | 100% | 100% | 100% | 100% | 100% | 100% | 100% | 100% |
| 28 | Cytoskeleton-associated protein 4 OS=Homo sapiens GN=CKAP4 PE=1 SV=2 | CKAP4 | 66 | 0.0037 | 100% | 100% | 100% | 100% | 100% | 100% | 100% | 100% | 100% | 100% | 100% | 100% |
| 29 | Cluster of HLA class I histocompatibility antigen, A-11 alpha chain OS=Homo sapiens GN=HLA-A PE=1 SV=1 (1A11_HUMAN) | 1A11 [16] | 41 | < 0.00010 | 100% | 100% | 100% | 100% | 100% | 100% | 100% | 100% | 100% | 100% | 100% | 100% |
| 30 | Chondroitin sulfate proteoglycan 4 OS=Homo sapiens GN=CSPG4 PE=1 SV=2 | CSPG4 | 251 | 0.0003 | 100% | 100% | 100% | 100% | 100% | 100% | 100% | 100% | 100% | 100% | 100% | 100% |
| 31 | 78 kDa glucose-regulated protein OS=Homo sapiens GN=HSPA5 PE=1 SV=2 | GRP78 | 72 | 0.18 | 100% | 100% | 100% | 100% | 100% | 100% | 100% | 100% | 100% | 100% | 100% | 100% |
| 32 | Glyceraldehyde-3-phosphate dehydrogenase OS=Homo sapiens GN=GAPDH PE=1 SV=3 | G3P | 36 | 0.00022 | 100% | 100% | 100% | 100% | 100% | 100% | 100% | 100% | 100% | 100% | 100% | 100% |
| 33 | Extended synaptotagmin-1 OS=Homo sapiens GN=ESYT1 PE=1 SV=1 | ESYT1 | 123 | 0.21 | 100% | 100% | 100% | 100% | 100% | 100% | 100% | 100% | 100% | 100% | 100% | 100% |
| 34 | Cluster of Prolow-density lipoprotein receptor-related protein 1 OS=Homo sapiens GN=LRP1 PE=1 SV=2 (LRP1_HUMAN) | LRP1 [2] | 505 | < 0.00010 | 100% | 100% | 100% | 100% | 100% | 100% | 100% | 100% | 100% | 100% | 100% | 100% |
| 35 | Unconventional myosin-Ic OS=Homo sapiens GN=MYO1C PE=1 SV=4 | MYO1C | 122 | < 0.00010 | 100% | 100% | 100% | 100% | 100% | 100% | 100% | 100% | 100% | 100% | 100% | 100% |
| 36 | Cluster of Endoplasmin OS=Homo sapiens GN=HSP90B1 PE=1 SV=1 (ENPL_HUMAN) | ENPL [9] | 92 | 0.099 | 100% | 100% | 100% | 100% | 100% | 100% | 100% | 100% | 100% | 100% | 100% | 100% |
| 37 | Integrin beta-1 OS=Homo sapiens GN=ITGB1 PE=1 SV=2 | ITB1 | 88 | 0.02 | 100% | 100% | 100% | 100% | 100% | 100% | 100% | 100% | 100% | 100% | 100% | 100% |
| 38 | Cluster of Moesin OS=Homo sapiens GN=MSN PE=1 SV=3 (MOES_HUMAN) | MOES [3] | 68 | 0.005 | 100% | 100% | 100% | 100% | 100% | 100% | 100% | 100% | 100% | 100% | 100% | 100% |
| 39 | Cluster of Catenin alpha-1 OS=Homo sapiens GN=CTNNA1 PE=1 SV=1 (CTNA1_HUMAN) | CTNA1 [3] | 100 | 0.00011 | 100% | 100% | 100% | 100% | 100% | 100% | 100% | 100% | 100% | 100% | 100% | 100% |
| 40 | Cluster of Guanine nucleotide-binding protein G(i) subunit alpha-2 OS=Homo sapiens GN=GNAI2 PE=1 SV=3 (GNAI2_HUMAN) | GNAI2 [8] | 40 | 0.015 | 100% | 100% | 100% | 100% | 100% | 100% | 100% | 100% | 100% | 100% | 100% | 100% |
| 41 | Cluster of Plasma membrane calcium-transporting ATPase 4 OS=Homo sapiens GN=ATP2B4 PE=1 SV=2 (AT2B4_HUMAN) | AT2B4 [4] | 138 | 0.0048 | 100% | 100% | 100% | 100% | 100% | 100% | 100% | 100% | 100% | 100% | 100% | 100% |
| 42 | 5'-nucleotidase OS=Homo sapiens GN=NT5E PE=1 SV=1 | 5NTD | 63 | < 0.00010 | 100% | 100% | 100% | 100% | 100% | 100% | 100% | 100% | 100% | 100% | 100% | 100% |
| 43 | ATP synthase subunit beta, mitochondrial OS=Homo sapiens GN=ATP5B PE=1 SV=3 | ATPB | 57 | 0.00016 | 100% | 100% | 100% | 100% | 100% | 100% | 100% | 100% | 100% | 100% | 100% | 100% |
| 44 | Cluster of Sodium/potassium-transporting ATPase subunit alpha-1 OS=Homo sapiens GN=ATP1A1 PE=1 SV=1 (AT1A1_HUMAN) | AT1A1 [6] | 113 | 0.013 | 100% | 100% | 100% | 100% | 100% | 100% | 100% | 100% | 100% | 100% | 100% | 100% |
| 45 | Cluster of Clathrin heavy chain 1 OS=Homo sapiens GN=CLTC PE=1 SV=5 (CLH1_HUMAN) | CLH1 [2] | 192 | 0.0038 | 100% | 100% | 100% | 100% | 100% | 100% | 100% | 100% | 100% | 100% | 100% | 100% |
| 46 | Protein disulfide-isomerase OS=Homo sapiens GN=P4HB PE=1 SV=3 | PDIA1 | 57 | 0.0094 | 100% | 100% | 100% | 100% | 100% | 100% | 100% | 100% | 100% | 100% | 100% | 100% |
| 47 | CD166 antigen OS=Homo sapiens GN=ALCAM PE=1 SV=2 | CD166 | 65 | 0.0036 | 100% | 100% | 100% | 100% | 100% | 100% | 100% | 100% | 100% | 100% | 100% | 100% |
| 48 | Cluster of Catenin beta-1 OS=Homo sapiens GN=CTNNB1 PE=1 SV=1 (CTNB1_HUMAN) | CTNB1 [2] | 85 | 0.0015 | 100% | 100% | 100% | 100% | 100% | 100% | 100% | 100% | 100% | 100% | 100% | 100% |
| 49 | Catenin delta-1 OS=Homo sapiens GN=CTNND1 PE=1 SV=1 | CTND1 | 108 | 0.011 | 100% | 100% | 100% | 100% | 100% | 100% | 100% | 100% | 100% | 100% | 100% | 100% |
| 50 | Aspartyl/asparaginyl beta-hydroxylase OS=Homo sapiens GN=ASPH PE=1 SV=3 | ASPH | 86 | 0.033 | 100% | 100% | 100% | 100% | 100% | 100% | 100% | 100% | 100% | 100% | 100% | 100% |
| 51 | Cluster of Heat shock cognate 71 kDa protein OS=Homo sapiens GN=HSPA8 PE=1 SV=1 (HSP7C_HUMAN) | HSP7C [2] | 71 | 0.027 | 100% | 100% | 100% | 100% | 100% | 100% | 100% | 100% | 100% | 100% | 100% | 100% |
| 52 | Integrin alpha-3 OS=Homo sapiens GN=ITGA3 PE=1 SV=5 | ITA3 | 117 | < 0.00010 | 100% | 100% | 100% | 100% | 100% | 100% | 100% | 100% | 100% | 100% | 100% | 100% |
| 53 | Spectrin beta chain, non-erythrocytic 1 OS=Homo sapiens GN=SPTBN1 PE=1 SV=2 | SPTB2 | 275 | 0.0027 | 100% | 100% | 100% | 100% | 100% | 100% | 100% | 100% | 100% | 100% | 100% | 100% |
| 54 | Golgi apparatus protein 1 OS=Homo sapiens GN=GLG1 PE=1 SV=2 | GSLG1 | 135 | 0.00018 | 100% | 100% | 100% | 100% | 100% | 100% | 100% | 100% | 100% | 100% | 100% | 100% |
| 55 | Aminopeptidase N OS=Homo sapiens GN=ANPEP PE=1 SV=4 | AMPN | 110 | < 0.00010 | 100% | 100% | 100% | 100% | 100% | 100% | 100% | 100% | 100% | 100% | 100% | 100% |
| 56 | ATP synthase subunit alpha, mitochondrial OS=Homo sapiens GN=ATP5A1 PE=1 SV=1 | ATPA | 60 | < 0.00010 | 100% | 100% | 100% | 100% | 100% | 100% | 100% | 100% | 100% | 100% | 100% | 100% |
| 57 | Cluster of Alpha-actinin-1 OS=Homo sapiens GN=ACTN1 PE=1 SV=2 (ACTN1_HUMAN) | ACTN1 [4] | 103 | 0.02 | 100% | 100% | 100% | 100% | 100% | 100% | 100% | 100% | 100% | 100% | 100% | 100% |
| 58 | Flotillin-2 OS=Homo sapiens GN=FLOT2 PE=1 SV=2 | FLOT2 | 47 | 0.0012 | 100% | 100% | 100% | 100% | 100% | 100% | 100% | 100% | 100% | 100% | 100% | 100% |
| 59 | Caveolin-1 OS=Homo sapiens GN=CAV1 PE=1 SV=4 | CAV1 | 20 | < 0.00010 | 100% | 100% | 100% | 100% | 100% | 100% | 100% | 100% | 100% | 100% | 100% | 100% |
| 60 | Integrin alpha-5 OS=Homo sapiens GN=ITGA5 PE=1 SV=2 | ITA5 | 115 | 0.042 | 100% | 100% | 100% | 100% | 100% | 100% | 100% | 100% | 100% | 100% | 100% | 100% |
| 61 | Polymerase I and transcript release factor OS=Homo sapiens GN=PTRF PE=1 SV=1 | PTRF | 43 | 0.00056 | 100% | 100% | 100% | 100% | 100% | 100% | 100% | 100% | 100% | 100% | 100% | 100% |
| 62 | Cluster of Talin-1 OS=Homo sapiens GN=TLN1 PE=1 SV=3 (TLN1_HUMAN) | TLN1 [2] | 270 | 0.067 | 100% | 100% | 100% | 100% | 100% | 100% | 100% | 100% | 100% | 100% | 100% | 100% |
| 63 | CD44 antigen OS=Homo sapiens GN=CD44 PE=1 SV=3 | CD44 | 82 | 0.00021 | 100% | 100% | 100% | 100% | 100% | 100% | 100% | 100% | 100% | 100% | 100% | 100% |
| 64 | Flotillin-1 OS=Homo sapiens GN=FLOT1 PE=1 SV=3 | FLOT1 | 47 | 0.0049 | 100% | 100% | 100% | 100% | 100% | 100% | 100% | 100% | 100% | 100% | 100% | 100% |
| 65 | Cation-independent mannose-6-phosphate receptor OS=Homo sapiens GN=IGF2R PE=1 SV=3 | MPRI | 274 | 0.013 | 100% | 100% | 100% | 100% | 100% | 100% | 100% | 100% | 100% | 100% | 100% | 100% |
| 66 | Cluster of Platelet-derived growth factor receptor beta OS=Homo sapiens GN=PDGFRB PE=1 SV=1 (PGFRB_HUMAN) | PGFRB [8] | 124 | 0.00052 | 100% | 100% | 100% | 100% | 100% | 100% | 100% | 100% | 100% | 100% | 100% | 100% |
| 67 | Transmembrane emp24 domain-containing protein 10 OS=Homo sapiens GN=TMED10 PE=1 SV=2 | TMEDA | 25 | 0.16 | 100% | 100% | 100% | 100% | 100% | 100% | 100% | 100% | 100% | 100% | 100% | 100% |
| 68 | Integrin alpha-V OS=Homo sapiens GN=ITGAV PE=1 SV=2 | ITAV | 116 | 0.00055 | 100% | 100% | 100% | 100% | 100% | 100% | 100% | 100% | 100% | 100% | 100% | 100% |
| 69 | Cluster of Pyruvate kinase PKM OS=Homo sapiens GN=PKM PE=1 SV=4 (KPYM_HUMAN) | KPYM [2] | 58 | 0.0014 | 100% | 100% | 100% | 100% | 100% | 100% | 100% | 100% | 100% | 100% | 100% | 100% |
| 70 | 4F2 cell-surface antigen heavy chain OS=Homo sapiens GN=SLC3A2 PE=1 SV=3 | 4F2 | 68 | 0.03 | 100% | 100% | 100% | 100% | 100% | 100% | 100% | 100% | 100% | 100% | 100% | 100% |
| 71 | Cluster of V-type proton ATPase subunit B, brain isoform OS=Homo sapiens GN=ATP6V1B2 PE=1 SV=3 (VATB2_HUMAN) | VATB2 [2] | 57 | 0.01 | 100% | 100% | 100% | 100% | 100% | 100% | 100% | 100% | 100% | 100% | 100% | 100% |
| 72 | EH domain-containing protein 2 OS=Homo sapiens GN=EHD2 PE=1 SV=2 | EHD2 | 61 | 0.00046 | 100% | 100% | 100% | 100% | 100% | 100% | 100% | 100% | 100% | 100% | 100% | 100% |
| 73 | X-ray repair cross-complementing protein 5 OS=Homo sapiens GN=XRCC5 PE=1 SV=3 | XRCC5 | 83 | 0.98 | 100% | 100% | 100% | 100% | 100% | 100% | 100% | 100% | 100% | 100% | 100% | 100% |
| 74 | V-type proton ATPase catalytic subunit A OS=Homo sapiens GN=ATP6V1A PE=1 SV=2 | VATA | 68 | 0.031 | 100% | 100% | 100% | 100% | 100% | 100% | 100% | 100% | 100% | 100% | 100% | 100% |
| 75 | Transferrin receptor protein 1 OS=Homo sapiens GN=TFRC PE=1 SV=2 | TFR1 | 85 | 0.0069 | 100% | 100% | 100% | 100% | 100% | 100% | 100% | 100% | 100% | 100% | 100% | 100% |
| 76 | Cluster of Guanine nucleotide-binding protein subunit alpha-11 OS=Homo sapiens GN=GNA11 PE=1 SV=2 (GNA11_HUMAN) | GNA11 [6] | 42 | 0.14 | 100% | 100% | 100% | 100% | 100% | 100% | 100% | 100% | 100% | 100% | 100% | 100% |
| 77 | Cluster of Ubiquitin-40S ribosomal protein S27a OS=Homo sapiens GN=RPS27A PE=1 SV=2 (RS27A_HUMAN) | RS27A [3] | 18 | 0.075 | 100% | 100% | 100% | 100% | 100% | 100% | 100% | 100% | 100% | 100% | 100% | 100% |
| 78 | Cluster of Ras-related protein Rab-2A OS=Homo sapiens GN=RAB2A PE=1 SV=1 (RAB2A_HUMAN) | RAB2A [2] | 24 | 0.023 | 100% | 100% | 100% | 100% | 100% | 100% | 100% | 100% | 100% | 100% | 100% | 100% |
| 79 | Cluster of AP-2 complex subunit beta OS=Homo sapiens GN=AP2B1 PE=1 SV=1 (AP2B1_HUMAN) | AP2B1 [2] | 105 | 0.77 | 100% | 100% | 100% | 100% | 100% | 100% | 100% | 100% | 100% | 100% | 100% | 100% |
| 80 | Ras GTPase-activating-like protein IQGAP1 OS=Homo sapiens GN=IQGAP1 PE=1 SV=1 | IQGA1 | 189 | 0.086 | 100% | 100% | 100% | 100% | 100% | 100% | 100% | 100% | 100% | 100% | 100% | 100% |
| 81 | Erythrocyte band 7 integral membrane protein OS=Homo sapiens GN=STOM PE=1 SV=3 | STOM | 32 | 0.01 | 100% | 100% | 100% | 100% | 100% | 100% | 100% | 100% | 100% | 100% | 100% | 100% |
| 82 | Catechol O-methyltransferase OS=Homo sapiens GN=COMT PE=1 SV=2 | COMT | 30 | 0.0097 | 100% | 100% | 100% | 100% | 100% | 100% | 100% | 100% | 100% | 100% | 100% | 100% |
| 83 | Voltage-dependent anion-selective channel protein 1 OS=Homo sapiens GN=VDAC1 PE=1 SV=2 | VDAC1 | 31 | 0.095 | 100% | 100% | 100% | 100% | 100% | 100% | 100% | 100% | 100% | 100% | 100% | 100% |
| 84 | Extended synaptotagmin-2 OS=Homo sapiens GN=ESYT2 PE=1 SV=1 | ESYT2 | 102 | 0.013 | 100% | 100% | 100% | 100% | 100% | 100% | 100% | 100% | 100% | 100% | 100% | 100% |
| 85 | Vesicle-associated membrane protein-associated protein A OS=Homo sapiens GN=VAPA PE=1 SV=3 | VAPA | 28 | 0.0033 | 100% | 100% | 100% | 100% | 100% | 100% | 100% | 100% | 100% | 100% | 100% | 100% |
| 86 | V-type proton ATPase 116 kDa subunit a isoform 3 OS=Homo sapiens GN=TCIRG1 PE=1 SV=3 | VPP3 | 93 | 0.00023 | 100% | 100% | 100% | 100% | 100% | 100% | 100% | 100% | 100% | 100% | 100% | 100% |
| 87 | Cluster of Unconventional myosin-Ib OS=Homo sapiens GN=MYO1B PE=1 SV=3 (MYO1B_HUMAN) | MYO1B [2] | 132 | < 0.00010 | 100% | 100% | 100% | 100% | 100% | 100% | 100% | 100% | 100% | 100% | 100% | 100% |
| 88 | Cluster of Ephrin type-A receptor 2 OS=Homo sapiens GN=EPHA2 PE=1 SV=2 (EPHA2_HUMAN) | EPHA2 [9] | 108 | < 0.00010 | 100% | 100% | 100% | 100% | 100% | 100% | 100% | 100% | 100% | 100% | 100% | 100% |
| 89 | Protein disulfide-isomerase A6 OS=Homo sapiens GN=PDIA6 PE=1 SV=1 | PDIA6 | 48 | 0.21 | 100% | 100% | 100% | 100% | 100% | 100% | 100% | 100% | 100% | 100% | 100% | 100% |
| 90 | Utrophin OS=Homo sapiens GN=UTRN PE=1 SV=2 | UTRO | 394 | 0.0077 | 100% | 100% | 100% | 100% | 100% | 100% | 100% | 100% | 100% | 100% | 100% | 100% |
| 91 | Desmoplakin OS=Homo sapiens GN=DSP PE=1 SV=3 | DESP | 332 | < 0.00010 | 100% | 100% | 100% | 95% | 100% | 100% | 100% | 100% | 100% | 100% | 100% | 100% |
| 92 | Cluster of Plexin-B2 OS=Homo sapiens GN=PLXNB2 PE=1 SV=3 (PLXB2_HUMAN) | PLXB2 [3] | 205 | 0.58 | 100% | 100% | 100% | 100% | 100% | 100% | 100% | 100% | 100% | 100% | 100% | 100% |
| 93 | Cluster of Alpha-enolase OS=Homo sapiens GN=ENO1 PE=1 SV=2 (ENOA_HUMAN) | ENOA [3] | 47 | 0.0014 | 100% | 100% | 100% | 100% | 100% | 100% | 100% | 100% | 100% | 100% | 100% | 100% |
| 94 | Lysosome-associated membrane glycoprotein 1 OS=Homo sapiens GN=LAMP1 PE=1 SV=3 | LAMP1 | 45 | 0.00011 | 100% | 100% | 100% | 100% | 100% | 100% | 100% | 100% | 100% | 100% | 100% | 100% |
| 95 | PRA1 family protein 3 OS=Homo sapiens GN=ARL6IP5 PE=1 SV=1 | PRAF3 | 22 | 0.16 | 100% | 100% | 100% | 100% | 100% | 100% | 100% | 100% | 100% | 100% | 100% | 100% |
| 96 | Prenylcysteine oxidase 1 OS=Homo sapiens GN=PCYOX1 PE=1 SV=3 | PCYOX | 57 | 0.029 | 100% | 100% | 100% | 100% | 100% | 100% | 100% | 100% | 100% | 100% | 100% | 100% |
| 97 | Cell division control protein 42 homolog OS=Homo sapiens GN=CDC42 PE=1 SV=2 | CDC42 | 21 | 0.47 | 100% | 100% | 100% | 100% | 100% | 100% | 100% | 100% | 100% | 100% | 100% | 100% |
| 98 | LIM domain and actin-binding protein 1 OS=Homo sapiens GN=LIMA1 PE=1 SV=1 | LIMA1 | 85 | 0.0014 | 100% | 100% | 100% | 100% | 100% | 100% | 100% | 100% | 100% | 100% | 100% | 100% |
| 99 | Glypican-1 OS=Homo sapiens GN=GPC1 PE=1 SV=2 | GPC1 | 62 | 0.0016 | 100% | 100% | 100% | 100% | 100% | 100% | 100% | 100% | 100% | 100% | 100% | 100% |
| 100 | Basigin OS=Homo sapiens GN=BSG PE=1 SV=2 | BASI | 42 | 0.0021 | 100% | 100% | 100% | 100% | 100% | 100% | 100% | 100% | 100% | 100% | 100% | 100% |
| 101 | Prohibitin OS=Homo sapiens GN=PHB PE=1 SV=1 | PHB | 30 | 0.00034 | 100% | 100% | 100% | 100% | 100% | 100% | 100% | 100% | 100% | 100% | 100% | 100% |
| 102 | Microtubule-associated protein 1B OS=Homo sapiens GN=MAP1B PE=1 SV=2 | MAP1B | 271 | 0.003 | 100% | 100% | 100% | 100% | 100% | 100% | 100% | 100% | 100% | 100% | 100% | 100% |
| 103 | Syntenin-1 OS=Homo sapiens GN=SDCBP PE=1 SV=1 | SDCB1 | 32 | 0.00021 | 100% | 100% | 100% | 100% | 100% | 100% | 100% | 100% | 100% | 100% | 100% | 100% |
| 104 | Cluster of Ras-related protein Rap-1b-like protein OS=Homo sapiens PE=2 SV=1 (RP1BL_HUMAN) | RP1BL [2] | 21 | 0.088 | 100% | 100% | 100% | 100% | 100% | 100% | 100% | 100% | 100% | 100% | 100% | 100% |
| 105 | Neutral amino acid transporter B(0) OS=Homo sapiens GN=SLC1A5 PE=1 SV=2 | AAAT | 57 | 0.014 | 100% | 100% | 100% | 100% | 100% | 100% | 100% | 100% | 100% | 100% | 100% | 100% |
| 106 | T-complex protein 1 subunit gamma OS=Homo sapiens GN=CCT3 PE=1 SV=4 | TCPG | 61 | 0.037 | 100% | 100% | 100% | 100% | 100% | 100% | 100% | 100% | 100% | 100% | 100% | 100% |
| 107 | CD97 antigen OS=Homo sapiens GN=CD97 PE=1 SV=4 | CD97 | 92 | 0.18 | 100% | 100% | 100% | 100% | 100% | 100% | 100% | 100% | 100% | 100% | 100% | 100% |
| 108 | Cluster of Cadherin-2 OS=Homo sapiens GN=CDH2 PE=1 SV=4 (CADH2_HUMAN) | CADH2 [2] | 100 | 0.0011 | 100% | 100% | 100% | 100% | 100% | 100% | 100% | 100% | 100% | 100% | 100% | 100% |
| 109 | Cluster of Ras-related C3 botulinum toxin substrate 1 OS=Homo sapiens GN=RAC1 PE=1 SV=1 (RAC1_HUMAN) | RAC1 [2] | 21 | 0.64 | 100% | 100% | 100% | 100% | 100% | 100% | 100% | 100% | 100% | 100% | 100% | 100% |
| 110 | Filamin-B OS=Homo sapiens GN=FLNB PE=1 SV=2 | FLNB | 278 | 0.022 | 100% | 100% | 100% | 100% | 100% | 100% | 100% | 100% | 100% | 100% | 100% | 100% |
| 111 | Ras-related protein Rab-14 OS=Homo sapiens GN=RAB14 PE=1 SV=4 | RAB14 | 24 | 0.012 | 100% | 100% | 100% | 100% | 100% | 100% | 100% | 100% | 100% | 100% | 100% | 100% |
| 112 | Beta-2-microglobulin OS=Homo sapiens GN=B2M PE=1 SV=1 | B2MG | 14 | 0.011 | 100% | 100% | 100% | 100% | 100% | 100% | 100% | 100% | 100% | 100% | 100% | 100% |
| 113 | Sodium/potassium-transporting ATPase subunit beta-3 OS=Homo sapiens GN=ATP1B3 PE=1 SV=1 | AT1B3 | 32 | 0.056 | 100% | 100% | 100% | 100% | 100% | 100% | 100% | 100% | 100% | 100% | 100% | 100% |
| 114 | AP-2 complex subunit alpha-1 OS=Homo sapiens GN=AP2A1 PE=1 SV=3 | AP2A1 | 108 | 0.1 | 100% | 100% | 100% | 100% | 100% | 100% | 100% | 100% | 100% | 100% | 100% | 100% |
| 115 | Cluster of Transforming protein RhoA OS=Homo sapiens GN=RHOA PE=1 SV=1 (RHOA_HUMAN) | RHOA [3] | 22 | 0.27 | 100% | 100% | 100% | 100% | 100% | 100% | 100% | 100% | 100% | 100% | 100% | 100% |
| 116 | Coronin-1C OS=Homo sapiens GN=CORO1C PE=1 SV=1 | COR1C | 53 | 0.048 | 100% | 100% | 100% | 100% | 100% | 100% | 100% | 100% | 100% | 100% | 100% | 100% |
| 117 | Annexin A1 OS=Homo sapiens GN=ANXA1 PE=1 SV=2 | ANXA1 | 39 | 0.00091 | 100% | 100% | 100% | 100% | 100% | 100% | 100% | 100% | 100% | 100% | 100% | 100% |
| 118 | Thy-1 membrane glycoprotein OS=Homo sapiens GN=THY1 PE=1 SV=2 | THY1 | 18 | 0.37 | 100% | 100% | 100% | 100% | 100% | 100% | 100% | 100% | 100% | 100% | 100% | 100% |
| 119 | Integrin beta-5 OS=Homo sapiens GN=ITGB5 PE=1 SV=1 | ITB5 | 88 | 0.042 | 100% | 100% | 100% | 100% | 100% | 100% | 100% | 100% | 100% | 100% | 100% | 100% |
| 120 | Vesicle-fusing ATPase OS=Homo sapiens GN=NSF PE=1 SV=3 | NSF | 83 | 0.11 | 100% | 100% | 100% | 100% | 100% | 100% | 100% | 100% | 100% | 100% | 100% | 100% |
| 121 | Protein disulfide-isomerase TMX3 OS=Homo sapiens GN=TMX3 PE=1 SV=2 | TMX3 | 52 | 0.83 | 100% | 100% | 100% | 100% | 100% | 100% | 100% | 100% | 100% | 100% | 100% | 100% |
| 122 | Intercellular adhesion molecule 1 OS=Homo sapiens GN=ICAM1 PE=1 SV=2 | ICAM1 | 58 | 0.0011 | 99% | 100% | 100% | 100% | 100% | 100% | 100% | 100% | 100% | 100% | 100% | 100% |
| 123 | Niemann-Pick C1 protein OS=Homo sapiens GN=NPC1 PE=1 SV=2 | NPC1 | 142 | 0.0013 | 100% | 100% | 100% | 100% | 100% | 100% | 100% | 100% | 100% | 100% | 100% | 100% |
| 124 | CD109 antigen OS=Homo sapiens GN=CD109 PE=1 SV=2 | CD109 | 162 | 0.018 | 100% | 100% | 100% | 100% | 100% | 100% | 100% | 100% | 100% | 100% | 100% | 100% |
| 125 | Guanine nucleotide-binding protein G(I)/G(S)/G(T) subunit beta-1 OS=Homo sapiens GN=GNB1 PE=1 SV=3 | GBB1 | 37 | 0.054 | 100% | 100% | 100% | 100% | 100% | 100% | 100% | 100% | 100% | 100% | 100% | 100% |
| 126 | Matrix metalloproteinase-14 OS=Homo sapiens GN=MMP14 PE=1 SV=3 | MMP14 | 66 | 0.018 | 100% | 100% | 100% | 100% | 100% | 100% | 100% | 100% | 100% | 100% | 100% | 100% |
| 127 | Ras-related protein Rab-5C OS=Homo sapiens GN=RAB5C PE=1 SV=2 | RAB5C | 23 | 0.91 | 100% | 100% | 100% | 100% | 100% | 100% | 100% | 100% | 100% | 100% | 100% | 100% |
| 128 | Rho guanine nucleotide exchange factor 2 OS=Homo sapiens GN=ARHGEF2 PE=1 SV=4 | ARHG2 | 112 | 0.0016 | 100% | 100% | 100% | 100% | 100% | 100% | 100% | 100% | 100% | 100% | 100% | 100% |
| 129 | Stimulator of interferon genes protein OS=Homo sapiens GN=TMEM173 PE=1 SV=1 | STING | 42 | 0.0034 | 100% | 100% | 100% | 100% | 100% | 100% | 100% | 100% | 100% | 100% | 100% | 100% |
| 130 | Microtubule-associated protein 4 OS=Homo sapiens GN=MAP4 PE=1 SV=3 | MAP4 | 121 | 0.054 | 100% | 100% | 100% | 100% | 100% | 100% | 100% | 100% | 100% | 100% | 100% | 100% |
| 131 | Tyrosine-protein kinase receptor UFO OS=Homo sapiens GN=AXL PE=1 SV=3 | UFO | 98 | 0.0052 | 100% | 100% | 100% | 100% | 100% | 100% | 100% | 100% | 100% | 100% | 100% | 100% |
| 132 | Prostaglandin F2 receptor negative regulator OS=Homo sapiens GN=PTGFRN PE=1 SV=2 | FPRP | 99 | 0.02 | 100% | 100% | 100% | 100% | 100% | 100% | 100% | 100% | 100% | 100% | 100% | 100% |
| 133 | Carboxypeptidase D OS=Homo sapiens GN=CPD PE=1 SV=2 | CBPD | 153 | 0.52 | 100% | 100% | 100% | 100% | 100% | 100% | 100% | 100% | 100% | 100% | 100% | 100% |
| 134 | Epidermal growth factor receptor OS=Homo sapiens GN=EGFR PE=1 SV=2 | EGFR | 134 | 0.015 | 100% | 100% | 100% | 100% | 100% | 100% | 100% | 100% | 100% | 100% | 100% | 100% |
| 135 | Polycystin-2 OS=Homo sapiens GN=PKD2 PE=1 SV=3 | PKD2 | 110 | 0.00024 | 100% | 100% | 100% | 100% | 100% | 100% | 100% | 100% | 100% | 100% | 100% | 100% |
| 136 | Phosphatidylinositol 4-kinase type 2-alpha OS=Homo sapiens GN=PI4K2A PE=1 SV=1 | P4K2A | 54 | 0.019 | 100% | 100% | 100% | 100% | 100% | 100% | 100% | 100% | 100% | 100% | 100% | 100% |
| 137 | Cluster of Ras-related protein Rab-8A OS=Homo sapiens GN=RAB8A PE=1 SV=1 (RAB8A_HUMAN) | RAB8A [2] | 24 | 0.011 | 100% | 100% | 100% | 100% | 100% | 100% | 100% | 100% | 100% | 100% | 100% | 100% |
| 138 | Sec1 family domain-containing protein 1 OS=Homo sapiens GN=SCFD1 PE=1 SV=4 | SCFD1 | 72 | 0.0041 | 100% | 100% | 100% | 100% | 100% | 100% | 100% | 100% | 100% | 100% | 100% | 100% |
| 139 | Cluster of Protocadherin Fat 1 OS=Homo sapiens GN=FAT1 PE=1 SV=2 (FAT1_HUMAN) | FAT1 [2] | 506 | < 0.00010 | 100% | 100% | 100% | 100% | 100% | 100% | 100% | 100% | 100% | 100% | 100% | 100% |
| 140 | Poliovirus receptor OS=Homo sapiens GN=PVR PE=1 SV=2 | PVR | 45 | < 0.00010 | 100% | 100% | 100% | 100% | 100% | 100% | 100% | 100% | 100% | 100% | 100% | 100% |
| 141 | Heme oxygenase 2 OS=Homo sapiens GN=HMOX2 PE=1 SV=2 | HMOX2 | 36 | 0.83 | 100% | 100% | 100% | 100% | 100% | 100% | 100% | 100% | 100% | 100% | 100% | 100% |
| 142 | Minor histocompatibility antigen H13 OS=Homo sapiens GN=HM13 PE=1 SV=1 | HM13 | 41 | 0.47 | 100% | 100% | 100% | 100% | 100% | 100% | 100% | 100% | 100% | 100% | 100% | 100% |
| 143 | Syntaxin-7 OS=Homo sapiens GN=STX7 PE=1 SV=4 | STX7 | 30 | 0.15 | 100% | 100% | 100% | 100% | 100% | 100% | 100% | 100% | 100% | 100% | 100% | 100% |
| 144 | Septin-2 OS=Homo sapiens GN=SEPT2 PE=1 SV=1 | SEPT2 | 41 | 0.0036 | 100% | 100% | 100% | 100% | 100% | 100% | 100% | 100% | 100% | 100% | 100% | 100% |
| 145 | Neuroplastin OS=Homo sapiens GN=NPTN PE=1 SV=2 | NPTN | 44 | 0.66 | 100% | 100% | 100% | 100% | 100% | 100% | 100% | 100% | 100% | 100% | 100% | 100% |
| 146 | ATP-citrate synthase OS=Homo sapiens GN=ACLY PE=1 SV=3 | ACLY | 121 | 0.0078 | 100% | 100% | 100% | 100% | 100% | 100% | 100% | 100% | 100% | 100% | 100% | 100% |
| 147 | Brain acid soluble protein 1 OS=Homo sapiens GN=BASP1 PE=1 SV=2 | BASP1 | 23 | 0.097 | 100% | 100% | 100% | 100% | 100% | 100% | 100% | 100% | 100% | 100% | 100% | 100% |
| 148 | Ragulator complex protein LAMTOR1 OS=Homo sapiens GN=LAMTOR1 PE=1 SV=2 | LTOR1 | 18 | 0.00031 | 100% | 100% | 100% | 100% | 100% | 100% | 100% | 100% | 100% | 100% | 100% | 100% |
| 149 | Cluster of Ras-related protein Ral-B OS=Homo sapiens GN=RALB PE=1 SV=1 (RALB_HUMAN) | RALB [2] | 23 | 0.014 | 100% | 100% | 100% | 100% | 100% | 100% | 100% | 100% | 100% | 100% | 100% | 100% |
| 150 | Raftlin OS=Homo sapiens GN=RFTN1 PE=1 SV=4 | RFTN1 | 63 | 0.017 | 100% | 100% | 100% | 100% | 100% | 100% | 100% | 100% | 100% | 100% | 100% | 100% |
| 151 | Cadherin-13 OS=Homo sapiens GN=CDH13 PE=1 SV=1 | CAD13 | 78 | < 0.00010 | 100% | 100% | 100% | 100% | 95% | 18% | 100% | 100% | 100% | 98% | 95% | 100% |
| 152 | Vinculin OS=Homo sapiens GN=VCL PE=1 SV=4 | VINC | 124 | 0.49 | 100% | 100% | 100% | 100% | 100% | 100% | 100% | 100% | 100% | 100% | 100% | 100% |
| 153 | Cluster of Solute carrier family 12 member 4 OS=Homo sapiens GN=SLC12A4 PE=1 SV=2 (S12A4_HUMAN) | S12A4 [3] | 121 | 0.18 | 0 | 100% | 100% | 100% | 100% | 100% | 100% | 100% | 100% | 100% | 100% | 100% |
| 154 | Galectin-3 OS=Homo sapiens GN=LGALS3 PE=1 SV=5 | LEG3 | 26 | 0.0035 | 100% | 100% | 100% | 100% | 100% | 100% | 100% | 100% | 100% | 100% | 100% | 100% |
| 155 | Erlin-2 OS=Homo sapiens GN=ERLIN2 PE=1 SV=1 | ERLN2 | 38 | 0.47 | 100% | 100% | 100% | 100% | 100% | 100% | 100% | 100% | 100% | 100% | 100% | 100% |
| 156 | Cluster of Vesicle-associated membrane protein 3 OS=Homo sapiens GN=VAMP3 PE=1 SV=3 (VAMP3_HUMAN) | VAMP3 [2] | 11 | 0.047 | 100% | 100% | 100% | 100% | 100% | 100% | 100% | 100% | 100% | 100% | 100% | 100% |
| 157 | Cluster of Ras-related protein Rab-11B OS=Homo sapiens GN=RAB11B PE=1 SV=4 (RB11B_HUMAN) | RB11B [3] | 24 | 0.19 | 100% | 100% | 100% | 100% | 100% | 100% | 100% | 100% | 100% | 100% | 100% | 100% |
| 158 | Neutral amino acid transporter A OS=Homo sapiens GN=SLC1A4 PE=1 SV=1 | SATT | 56 | 0.94 | 100% | 100% | 100% | 100% | 100% | 100% | 100% | 100% | 100% | 100% | 100% | 100% |
| 159 | Alpha-2-macroglobulin receptor-associated protein OS=Homo sapiens GN=LRPAP1 PE=1 SV=1 | AMRP | 41 | 0.015 | 100% | 100% | 100% | 100% | 100% | 100% | 100% | 100% | 100% | 100% | 100% | 100% |
| 160 | Constitutive coactivator of PPAR-gamma-like protein 1 OS=Homo sapiens GN=FAM120A PE=1 SV=2 | F120A | 122 | 0.002 | 100% | 100% | 100% | 100% | 100% | 100% | 100% | 100% | 100% | 100% | 100% | 100% |
| 161 | Discoidin domain-containing receptor 2 OS=Homo sapiens GN=DDR2 PE=1 SV=2 | DDR2 | 97 | 0.026 | 100% | 100% | 100% | 100% | 100% | 100% | 100% | 100% | 100% | 100% | 100% | 100% |
| 162 | Ras-related protein Rab-18 OS=Homo sapiens GN=RAB18 PE=1 SV=1 | RAB18 | 23 | 0.15 | 100% | 100% | 100% | 100% | 100% | 100% | 100% | 100% | 100% | 100% | 100% | 100% |
| 163 | Vasorin OS=Homo sapiens GN=VASN PE=1 SV=1 | VASN | 72 | 0.014 | 100% | 100% | 100% | 100% | 100% | 100% | 100% | 100% | 100% | 100% | 100% | 100% |
| 164 | Fermitin family homolog 2 OS=Homo sapiens GN=FERMT2 PE=1 SV=1 | FERM2 | 78 | 0.082 | 100% | 100% | 100% | 100% | 100% | 100% | 100% | 100% | 100% | 100% | 100% | 100% |
| 165 | Guanine nucleotide-binding protein G(I)/G(S)/G(O) subunit gamma-12 OS=Homo sapiens GN=GNG12 PE=1 SV=3 | GBG12 | 8 | 0.042 | 100% | 100% | 100% | 100% | 100% | 100% | 100% | 100% | 100% | 100% | 100% | 100% |
| 166 | Nucleobindin-2 OS=Homo sapiens GN=NUCB2 PE=1 SV=2 | NUCB2 | 50 | 0.00037 | 100% | 100% | 100% | 100% | 100% | 100% | 100% | 100% | 100% | 100% | 100% | 100% |
| 167 | Multidrug resistance-associated protein 1 OS=Homo sapiens GN=ABCC1 PE=1 SV=3 | MRP1 | 172 | 0.0046 | 100% | 100% | 100% | 100% | 100% | 100% | 100% | 100% | 100% | 100% | 100% | 100% |
| 168 | Sulfhydryl oxidase 2 OS=Homo sapiens GN=QSOX2 PE=1 SV=3 | QSOX2 | 78 | 0.016 | 95% | 100% | 100% | 100% | 100% | 100% | 100% | 100% | 100% | 100% | 100% | 100% |
| 169 | Microtubule-actin cross-linking factor 1, isoforms 1/2/3/5 OS=Homo sapiens GN=MACF1 PE=1 SV=4 | MACF1 | 838 | 0.075 | 100% | 80% | 100% | 100% | 100% | 100% | 98% | 89% | 88% | 100% | 100% | 61% |
| 170 | NADH-cytochrome b5 reductase 1 OS=Homo sapiens GN=CYB5R1 PE=1 SV=1 | NB5R1 | 34 | 0.015 | 100% | 100% | 100% | 100% | 100% | 100% | 100% | 100% | 100% | 100% | 100% | 100% |
| 171 | Palladin OS=Homo sapiens GN=PALLD PE=1 SV=3 | PALLD | 151 | 0.14 | 100% | 100% | 100% | 100% | 100% | 100% | 100% | 100% | 100% | 100% | 100% | 100% |
| 172 | 60 kDa heat shock protein, mitochondrial OS=Homo sapiens GN=HSPD1 PE=1 SV=2 | CH60 | 61 | < 0.00010 | 100% | 100% | 100% | 100% | 100% | 100% | 100% | 100% | 100% | 100% | 100% | 100% |
| 173 | CD81 antigen OS=Homo sapiens GN=CD81 PE=1 SV=1 | CD81 | 26 | 0.043 | 100% | 100% | 100% | 100% | 100% | 100% | 100% | 100% | 100% | 100% | 100% | 100% |
| 174 | Cell migration-inducing and hyaluronan-binding protein OS=Homo sapiens GN=CEMIP PE=1 SV=2 | CEMIP | 153 | 0.0043 | 100% | 100% | 100% | 0 | 0 | 16% | 100% | 100% | 100% | 92% | 0 | 97% |
| 175 | Long-chain fatty acid transport protein 1 OS=Homo sapiens GN=SLC27A1 PE=2 SV=1 | S27A1 | 71 | 0.033 | 100% | 100% | 100% | 100% | 100% | 100% | 100% | 100% | 100% | 100% | 100% | 100% |
| 176 | Cluster of Ras-related protein Rab-32 OS=Homo sapiens GN=RAB32 PE=1 SV=3 (RAB32_HUMAN) | RAB32 [2] | 25 | 0.016 | 100% | 100% | 100% | 100% | 100% | 100% | 98% | 100% | 100% | 100% | 100% | 100% |
| 177 | Cluster of Calcium/calmodulin-dependent protein kinase type II subunit delta OS=Homo sapiens GN=CAMK2D PE=1 SV=3 (KCC2D_HUMAN) | KCC2D [3] | 56 | 0.14 | 100% | 100% | 100% | 83% | 100% | 100% | 100% | 100% | 100% | 100% | 100% | 100% |
| 178 | ATP synthase subunit O, mitochondrial OS=Homo sapiens GN=ATP5O PE=1 SV=1 | ATPO | 23 | 0.0011 | 100% | 100% | 100% | 100% | 100% | 100% | 100% | 100% | 100% | 100% | 100% | 100% |
| 179 | EH domain-containing protein 1 OS=Homo sapiens GN=EHD1 PE=1 SV=2 | EHD1 | 61 | 0.16 | 100% | 100% | 100% | 100% | 100% | 100% | 100% | 100% | 100% | 100% | 100% | 100% |
| 180 | Monocarboxylate transporter 4 OS=Homo sapiens GN=SLC16A3 PE=1 SV=1 | MOT4 | 49 | 0.33 | 100% | 100% | 100% | 100% | 100% | 100% | 100% | 100% | 100% | 100% | 100% | 100% |
| 181 | Neurogenic locus notch homolog protein 3 OS=Homo sapiens GN=NOTCH3 PE=1 SV=2 | NOTC3 | 244 | 0.00013 | 100% | 100% | 100% | 100% | 100% | 100% | 100% | 100% | 100% | 100% | 100% | 100% |
| 182 | Transient receptor potential cation channel subfamily V member 2 OS=Homo sapiens GN=TRPV2 PE=1 SV=1 | TRPV2 | 86 | 0.055 | 100% | 100% | 100% | 100% | 100% | 100% | 100% | 100% | 100% | 100% | 100% | 100% |
| 183 | Drebrin OS=Homo sapiens GN=DBN1 PE=1 SV=4 | DREB | 71 | 0.16 | 100% | 100% | 100% | 100% | 100% | 100% | 100% | 100% | 100% | 100% | 100% | 100% |
| 184 | AP-2 complex subunit alpha-2 OS=Homo sapiens GN=AP2A2 PE=1 SV=2 | AP2A2 | 104 | 0.15 | 100% | 100% | 100% | 100% | 100% | 100% | 100% | 100% | 100% | 100% | 100% | 100% |
| 185 | Apoptotic chromatin condensation inducer in the nucleus OS=Homo sapiens GN=ACIN1 PE=1 SV=2 | ACINU | 152 | 0.12 | 100% | 100% | 100% | 87% | 100% | 100% | 100% | 100% | 100% | 100% | 100% | 100% |
| 186 | 2',3'-cyclic-nucleotide 3'-phosphodiesterase OS=Homo sapiens GN=CNP PE=1 SV=2 | CN37 | 48 | 0.14 | 100% | 100% | 100% | 100% | 100% | 100% | 100% | 100% | 100% | 100% | 100% | 100% |
| 187 | Myeloid-associated differentiation marker OS=Homo sapiens GN=MYADM PE=1 SV=2 | MYADM | 35 | 0.42 | 100% | 100% | 100% | 100% | 100% | 95% | 100% | 100% | 95% | 99% | 99% | 100% |
| 188 | Phospholipid scramblase 3 OS=Homo sapiens GN=PLSCR3 PE=1 SV=2 | PLS3 | 32 | 0.048 | 100% | 100% | 100% | 100% | 100% | 100% | 100% | 100% | 100% | 100% | 100% | 95% |
| 189 | Seprase OS=Homo sapiens GN=FAP PE=1 SV=5 | SEPR | 88 | 0.3 | 100% | 100% | 100% | 100% | 100% | 100% | 100% | 100% | 100% | 100% | 100% | 100% |
| 190 | Anoctamin-10 OS=Homo sapiens GN=ANO10 PE=1 SV=2 | ANO10 | 76 | 0.18 | 100% | 100% | 100% | 100% | 100% | 100% | 100% | 100% | 100% | 100% | 100% | 100% |
| 191 | AP-2 complex subunit mu OS=Homo sapiens GN=AP2M1 PE=1 SV=2 | AP2M1 | 50 | 0.25 | 100% | 100% | 100% | 100% | 100% | 100% | 100% | 100% | 100% | 12% | 100% | 100% |
| 192 | CD63 antigen OS=Homo sapiens GN=CD63 PE=1 SV=2 | CD63 | 26 | 0.052 | 100% | 100% | 100% | 100% | 100% | 100% | 100% | 100% | 100% | 100% | 100% | 100% |
| 193 | Cluster of Septin-7 OS=Homo sapiens GN=SEPT7 PE=1 SV=2 (SEPT7_HUMAN) | SEPT7 [2] | 51 | 0.0052 | 100% | 100% | 100% | 64% | 0 | 100% | 100% | 100% | 100% | 100% | 100% | 100% |
| 194 | Cluster of Cofilin-1 OS=Homo sapiens GN=CFL1 PE=1 SV=3 (COF1_HUMAN) | COF1 [2] | 19 | 0.27 | 100% | 100% | 100% | 100% | 100% | 100% | 100% | 100% | 100% | 100% | 100% | 100% |
| 195 | Integrin-linked protein kinase OS=Homo sapiens GN=ILK PE=1 SV=2 | ILK | 51 | 0.27 | 100% | 100% | 100% | 100% | 100% | 100% | 100% | 100% | 100% | 100% | 100% | 100% |
| 196 | Cluster of GTPase HRas OS=Homo sapiens GN=HRAS PE=1 SV=1 (RASH_HUMAN) | RASH [2] | 21 | 0.59 | 100% | 100% | 100% | 100% | 100% | 100% | 100% | 100% | 100% | 100% | 100% | 100% |
| 197 | Syntaxin-binding protein 1 OS=Homo sapiens GN=STXBP1 PE=1 SV=1 | STXB1 | 68 | 0.12 | 100% | 100% | 100% | 100% | 100% | 100% | 100% | 95% | 95% | 100% | 100% | 100% |
| 198 | Integrin alpha-11 OS=Homo sapiens GN=ITGA11 PE=1 SV=2 | ITA11 | 133 | 0.0098 | 100% | 100% | 100% | 66% | 100% | 100% | 100% | 100% | 100% | 100% | 100% | 100% |
| 199 | Integrin alpha-1 OS=Homo sapiens GN=ITGA1 PE=1 SV=2 | ITA1 | 131 | 0.71 | 100% | 100% | 100% | 100% | 100% | 100% | 100% | 100% | 100% | 100% | 100% | 100% |
| 200 | Cluster of Fatty aldehyde dehydrogenase OS=Homo sapiens GN=ALDH3A2 PE=1 SV=1 (AL3A2_HUMAN) | AL3A2 [3] | 55 | 0.0074 | 100% | 100% | 100% | 100% | 100% | 100% | 100% | 100% | 100% | 100% | 100% | 100% |
| 201 | 45 kDa calcium-binding protein OS=Homo sapiens GN=SDF4 PE=1 SV=1 | CAB45 | 42 | 0.84 | 100% | 100% | 100% | 100% | 100% | 100% | 100% | 100% | 100% | 100% | 100% | 100% |
| 202 | Lanosterol 14-alpha demethylase OS=Homo sapiens GN=CYP51A1 PE=1 SV=3 | CP51A | 57 | 0.05 | 100% | 100% | 100% | 100% | 100% | 100% | 100% | 100% | 100% | 100% | 100% | 100% |
| 203 | V-type proton ATPase 116 kDa subunit a isoform 1 OS=Homo sapiens GN=ATP6V0A1 PE=2 SV=3 | VPP1 | 96 | 0.48 | 100% | 100% | 100% | 100% | 100% | 100% | 100% | 100% | 100% | 100% | 92% | 100% |
| 204 | Choline transporter-like protein 2 OS=Homo sapiens GN=SLC44A2 PE=1 SV=3 | CTL2 | 80 | 0.0034 | 100% | 100% | 100% | 100% | 100% | 100% | 100% | 100% | 95% | 100% | 100% | 100% |
| 205 | Neurogenic locus notch homolog protein 2 OS=Homo sapiens GN=NOTCH2 PE=1 SV=3 | NOTC2 | 265 | < 0.00010 | 100% | 100% | 100% | 100% | 100% | 100% | 0 | 49% | 72% | 100% | 100% | 100% |
| 206 | Neuropilin-1 OS=Homo sapiens GN=NRP1 PE=1 SV=3 | NRP1 | 103 | 0.002 | 100% | 100% | 100% | 0 | 100% | 13% | 100% | 100% | 100% | 100% | 100% | 100% |
| 207 | Leucyl-cystinyl aminopeptidase OS=Homo sapiens GN=LNPEP PE=1 SV=3 | LCAP | 117 | 0.16 | 100% | 100% | 100% | 100% | 100% | 100% | 100% | 100% | 100% | 100% | 100% | 100% |
| 208 | Syntaxin-binding protein 3 OS=Homo sapiens GN=STXBP3 PE=1 SV=2 | STXB3 | 68 | 0.24 | 100% | 100% | 100% | 100% | 100% | 100% | 100% | 100% | 100% | 100% | 100% | 100% |
| 209 | Disintegrin and metalloproteinase domain-containing protein 9 OS=Homo sapiens GN=ADAM9 PE=1 SV=1 | ADAM9 | 91 | 0.15 | 100% | 100% | 100% | 100% | 100% | 100% | 100% | 100% | 100% | 100% | 100% | 100% |
| 210 | Integrin alpha-6 OS=Homo sapiens GN=ITGA6 PE=1 SV=5 | ITA6 | 127 | < 0.00010 | 100% | 100% | 100% | 100% | 100% | 100% | 0 | 95% | 0 | 100% | 100% | 100% |
| 211 | PDZ and LIM domain protein 5 OS=Homo sapiens GN=PDLIM5 PE=1 SV=5 | PDLI5 | 64 | 0.00034 | 100% | 100% | 100% | 99% | 100% | 100% | 100% | 100% | 100% | 100% | 100% | 100% |
| 212 | Heme oxygenase 1 OS=Homo sapiens GN=HMOX1 PE=1 SV=1 | HMOX1 | 33 | < 0.00010 | 100% | 100% | 100% | 0 | 0 | 95% | 100% | 100% | 100% | 100% | 100% | 100% |
| 213 | 40S ribosomal protein SA OS=Homo sapiens GN=RPSA PE=1 SV=4 | RSSA | 33 | 0.13 | 0 | 100% | 100% | 100% | 100% | 100% | 100% | 100% | 100% | 100% | 62% | 100% |
| 214 | Syntaxin-4 OS=Homo sapiens GN=STX4 PE=1 SV=2 | STX4 | 34 | 0.055 | 100% | 100% | 100% | 100% | 100% | 100% | 100% | 100% | 100% | 100% | 100% | 100% |
| 215 | SPARC OS=Homo sapiens GN=SPARC PE=1 SV=1 | SPRC | 35 | 0.0083 | 100% | 100% | 100% | 100% | 100% | 100% | 100% | 100% | 100% | 100% | 100% | 100% |
| 216 | Cluster of Sodium bicarbonate cotransporter 3 OS=Homo sapiens GN=SLC4A7 PE=1 SV=2 (S4A7_HUMAN) | S4A7 [2] | 136 | 0.13 | 100% | 100% | 100% | 100% | 100% | 100% | 100% | 100% | 100% | 100% | 95% | 100% |
| 217 | Trans-Golgi network integral membrane protein 2 OS=Homo sapiens GN=TGOLN2 PE=1 SV=2 | TGON2 | 51 | 0.05 | 100% | 100% | 100% | 100% | 100% | 100% | 100% | 100% | 100% | 100% | 100% | 100% |
| 218 | Ras-related protein Rab-23 OS=Homo sapiens GN=RAB23 PE=1 SV=1 | RAB23 | 27 | 0.0027 | 100% | 100% | 100% | 100% | 100% | 100% | 100% | 100% | 100% | 90% | 100% | 94% |
| 219 | Protein tweety homolog 3 OS=Homo sapiens GN=TTYH3 PE=1 SV=3 | TTYH3 | 58 | 0.00035 | 95% | 100% | 100% | 100% | 100% | 100% | 100% | 100% | 100% | 100% | 95% | 100% |
| 220 | Cluster of Tyrosine-protein kinase Fyn OS=Homo sapiens GN=FYN PE=1 SV=3 (FYN_HUMAN) | FYN [7] | 61 | 0.0023 | 100% | 100% | 100% | 100% | 100% | 100% | 98% | 100% | 100% | 100% | 100% | 100% |
| 221 | Solute carrier family 2, facilitated glucose transporter member 1 OS=Homo sapiens GN=SLC2A1 PE=1 SV=2 | GTR1 | 54 | 0.039 | 100% | 100% | 100% | 100% | 100% | 100% | 100% | 100% | 100% | 100% | 100% | 100% |
| 222 | Nicastrin OS=Homo sapiens GN=NCSTN PE=1 SV=2 | NICA | 78 | 0.0028 | 100% | 100% | 100% | 100% | 100% | 100% | 100% | 100% | 100% | 100% | 100% | 100% |
| 223 | Protein RER1 OS=Homo sapiens GN=RER1 PE=1 SV=1 | RER1 | 23 | 0.041 | 95% | 100% | 100% | 100% | 100% | 100% | 100% | 100% | 100% | 100% | 100% | 100% |
| 224 | Stromal interaction molecule 1 OS=Homo sapiens GN=STIM1 PE=1 SV=3 | STIM1 | 77 | 0.14 | 100% | 100% | 100% | 100% | 100% | 100% | 100% | 95% | 100% | 100% | 100% | 90% |
| 225 | V-type proton ATPase subunit H OS=Homo sapiens GN=ATP6V1H PE=1 SV=1 | VATH | 56 | 0.9 | 100% | 100% | 100% | 100% | 100% | 100% | 100% | 100% | 100% | 100% | 100% | 100% |
| 226 | Cluster of Disks large homolog 1 OS=Homo sapiens GN=DLG1 PE=1 SV=2 (DLG1_HUMAN) | DLG1 [3] | 100 | 0.6 | 100% | 100% | 100% | 100% | 98% | 100% | 95% | 100% | 100% | 100% | 100% | 100% |
| 227 | Cluster of Ras-related protein Rab-3B OS=Homo sapiens GN=RAB3B PE=1 SV=2 (RAB3B_HUMAN) | RAB3B [3] | 25 | 0.039 | 100% | 100% | 100% | 100% | 100% | 100% | 100% | 100% | 100% | 100% | 82% | 97% |
| 228 | CD151 antigen OS=Homo sapiens GN=CD151 PE=1 SV=3 | CD151 | 28 | 0.022 | 100% | 100% | 100% | 100% | 100% | 100% | 95% | 99% | 100% | 100% | 100% | 100% |
| 229 | Plasminogen activator inhibitor 1 OS=Homo sapiens GN=SERPINE1 PE=1 SV=1 | PAI1 | 45 | 0.001 | 100% | 100% | 100% | 100% | 100% | 100% | 100% | 100% | 100% | 0 | 95% | 100% |
| 230 | Connective tissue growth factor OS=Homo sapiens GN=CTGF PE=1 SV=2 | CTGF | 38 | 0.061 | 100% | 100% | 0 | 100% | 100% | 100% | 100% | 100% | 100% | 100% | 100% | 95% |
| 231 | Cluster of Plastin-3 OS=Homo sapiens GN=PLS3 PE=1 SV=4 (PLST_HUMAN) | PLST [3] | 71 | 0.0087 | 100% | 100% | 100% | 0 | 100% | 100% | 100% | 100% | 100% | 100% | 100% | 100% |
| 232 | Atrial natriuretic peptide receptor 3 OS=Homo sapiens GN=NPR3 PE=1 SV=2 | ANPRC | 60 | 0.0005 | 100% | 100% | 100% | 100% | 100% | 100% | 0 | 0 | 0 | 100% | 100% | 100% |
| 233 | Plasminogen activator inhibitor 1 RNA-binding protein OS=Homo sapiens GN=SERBP1 PE=1 SV=2 | PAIRB | 45 | 0.14 | 100% | 100% | 100% | 100% | 100% | 100% | 100% | 100% | 100% | 100% | 100% | 100% |
| 234 | Sodium/potassium-transporting ATPase subunit beta-1 OS=Homo sapiens GN=ATP1B1 PE=1 SV=1 | AT1B1 | 35 | 0.0079 | 100% | 100% | 100% | 100% | 100% | 100% | 100% | 100% | 100% | 100% | 100% | 100% |
| 235 | Semaphorin-7A OS=Homo sapiens GN=SEMA7A PE=1 SV=1 | SEM7A | 75 | 0.00011 | 100% | 100% | 100% | 0 | 95% | 95% | 0 | 0 | 9% | 0 | 95% | 15% |
| 236 | Protein XRP2 OS=Homo sapiens GN=RP2 PE=1 SV=4 | XRP2 | 40 | 0.014 | 100% | 100% | 100% | 100% | 100% | 100% | 98% | 100% | 95% | 100% | 100% | 100% |
| 237 | Reticulon-3 OS=Homo sapiens GN=RTN3 PE=1 SV=2 | RTN3 | 113 | 0.69 | 100% | 100% | 100% | 80% | 100% | 100% | 100% | 10% | 100% | 74% | 72% | 100% |
| 238 | Copine-3 OS=Homo sapiens GN=CPNE3 PE=1 SV=1 | CPNE3 | 60 | 0.00016 | 0 | 100% | 100% | 100% | 100% | 100% | 100% | 100% | 100% | 100% | 95% | 49% |
| 239 | Lysosome-associated membrane glycoprotein 2 OS=Homo sapiens GN=LAMP2 PE=1 SV=2 | LAMP2 | 45 | < 0.00010 | 100% | 100% | 100% | 100% | 95% | 100% | 100% | 100% | 100% | 0 | 62% | 100% |
| 240 | Zinc transporter ZIP14 OS=Homo sapiens GN=SLC39A14 PE=1 SV=3 | S39AE | 54 | 0.23 | 95% | 100% | 100% | 95% | 100% | 100% | 100% | 100% | 100% | 100% | 100% | 100% |
| 241 | Double-stranded RNA-binding protein Staufen homolog 1 OS=Homo sapiens GN=STAU1 PE=1 SV=2 | STAU1 | 63 | 0.53 | 100% | 100% | 100% | 100% | 100% | 100% | 100% | 97% | 100% | 100% | 100% | 100% |
| 242 | Cluster of Plexin-A1 OS=Homo sapiens GN=PLXNA1 PE=1 SV=3 (PLXA1_HUMAN) | PLXA1 [4] | 211 | 0.2 | 95% | 100% | 100% | 100% | 100% | 100% | 100% | 98% | 95% | 100% | 100% | 100% |
| 243 | ATP-dependent RNA helicase DDX50 OS=Homo sapiens GN=DDX50 PE=1 SV=1 | DDX50 | 83 | 0.52 | 100% | 100% | 100% | 100% | 100% | 100% | 100% | 100% | 100% | 100% | 100% | 100% |
| 244 | Vesicle-associated membrane protein 7 OS=Homo sapiens GN=VAMP7 PE=1 SV=3 | VAMP7 | 25 | 0.97 | 100% | 100% | 100% | 100% | 100% | 100% | 100% | 98% | 100% | 100% | 100% | 100% |
| 245 | Cluster of Phosphatidylinositol-binding clathrin assembly protein OS=Homo sapiens GN=PICALM PE=1 SV=2 (PICAL_HUMAN) | PICAL [2] | 71 | 1 | 100% | 100% | 100% | 100% | 99% | 100% | 100% | 100% | 100% | 100% | 100% | 100% |
| 246 | Cluster of Protein kinase C alpha type OS=Homo sapiens GN=PRKCA PE=1 SV=4 (KPCA_HUMAN) | KPCA [2] | 77 | 0.1 | 100% | 100% | 100% | 12% | 100% | 100% | 0 | 0 | 0 | 100% | 100% | 100% |
| 247 | Cadherin-11 OS=Homo sapiens GN=CDH11 PE=1 SV=2 | CAD11 | 88 | 0.0001 | 100% | 100% | 100% | 100% | 100% | 100% | 95% | 0 | 100% | 0 | 100% | 100% |
| 248 | Cluster of ADP-ribosylation factor 4 OS=Homo sapiens GN=ARF4 PE=1 SV=3 (ARF4_HUMAN) | ARF4 [4] | 21 | 0.16 | 100% | 100% | 100% | 0 | 100% | 0 | 100% | 100% | 81% | 100% | 100% | 100% |
| 249 | Protein kinase C and casein kinase substrate in neurons protein 3 OS=Homo sapiens GN=PACSIN3 PE=1 SV=2 | PACN3 | 48 | 0.0037 | 0 | 0 | 0 | 100% | 100% | 100% | 100% | 100% | 100% | 100% | 100% | 100% |
| 250 | Piezo-type mechanosensitive ion channel component 1 OS=Homo sapiens GN=PIEZO1 PE=1 SV=4 | PIEZ1 | 287 | 0.044 | 100% | 100% | 100% | 95% | 100% | 100% | 95% | 100% | 100% | 100% | 100% | 100% |
| 251 | Cluster of Hepatocyte growth factor receptor OS=Homo sapiens GN=MET PE=1 SV=4 (MET_HUMAN) | MET [3] | 156 | 0.17 | 93% | 100% | 91% | 99% | 100% | 100% | 100% | 0 | 0 | 47% | 0 | 0 |
| 252 | Ras-related protein Rab-13 OS=Homo sapiens GN=RAB13 PE=1 SV=1 | RAB13 | 23 | 0.036 | 100% | 100% | 100% | 100% | 100% | 100% | 100% | 100% | 100% | 100% | 100% | 100% |
| 253 | Large neutral amino acids transporter small subunit 1 OS=Homo sapiens GN=SLC7A5 PE=1 SV=2 | LAT1 | 55 | 0.18 | 100% | 100% | 100% | 100% | 100% | 100% | 100% | 100% | 100% | 100% | 100% | 100% |
| 254 | Inactive tyrosine-protein kinase 7 OS=Homo sapiens GN=PTK7 PE=1 SV=2 | PTK7 | 118 | 0.19 | 95% | 100% | 95% | 100% | 100% | 100% | 100% | 95% | 100% | 100% | 100% | 100% |
| 255 | Extracellular sulfatase Sulf-1 OS=Homo sapiens GN=SULF1 PE=1 SV=1 | SULF1 | 101 | 0.0012 | 100% | 100% | 100% | 0 | 0 | 0 | 100% | 100% | 100% | 0 | 0 | 0 |
| 256 | Sn1-specific diacylglycerol lipase beta OS=Homo sapiens GN=DAGLB PE=1 SV=2 | DGLB | 74 | 0.47 | 100% | 100% | 100% | 99% | 100% | 100% | 100% | 100% | 100% | 100% | 100% | 100% |
| 257 | Inositol 1,4,5-trisphosphate receptor type 3 OS=Homo sapiens GN=ITPR3 PE=1 SV=2 | ITPR3 | 304 | 0.0083 | 100% | 100% | 100% | 0 | 0 | 0 | 7% | 0 | 8% | 100% | 100% | 100% |
| 258 | Na(+)/H(+) exchange regulatory cofactor NHE-RF2 OS=Homo sapiens GN=SLC9A3R2 PE=1 SV=2 | NHRF2 | 37 | 0.65 | 100% | 100% | 100% | 100% | 100% | 100% | 100% | 15% | 100% | 0 | 100% | 100% |
| 259 | Probable phospholipid-transporting ATPase IG OS=Homo sapiens GN=ATP11C PE=1 SV=3 | AT11C | 129 | 0.41 | 100% | 100% | 95% | 100% | 100% | 100% | 100% | 100% | 100% | 100% | 100% | 100% |
| 260 | Solute carrier family 2, facilitated glucose transporter member 3 OS=Homo sapiens GN=SLC2A3 PE=1 SV=1 | GTR3 | 54 | 0.4 | 95% | 95% | 100% | 100% | 100% | 100% | 100% | 100% | 100% | 95% | 95% | 95% |
| 261 | Podocalyxin OS=Homo sapiens GN=PODXL PE=1 SV=2 | PODXL | 59 | 0.15 | 100% | 100% | 100% | 100% | 100% | 100% | 100% | 100% | 100% | 100% | 100% | 100% |
| 262 | Leukocyte surface antigen CD47 OS=Homo sapiens GN=CD47 PE=1 SV=1 | CD47 | 35 | 0.66 | 100% | 100% | 100% | 100% | 100% | 100% | 100% | 100% | 100% | 100% | 100% | 100% |
| 263 | Calmodulin OS=Homo sapiens GN=CALM1 PE=1 SV=2 | CALM | 17 | 0.018 | 100% | 100% | 100% | 95% | 100% | 95% | 95% | 95% | 99% | 95% | 95% | 95% |
| 264 | Cluster of Alpha-parvin OS=Homo sapiens GN=PARVA PE=1 SV=1 (PARVA_HUMAN) | PARVA [2] | 42 | 0.056 | 95% | 100% | 100% | 100% | 100% | 100% | 100% | 100% | 100% | 100% | 100% | 100% |
| 265 | Beta-1,4-galactosyltransferase 1 OS=Homo sapiens GN=B4GALT1 PE=1 SV=5 | B4GT1 | 44 | 0.85 | 100% | 100% | 100% | 100% | 100% | 100% | 100% | 100% | 100% | 100% | 100% | 96% |
| 266 | Solute carrier family 12 member 9 OS=Homo sapiens GN=SLC12A9 PE=1 SV=1 | S12A9 | 96 | 0.066 | 100% | 95% | 100% | 100% | 100% | 100% | 100% | 100% | 100% | 100% | 100% | 0 |
| 267 | Annexin A4 OS=Homo sapiens GN=ANXA4 PE=1 SV=4 | ANXA4 | 36 | 0.035 | 88% | 100% | 100% | 100% | 100% | 100% | 100% | 100% | 100% | 100% | 98% | 100% |
| 268 | V-type proton ATPase subunit C 1 OS=Homo sapiens GN=ATP6V1C1 PE=1 SV=4 | VATC1 | 44 | 0.37 | 0 | 100% | 100% | 15% | 99% | 0 | 100% | 100% | 98% | 100% | 97% | 99% |
| 269 | ADP-ribosyl cyclase/cyclic ADP-ribose hydrolase 2 OS=Homo sapiens GN=BST1 PE=1 SV=2 | BST1 | 36 | 0.022 | 100% | 100% | 100% | 95% | 100% | 100% | 100% | 100% | 100% | 100% | 100% | 100% |
| 270 | Synaptosomal-associated protein 23 OS=Homo sapiens GN=SNAP23 PE=1 SV=1 | SNP23 | 23 | 0.00025 | 0 | 100% | 100% | 100% | 100% | 100% | 0 | 95% | 0 | 100% | 100% | 100% |
| 271 | Apolipoprotein B-100 OS=Homo sapiens GN=APOB PE=1 SV=2 | APOB | 516 | 0.07 | 0 | 100% | 100% | 93% | 100% | 100% | 100% | 100% | 100% | 0 | 0 | 95% |
| 272 | Sodium-coupled neutral amino acid transporter 2 OS=Homo sapiens GN=SLC38A2 PE=1 SV=2 | S38A2 | 56 | 0.0084 | 95% | 100% | 100% | 95% | 95% | 0 | 100% | 100% | 100% | 100% | 100% | 100% |
| 273 | Alkaline phosphatase, tissue-nonspecific isozyme OS=Homo sapiens GN=ALPL PE=1 SV=4 | PPBT | 57 | 0.00032 | 100% | 100% | 100% | 0 | 19% | 0 | 95% | 100% | 100% | 0 | 48% | 31% |
| 274 | Malate dehydrogenase, mitochondrial OS=Homo sapiens GN=MDH2 PE=1 SV=3 | MDHM | 36 | 0.64 | 100% | 100% | 100% | 95% | 100% | 100% | 100% | 100% | 100% | 100% | 100% | 100% |
| 275 | Unconventional myosin-VI OS=Homo sapiens GN=MYO6 PE=1 SV=4 | MYO6 | 150 | 0.017 | 0 | 0 | 95% | 0 | 100% | 100% | 0 | 0 | 0 | 100% | 100% | 100% |
| 276 | Serum paraoxonase/arylesterase 2 OS=Homo sapiens GN=PON2 PE=1 SV=3 | PON2 | 39 | 0.16 | 100% | 100% | 99% | 100% | 100% | 100% | 30% | 56% | 95% | 97% | 100% | 100% |
| 277 | Ras-related protein Rab-5B OS=Homo sapiens GN=RAB5B PE=1 SV=1 | RAB5B | 24 | 0.058 | 100% | 100% | 100% | 100% | 100% | 100% | 100% | 100% | 100% | 100% | 100% | 100% |
| 278 | Reversion-inducing cysteine-rich protein with Kazal motifs OS=Homo sapiens GN=RECK PE=1 SV=1 | RECK | 106 | 0.66 | 100% | 100% | 100% | 100% | 100% | 100% | 100% | 95% | 100% | 100% | 100% | 100% |
| 279 | Cluster of High affinity cationic amino acid transporter 1 OS=Homo sapiens GN=SLC7A1 PE=1 SV=1 (CTR1_HUMAN) | CTR1 [2] | 68 | 0.26 | 95% | 95% | 100% | 100% | 100% | 99% | 100% | 100% | 100% | 100% | 98% | 100% |
| 280 | Protein S100-A16 OS=Homo sapiens GN=S100A16 PE=1 SV=1 | S10AG | 12 | 0.42 | 100% | 100% | 95% | 95% | 100% | 100% | 95% | 100% | 95% | 100% | 100% | 100% |
| 281 | Stomatin-like protein 2, mitochondrial OS=Homo sapiens GN=STOML2 PE=1 SV=1 | STML2 | 39 | < 0.00010 | 95% | 55% | 95% | 99% | 100% | 100% | 0 | 0 | 0 | 100% | 100% | 100% |
| 282 | Anoctamin-6 OS=Homo sapiens GN=ANO6 PE=1 SV=2 | ANO6 | 106 | 0.01 | 95% | 100% | 95% | 0 | 100% | 100% | 100% | 100% | 99% | 100% | 100% | 100% |
| 283 | Immunoglobulin superfamily member 8 OS=Homo sapiens GN=IGSF8 PE=1 SV=1 | IGSF8 | 65 | < 0.00010 | 100% | 100% | 100% | 100% | 100% | 100% | 0 | 0 | 0 | 95% | 0 | 95% |
| 284 | Ras-related protein Rab-5A OS=Homo sapiens GN=RAB5A PE=1 SV=2 | RAB5A | 24 | 0.24 | 100% | 100% | 100% | 100% | 100% | 100% | 100% | 100% | 100% | 100% | 100% | 100% |
| 285 | CD9 antigen OS=Homo sapiens GN=CD9 PE=1 SV=4 | CD9 | 25 | 0.012 | 100% | 100% | 100% | 100% | 95% | 100% | 95% | 100% | 95% | 100% | 100% | 100% |
| 286 | Proteolipid protein 2 OS=Homo sapiens GN=PLP2 PE=1 SV=1 | PLP2 | 17 | 0.61 | 100% | 100% | 100% | 100% | 100% | 100% | 95% | 100% | 100% | 100% | 95% | 100% |
| 287 | Cluster of Ras-related protein Rab-31 OS=Homo sapiens GN=RAB31 PE=1 SV=1 (RAB31_HUMAN) | RAB31 [2] | 22 | 0.022 | 100% | 100% | 100% | 100% | 100% | 100% | 100% | 100% | 100% | 0 | 0 | 0 |
| 288 | Cluster of Alpha-adducin OS=Homo sapiens GN=ADD1 PE=1 SV=2 (ADDA_HUMAN) | ADDA [2] | 81 | 0.017 | 100% | 100% | 100% | 0 | 12% | 0 | 80% | 0 | 0 | 94% | 100% | 100% |
| 289 | Magnesium transporter protein 1 OS=Homo sapiens GN=MAGT1 PE=1 SV=1 | MAGT1 | 38 | 0.015 | 95% | 100% | 100% | 0 | 0 | 95% | 95% | 95% | 100% | 100% | 100% | 100% |
| 290 | Nck-associated protein 1 OS=Homo sapiens GN=NCKAP1 PE=1 SV=1 | NCKP1 | 129 | 0.15 | 7% | 100% | 100% | 95% | 100% | 99% | 95% | 97% | 95% | 100% | 100% | 100% |
| 291 | Solute carrier family 12 member 2 OS=Homo sapiens GN=SLC12A2 PE=1 SV=1 | S12A2 | 131 | 0.29 | 0 | 0 | 100% | 9% | 100% | 100% | 0 | 100% | 0 | 0 | 100% | 100% |
| 292 | Alpha-crystallin B chain OS=Homo sapiens GN=CRYAB PE=1 SV=2 | CRYAB | 20 | 0.17 | 0 | 89% | 100% | 100% | 100% | 100% | 100% | 98% | 100% | 0 | 95% | 0 |
| 293 | Long-chain fatty acid transport protein 4 OS=Homo sapiens GN=SLC27A4 PE=1 SV=1 | S27A4 | 72 | 0.86 | 100% | 100% | 100% | 89% | 100% | 100% | 100% | 100% | 100% | 100% | 74% | 100% |
| 294 | Caveolin-2 OS=Homo sapiens GN=CAV2 PE=1 SV=2 | CAV2 | 18 | 0.63 | 92% | 100% | 95% | 100% | 95% | 100% | 95% | 100% | 95% | 95% | 95% | 95% |
| 295 | Transmembrane glycoprotein NMB OS=Homo sapiens GN=GPNMB PE=1 SV=2 | GPNMB | 64 | 0.67 | 100% | 0 | 100% | 100% | 95% | 95% | 100% | 95% | 99% | 95% | 98% | 95% |
| 296 | Protein-glutamine gamma-glutamyltransferase 2 OS=Homo sapiens GN=TGM2 PE=1 SV=2 | TGM2 | 77 | 0.018 | 100% | 100% | 100% | 0 | 22% | 100% | 95% | 0 | 95% | 95% | 100% | 100% |
| 297 | Anthrax toxin receptor 2 OS=Homo sapiens GN=ANTXR2 PE=1 SV=5 | ANTR2 | 54 | 0.00044 | 96% | 100% | 100% | 100% | 100% | 100% | 0 | 0 | 0 | 90% | 0 | 0 |
| 298 | Integrin beta-3 OS=Homo sapiens GN=ITGB3 PE=1 SV=2 | ITB3 | 87 | 0.53 | 0 | 100% | 100% | 100% | 95% | 100% | 100% | 100% | 100% | 100% | 25% | 100% |
| 299 | Leucine-rich repeat-containing protein 8A OS=Homo sapiens GN=LRRC8A PE=1 SV=1 | LRC8A | 94 | 0.0096 | 95% | 100% | 100% | 100% | 100% | 100% | 100% | 0 | 95% | 0 | 100% | 95% |
| 300 | Protein YIPF3 OS=Homo sapiens GN=YIPF3 PE=1 SV=1 | YIPF3 | 38 | 0.054 | 100% | 100% | 100% | 100% | 100% | 100% | 100% | 100% | 100% | 72% | 5% | 95% |
| 301 | Protein scribble homolog OS=Homo sapiens GN=SCRIB PE=1 SV=4 | SCRIB | 175 | 0.75 | 99% | 100% | 100% | 100% | 100% | 100% | 8% | 100% | 100% | 95% | 0 | 100% |
| 302 | Cyclin-Y-like protein 1 OS=Homo sapiens GN=CCNYL1 PE=1 SV=2 | CCYL1 | 41 | 0.44 | 100% | 100% | 100% | 95% | 100% | 100% | 100% | 100% | 100% | 0 | 86% | 95% |
| 303 | Peptidyl-glycine alpha-amidating monooxygenase OS=Homo sapiens GN=PAM PE=1 SV=2 | AMD | 108 | 0.05 | 100% | 100% | 95% | 100% | 100% | 100% | 0 | 0 | 0 | 100% | 100% | 100% |
| 304 | Transforming growth factor-beta-induced protein ig-h3 OS=Homo sapiens GN=TGFBI PE=1 SV=1 | BGH3 | 75 | 0.0056 | 94% | 6% | 8% | 0 | 0 | 0 | 100% | 100% | 100% | 0 | 0 | 0 |
| 305 | Probable lysosomal cobalamin transporter OS=Homo sapiens GN=LMBRD1 PE=1 SV=1 | LMBD1 | 61 | 0.11 | 100% | 95% | 100% | 95% | 100% | 95% | 100% | 99% | 95% | 100% | 100% | 100% |
| 306 | Monocarboxylate transporter 1 OS=Homo sapiens GN=SLC16A1 PE=1 SV=3 | MOT1 | 54 | 0.012 | 95% | 0 | 100% | 0 | 100% | 100% | 76% | 95% | 0 | 100% | 100% | 100% |
| 307 | Endothelial protein C receptor OS=Homo sapiens GN=PROCR PE=1 SV=1 | EPCR | 27 | 0.1 | 100% | 100% | 100% | 100% | 100% | 100% | 100% | 100% | 100% | 95% | 95% | 95% |
| 308 | Transmembrane emp24 domain-containing protein 1 OS=Homo sapiens GN=TMED1 PE=1 SV=1 | TMED1 | 25 | 0.14 | 100% | 100% | 100% | 95% | 100% | 95% | 100% | 95% | 95% | 100% | 100% | 57% |
| 309 | Armadillo repeat protein deleted in velo-cardio-facial syndrome OS=Homo sapiens GN=ARVCF PE=1 SV=1 | ARVC | 105 | 0.0003 | 0 | 0 | 0 | 100% | 100% | 100% | 0 | 0 | 0 | 100% | 100% | 100% |
| 310 | Dyslexia-associated protein KIAA0319-like protein OS=Homo sapiens GN=KIAA0319L PE=1 SV=2 | K319L | 116 | 0.0074 | 0 | 68% | 97% | 0 | 100% | 100% | 0 | 0 | 0 | 100% | 98% | 100% |
| 311 | Dystrophin OS=Homo sapiens GN=DMD PE=1 SV=3 | DMD | 427 | 0.00021 | 0 | 0 | 0 | 100% | 100% | 100% | 0 | 61% | 0 | 0 | 100% | 0 |
| 312 | Chloride intracellular channel protein 1 OS=Homo sapiens GN=CLIC1 PE=1 SV=4 | CLIC1 | 27 | 0.0014 | 100% | 100% | 100% | 23% | 0 | 0 | 95% | 79% | 0 | 100% | 100% | 100% |
| 313 | Cathepsin Z OS=Homo sapiens GN=CTSZ PE=1 SV=1 | CATZ | 34 | 0.2 | 95% | 95% | 100% | 95% | 87% | 100% | 95% | 95% | 100% | 100% | 100% | 100% |
| 314 | Major prion protein OS=Homo sapiens GN=PRNP PE=1 SV=1 | PRIO | 28 | 0.69 | 100% | 63% | 100% | 95% | 65% | 100% | 42% | 100% | 100% | 100% | 100% | 100% |
| 315 | Transforming growth factor beta-1 OS=Homo sapiens GN=TGFB1 PE=1 SV=2 | TGFB1 | 44 | 0.18 | 0 | 20% | 95% | 95% | 100% | 100% | 99% | 100% | 100% | 95% | 80% | 18% |
| 316 | Ras-related protein Rab-35 OS=Homo sapiens GN=RAB35 PE=1 SV=1 | RAB35 | 23 | 0.14 | 100% | 100% | 100% | 83% | 88% | 0 | 98% | 100% | 91% | 100% | 98% | 75% |
| 317 | Cluster of Glucose-6-phosphate isomerase OS=Homo sapiens GN=GPI PE=1 SV=4 (G6PI_HUMAN) | G6PI [2] | 63 | 0.23 | 92% | 100% | 100% | 0 | 0 | 0 | 0 | 100% | 100% | 0 | 56% | 0 |
| 318 | EH domain-containing protein 4 OS=Homo sapiens GN=EHD4 PE=1 SV=1 | EHD4 | 61 | 0.97 | 0 | 100% | 100% | 0 | 100% | 100% | 0 | 100% | 100% | 100% | 100% | 92% |
| 319 | Guanine nucleotide-binding protein subunit beta-4 OS=Homo sapiens GN=GNB4 PE=1 SV=3 | GBB4 | 38 | 0.19 | 100% | 100% | 100% | 74% | 44% | 100% | 100% | 100% | 100% | 100% | 100% | 90% |
| 320 | Lysosomal Pro-X carboxypeptidase OS=Homo sapiens GN=PRCP PE=1 SV=1 | PCP | 56 | 0.29 | 95% | 95% | 95% | 0 | 100% | 0 | 100% | 97% | 95% | 100% | 100% | 100% |
| 321 | Tripartite motif-containing protein 4 OS=Homo sapiens GN=TRIM4 PE=2 SV=2 | TRIM4 | 57 | 0.25 | 100% | 95% | 0 | 21% | 100% | 0 | 19% | 84% | 0 | 17% | 31% | 92% |
| 322 | Cluster of Protein lin-7 homolog C OS=Homo sapiens GN=LIN7C PE=1 SV=1 (LIN7C_HUMAN) | LIN7C [2] | 22 | 0.0037 | 0 | 100% | 0 | 95% | 100% | 100% | 100% | 100% | 100% | 16% | 95% | 56% |
| 323 | Anion exchange protein 2 OS=Homo sapiens GN=SLC4A2 PE=1 SV=4 | B3A2 | 137 | 0.0001 | 97% | 13% | 97% | 100% | 100% | 100% | 0 | 96% | 0 | 96% | 95% | 95% |
| 324 | Choline transporter-like protein 1 OS=Homo sapiens GN=SLC44A1 PE=1 SV=1 | CTL1 | 73 | 0.042 | 95% | 95% | 100% | 95% | 95% | 100% | 95% | 100% | 95% | 100% | 100% | 100% |
| 325 | Na(+)/H(+) exchange regulatory cofactor NHE-RF1 OS=Homo sapiens GN=SLC9A3R1 PE=1 SV=4 | NHRF1 | 39 | 0.8 | 98% | 100% | 100% | 0 | 100% | 100% | 95% | 95% | 95% | 100% | 95% | 95% |
| 326 | Sialin OS=Homo sapiens GN=SLC17A5 PE=1 SV=2 | S17A5 | 55 | 0.31 | 95% | 100% | 100% | 0 | 95% | 68% | 100% | 100% | 100% | 100% | 100% | 22% |
| 327 | Sphingomyelin phosphodiesterase OS=Homo sapiens GN=SMPD1 PE=1 SV=4 | ASM | 70 | 0.82 | 17% | 95% | 100% | 51% | 100% | 100% | 95% | 100% | 0 | 100% | 100% | 100% |
| 328 | Membrane cofactor protein OS=Homo sapiens GN=CD46 PE=1 SV=3 | MCP | 44 | 0.011 | 0 | 96% | 95% | 98% | 100% | 95% | 0 | 21% | 95% | 100% | 100% | 100% |
| 329 | Long-chain-fatty-acid--CoA ligase 1 OS=Homo sapiens GN=ACSL1 PE=1 SV=1 | ACSL1 | 78 | 0.25 | 95% | 100% | 100% | 0 | 100% | 100% | 100% | 92% | 56% | 83% | 0 | 95% |
| 330 | Cluster of Interferon-induced transmembrane protein 1 OS=Homo sapiens GN=IFITM1 PE=1 SV=3 (IFM1_HUMAN) | IFM1 [2] | 14 | 0.034 | 95% | 100% | 100% | 95% | 95% | 95% | 95% | 95% | 95% | 95% | 100% | 95% |
| 331 | Complement decay-accelerating factor OS=Homo sapiens GN=CD55 PE=1 SV=4 | DAF | 41 | 0.77 | 95% | 95% | 100% | 95% | 100% | 100% | 100% | 0 | 100% | 0 | 100% | 29% |
| 332 | Synaptosomal-associated protein 29 OS=Homo sapiens GN=SNAP29 PE=1 SV=1 | SNP29 | 29 | 0.1 | 95% | 100% | 100% | 100% | 100% | 100% | 95% | 95% | 69% | 95% | 95% | 100% |
| 333 | Dystroglycan OS=Homo sapiens GN=DAG1 PE=1 SV=2 | DAG1 | 97 | < 0.00010 | 0 | 0 | 0 | 100% | 100% | 100% | 0 | 20% | 0 | 95% | 100% | 100% |
| 334 | FERM, RhoGEF and pleckstrin domain-containing protein 1 OS=Homo sapiens GN=FARP1 PE=1 SV=1 | FARP1 | 119 | 0.65 | 0 | 100% | 100% | 0 | 100% | 0 | 100% | 98% | 12% | 39% | 100% | 0 |
| 335 | Vesicle-associated membrane protein 5 OS=Homo sapiens GN=VAMP5 PE=1 SV=1 | VAMP5 | 13 | 0.00018 | 100% | 98% | 100% | 83% | 0 | 83% | 0 | 0 | 0 | 100% | 100% | 100% |
| 336 | Syntaxin-6 OS=Homo sapiens GN=STX6 PE=1 SV=1 | STX6 | 29 | 0.0016 | 0 | 0 | 0 | 95% | 100% | 95% | 100% | 100% | 100% | 100% | 100% | 95% |
| 337 | Syntaxin-2 OS=Homo sapiens GN=STX2 PE=1 SV=3 | STX2 | 33 | 0.19 | 0 | 100% | 100% | 95% | 100% | 99% | 95% | 47% | 0 | 95% | 97% | 95% |
| 338 | Tight junction protein ZO-1 OS=Homo sapiens GN=TJP1 PE=1 SV=3 | ZO1 | 195 | 0.44 | 0 | 100% | 95% | 100% | 100% | 100% | 95% | 95% | 95% | 100% | 98% | 100% |
| 339 | Progressive ankylosis protein homolog OS=Homo sapiens GN=ANKH PE=1 SV=2 | ANKH | 54 | 0.00012 | 0 | 95% | 95% | 0 | 0 | 0 | 100% | 100% | 100% | 0 | 95% | 0 |
| 340 | Endoplasmic reticulum aminopeptidase 1 OS=Homo sapiens GN=ERAP1 PE=1 SV=3 | ERAP1 | 107 | 0.3 | 0 | 100% | 100% | 0 | 100% | 98% | 7% | 0 | 0 | 100% | 100% | 100% |
| 341 | Glutathione S-transferase P OS=Homo sapiens GN=GSTP1 PE=1 SV=2 | GSTP1 | 23 | 0.49 | 17% | 100% | 52% | 95% | 100% | 100% | 100% | 59% | 100% | 100% | 100% | 0 |
| 342 | Phospholipid scramblase 1 OS=Homo sapiens GN=PLSCR1 PE=1 SV=1 | PLS1 | 35 | 0.1 | 0 | 0 | 99% | 0 | 99% | 100% | 11% | 22% | 0 | 99% | 100% | 100% |
| 343 | Fragile X mental retardation protein 1 OS=Homo sapiens GN=FMR1 PE=1 SV=1 | FMR1 | 71 | 0.002 | 99% | 95% | 100% | 95% | 98% | 98% | 0 | 0 | 0 | 100% | 100% | 100% |
| 344 | CDP-diacylglycerol--inositol 3-phosphatidyltransferase OS=Homo sapiens GN=CDIPT PE=1 SV=1 | CDIPT | 24 | 0.034 | 100% | 100% | 100% | 84% | 100% | 100% | 0 | 0 | 0 | 95% | 100% | 100% |
| 345 | Dynein light chain 1, cytoplasmic OS=Homo sapiens GN=DYNLL1 PE=1 SV=1 | DYL1 | 10 | 0.16 | 95% | 100% | 100% | 0 | 95% | 7% | 95% | 100% | 0 | 100% | 95% | 0 |
| 346 | Serine incorporator 1 OS=Homo sapiens GN=SERINC1 PE=1 SV=1 | SERC1 | 50 | 0.11 | 100% | 100% | 100% | 100% | 100% | 95% | 95% | 95% | 95% | 95% | 95% | 95% |
| 347 | Excitatory amino acid transporter 3 OS=Homo sapiens GN=SLC1A1 PE=1 SV=2 | EAA3 | 57 | 0.17 | 95% | 95% | 0 | 100% | 95% | 99% | 95% | 0 | 95% | 95% | 0 | 95% |
| 348 | Cluster of Formin-like protein 2 OS=Homo sapiens GN=FMNL2 PE=1 SV=3 (FMNL2_HUMAN) | FMNL2 [2] | 123 | 0.031 | 100% | 100% | 100% | 0 | 13% | 0 | 100% | 51% | 100% | 0 | 100% | 0 |
| 349 | Occludin OS=Homo sapiens GN=OCLN PE=1 SV=1 | OCLN | 59 | 0.0022 | 0 | 0 | 0 | 100% | 100% | 100% | 0 | 0 | 0 | 0 | 95% | 0 |
| 350 | Gap junction alpha-1 protein OS=Homo sapiens GN=GJA1 PE=1 SV=2 | CXA1 | 43 | 0.82 | 0 | 100% | 100% | 0 | 100% | 100% | 0 | 100% | 100% | 99% | 95% | 0 |
| 351 | Steryl-sulfatase OS=Homo sapiens GN=STS PE=1 SV=2 | STS | 65 | 0.67 | 100% | 100% | 0 | 71% | 85% | 100% | 0 | 0 | 0 | 100% | 98% | 6% |
| 352 | Complement C4-A OS=Homo sapiens GN=C4A PE=1 SV=2 | CO4A (+1) | 193 | 0.0014 | 0 | 0 | 0 | 0 | 0 | 0 | 100% | 100% | 100% | 95% | 100% | 100% |
| 353 | Protein YIPF4 OS=Homo sapiens GN=YIPF4 PE=1 SV=1 | YIPF4 | 27 | 0.12 | 0 | 100% | 21% | 95% | 95% | 100% | 100% | 100% | 0 | 95% | 95% | 95% |
| 354 | Dystonin OS=Homo sapiens GN=DST PE=1 SV=4 | DYST | 861 | 0.22 | 0 | 57% | 0 | 0 | 100% | 49% | 15% | 99% | 69% | 54% | 0 | 5% |
| 355 | Rho guanine nucleotide exchange factor 5 OS=Homo sapiens GN=ARHGEF5 PE=1 SV=3 | ARHG5 | 177 | 0.8 | 100% | 72% | 18% | 56% | 75% | 95% | 29% | 100% | 100% | 0 | 0 | 0 |
| 356 | Ectonucleotide pyrophosphatase/phosphodiesterase family member 1 OS=Homo sapiens GN=ENPP1 PE=1 SV=2 | ENPP1 | 105 | 0.04 | 95% | 100% | 100% | 0 | 0 | 69% | 0 | 0 | 0 | 0 | 0 | 95% |
| 357 | Type-1 angiotensin II receptor-associated protein OS=Homo sapiens GN=AGTRAP PE=1 SV=1 | ATRAP | 17 | 0.28 | 95% | 100% | 95% | 95% | 95% | 95% | 100% | 95% | 95% | 95% | 97% | 95% |
| 358 | Copine-2 OS=Homo sapiens GN=CPNE2 PE=1 SV=3 | CPNE2 | 61 | 0.43 | 0 | 30% | 100% | 95% | 100% | 95% | 7% | 22% | 95% | 0 | 100% | 100% |
| 359 | Signal peptide peptidase-like 2A OS=Homo sapiens GN=SPPL2A PE=1 SV=2 | SPP2A | 58 | 0.67 | 95% | 95% | 95% | 95% | 95% | 89% | 100% | 26% | 100% | 0 | 95% | 95% |
| 360 | Electrogenic sodium bicarbonate cotransporter 1 OS=Homo sapiens GN=SLC4A4 PE=1 SV=1 | S4A4 | 121 | < 0.00010 | 0 | 0 | 0 | 100% | 100% | 100% | 0 | 0 | 0 | 100% | 95% | 100% |
| 361 | Coatomer subunit beta OS=Homo sapiens GN=COPB1 PE=1 SV=3 | COPB | 107 | 0.28 | 0 | 0 | 98% | 0 | 100% | 100% | 95% | 95% | 100% | 95% | 0 | 99% |
| 362 | DnaJ homolog subfamily C member 5 OS=Homo sapiens GN=DNAJC5 PE=1 SV=1 | DNJC5 | 22 | 0.22 | 0 | 100% | 100% | 95% | 95% | 95% | 100% | 95% | 100% | 95% | 0 | 95% |
| 363 | Sigma non-opioid intracellular receptor 1 OS=Homo sapiens GN=SIGMAR1 PE=1 SV=1 | SGMR1 | 25 | 0.89 | 20% | 100% | 100% | 95% | 10% | 95% | 100% | 95% | 95% | 95% | 0 | 100% |
| 364 | Annexin A7 OS=Homo sapiens GN=ANXA7 PE=1 SV=3 | ANXA7 | 53 | 0.99 | 95% | 95% | 0 | 100% | 63% | 100% | 95% | 41% | 95% | 100% | 99% | 0 |
| 365 | Src substrate cortactin OS=Homo sapiens GN=CTTN PE=1 SV=2 | SRC8 | 62 | 0.8 | 95% | 0 | 100% | 14% | 95% | 100% | 6% | 95% | 0 | 100% | 9% | 0 |
| 366 | Inositol 1,4,5-trisphosphate receptor type 1 OS=Homo sapiens GN=ITPR1 PE=1 SV=3 | ITPR1 | 314 | 0.00025 | 0 | 0 | 0 | 95% | 95% | 95% | 0 | 0 | 0 | 96% | 95% | 100% |
| 367 | DnaJ homolog subfamily C member 1 OS=Homo sapiens GN=DNAJC1 PE=1 SV=1 | DNJC1 | 64 | 0.41 | 0 | 100% | 100% | 95% | 0 | 0 | 0 | 0 | 95% | 16% | 100% | 100% |
| 368 | Kin of IRRE-like protein 1 OS=Homo sapiens GN=KIRREL PE=1 SV=2 | KIRR1 | 84 | 0.049 | 0 | 0 | 0 | 6% | 100% | 100% | 100% | 99% | 100% | 0 | 0 | 0 |
| 369 | 1-phosphatidylinositol 4,5-bisphosphate phosphodiesterase delta-3 OS=Homo sapiens GN=PLCD3 PE=1 SV=3 | PLCD3 | 89 | 0.02 | 100% | 100% | 100% | 0 | 99% | 100% | 0 | 0 | 0 | 60% | 95% | 0 |
| 370 | Exocyst complex component 1 OS=Homo sapiens GN=EXOC1 PE=1 SV=4 | EXOC1 | 102 | 0.81 | 0 | 100% | 95% | 45% | 73% | 100% | 0 | 9% | 95% | 95% | 95% | 0 |
| 371 | Rab3 GTPase-activating protein non-catalytic subunit OS=Homo sapiens GN=RAB3GAP2 PE=1 SV=1 | RBGPR | 156 | 0.24 | 0 | 0 | 0 | 66% | 98% | 100% | 63% | 63% | 0 | 100% | 100% | 65% |
| 372 | Cluster of Presenilin-1 OS=Homo sapiens GN=PSEN1 PE=1 SV=1 (PSN1_HUMAN) | PSN1 [2] | 53 | 0.26 | 31% | 100% | 100% | 0 | 13% | 51% | 99% | 89% | 7% | 0 | 25% | 0 |
| 373 | Atrial natriuretic peptide receptor 2 OS=Homo sapiens GN=NPR2 PE=1 SV=1 | ANPRB | 117 | 0.18 | 100% | 100% | 100% | 0 | 0 | 95% | 95% | 95% | 95% | 95% | 100% | 0 |
| 374 | Low-density lipoprotein receptor OS=Homo sapiens GN=LDLR PE=1 SV=1 | LDLR | 95 | 0.025 | 0 | 0 | 0 | 95% | 100% | 0 | 95% | 100% | 100% | 95% | 100% | 100% |
| 375 | Cluster of Sister chromatid cohesion protein PDS5 homolog A OS=Homo sapiens GN=PDS5A PE=1 SV=1 (PDS5A_HUMAN) | PDS5A [2] | 151 | 0.00021 | 0 | 0 | 0 | 99% | 100% | 100% | 100% | 99% | 100% | 0 | 0 | 0 |
| 376 | Syndecan-4 OS=Homo sapiens GN=SDC4 PE=1 SV=2 | SDC4 | 22 | 0.38 | 0 | 100% | 21% | 0 | 99% | 83% | 0 | 0 | 0 | 95% | 95% | 33% |
| 377 | Protein HEG homolog 1 OS=Homo sapiens GN=HEG1 PE=1 SV=3 | HEG1 | 147 | 0.042 | 0 | 95% | 100% | 95% | 95% | 99% | 95% | 95% | 98% | 0 | 0 | 0 |
| 378 | Glypican-6 OS=Homo sapiens GN=GPC6 PE=1 SV=1 | GPC6 | 63 | 0.00041 | 100% | 100% | 100% | 0 | 0 | 0 | 0 | 0 | 0 | 95% | 100% | 100% |
| 379 | Probable phospholipid-transporting ATPase IIA OS=Homo sapiens GN=ATP9A PE=1 SV=3 | ATP9A | 119 | 0.012 | 0 | 95% | 95% | 100% | 95% | 95% | 100% | 100% | 100% | 0 | 0 | 52% |
| 380 | RNA-binding protein EWS OS=Homo sapiens GN=EWSR1 PE=1 SV=1 | EWS | 68 | 0.17 | 5% | 95% | 95% | 95% | 95% | 95% | 95% | 100% | 95% | 95% | 95% | 95% |
| 381 | Monocarboxylate transporter 2 OS=Homo sapiens GN=SLC16A7 PE=1 SV=2 | MOT2 | 52 | 0.52 | 95% | 0 | 99% | 100% | 95% | 0 | 100% | 95% | 99% | 0 | 0 | 95% |
| 382 | Frizzled-7 OS=Homo sapiens GN=FZD7 PE=1 SV=2 | FZD7 | 64 | 0.8 | 63% | 95% | 95% | 99% | 95% | 0 | 13% | 100% | 100% | 60% | 95% | 0 |
| 383 | Receptor-type tyrosine-protein phosphatase alpha OS=Homo sapiens GN=PTPRA PE=1 SV=2 | PTPRA | 91 | 0.72 | 16% | 100% | 0 | 99% | 95% | 48% | 0 | 94% | 0 | 95% | 97% | 0 |
| 384 | Zyxin OS=Homo sapiens GN=ZYX PE=1 SV=1 | ZYX | 61 | 0.86 | 100% | 11% | 98% | 0 | 71% | 95% | 99% | 95% | 0 | 95% | 0 | 0 |
| 385 | Tumor necrosis factor receptor superfamily member 10B OS=Homo sapiens GN=TNFRSF10B PE=1 SV=2 | TR10B | 48 | 0.028 | 0 | 0 | 0 | 95% | 95% | 95% | 95% | 95% | 95% | 95% | 95% | 95% |
| 386 | Sortilin OS=Homo sapiens GN=SORT1 PE=1 SV=3 | SORT | 92 | 0.15 | 0 | 0 | 0 | 58% | 100% | 100% | 95% | 95% | 0 | 100% | 95% | 95% |
| 387 | Dipeptidyl peptidase 4 OS=Homo sapiens GN=DPP4 PE=1 SV=2 | DPP4 | 88 | 0.11 | 100% | 0 | 100% | 0 | 0 | 64% | 0 | 0 | 0 | 0 | 100% | 95% |
| 388 | Exocyst complex component 2 OS=Homo sapiens GN=EXOC2 PE=1 SV=1 | EXOC2 | 104 | 0.57 | 0 | 0 | 93% | 11% | 95% | 100% | 0 | 0 | 0 | 100% | 0 | 42% |
| 389 | Anthrax toxin receptor 1 OS=Homo sapiens GN=ANTXR1 PE=1 SV=2 | ANTR1 | 63 | 0.18 | 42% | 100% | 0 | 0 | 60% | 100% | 0 | 28% | 0 | 0 | 93% | 11% |
| 390 | Calcium-activated potassium channel subunit alpha-1 OS=Homo sapiens GN=KCNMA1 PE=1 SV=2 | KCMA1 | 138 | 0.44 | 100% | 0 | 0 | 0 | 0 | 8% | 85% | 15% | 6% | 13% | 0 | 0 |
| 391 | Vacuole membrane protein 1 OS=Homo sapiens GN=VMP1 PE=1 SV=1 | VMP1 | 46 | 0.077 | 0 | 95% | 0 | 95% | 95% | 95% | 95% | 95% | 95% | 95% | 96% | 95% |
| 392 | Bis(5'-adenosyl)-triphosphatase ENPP4 OS=Homo sapiens GN=ENPP4 PE=1 SV=3 | ENPP4 | 52 | 0.0012 | 0 | 0 | 0 | 100% | 100% | 100% | 0 | 0 | 0 | 95% | 95% | 0 |
| 393 | Epsilon-sarcoglycan OS=Homo sapiens GN=SGCE PE=1 SV=6 | SGCE | 50 | 0.04 | 0 | 22% | 95% | 100% | 95% | 100% | 0 | 0 | 0 | 0 | 0 | 100% |
| 394 | Transmembrane protein 192 OS=Homo sapiens GN=TMEM192 PE=1 SV=1 | TM192 | 31 | 0.006 | 0 | 95% | 0 | 0 | 0 | 95% | 100% | 100% | 100% | 95% | 0 | 95% |
| 395 | Lysophosphatidic acid receptor 1 OS=Homo sapiens GN=LPAR1 PE=1 SV=3 | LPAR1 | 41 | 0.0026 | 100% | 100% | 100% | 95% | 95% | 95% | 0 | 0 | 0 | 0 | 0 | 95% |
| 396 | Urea transporter 1 OS=Homo sapiens GN=SLC14A1 PE=1 SV=2 | UT1 | 43 | 0.02 | 0 | 0 | 0 | 0 | 0 | 0 | 100% | 100% | 100% | 0 | 0 | 0 |
| 397 | Cysteine-rich motor neuron 1 protein OS=Homo sapiens GN=CRIM1 PE=1 SV=1 | CRIM1 | 114 | 0.59 | 59% | 95% | 100% | 0 | 100% | 0 | 100% | 0 | 100% | 0 | 0 | 0 |
| 398 | Palmitoyltransferase ZDHHC17 OS=Homo sapiens GN=ZDHHC17 PE=1 SV=2 | ZDH17 | 73 | 0.048 | 0 | 0 | 0 | 100% | 100% | 95% | 0 | 0 | 0 | 0 | 100% | 100% |
| 399 | Syndecan-2 OS=Homo sapiens GN=SDC2 PE=1 SV=2 | SDC2 | 22 | < 0.00010 | 95% | 0 | 0 | 0 | 8% | 11% | 100% | 100% | 95% | 0 | 0 | 0 |
| 400 | ATP-binding cassette sub-family B member 6, mitochondrial OS=Homo sapiens GN=ABCB6 PE=1 SV=1 | ABCB6 | 94 | < 0.00010 | 0 | 0 | 0 | 0 | 0 | 0 | 100% | 100% | 100% | 0 | 24% | 95% |
| 401 | Neurofascin OS=Homo sapiens GN=NFASC PE=1 SV=4 | NFASC | 150 | 0.12 | 100% | 95% | 95% | 0 | 100% | 95% | 0 | 0 | 0 | 9% | 95% | 95% |
| 402 | ATP-binding cassette sub-family A member 3 OS=Homo sapiens GN=ABCA3 PE=1 SV=2 | ABCA3 | 191 | 0.35 | 0 | 0 | 0 | 95% | 0 | 95% | 16% | 100% | 0 | 100% | 78% | 22% |
| 403 | Calpain small subunit 1 OS=Homo sapiens GN=CAPNS1 PE=1 SV=1 | CPNS1 | 28 | 0.27 | 0 | 95% | 95% | 95% | 95% | 95% | 95% | 95% | 95% | 0 | 95% | 95% |
| 404 | Cyclin-Y OS=Homo sapiens GN=CCNY PE=1 SV=2 | CCNY | 39 | 0.084 | 100% | 100% | 95% | 95% | 99% | 95% | 99% | 0 | 0 | 0 | 0 | 95% |
| 405 | Receptor-type tyrosine-protein phosphatase kappa OS=Homo sapiens GN=PTPRK PE=1 SV=2 | PTPRK | 162 | 0.0024 | 0 | 0 | 0 | 99% | 100% | 95% | 0 | 0 | 0 | 95% | 97% | 100% |
| 406 | Actin-related protein 2/3 complex subunit 2 OS=Homo sapiens GN=ARPC2 PE=1 SV=1 | ARPC2 | 34 | 0.048 | 0 | 0 | 0 | 95% | 100% | 90% | 95% | 95% | 95% | 0 | 5% | 27% |
| 407 | Y+L amino acid transporter 2 OS=Homo sapiens GN=SLC7A6 PE=1 SV=3 | YLAT2 | 57 | 0.047 | 0 | 0 | 65% | 95% | 100% | 100% | 100% | 0 | 100% | 95% | 0 | 0 |
| 408 | Leucine-rich repeat-containing protein 8D OS=Homo sapiens GN=LRRC8D PE=1 SV=1 | LRC8D | 98 | 0.44 | 0 | 65% | 0 | 0 | 0 | 0 | 100% | 95% | 0 | 0 | 0 | 0 |
| 409 | Band 4.1-like protein 3 OS=Homo sapiens GN=EPB41L3 PE=1 SV=2 | E41L3 | 121 | 0.43 | 0 | 0 | 67% | 73% | 39% | 97% | 0 | 0 | 0 | 78% | 96% | 100% |
| 410 | CD99 antigen OS=Homo sapiens GN=CD99 PE=1 SV=1 | CD99 | 19 | 0.1 | 95% | 0 | 95% | 95% | 95% | 95% | 63% | 0 | 95% | 0 | 0 | 0 |
| 411 | Protein Niban OS=Homo sapiens GN=FAM129A PE=1 SV=1 | NIBAN | 103 | 0.024 | 95% | 100% | 95% | 0 | 0 | 0 | 0 | 0 | 0 | 95% | 97% | 100% |
| 412 | cAMP-dependent protein kinase type I-alpha regulatory subunit OS=Homo sapiens GN=PRKAR1A PE=1 SV=1 | KAP0 | 43 | 0.54 | 0 | 100% | 0 | 99% | 15% | 0 | 0 | 0 | 0 | 95% | 100% | 0 |
| 413 | Membrane magnesium transporter 1 OS=Homo sapiens GN=MMGT1 PE=1 SV=1 | MMGT1 | 15 | 0.48 | 0 | 95% | 0 | 95% | 0 | 100% | 95% | 0 | 0 | 95% | 0 | 95% |
| 414 | MAGUK p55 subfamily member 6 OS=Homo sapiens GN=MPP6 PE=1 SV=2 | MPP6 | 61 | 0.44 | 63% | 100% | 100% | 0 | 0 | 0 | 0 | 0 | 0 | 95% | 62% | 65% |
| 415 | Probable palmitoyltransferase ZDHHC20 OS=Homo sapiens GN=ZDHHC20 PE=1 SV=1 | ZDH20 | 42 | 0.33 | 0 | 0 | 0 | 0 | 99% | 99% | 0 | 83% | 95% | 0 | 95% | 95% |
| 416 | Protein EFR3 homolog A OS=Homo sapiens GN=EFR3A PE=1 SV=2 | EFR3A | 93 | 0.6 | 0 | 0 | 0 | 0 | 0 | 99% | 0 | 0 | 0 | 29% | 97% | 95% |
| 417 | Disintegrin and metalloproteinase domain-containing protein 17 OS=Homo sapiens GN=ADAM17 PE=1 SV=1 | ADA17 | 93 | 0.076 | 0 | 95% | 95% | 95% | 0 | 95% | 0 | 0 | 0 | 95% | 77% | 95% |
| 418 | 3-keto-steroid reductase OS=Homo sapiens GN=HSD17B7 PE=1 SV=1 | DHB7 | 38 | 0.29 | 0 | 0 | 95% | 95% | 95% | 33% | 5% | 95% | 95% | 0 | 0 | 0 |
| 419 | Nuclear factor of activated T-cells, cytoplasmic 2 OS=Homo sapiens GN=NFATC2 PE=1 SV=2 | NFAC2 | 100 | 0.59 | 17% | 0 | 0 | 92% | 0 | 12% | 0 | 42% | 24% | 0 | 12% | 82% |
| 420 | Programmed cell death 1 ligand 2 OS=Homo sapiens GN=PDCD1LG2 PE=1 SV=2 | PD1L2 | 31 | 0.00099 | 0 | 0 | 0 | 99% | 100% | 100% | 0 | 0 | 0 | 0 | 0 | 0 |
| 421 | Receptor-type tyrosine-protein phosphatase eta OS=Homo sapiens GN=PTPRJ PE=1 SV=3 | PTPRJ | 146 | 0.0035 | 0 | 0 | 0 | 100% | 99% | 100% | 86% | 0 | 62% | 95% | 0 | 95% |
| 422 | Retinoic acid-induced protein 3 OS=Homo sapiens GN=GPRC5A PE=1 SV=2 | RAI3 | 40 | 0.00036 | 0 | 0 | 0 | 100% | 100% | 100% | 0 | 0 | 0 | 0 | 0 | 0 |
| 423 | E3 ubiquitin-protein ligase CHIP OS=Homo sapiens GN=STUB1 PE=1 SV=2 | CHIP | 35 | 0.18 | 0 | 97% | 100% | 100% | 95% | 0 | 0 | 0 | 0 | 0 | 0 | 0 |
| 424 | Protein S100-A8 OS=Homo sapiens GN=S100A8 PE=1 SV=1 | S10A8 | 11 | 0.052 | 0 | 100% | 100% | 0 | 0 | 0 | 0 | 0 | 0 | 0 | 0 | 0 |
| 425 | Vasodilator-stimulated phosphoprotein OS=Homo sapiens GN=VASP PE=1 SV=3 | VASP | 40 | 0.71 | 0 | 100% | 0 | 0 | 0 | 0 | 100% | 23% | 0 | 89% | 0 | 95% |
| 426 | V-type proton ATPase subunit G 1 OS=Homo sapiens GN=ATP6V1G1 PE=1 SV=3 | VATG1 | 14 | 0.4 | 95% | 45% | 10% | 95% | 0 | 47% | 95% | 0 | 100% | 0 | 0 | 0 |
| 427 | Ras-related protein Rab-9A OS=Homo sapiens GN=RAB9A PE=1 SV=1 | RAB9A | 23 | 0.71 | 0 | 0 | 100% | 0 | 42% | 94% | 0 | 100% | 18% | 0 | 0 | 0 |
| 428 | Amyloid-like protein 2 OS=Homo sapiens GN=APLP2 PE=1 SV=2 | APLP2 | 87 | 0.12 | 0 | 95% | 100% | 0 | 0 | 0 | 100% | 0 | 100% | 29% | 0 | 0 |
| 429 | Serine/threonine-protein kinase MRCK beta OS=Homo sapiens GN=CDC42BPB PE=1 SV=2 | MRCKB | 194 | 0.26 | 0 | 95% | 100% | 0 | 0 | 9% | 0 | 0 | 0 | 38% | 0 | 99% |
| 430 | Abl interactor 1 OS=Homo sapiens GN=ABI1 PE=1 SV=4 | ABI1 | 55 | 0.37 | 0 | 0 | 6% | 0 | 95% | 95% | 0 | 0 | 99% | 95% | 95% | 0 |
| 431 | Protein NDRG1 OS=Homo sapiens GN=NDRG1 PE=1 SV=1 | NDRG1 | 43 | 0.0068 | 95% | 0 | 95% | 0 | 0 | 0 | 95% | 95% | 95% | 95% | 95% | 95% |
| 432 | Taste receptor type 1 member 1 OS=Homo sapiens GN=TAS1R1 PE=2 SV=1 | TS1R1 | 93 | 0.59 | 0 | 0 | 88% | 8% | 12% | 0 | 0 | 0 | 95% | 0 | 0 | 0 |
| 433 | Cluster of Receptor tyrosine-protein kinase erbB-2 OS=Homo sapiens GN=ERBB2 PE=1 SV=1 (ERBB2_HUMAN) | ERBB2 [2] | 138 | 0.53 | 0 | 0 | 97% | 0 | 100% | 100% | 0 | 100% | 95% | 90% | 0 | 0 |
| 434 | Integrin alpha-2 OS=Homo sapiens GN=ITGA2 PE=1 SV=1 | ITA2 | 129 | 0.12 | 100% | 100% | 0 | 0 | 0 | 0 | 0 | 0 | 0 | 0 | 0 | 0 |
| 435 | Carbonic anhydrase 12 OS=Homo sapiens GN=CA12 PE=1 SV=1 | CAH12 | 39 | 0.39 | 100% | 98% | 31% | 0 | 0 | 0 | 0 | 0 | 95% | 0 | 95% | 0 |
| 436 | Monocyte differentiation antigen CD14 OS=Homo sapiens GN=CD14 PE=1 SV=2 | CD14 | 40 | 0.027 | 0 | 0 | 0 | 95% | 95% | 100% | 0 | 0 | 0 | 0 | 95% | 95% |
| 437 | Interleukin-6 receptor subunit beta OS=Homo sapiens GN=IL6ST PE=1 SV=2 | IL6RB | 104 | 0.45 | 0 | 95% | 95% | 0 | 0 | 15% | 0 | 18% | 0 | 21% | 0 | 100% |
| 438 | Chloride intracellular channel protein 4 OS=Homo sapiens GN=CLIC4 PE=1 SV=4 | CLIC4 | 29 | 0.25 | 98% | 100% | 0 | 0 | 0 | 0 | 11% | 97% | 14% | 0 | 0 | 0 |
| 439 | Dedicator of cytokinesis protein 5 OS=Homo sapiens GN=DOCK5 PE=1 SV=3 | DOCK5 | 215 | 0.59 | 0 | 95% | 100% | 0 | 100% | 0 | 0 | 0 | 89% | 0 | 0 | 0 |
| 440 | Integrin alpha-4 OS=Homo sapiens GN=ITGA4 PE=1 SV=3 | ITA4 | 115 | 0.59 | 0 | 0 | 95% | 0 | 0 | 0 | 0 | 0 | 0 | 64% | 0 | 100% |
| 441 | Tight junction protein ZO-2 OS=Homo sapiens GN=TJP2 PE=1 SV=2 | ZO2 | 134 | 0.44 | 0 | 0 | 0 | 0 | 0 | 18% | 0 | 0 | 0 | 100% | 0 | 62% |
| 442 | Palmitoyltransferase ZDHHC5 OS=Homo sapiens GN=ZDHHC5 PE=1 SV=2 | ZDHC5 | 78 | 0.18 | 0 | 95% | 0 | 0 | 37% | 72% | 100% | 94% | 0 | 0 | 0 | 0 |
| 443 | Inositol 1,4,5-trisphosphate receptor-interacting protein OS=Homo sapiens GN=ITPRIP PE=1 SV=1 | IPRI | 62 | 0.37 | 0 | 39% | 95% | 99% | 95% | 0 | 0 | 0 | 0 | 0 | 0 | 95% |
| 444 | MAGUK p55 subfamily member 5 OS=Homo sapiens GN=MPP5 PE=1 SV=3 | MPP5 | 77 | 0.004 | 0 | 0 | 0 | 95% | 95% | 95% | 0 | 0 | 0 | 0 | 95% | 0 |
| 445 | Sodium-dependent phosphate transporter 2 OS=Homo sapiens GN=SLC20A2 PE=1 SV=1 | S20A2 | 70 | 0.0032 | 95% | 0 | 95% | 95% | 95% | 95% | 0 | 0 | 0 | 0 | 0 | 0 |
| 446 | Ephrin-B1 OS=Homo sapiens GN=EFNB1 PE=1 SV=1 | EFNB1 | 38 | 0.37 | 90% | 0 | 0 | 0 | 95% | 95% | 0 | 0 | 0 | 95% | 95% | 0 |
| 447 | Olfactory receptor 5K2 OS=Homo sapiens GN=OR5K2 PE=2 SV=3 | OR5K2 | 36 | 0.44 | 0 | 0 | 0 | 0 | 0 | 50% | 12% | 0 | 95% | 0 | 0 | 0 |
| 448 | Cadherin-6 OS=Homo sapiens GN=CDH6 PE=1 SV=1 | CADH6 | 88 | < 0.00010 | 95% | 100% | 100% | 0 | 0 | 0 | 0 | 0 | 14% | 0 | 0 | 0 |
| 449 | Transient receptor potential cation channel subfamily M member 4 OS=Homo sapiens GN=TRPM4 PE=1 SV=1 | TRPM4 | 134 | 0.00057 | 0 | 0 | 0 | 100% | 95% | 100% | 0 | 0 | 0 | 0 | 0 | 20% |
| 450 | Neuronal growth regulator 1 OS=Homo sapiens GN=NEGR1 PE=1 SV=3 | NEGR1 | 39 | 0.021 | 95% | 100% | 95% | 0 | 0 | 95% | 0 | 0 | 0 | 0 | 0 | 0 |
| 451 | Tumor suppressor candidate 3 OS=Homo sapiens GN=TUSC3 PE=1 SV=1 | TUSC3 | 40 | 0.59 | 0 | 100% | 0 | 0 | 23% | 0 | 0 | 0 | 0 | 0 | 82% | 0 |
| 452 | LanC-like protein 1 OS=Homo sapiens GN=LANCL1 PE=1 SV=1 | LANC1 | 45 | 0.12 | 0 | 95% | 95% | 0 | 0 | 0 | 95% | 0 | 99% | 0 | 0 | 0 |
| 453 | Pannexin-1 OS=Homo sapiens GN=PANX1 PE=1 SV=4 | PANX1 | 48 | 0.8 | 0 | 0 | 0 | 7% | 0 | 95% | 0 | 0 | 99% | 0 | 98% | 0 |
| 454 | Metal transporter CNNM3 OS=Homo sapiens GN=CNNM3 PE=1 SV=1 | CNNM3 | 76 | 0.24 | 0 | 0 | 0 | 0 | 97% | 0 | 0 | 0 | 0 | 0 | 95% | 95% |
| 455 | Casein kinase II subunit alpha OS=Homo sapiens GN=CSNK2A1 PE=1 SV=1 | CSK21 (+1) | 45 | 0.013 | 0 | 0 | 0 | 95% | 95% | 0 | 0 | 0 | 8% | 95% | 95% | 95% |
| 456 | Agrin OS=Homo sapiens GN=AGRN PE=1 SV=5 | AGRIN | 217 | 0.44 | 0 | 0 | 0 | 0 | 69% | 23% | 0 | 0 | 0 | 95% | 66% | 0 |
| 457 | Breast cancer type 1 susceptibility protein OS=Homo sapiens GN=BRCA1 PE=1 SV=2 | BRCA1 | 208 | 0.44 | 0 | 0 | 0 | 18% | 0 | 0 | 0 | 12% | 21% | 0 | 0 | 95% |
| 458 | TGF-beta receptor type-1 OS=Homo sapiens GN=TGFBR1 PE=1 SV=1 | TGFR1 | 56 | 0.41 | 0 | 95% | 95% | 25% | 95% | 0 | 0 | 0 | 0 | 95% | 95% | 0 |
| 459 | Tyrosine-protein phosphatase non-receptor type substrate 1 OS=Homo sapiens GN=SIRPA PE=1 SV=2 | SHPS1 | 55 | 0.00029 | 95% | 100% | 100% | 0 | 0 | 0 | 0 | 0 | 0 | 0 | 0 | 0 |
| 460 | Sodium-independent sulfate anion transporter OS=Homo sapiens GN=SLC26A11 PE=2 SV=2 | S2611 | 65 | 0.17 | 0 | 0 | 95% | 0 | 0 | 0 | 11% | 82% | 100% | 0 | 0 | 0 |
| 461 | Insulin-like growth factor 1 receptor OS=Homo sapiens GN=IGF1R PE=1 SV=1 | IGF1R | 155 | 0.17 | 0 | 0 | 95% | 0 | 73% | 100% | 0 | 0 | 0 | 0 | 0 | 0 |
| 462 | Adenylate cyclase type 9 OS=Homo sapiens GN=ADCY9 PE=1 SV=4 | ADCY9 | 151 | 0.44 | 0 | 0 | 0 | 0 | 0 | 100% | 0 | 0 | 0 | 0 | 0 | 0 |
| 463 | Neogenin OS=Homo sapiens GN=NEO1 PE=1 SV=2 | NEO1 | 160 | 0.24 | 0 | 0 | 0 | 13% | 0 | 100% | 0 | 0 | 0 | 0 | 95% | 95% |
| 464 | Sorting nexin-9 OS=Homo sapiens GN=SNX9 PE=1 SV=1 | SNX9 | 67 | 0.44 | 19% | 99% | 0 | 0 | 0 | 0 | 0 | 0 | 0 | 0 | 0 | 0 |
| 465 | Integrin alpha-8 OS=Homo sapiens GN=ITGA8 PE=1 SV=3 | ITA8 | 117 | 0.052 | 0 | 0 | 0 | 0 | 0 | 0 | 95% | 98% | 90% | 0 | 0 | 0 |
| 466 | N-acetylated-alpha-linked acidic dipeptidase 2 OS=Homo sapiens GN=NAALAD2 PE=1 SV=1 | NALD2 | 84 | 0.29 | 0 | 0 | 0 | 95% | 0 | 0 | 0 | 0 | 12% | 0 | 98% | 95% |
| 467 | Plakophilin-4 OS=Homo sapiens GN=PKP4 PE=1 SV=2 | PKP4 | 132 | 0.6 | 0 | 0 | 0 | 33% | 0 | 97% | 0 | 0 | 0 | 0 | 95% | 0 |
| 468 | Probable phospholipid-transporting ATPase IC OS=Homo sapiens GN=ATP8B1 PE=1 SV=3 | AT8B1 | 144 | < 0.00010 | 13% | 0 | 0 | 0 | 0 | 0 | 95% | 80% | 71% | 0 | 0 | 0 |
| 469 | Drebrin-like protein OS=Homo sapiens GN=DBNL PE=1 SV=1 | DBNL | 48 | 0.052 | 0 | 23% | 0 | 0 | 0 | 8% | 0 | 0 | 0 | 95% | 95% | 20% |
| 470 | Paralemmin-2 OS=Homo sapiens GN=PALM2 PE=2 SV=3 | PALM2 | 42 | 0.55 | 0 | 0 | 0 | 0 | 95% | 10% | 0 | 0 | 0 | 95% | 0 | 0 |
| 471 | Phospholipid scramblase 4 OS=Homo sapiens GN=PLSCR4 PE=1 SV=2 | PLS4 | 37 | 0.41 | 0 | 95% | 95% | 0 | 95% | 0 | 0 | 0 | 0 | 95% | 95% | 0 |
| 472 | Synaptotagmin-11 OS=Homo sapiens GN=SYT11 PE=1 SV=2 | SYT11 | 48 | 0.17 | 0 | 0 | 95% | 46% | 95% | 95% | 0 | 0 | 0 | 0 | 0 | 0 |
| 473 | Clathrin light chain A OS=Homo sapiens GN=CLTA PE=1 SV=1 | CLCA | 27 | 0.44 | 0 | 48% | 0 | 14% | 0 | 0 | 0 | 0 | 0 | 92% | 0 | 0 |
| 474 | Basal cell adhesion molecule OS=Homo sapiens GN=BCAM PE=1 SV=2 | BCAM | 67 | 0.57 | 0 | 100% | 0 | 0 | 0 | 0 | 0 | 0 | 0 | 95% | 0 | 0 |
| 475 | Protein jagged-1 OS=Homo sapiens GN=JAG1 PE=1 SV=3 | JAG1 | 134 | 0.094 | 0 | 0 | 0 | 0 | 0 | 0 | 0 | 100% | 99% | 0 | 0 | 0 |
| 476 | Tumor necrosis factor receptor superfamily member 11B OS=Homo sapiens GN=TNFRSF11B PE=1 SV=3 | TR11B | 46 | 0.0014 | 0 | 0 | 0 | 0 | 0 | 0 | 95% | 95% | 100% | 0 | 0 | 0 |
| 477 | Consortin OS=Homo sapiens GN=CNST PE=1 SV=3 | CNST | 80 | < 0.00010 | 0 | 0 | 0 | 0 | 0 | 0 | 95% | 100% | 95% | 0 | 0 | 0 |
| 478 | Sodium channel protein type 4 subunit alpha OS=Homo sapiens GN=SCN4A PE=1 SV=4 | SCN4A | 208 | 0.44 | 0 | 0 | 0 | 0 | 0 | 100% | 0 | 0 | 0 | 0 | 0 | 0 |
| 479 | LDLR chaperone MESD OS=Homo sapiens GN=MESDC2 PE=1 SV=2 | MESD | 26 | 0.44 | 0 | 0 | 0 | 0 | 0 | 0 | 0 | 0 | 0 | 99% | 13% | 6% |
| 480 | DnaJ homolog subfamily B member 4 OS=Homo sapiens GN=DNAJB4 PE=1 SV=1 | DNJB4 | 38 | 0.44 | 0 | 0 | 0 | 0 | 0 | 0 | 96% | 95% | 0 | 0 | 0 | 0 |
| 481 | Collagen alpha-1(XIII) chain OS=Homo sapiens GN=COL13A1 PE=1 SV=1 | CODA1 | 70 | < 0.00010 | 0 | 0 | 0 | 95% | 95% | 95% | 0 | 0 | 0 | 0 | 0 | 0 |
| 482 | Exocyst complex component 8 OS=Homo sapiens GN=EXOC8 PE=1 SV=2 | EXOC8 | 82 | 0.19 | 0 | 0 | 0 | 95% | 95% | 0 | 40% | 0 | 0 | 0 | 0 | 95% |
| 483 | Junctional adhesion molecule B OS=Homo sapiens GN=JAM2 PE=1 SV=1 | JAM2 | 33 | 0.44 | 0 | 0 | 0 | 95% | 17% | 95% | 0 | 0 | 0 | 0 | 0 | 0 |
| 484 | Tetratricopeptide repeat protein 17 OS=Homo sapiens GN=TTC17 PE=1 SV=1 | TTC17 | 130 | 0.055 | 0 | 0 | 0 | 95% | 0 | 95% | 0 | 0 | 0 | 0 | 0 | 0 |
| 485 | Disabled homolog 2 OS=Homo sapiens GN=DAB2 PE=1 SV=3 | DAB2 | 82 | 0.056 | 95% | 11% | 95% | 0 | 0 | 0 | 0 | 0 | 0 | 0 | 0 | 0 |
| 486 | Glycerophosphodiester phosphodiesterase 1 OS=Homo sapiens GN=GDE1 PE=1 SV=1 | GDE1 | 38 | 0.59 | 0 | 95% | 8% | 0 | 95% | 0 | 0 | 0 | 0 | 0 | 0 | 57% |
| 487 | Probable G-protein coupled receptor 176 OS=Homo sapiens GN=GPR176 PE=2 SV=1 | GP176 | 57 | 0.8 | 0 | 0 | 95% | 0 | 0 | 0 | 95% | 0 | 0 | 25% | 95% | 0 |
| 488 | Intermediate conductance calcium-activated potassium channel protein 4 OS=Homo sapiens GN=KCNN4 PE=1 SV=1 | KCNN4 | 48 | 0.052 | 0 | 0 | 0 | 0 | 0 | 0 | 95% | 0 | 95% | 0 | 0 | 5% |
| 489 | LIM and senescent cell antigen-like-containing domain protein 2 OS=Homo sapiens GN=LIMS2 PE=1 SV=1 | LIMS2 | 39 | 0.6 | 0 | 13% | 0 | 0 | 0 | 95% | 0 | 0 | 0 | 0 | 0 | 95% |
| 490 | Monocarboxylate transporter 8 OS=Homo sapiens GN=SLC16A2 PE=1 SV=2 | MOT8 | 60 | 0.28 | 9% | 95% | 95% | 0 | 0 | 95% | 0 | 0 | 0 | 0 | 0 | 0 |
| 491 | Solute carrier family 41 member 3 OS=Homo sapiens GN=SLC41A3 PE=2 SV=2 | S41A3 | 55 | 0.0097 | 0 | 0 | 0 | 0 | 0 | 0 | 95% | 0 | 0 | 95% | 95% | 95% |
| 492 | Succinate dehydrogenase [ubiquinone] iron-sulfur subunit, mitochondrial OS=Homo sapiens GN=SDHB PE=1 SV=3 | SDHB | 32 | 0.44 | 0 | 0 | 0 | 0 | 19% | 0 | 0 | 0 | 0 | 23% | 95% | 0 |
| 493 | Very low-density lipoprotein receptor OS=Homo sapiens GN=VLDLR PE=1 SV=1 | VLDLR | 96 | 0.55 | 0 | 0 | 0 | 0 | 100% | 0 | 0 | 0 | 0 | 0 | 95% | 0 |
| 494 | Tyrosine-protein phosphatase non-receptor type 13 OS=Homo sapiens GN=PTPN13 PE=1 SV=2 | PTN13 | 277 | 0.44 | 0 | 0 | 0 | 0 | 0 | 0 | 0 | 0 | 0 | 0 | 0 | 100% |
| 495 | Mitochondrial Rho GTPase 2 OS=Homo sapiens GN=RHOT2 PE=1 SV=2 | MIRO2 | 68 | 0.24 | 0 | 0 | 0 | 0 | 0 | 95% | 0 | 0 | 0 | 8% | 100% | 95% |
| 496 | DnaJ homolog subfamily A member 3, mitochondrial OS=Homo sapiens GN=DNAJA3 PE=1 SV=2 | DNJA3 | 52 | 0.44 | 0 | 0 | 0 | 0 | 0 | 0 | 0 | 0 | 0 | 0 | 99% | 0 |
| 497 | Lysine--tRNA ligase OS=Homo sapiens GN=KARS PE=1 SV=3 | SYK | 68 | 0.44 | 0 | 0 | 0 | 0 | 99% | 0 | 0 | 0 | 0 | 0 | 33% | 0 |
| 498 | Excitatory amino acid transporter 1 OS=Homo sapiens GN=SLC1A3 PE=1 SV=1 | EAA1 | 60 | 0.59 | 0 | 0 | 0 | 0 | 39% | 0 | 0 | 0 | 0 | 98% | 0 | 0 |
| 499 | ADP-ribosylation factor 6 OS=Homo sapiens GN=ARF6 PE=1 SV=2 | ARF6 | 20 | 0.44 | 0 | 0 | 98% | 0 | 0 | 0 | 0 | 0 | 39% | 63% | 0 | 0 |
| 500 | Bardet-Biedl syndrome 5 protein OS=Homo sapiens GN=BBS5 PE=1 SV=1 | BBS5 | 39 | 0.44 | 0 | 0 | 0 | 95% | 12% | 0 | 6% | 0 | 0 | 0 | 0 | 0 |
| 501 | Attractin OS=Homo sapiens GN=ATRN PE=1 SV=2 | ATRN | 159 | 0.052 | 0 | 0 | 0 | 0 | 95% | 95% | 0 | 0 | 0 | 0 | 0 | 0 |
| 502 | 3-hydroxyacyl-CoA dehydrogenase type-2 OS=Homo sapiens GN=HSD17B10 PE=1 SV=3 | HCD2 | 27 | 0.44 | 0 | 0 | 0 | 0 | 0 | 0 | 0 | 0 | 0 | 95% | 54% | 65% |
| 503 | LIM and senescent cell antigen-like-containing domain protein 1 OS=Homo sapiens GN=LIMS1 PE=1 SV=4 | LIMS1 | 37 | 0.44 | 0 | 0 | 81% | 0 | 0 | 95% | 0 | 0 | 0 | 0 | 0 | 0 |
| 504 | Microsomal glutathione S-transferase 2 OS=Homo sapiens GN=MGST2 PE=1 SV=1 | MGST2 | 17 | 0.093 | 0 | 0 | 0 | 0 | 0 | 0 | 0 | 0 | 0 | 0 | 95% | 95% |
| 505 | Sialidase-1 OS=Homo sapiens GN=NEU1 PE=1 SV=1 | NEUR1 | 45 | < 0.00010 | 0 | 0 | 0 | 0 | 0 | 0 | 0 | 0 | 0 | 95% | 75% | 95% |
| 506 | Tissue factor OS=Homo sapiens GN=F3 PE=1 SV=1 | TF | 33 | 0.59 | 0 | 0 | 95% | 0 | 0 | 0 | 0 | 0 | 0 | 0 | 0 | 95% |
| 507 | Phosphatidylinositol 4-phosphate 3-kinase C2 domain-containing subunit beta OS=Homo sapiens GN=PIK3C2B PE=1 SV=2 | P3C2B | 185 | 0.44 | 0 | 0 | 0 | 0 | 0 | 0 | 0 | 94% | 0 | 0 | 0 | 0 |
| 508 | Glypican-4 OS=Homo sapiens GN=GPC4 PE=1 SV=4 | GPC4 | 62 | 0.44 | 0 | 0 | 0 | 30% | 0 | 92% | 0 | 0 | 0 | 0 | 0 | 0 |
| 509 | Protocadherin-10 OS=Homo sapiens GN=PCDH10 PE=2 SV=2 | PCD10 | 113 | 0.44 | 0 | 0 | 0 | 0 | 0 | 100% | 0 | 0 | 0 | 0 | 0 | 0 |
| 510 | Tumor necrosis factor receptor superfamily member 6 OS=Homo sapiens GN=FAS PE=1 SV=1 | TNR6 | 38 | 0.44 | 0 | 0 | 0 | 0 | 100% | 0 | 0 | 0 | 0 | 0 | 0 | 0 |
| 511 | Solute carrier family 22 member 12 OS=Homo sapiens GN=SLC22A12 PE=1 SV=1 | S22AC | 60 | 0.44 | 0 | 0 | 0 | 0 | 100% | 0 | 0 | 0 | 0 | 0 | 0 | 0 |
| 512 | Repulsive guidance molecule A OS=Homo sapiens GN=RGMA PE=1 SV=3 | RGMA | 49 | 0.44 | 0 | 0 | 99% | 0 | 0 | 0 | 0 | 0 | 0 | 0 | 0 | 0 |
| 513 | Protein numb homolog OS=Homo sapiens GN=NUMB PE=1 SV=2 | NUMB | 71 | 0.59 | 0 | 0 | 99% | 0 | 0 | 0 | 0 | 86% | 0 | 0 | 0 | 0 |
| 514 | Oxysterol-binding protein-related protein 3 OS=Homo sapiens GN=OSBPL3 PE=1 SV=1 | OSBL3 | 101 | 0.44 | 0 | 0 | 0 | 0 | 0 | 98% | 0 | 0 | 0 | 0 | 0 | 0 |
| 515 | Actin-like protein 6A OS=Homo sapiens GN=ACTL6A PE=1 SV=1 | ACL6A | 47 | 0.44 | 0 | 0 | 0 | 95% | 0 | 0 | 0 | 0 | 0 | 0 | 7% | 0 |
| 516 | Oxidized low-density lipoprotein receptor 1 OS=Homo sapiens GN=OLR1 PE=1 SV=1 | OLR1 | 31 | 0.055 | 0 | 0 | 0 | 95% | 95% | 0 | 0 | 0 | 0 | 0 | 0 | 0 |
| 517 | Bone marrow stromal antigen 2 OS=Homo sapiens GN=BST2 PE=1 SV=1 | BST2 | 20 | 0.44 | 0 | 0 | 0 | 18% | 0 | 0 | 0 | 0 | 0 | 0 | 95% | 0 |
| 518 | Butyrophilin subfamily 3 member A3 OS=Homo sapiens GN=BTN3A3 PE=1 SV=1 | BT3A3 | 65 | 0.052 | 0 | 0 | 0 | 0 | 0 | 0 | 0 | 0 | 0 | 0 | 95% | 95% |
| 519 | Desmocollin-3 OS=Homo sapiens GN=DSC3 PE=1 SV=3 | DSC3 | 100 | 0.44 | 0 | 0 | 0 | 0 | 0 | 0 | 0 | 0 | 0 | 0 | 8% | 95% |
| 520 | Epsin-1 OS=Homo sapiens GN=EPN1 PE=1 SV=2 | EPN1 | 60 | 0.052 | 0 | 95% | 68% | 0 | 0 | 0 | 0 | 0 | 0 | 0 | 0 | 0 |
| 521 | Integral membrane protein 2B OS=Homo sapiens GN=ITM2B PE=1 SV=1 | ITM2B | 30 | 0.44 | 0 | 0 | 64% | 0 | 0 | 0 | 0 | 0 | 0 | 0 | 0 | 95% |
| 522 | Integral membrane protein 2C OS=Homo sapiens GN=ITM2C PE=1 SV=1 | ITM2C | 30 | 0.44 | 0 | 0 | 95% | 0 | 0 | 0 | 0 | 0 | 0 | 0 | 0 | 0 |
| 523 | Leptin receptor OS=Homo sapiens GN=LEPR PE=1 SV=2 | LEPR | 132 | 0.44 | 0 | 7% | 95% | 0 | 0 | 0 | 0 | 0 | 0 | 0 | 0 | 0 |
| 524 | Sodium/calcium exchanger 1 OS=Homo sapiens GN=SLC8A1 PE=1 SV=3 | NAC1 | 109 | 0.056 | 95% | 0 | 95% | 0 | 0 | 0 | 0 | 0 | 0 | 0 | 0 | 0 |
| 525 | Nucleoporin NDC1 OS=Homo sapiens GN=NDC1 PE=1 SV=2 | NDC1 | 76 | 0.44 | 0 | 0 | 0 | 42% | 0 | 0 | 0 | 0 | 0 | 0 | 0 | 95% |
| 526 | Phosphatidylinositol glycan anchor biosynthesis class U protein OS=Homo sapiens GN=PIGU PE=1 SV=3 | PIGU | 50 | 0.6 | 95% | 0 | 0 | 0 | 0 | 0 | 0 | 95% | 0 | 0 | 0 | 0 |
| 527 | Sodium-coupled neutral amino acid transporter 5 OS=Homo sapiens GN=SLC38A5 PE=1 SV=1 | S38A5 | 51 | 0.44 | 0 | 0 | 0 | 0 | 0 | 0 | 95% | 0 | 0 | 0 | 0 | 0 |
| 528 | Zinc transporter ZIP1 OS=Homo sapiens GN=SLC39A1 PE=1 SV=1 | S39A1 | 34 | 0.052 | 0 | 0 | 0 | 0 | 0 | 0 | 95% | 95% | 0 | 0 | 0 | 0 |
| 529 | SRSF protein kinase 1 OS=Homo sapiens GN=SRPK1 PE=1 SV=2 | SRPK1 | 74 | 0.44 | 0 | 0 | 0 | 0 | 0 | 0 | 0 | 95% | 0 | 0 | 0 | 8% |
| 530 | Angiopoietin-1 receptor OS=Homo sapiens GN=TEK PE=1 SV=2 | TIE2 | 126 | 0.44 | 0 | 0 | 0 | 0 | 0 | 95% | 0 | 0 | 0 | 0 | 0 | 0 |
| 531 | Transmembrane 7 superfamily member 3 OS=Homo sapiens GN=TM7SF3 PE=2 SV=1 | TM7S3 | 64 | 0.44 | 0 | 0 | 0 | 0 | 0 | 0 | 10% | 95% | 0 | 0 | 0 | 0 |
| 532 | Transmembrane protein 97 OS=Homo sapiens GN=TMEM97 PE=1 SV=1 | TMM97 | 21 | 0.44 | 0 | 0 | 0 | 0 | 0 | 0 | 0 | 0 | 0 | 95% | 7% | 0 |
| 533 | Two pore calcium channel protein 1 OS=Homo sapiens GN=TPCN1 PE=1 SV=3 | TPC1 | 94 | 0.44 | 0 | 0 | 0 | 0 | 0 | 0 | 95% | 0 | 0 | 0 | 0 | 0 |
| 534 | Tumor susceptibility gene 101 protein OS=Homo sapiens GN=TSG101 PE=1 SV=2 | TS101 | 44 | 0.052 | 0 | 0 | 0 | 0 | 0 | 0 | 0 | 95% | 95% | 0 | 0 | 0 |
| 535 | Serine/threonine-protein kinase WNK2 OS=Homo sapiens GN=WNK2 PE=1 SV=4 | WNK2 | 243 | 0.44 | 0 | 0 | 0 | 0 | 0 | 0 | 0 | 0 | 0 | 0 | 95% | 0 |
| 536 | Glutamyl aminopeptidase OS=Homo sapiens GN=ENPEP PE=1 SV=3 | AMPE | 109 | 0.44 | 0 | 0 | 0 | 0 | 95% | 0 | 0 | 0 | 0 | 0 | 0 | 0 |
| 537 | Diacylglycerol kinase epsilon OS=Homo sapiens GN=DGKE PE=1 SV=1 | DGKE | 64 | 0.44 | 0 | 0 | 0 | 0 | 0 | 95% | 0 | 0 | 0 | 0 | 0 | 0 |
| 538 | Endoplasmic reticulum aminopeptidase 2 OS=Homo sapiens GN=ERAP2 PE=1 SV=2 | ERAP2 | 110 | 0.44 | 0 | 0 | 0 | 0 | 0 | 0 | 0 | 95% | 0 | 0 | 0 | 0 |
| 539 | Multidrug resistance protein 3 OS=Homo sapiens GN=ABCB4 PE=1 SV=2 | MDR3 | 142 | 0.44 | 0 | 0 | 0 | 0 | 0 | 0 | 95% | 0 | 0 | 0 | 0 | 0 |
| 540 | Molybdate-anion transporter OS=Homo sapiens GN=MFSD5 PE=1 SV=2 | MFSD5 | 50 | 0.44 | 0 | 0 | 0 | 0 | 0 | 0 | 95% | 0 | 0 | 0 | 0 | 0 |
| 541 | Sodium/hydrogen exchanger 1 OS=Homo sapiens GN=SLC9A1 PE=1 SV=2 | SL9A1 | 91 | 0.44 | 0 | 95% | 0 | 0 | 0 | 0 | 0 | 0 | 0 | 0 | 0 | 0 |
| 542 | Signal recognition particle subunit SRP72 OS=Homo sapiens GN=SRP72 PE=1 SV=3 | SRP72 | 75 | 0.44 | 0 | 0 | 0 | 0 | 0 | 95% | 0 | 0 | 0 | 0 | 0 | 0 |
| 543 | Tax1-binding protein 3 OS=Homo sapiens GN=TAX1BP3 PE=1 SV=2 | TX1B3 | 14 | 0.44 | 0 | 95% | 0 | 0 | 0 | 0 | 0 | 0 | 0 | 0 | 0 | 0 |
| 544 | Urokinase plasminogen activator surface receptor OS=Homo sapiens GN=PLAUR PE=1 SV=1 | UPAR | 37 | 0.44 | 0 | 0 | 0 | 0 | 95% | 0 | 0 | 0 | 0 | 0 | 0 | 0 |
| 545 | D(3) dopamine receptor OS=Homo sapiens GN=DRD3 PE=1 SV=2 | DRD3 | 44 | 0.44 | 0 | 0 | 0 | 0 | 0 | 0 | 0 | 93% | 0 | 0 | 0 | 0 |
| 546 | Methionine aminopeptidase 2 OS=Homo sapiens GN=METAP2 PE=1 SV=1 | MAP2 | 53 | 0.44 | 0 | 0 | 0 | 0 | 0 | 0 | 0 | 0 | 93% | 0 | 0 | 0 |
| 547 | Oncostatin-M-specific receptor subunit beta OS=Homo sapiens GN=OSMR PE=1 SV=1 | OSMR | 111 | 0.44 | 0 | 0 | 0 | 0 | 0 | 0 | 0 | 0 | 0 | 100% | 48% | 55% |
| 548 | Calnexin OS=Homo sapiens GN=CANX PE=1 SV=2 | CALX | 68 | 0.68 | 100% | 100% | 100% | 100% | 100% | 100% | 100% | 100% | 100% | 100% | 100% | 100% |
| 549 | Cluster of Tubulin beta chain OS=Homo sapiens GN=TUBB PE=1 SV=2 (TBB5_HUMAN) | TBB5 [7] | 50 | 0.61 | 100% | 100% | 100% | 100% | 100% | 100% | 100% | 100% | 100% | 100% | 100% | 100% |
| 550 | Protein disulfide-isomerase A3 OS=Homo sapiens GN=PDIA3 PE=1 SV=4 | PDIA3 | 57 | 0.002 | 100% | 100% | 100% | 100% | 100% | 100% | 100% | 100% | 100% | 100% | 100% | 100% |
| 551 | Transitional endoplasmic reticulum ATPase OS=Homo sapiens GN=VCP PE=1 SV=4 | TERA | 89 | 0.0048 | 100% | 100% | 100% | 100% | 100% | 100% | 100% | 100% | 100% | 100% | 100% | 100% |
| 552 | Cluster of Tubulin alpha-4A chain OS=Homo sapiens GN=TUBA4A PE=1 SV=1 (TBA4A_HUMAN) | TBA4A [4] | 50 | 0.014 | 100% | 100% | 100% | 100% | 100% | 100% | 100% | 100% | 100% | 100% | 100% | 100% |
| 553 | Voltage-dependent anion-selective channel protein 2 OS=Homo sapiens GN=VDAC2 PE=1 SV=2 | VDAC2 | 32 | 0.29 | 100% | 100% | 100% | 100% | 100% | 100% | 100% | 100% | 100% | 100% | 100% | 100% |
| 554 | T-complex protein 1 subunit beta OS=Homo sapiens GN=CCT2 PE=1 SV=4 | TCPB | 57 | 0.0027 | 100% | 100% | 100% | 100% | 100% | 100% | 100% | 100% | 100% | 100% | 100% | 100% |
| 555 | Dihydrolipoyllysine-residue succinyltransferase component of 2-oxoglutarate dehydrogenase complex, mitochondrial OS=Homo sapiens GN=DLST PE=1 SV=4 | ODO2 | 49 | 0.0074 | 100% | 100% | 100% | 100% | 100% | 100% | 100% | 100% | 100% | 100% | 100% | 100% |
| 556 | T-complex protein 1 subunit alpha OS=Homo sapiens GN=TCP1 PE=1 SV=1 | TCPA | 60 | 0.0054 | 100% | 100% | 100% | 100% | 100% | 100% | 100% | 100% | 100% | 100% | 100% | 100% |
| 557 | Cluster of L-lactate dehydrogenase A chain OS=Homo sapiens GN=LDHA PE=1 SV=2 (LDHA_HUMAN) | LDHA [4] | 37 | 0.00036 | 100% | 100% | 100% | 100% | 100% | 100% | 100% | 100% | 100% | 100% | 100% | 100% |
| 558 | T-complex protein 1 subunit epsilon OS=Homo sapiens GN=CCT5 PE=1 SV=1 | TCPE | 60 | 0.34 | 100% | 100% | 100% | 100% | 100% | 100% | 100% | 100% | 100% | 100% | 100% | 100% |
| 559 | Guanine nucleotide-binding protein G(I)/G(S)/G(T) subunit beta-2 OS=Homo sapiens GN=GNB2 PE=1 SV=3 | GBB2 | 37 | 0.32 | 100% | 100% | 100% | 100% | 100% | 100% | 100% | 100% | 100% | 100% | 100% | 100% |
| 560 | Dihydrolipoyllysine-residue acetyltransferase component of pyruvate dehydrogenase complex, mitochondrial OS=Homo sapiens GN=DLAT PE=1 SV=3 | ODP2 | 69 | 0.041 | 100% | 100% | 100% | 100% | 100% | 100% | 100% | 100% | 100% | 100% | 100% | 100% |
| 561 | Cluster of Alpha-soluble NSF attachment protein OS=Homo sapiens GN=NAPA PE=1 SV=3 (SNAA_HUMAN) | SNAA [2] | 33 | 0.1 | 100% | 100% | 100% | 100% | 100% | 100% | 100% | 100% | 100% | 100% | 100% | 100% |
| 562 | Cluster of Dihydropyrimidinase-related protein 2 OS=Homo sapiens GN=DPYSL2 PE=1 SV=1 (DPYL2_HUMAN) | DPYL2 [2] | 62 | 0.091 | 100% | 100% | 100% | 100% | 100% | 100% | 100% | 100% | 100% | 100% | 100% | 100% |
| 563 | Cytochrome b-c1 complex subunit 1, mitochondrial OS=Homo sapiens GN=UQCRC1 PE=1 SV=3 | QCR1 | 53 | 0.0011 | 100% | 100% | 100% | 100% | 100% | 100% | 100% | 95% | 98% | 100% | 100% | 100% |
| 564 | Programmed cell death 6-interacting protein OS=Homo sapiens GN=PDCD6IP PE=1 SV=1 | PDC6I | 96 | 0.0068 | 100% | 100% | 100% | 0 | 100% | 100% | 100% | 100% | 100% | 100% | 100% | 100% |
| 565 | Stress-70 protein, mitochondrial OS=Homo sapiens GN=HSPA9 PE=1 SV=2 | GRP75 | 74 | 0.66 | 100% | 100% | 100% | 25% | 100% | 100% | 100% | 100% | 100% | 100% | 100% | 100% |
| 566 | NADH-ubiquinone oxidoreductase 75 kDa subunit, mitochondrial OS=Homo sapiens GN=NDUFS1 PE=1 SV=3 | NDUS1 | 79 | 0.0053 | 100% | 100% | 100% | 100% | 100% | 100% | 0 | 98% | 98% | 100% | 100% | 100% |
| 567 | Cytochrome b-c1 complex subunit 2, mitochondrial OS=Homo sapiens GN=UQCRC2 PE=1 SV=3 | QCR2 | 48 | 0.0005 | 100% | 100% | 100% | 100% | 100% | 100% | 100% | 100% | 99% | 100% | 100% | 100% |
| 568 | Serum albumin OS=Homo sapiens GN=ALB PE=1 SV=2 | ALBU | 69 | 0.00027 | 100% | 95% | 100% | 100% | 100% | 100% | 100% | 100% | 100% | 100% | 95% | 100% |
| 569 | Cluster of Septin-11 OS=Homo sapiens GN=SEPT11 PE=1 SV=3 (SEP11_HUMAN) | SEP11 [3] | 49 | 0.021 | 100% | 100% | 100% | 100% | 100% | 76% | 100% | 100% | 100% | 100% | 100% | 100% |
| 570 | Dihydrolipoyl dehydrogenase, mitochondrial OS=Homo sapiens GN=DLD PE=1 SV=2 | DLDH | 54 | 0.37 | 95% | 100% | 100% | 100% | 100% | 100% | 100% | 100% | 100% | 100% | 100% | 100% |
| 571 | ATP synthase subunit gamma, mitochondrial OS=Homo sapiens GN=ATP5C1 PE=1 SV=1 | ATPG | 33 | < 0.00010 | 95% | 95% | 95% | 100% | 100% | 100% | 100% | 99% | 95% | 100% | 100% | 100% |
| 572 | Transketolase OS=Homo sapiens GN=TKT PE=1 SV=3 | TKT | 68 | 0.0029 | 100% | 100% | 100% | 0 | 0 | 0 | 100% | 100% | 100% | 100% | 100% | 100% |
| 573 | D-3-phosphoglycerate dehydrogenase OS=Homo sapiens GN=PHGDH PE=1 SV=4 | SERA | 57 | 0.36 | 95% | 100% | 100% | 100% | 100% | 100% | 100% | 100% | 100% | 100% | 100% | 77% |
| 574 | ATP synthase F(0) complex subunit B1, mitochondrial OS=Homo sapiens GN=ATP5F1 PE=1 SV=2 | AT5F1 | 29 | 0.038 | 0 | 0 | 100% | 12% | 100% | 100% | 0 | 0 | 0 | 100% | 100% | 100% |
| 575 | NADH dehydrogenase [ubiquinone] iron-sulfur protein 3, mitochondrial OS=Homo sapiens GN=NDUFS3 PE=1 SV=1 | NDUS3 | 30 | 0.044 | 95% | 100% | 100% | 100% | 100% | 100% | 0 | 100% | 0 | 100% | 95% | 100% |
| 576 | Alpha-centractin OS=Homo sapiens GN=ACTR1A PE=1 SV=1 | ACTZ | 43 | 0.41 | 95% | 100% | 95% | 100% | 100% | 98% | 95% | 97% | 95% | 95% | 100% | 100% |
| 577 | Succinate dehydrogenase [ubiquinone] flavoprotein subunit, mitochondrial OS=Homo sapiens GN=SDHA PE=1 SV=2 | SDHA | 73 | 0.0066 | 0 | 0 | 100% | 100% | 100% | 100% | 0 | 0 | 0 | 100% | 100% | 100% |
| 578 | Peroxiredoxin-1 OS=Homo sapiens GN=PRDX1 PE=1 SV=1 | PRDX1 | 22 | 0.031 | 99% | 100% | 100% | 100% | 99% | 100% | 100% | 100% | 100% | 93% | 6% | 90% |
| 579 | Gamma-soluble NSF attachment protein OS=Homo sapiens GN=NAPG PE=1 SV=1 | SNAG | 35 | 0.13 | 95% | 95% | 85% | 95% | 100% | 95% | 100% | 100% | 100% | 100% | 100% | 99% |
| 580 | Cluster of Rab GDP dissociation inhibitor alpha OS=Homo sapiens GN=GDI1 PE=1 SV=2 (GDIA_HUMAN) | GDIA [2] | 51 | 0.45 | 95% | 100% | 0 | 0 | 0 | 95% | 99% | 0 | 0 | 100% | 95% | 98% |
| 581 | Pyruvate dehydrogenase E1 component subunit alpha, somatic form, mitochondrial OS=Homo sapiens GN=PDHA1 PE=1 SV=3 | ODPA | 43 | 0.38 | 0 | 0 | 0 | 0 | 70% | 95% | 99% | 0 | 100% | 100% | 100% | 12% |
| 582 | Calcium-binding mitochondrial carrier protein Aralar1 OS=Homo sapiens GN=SLC25A12 PE=1 SV=2 | CMC1 | 75 | 0.0029 | 91% | 99% | 0 | 100% | 100% | 100% | 0 | 0 | 0 | 100% | 99% | 100% |
| 583 | Cluster of Nucleoside diphosphate kinase A OS=Homo sapiens GN=NME1 PE=1 SV=1 (NDKA_HUMAN) | NDKA [2] | 17 | 0.28 | 0 | 100% | 100% | 0 | 95% | 68% | 100% | 95% | 100% | 95% | 0 | 0 |
| 584 | NADH dehydrogenase [ubiquinone] 1 alpha subcomplex subunit 10, mitochondrial OS=Homo sapiens GN=NDUFA10 PE=1 SV=1 | NDUAA | 41 | 0.11 | 0 | 0 | 19% | 0 | 95% | 95% | 0 | 0 | 0 | 100% | 100% | 18% |
| 585 | Macrophage migration inhibitory factor OS=Homo sapiens GN=MIF PE=1 SV=4 | MIF | 12 | 0.36 | 100% | 100% | 57% | 0 | 0 | 0 | 0 | 95% | 0 | 99% | 75% | 18% |
| 586 | Cytochrome c oxidase subunit 5A, mitochondrial OS=Homo sapiens GN=COX5A PE=1 SV=2 | COX5A | 17 | 0.052 | 0 | 0 | 0 | 6% | 9% | 0 | 0 | 0 | 0 | 100% | 0 | 100% |
| 587 | Thioredoxin-dependent peroxide reductase, mitochondrial OS=Homo sapiens GN=PRDX3 PE=1 SV=3 | PRDX3 | 28 | 0.33 | 0 | 0 | 95% | 0 | 0 | 0 | 0 | 100% | 8% | 95% | 95% | 95% |
| 588 | Malate dehydrogenase, cytoplasmic OS=Homo sapiens GN=MDH1 PE=1 SV=4 | MDHC | 36 | 0.12 | 16% | 95% | 95% | 0 | 0 | 0 | 36% | 0 | 0 | 95% | 95% | 0 |
| 589 | Cytochrome b-c1 complex subunit Rieske, mitochondrial OS=Homo sapiens GN=UQCRFS1 PE=1 SV=2 | UCRI | 30 | 0.44 | 0 | 0 | 0 | 0 | 90% | 7% | 0 | 0 | 0 | 95% | 0 | 0 |
| 590 | Ras GTPase-activating-like protein IQGAP3 OS=Homo sapiens GN=IQGAP3 PE=1 SV=2 | IQGA3 | 185 | 0.0048 | 0 | 0 | 0 | 100% | 100% | 100% | 100% | 52% | 0 | 0 | 0 | 10% |
| 591 | Laminin subunit beta-1 OS=Homo sapiens GN=LAMB1 PE=1 SV=2 | LAMB1 | 198 | 0.05 | 0 | 0 | 0 | 95% | 100% | 0 | 0 | 0 | 0 | 95% | 95% | 100% |
| 592 | Neurofibromin OS=Homo sapiens GN=NF1 PE=1 SV=2 | NF1 | 319 | 0.6 | 10% | 48% | 66% | 0 | 78% | 98% | 0 | 0 | 0 | 17% | 100% | 53% |
| 593 | Ras GTPase-activating protein nGAP OS=Homo sapiens GN=RASAL2 PE=1 SV=2 | NGAP | 129 | 0.44 | 0 | 0 | 0 | 0 | 0 | 0 | 95% | 17% | 0 | 0 | 0 | 0 |
| 594 | Spectrin alpha chain, erythrocytic 1 OS=Homo sapiens GN=SPTA1 PE=1 SV=5 | SPTA1 | 280 | 0.44 | 0 | 6% | 0 | 0 | 0 | 0 | 0 | 0 | 0 | 95% | 13% | 0 |
| 595 | Ras-related protein Rab-21 OS=Homo sapiens GN=RAB21 PE=1 SV=3 | RAB21 | 24 | 0.063 | 100% | 100% | 100% | 100% | 100% | 100% | 100% | 100% | 100% | 100% | 100% | 100% |
| 596 | TNF receptor-associated factor 2 OS=Homo sapiens GN=TRAF2 PE=1 SV=2 | TRAF2 | 56 | 0.013 | 0 | 5% | 0 | 60% | 99% | 12% | 65% | 12% | 45% | 95% | 96% | 87% |
| 597 | Glucose-6-phosphate 1-dehydrogenase OS=Homo sapiens GN=G6PD PE=1 SV=4 | G6PD | 59 | 0.44 | 0 | 0 | 0 | 0 | 0 | 0 | 0 | 0 | 95% | 0 | 0 | 0 |
| 598 | Toll-interacting protein OS=Homo sapiens GN=TOLLIP PE=1 SV=1 | TOLIP | 30 | 0.59 | 0 | 0 | 0 | 95% | 0 | 0 | 54% | 17% | 42% | 95% | 0 | 0 |
| 599 | Protein LYRIC OS=Homo sapiens GN=MTDH PE=1 SV=2 | LYRIC | 64 | 0.03 | 100% | 100% | 100% | 100% | 100% | 100% | 100% | 100% | 100% | 100% | 100% | 100% |
| 600 | Cluster of 14-3-3 protein zeta/delta OS=Homo sapiens GN=YWHAZ PE=1 SV=1 (1433Z_HUMAN) | 1433Z [7] | 28 | 0.025 | 100% | 100% | 100% | 100% | 100% | 100% | 100% | 100% | 100% | 100% | 100% | 100% |
| 601 | Cluster of Myosin-7B OS=Homo sapiens GN=MYH7B PE=1 SV=3 (MYH7B_HUMAN) | MYH7B [8] | 221 | 0.59 | 95% | 45% | 93% | 45% | 0 | 100% | 73% | 46% | 80% | 89% | 90% | 0 |
| 602 | Integrin alpha-7 OS=Homo sapiens GN=ITGA7 PE=1 SV=3 | ITA7 | 129 | 0.0026 | 100% | 100% | 100% | 100% | 100% | 100% | 100% | 100% | 100% | 100% | 100% | 100% |
| 603 | Heterogeneous nuclear ribonucleoprotein M OS=Homo sapiens GN=HNRNPM PE=1 SV=3 | HNRPM | 78 | < 0.00010 | 100% | 100% | 100% | 100% | 100% | 100% | 100% | 100% | 100% | 100% | 100% | 100% |
| 604 | Cluster of Sarcoplasmic/endoplasmic reticulum calcium ATPase 2 OS=Homo sapiens GN=ATP2A2 PE=1 SV=1 (AT2A2_HUMAN) | AT2A2 [3] | 115 | 0.14 | 100% | 100% | 100% | 100% | 100% | 100% | 100% | 100% | 100% | 100% | 100% | 100% |
| 605 | Kinectin OS=Homo sapiens GN=KTN1 PE=1 SV=1 | KTN1 | 156 | < 0.00010 | 100% | 100% | 100% | 100% | 100% | 100% | 100% | 100% | 100% | 100% | 100% | 100% |
| 606 | Cluster of ADP/ATP translocase 2 OS=Homo sapiens GN=SLC25A5 PE=1 SV=7 (ADT2_HUMAN) | ADT2 [4] | 33 | 0.0015 | 100% | 100% | 100% | 100% | 100% | 100% | 100% | 100% | 100% | 100% | 100% | 100% |
| 607 | B-cell receptor-associated protein 31 OS=Homo sapiens GN=BCAP31 PE=1 SV=3 | BAP31 | 28 | 0.087 | 100% | 100% | 100% | 100% | 100% | 100% | 100% | 100% | 100% | 100% | 100% | 100% |
| 608 | Vesicular integral-membrane protein VIP36 OS=Homo sapiens GN=LMAN2 PE=1 SV=1 | LMAN2 | 40 | 0.13 | 100% | 100% | 100% | 100% | 100% | 100% | 100% | 100% | 100% | 100% | 100% | 100% |
| 609 | C-type mannose receptor 2 OS=Homo sapiens GN=MRC2 PE=1 SV=2 | MRC2 | 167 | 0.016 | 100% | 100% | 100% | 100% | 100% | 100% | 100% | 100% | 100% | 100% | 100% | 100% |
| 610 | Phosphate carrier protein, mitochondrial OS=Homo sapiens GN=SLC25A3 PE=1 SV=2 | MPCP | 40 | 0.019 | 100% | 100% | 100% | 100% | 100% | 100% | 100% | 100% | 100% | 100% | 100% | 100% |
| 611 | Calcium-transporting ATPase type 2C member 1 OS=Homo sapiens GN=ATP2C1 PE=1 SV=3 | AT2C1 | 101 | < 0.00010 | 100% | 100% | 100% | 100% | 100% | 100% | 100% | 100% | 100% | 100% | 100% | 100% |
| 612 | Leucine-rich repeat-containing protein 32 OS=Homo sapiens GN=LRRC32 PE=1 SV=1 | LRC32 | 72 | 0.026 | 100% | 100% | 100% | 100% | 100% | 100% | 100% | 100% | 100% | 100% | 95% | 100% |
| 613 | Transmembrane 9 superfamily member 2 OS=Homo sapiens GN=TM9SF2 PE=1 SV=1 | TM9S2 | 76 | 0.98 | 100% | 100% | 100% | 100% | 100% | 100% | 100% | 100% | 100% | 100% | 100% | 100% |
| 614 | Fatty acid desaturase 2 OS=Homo sapiens GN=FADS2 PE=1 SV=1 | FADS2 | 52 | 0.01 | 0 | 100% | 100% | 100% | 100% | 100% | 100% | 100% | 100% | 100% | 100% | 100% |
| 615 | Syntaxin-8 OS=Homo sapiens GN=STX8 PE=1 SV=2 | STX8 | 27 | 0.24 | 100% | 100% | 100% | 100% | 100% | 100% | 100% | 100% | 100% | 100% | 98% | 100% |
| 616 | Cation-dependent mannose-6-phosphate receptor OS=Homo sapiens GN=M6PR PE=1 SV=1 | MPRD | 31 | 0.77 | 100% | 100% | 100% | 100% | 100% | 100% | 100% | 100% | 100% | 100% | 100% | 100% |
| 617 | Trophoblast glycoprotein OS=Homo sapiens GN=TPBG PE=1 SV=1 | TPBG | 46 | 0.39 | 100% | 100% | 100% | 100% | 100% | 100% | 100% | 100% | 100% | 100% | 100% | 100% |
| 618 | Myelin protein zero-like protein 1 OS=Homo sapiens GN=MPZL1 PE=1 SV=1 | MPZL1 | 29 | 0.039 | 100% | 100% | 100% | 100% | 100% | 100% | 100% | 95% | 100% | 100% | 100% | 100% |
| 619 | Probable cation-transporting ATPase 13A1 OS=Homo sapiens GN=ATP13A1 PE=1 SV=2 | AT131 | 133 | 0.3 | 81% | 100% | 100% | 100% | 100% | 100% | 100% | 100% | 30% | 100% | 98% | 100% |
| 620 | Sphingolipid delta(4)-desaturase DES1 OS=Homo sapiens GN=DEGS1 PE=1 SV=1 | DEGS1 | 38 | 0.034 | 100% | 100% | 100% | 95% | 95% | 100% | 100% | 100% | 100% | 100% | 100% | 100% |
| 621 | Transmembrane protein 245 OS=Homo sapiens GN=TMEM245 PE=1 SV=2 | TM245 | 101 | 0.027 | 95% | 100% | 15% | 100% | 100% | 100% | 100% | 100% | 100% | 100% | 100% | 100% |
| 622 | Calcium-binding mitochondrial carrier protein Aralar2 OS=Homo sapiens GN=SLC25A13 PE=1 SV=2 | CMC2 | 74 | < 0.00010 | 95% | 66% | 100% | 100% | 100% | 100% | 100% | 0 | 95% | 100% | 100% | 100% |
| 623 | Metalloreductase STEAP3 OS=Homo sapiens GN=STEAP3 PE=1 SV=2 | STEA3 | 55 | 0.015 | 94% | 95% | 95% | 100% | 100% | 100% | 0 | 0 | 0 | 100% | 100% | 100% |
| 624 | Tetraspanin-3 OS=Homo sapiens GN=TSPAN3 PE=2 SV=1 | TSN3 | 28 | 0.044 | 95% | 13% | 100% | 100% | 100% | 100% | 100% | 100% | 100% | 100% | 13% | 90% |
| 625 | Golgi membrane protein 1 OS=Homo sapiens GN=GOLM1 PE=1 SV=1 | GOLM1 | 45 | 0.017 | 0 | 95% | 15% | 99% | 100% | 99% | 100% | 100% | 100% | 99% | 68% | 51% |
| 626 | Discoidin, CUB and LCCL domain-containing protein 2 OS=Homo sapiens GN=DCBLD2 PE=1 SV=1 | DCBD2 | 85 | 0.0021 | 100% | 100% | 100% | 95% | 100% | 95% | 0 | 0 | 0 | 95% | 95% | 99% |
| 627 | Acetyl-coenzyme A transporter 1 OS=Homo sapiens GN=SLC33A1 PE=1 SV=1 | ACATN | 61 | 0.09 | 95% | 95% | 95% | 95% | 0 | 95% | 95% | 100% | 0 | 100% | 100% | 100% |
| 628 | Mas-related G-protein coupled receptor member F OS=Homo sapiens GN=MRGPRF PE=2 SV=1 | MRGRF | 38 | 0.073 | 89% | 99% | 0 | 0 | 0 | 0 | 0 | 0 | 0 | 0 | 0 | 0 |
| 629 | B-cell receptor-associated protein 29 OS=Homo sapiens GN=BCAP29 PE=1 SV=2 | BAP29 | 28 | 0.44 | 0 | 100% | 87% | 0 | 95% | 46% | 0 | 62% | 0 | 97% | 84% | 24% |
| 630 | Synaptophysin-like protein 1 OS=Homo sapiens GN=SYPL1 PE=1 SV=1 | SYPL1 | 29 | 0.47 | 95% | 100% | 95% | 95% | 0 | 95% | 95% | 95% | 95% | 95% | 95% | 95% |
| 631 | Solute carrier family 2, facilitated glucose transporter member 10 OS=Homo sapiens GN=SLC2A10 PE=1 SV=2 | GTR10 | 57 | 0.42 | 0 | 0 | 66% | 95% | 100% | 64% | 99% | 95% | 65% | 95% | 62% | 65% |
| 632 | Protein canopy homolog 2 OS=Homo sapiens GN=CNPY2 PE=1 SV=1 | CNPY2 | 21 | 0.061 | 95% | 100% | 95% | 95% | 95% | 95% | 95% | 95% | 95% | 95% | 0 | 0 |
| 633 | Mitochondrial 2-oxoglutarate/malate carrier protein OS=Homo sapiens GN=SLC25A11 PE=1 SV=3 | M2OM | 34 | 0.017 | 0 | 0 | 0 | 100% | 0 | 95% | 0 | 0 | 10% | 95% | 100% | 95% |
| 634 | Butyrophilin subfamily 2 member A1 OS=Homo sapiens GN=BTN2A1 PE=1 SV=3 | BT2A1 | 60 | 0.008 | 0 | 0 | 0 | 95% | 98% | 0 | 0 | 0 | 0 | 93% | 100% | 100% |
| 635 | Zinc transporter ZIP10 OS=Homo sapiens GN=SLC39A10 PE=1 SV=2 | S39AA | 94 | 0.29 | 95% | 0 | 0 | 95% | 100% | 90% | 0 | 0 | 0 | 91% | 95% | 0 |
| 636 | Probable cation-transporting ATPase 13A3 OS=Homo sapiens GN=ATP13A3 PE=1 SV=4 | AT133 | 138 | 0.52 | 0 | 0 | 0 | 95% | 95% | 0 | 95% | 98% | 14% | 95% | 95% | 0 |
| 637 | Tetraspanin-10 OS=Homo sapiens GN=TSPAN10 PE=2 SV=1 | TSN10 | 36 | 0.44 | 0 | 18% | 66% | 64% | 32% | 64% | 25% | 0 | 25% | 95% | 53% | 40% |
| 638 | Cluster of Protein NipSnap homolog 2 OS=Homo sapiens GN=GBAS PE=1 SV=1 (NIPS2_HUMAN) | NIPS2 [2] | 34 | 0.056 | 100% | 0 | 100% | 0 | 10% | 0 | 14% | 0 | 0 | 0 | 0 | 0 |
| 639 | Starch-binding domain-containing protein 1 OS=Homo sapiens GN=STBD1 PE=1 SV=1 | STBD1 | 39 | 0.4 | 8% | 99% | 100% | 0 | 100% | 29% | 0 | 0 | 0 | 0 | 0 | 0 |
| 640 | Caprin-1 OS=Homo sapiens GN=CAPRIN1 PE=1 SV=2 | CAPR1 | 78 | 0.22 | 0 | 0 | 93% | 0 | 95% | 100% | 0 | 95% | 0 | 0 | 0 | 0 |
| 641 | Protein FAM26E OS=Homo sapiens GN=FAM26E PE=2 SV=1 | FA26E | 35 | 0.0019 | 0 | 0 | 95% | 0 | 0 | 0 | 95% | 95% | 95% | 95% | 95% | 95% |
| 642 | Cystine/glutamate transporter OS=Homo sapiens GN=SLC7A11 PE=1 SV=1 | XCT | 55 | 0.043 | 0 | 0 | 95% | 0 | 0 | 0 | 95% | 95% | 95% | 95% | 95% | 0 |
| 643 | Receptor-type tyrosine-protein phosphatase gamma OS=Homo sapiens GN=PTPRG PE=1 SV=4 | PTPRG | 162 | 0.44 | 0 | 0 | 100% | 0 | 0 | 0 | 0 | 0 | 0 | 0 | 0 | 0 |
| 644 | Sodium- and chloride-dependent taurine transporter OS=Homo sapiens GN=SLC6A6 PE=1 SV=2 | SC6A6 | 70 | 0.097 | 0 | 0 | 0 | 0 | 0 | 0 | 0 | 0 | 0 | 0 | 98% | 95% |
| 645 | Probable cation-transporting ATPase 13A4 OS=Homo sapiens GN=ATP13A4 PE=2 SV=3 | AT134 | 134 | 0.44 | 0 | 0 | 0 | 100% | 0 | 0 | 0 | 0 | 0 | 0 | 0 | 0 |
| 646 | Protein JTB OS=Homo sapiens GN=JTB PE=1 SV=1 | JTB | 16 | < 0.00010 | 0 | 0 | 0 | 0 | 0 | 0 | 95% | 100% | 95% | 0 | 0 | 0 |
| 647 | Bax inhibitor 1 OS=Homo sapiens GN=TMBIM6 PE=1 SV=2 | BI1 | 27 | 0.6 | 0 | 0 | 0 | 0 | 28% | 95% | 0 | 0 | 0 | 9% | 0 | 95% |
| 648 | Serine/threonine-protein kinase DCLK1 OS=Homo sapiens GN=DCLK1 PE=1 SV=2 | DCLK1 | 82 | 0.052 | 0 | 0 | 0 | 0 | 5% | 0 | 0 | 95% | 95% | 0 | 0 | 0 |
| 649 | Receptor-type tyrosine-protein phosphatase mu OS=Homo sapiens GN=PTPRM PE=1 SV=2 | PTPRM | 164 | 0.59 | 0 | 95% | 0 | 0 | 0 | 95% | 0 | 0 | 0 | 0 | 0 | 0 |
| 650 | Solute carrier family 2, facilitated glucose transporter member 6 OS=Homo sapiens GN=SLC2A6 PE=1 SV=2 | GTR6 | 55 | 0.44 | 0 | 0 | 0 | 0 | 0 | 0 | 12% | 0 | 95% | 0 | 0 | 0 |
| 651 | Disintegrin and metalloproteinase domain-containing protein 29 OS=Homo sapiens GN=ADAM29 PE=1 SV=3 | ADA29 | 93 | 0.44 | 0 | 0 | 0 | 0 | 0 | 0 | 0 | 0 | 99% | 0 | 0 | 0 |
| 652 | Retinal-specific ATP-binding cassette transporter OS=Homo sapiens GN=ABCA4 PE=1 SV=3 | ABCA4 | 256 | 0.44 | 0 | 0 | 10% | 0 | 0 | 0 | 0 | 0 | 0 | 95% | 0 | 0 |
| 653 | Probable low affinity copper uptake protein 2 OS=Homo sapiens GN=SLC31A2 PE=1 SV=1 | COPT2 | 16 | 0.052 | 0 | 0 | 0 | 0 | 0 | 0 | 0 | 95% | 95% | 0 | 0 | 0 |
| 654 | Low-density lipoprotein receptor-related protein 12 OS=Homo sapiens GN=LRP12 PE=1 SV=1 | LRP12 | 95 | 0.44 | 0 | 0 | 0 | 0 | 95% | 55% | 0 | 0 | 0 | 0 | 0 | 0 |
| 655 | Proton myo-inositol cotransporter OS=Homo sapiens GN=SLC2A13 PE=1 SV=3 | MYCT | 70 | 0.052 | 0 | 0 | 0 | 0 | 0 | 0 | 95% | 0 | 95% | 0 | 0 | 0 |
| 656 | Probable cation-transporting ATPase 13A2 OS=Homo sapiens GN=ATP13A2 PE=1 SV=2 | AT132 | 129 | 0.44 | 0 | 0 | 0 | 0 | 0 | 0 | 0 | 95% | 0 | 0 | 0 | 0 |
| 657 | Transmembrane protein 11, mitochondrial OS=Homo sapiens GN=TMEM11 PE=1 SV=1 | TMM11 | 22 | 0.44 | 0 | 0 | 0 | 0 | 0 | 0 | 0 | 0 | 0 | 0 | 95% | 0 |
| 658 | Regulator of G-protein signaling 19 OS=Homo sapiens GN=RGS19 PE=1 SV=1 | RGS19 | 25 | 0.44 | 0 | 100% | 0 | 0 | 0 | 0 | 0 | 0 | 0 | 0 | 0 | 7% |
| 659 | Exocyst complex component 4 OS=Homo sapiens GN=EXOC4 PE=1 SV=1 | EXOC4 | 111 | 0.15 | 100% | 100% | 95% | 95% | 95% | 100% | 95% | 100% | 95% | 100% | 100% | 100% |
| 660 | Annexin A6 OS=Homo sapiens GN=ANXA6 PE=1 SV=3 | ANXA6 | 76 | < 0.00010 | 100% | 100% | 100% | 100% | 100% | 100% | 100% | 100% | 100% | 100% | 100% | 100% |
| 661 | 60S ribosomal protein L4 OS=Homo sapiens GN=RPL4 PE=1 SV=5 | RL4 | 48 | 0.28 | 100% | 100% | 100% | 100% | 100% | 100% | 100% | 100% | 100% | 100% | 100% | 100% |
| 662 | 60S ribosomal protein L7 OS=Homo sapiens GN=RPL7 PE=1 SV=1 | RL7 | 29 | 0.024 | 100% | 100% | 100% | 100% | 100% | 100% | 100% | 100% | 100% | 100% | 100% | 100% |
| 663 | Lysosome membrane protein 2 OS=Homo sapiens GN=SCARB2 PE=1 SV=2 | SCRB2 | 54 | 0.00013 | 100% | 100% | 100% | 100% | 100% | 100% | 100% | 100% | 100% | 100% | 100% | 100% |
| 664 | 60S acidic ribosomal protein P0 OS=Homo sapiens GN=RPLP0 PE=1 SV=1 | RLA0 | 34 | 0.21 | 100% | 100% | 100% | 100% | 100% | 100% | 100% | 100% | 100% | 100% | 100% | 100% |
| 665 | Cluster of 60S ribosomal protein L3 OS=Homo sapiens GN=RPL3 PE=1 SV=2 (RL3_HUMAN) | RL3 [2] | 46 | 0.034 | 100% | 100% | 100% | 100% | 100% | 100% | 100% | 100% | 100% | 100% | 100% | 100% |
| 666 | 40S ribosomal protein S3a OS=Homo sapiens GN=RPS3A PE=1 SV=2 | RS3A | 30 | 0.02 | 100% | 100% | 100% | 100% | 100% | 100% | 100% | 100% | 100% | 100% | 100% | 100% |
| 667 | Peptidyl-prolyl cis-trans isomerase B OS=Homo sapiens GN=PPIB PE=1 SV=2 | PPIB | 24 | 0.0018 | 100% | 100% | 100% | 100% | 100% | 100% | 100% | 100% | 100% | 100% | 100% | 100% |
| 668 | 60S ribosomal protein L5 OS=Homo sapiens GN=RPL5 PE=1 SV=3 | RL5 | 34 | 0.21 | 100% | 100% | 100% | 100% | 100% | 100% | 100% | 100% | 100% | 100% | 100% | 100% |
| 669 | 60S ribosomal protein L8 OS=Homo sapiens GN=RPL8 PE=1 SV=2 | RL8 | 28 | 0.048 | 100% | 100% | 100% | 100% | 100% | 100% | 100% | 100% | 100% | 100% | 100% | 100% |
| 670 | 40S ribosomal protein S4, X isoform OS=Homo sapiens GN=RPS4X PE=1 SV=2 | RS4X | 30 | 0.02 | 100% | 100% | 100% | 100% | 100% | 100% | 100% | 100% | 100% | 100% | 100% | 100% |
| 671 | 40S ribosomal protein S8 OS=Homo sapiens GN=RPS8 PE=1 SV=2 | RS8 | 24 | 0.31 | 100% | 100% | 100% | 100% | 100% | 100% | 100% | 100% | 100% | 100% | 100% | 100% |
| 672 | Cluster of Polyadenylate-binding protein 1 OS=Homo sapiens GN=PABPC1 PE=1 SV=2 (PABP1_HUMAN) | PABP1 [3] | 71 | 0.029 | 100% | 100% | 100% | 100% | 100% | 100% | 100% | 100% | 100% | 100% | 100% | 100% |
| 673 | 60S ribosomal protein L10a OS=Homo sapiens GN=RPL10A PE=1 SV=2 | RL10A | 25 | 0.00026 | 100% | 100% | 100% | 100% | 100% | 100% | 100% | 100% | 100% | 100% | 100% | 100% |
| 674 | Filamin-C OS=Homo sapiens GN=FLNC PE=1 SV=3 | FLNC | 291 | 0.3 | 100% | 100% | 100% | 100% | 100% | 100% | 100% | 100% | 100% | 100% | 100% | 100% |
| 675 | Cluster of 60S ribosomal protein L13a OS=Homo sapiens GN=RPL13A PE=1 SV=2 (RL13A_HUMAN) | RL13A [2] | 24 | 0.038 | 100% | 100% | 100% | 100% | 100% | 100% | 100% | 100% | 100% | 100% | 100% | 100% |
| 676 | 40S ribosomal protein S3 OS=Homo sapiens GN=RPS3 PE=1 SV=2 | RS3 | 27 | 0.0037 | 100% | 100% | 100% | 100% | 100% | 100% | 100% | 100% | 100% | 100% | 100% | 100% |
| 677 | 40S ribosomal protein S7 OS=Homo sapiens GN=RPS7 PE=1 SV=1 | RS7 | 22 | 0.31 | 100% | 100% | 100% | 100% | 100% | 100% | 100% | 100% | 100% | 100% | 100% | 100% |
| 678 | Nucleophosmin OS=Homo sapiens GN=NPM1 PE=1 SV=2 | NPM | 33 | 0.0065 | 100% | 100% | 100% | 100% | 100% | 100% | 100% | 100% | 100% | 100% | 100% | 100% |
| 679 | 60S ribosomal protein L12 OS=Homo sapiens GN=RPL12 PE=1 SV=1 | RL12 | 18 | 0.087 | 100% | 100% | 100% | 100% | 100% | 100% | 100% | 100% | 100% | 100% | 100% | 100% |
| 680 | 60S ribosomal protein L27 OS=Homo sapiens GN=RPL27 PE=1 SV=2 | RL27 | 16 | 0.34 | 100% | 100% | 100% | 100% | 100% | 100% | 100% | 100% | 100% | 100% | 100% | 100% |
| 681 | Gelsolin OS=Homo sapiens GN=GSN PE=1 SV=1 | GELS | 86 | 0.2 | 100% | 100% | 100% | 100% | 100% | 100% | 100% | 100% | 100% | 100% | 100% | 100% |
| 682 | 60S ribosomal protein L9 OS=Homo sapiens GN=RPL9 PE=1 SV=1 | RL9 | 22 | 0.07 | 100% | 100% | 100% | 100% | 100% | 100% | 100% | 100% | 100% | 100% | 100% | 100% |
| 683 | 40S ribosomal protein S11 OS=Homo sapiens GN=RPS11 PE=1 SV=3 | RS11 | 18 | 0.079 | 100% | 100% | 100% | 100% | 100% | 100% | 100% | 100% | 100% | 100% | 100% | 100% |
| 684 | 40S ribosomal protein S9 OS=Homo sapiens GN=RPS9 PE=1 SV=3 | RS9 | 23 | 0.2 | 100% | 100% | 100% | 100% | 100% | 100% | 100% | 100% | 100% | 100% | 100% | 100% |
| 685 | LIM domain only protein 7 OS=Homo sapiens GN=LMO7 PE=1 SV=3 | LMO7 | 193 | 0.024 | 100% | 100% | 100% | 100% | 100% | 100% | 100% | 100% | 100% | 100% | 100% | 100% |
| 686 | 40S ribosomal protein S14 OS=Homo sapiens GN=RPS14 PE=1 SV=3 | RS14 | 16 | 0.34 | 100% | 100% | 100% | 100% | 100% | 100% | 100% | 100% | 100% | 100% | 100% | 100% |
| 687 | 40S ribosomal protein S13 OS=Homo sapiens GN=RPS13 PE=1 SV=2 | RS13 | 17 | 0.35 | 100% | 100% | 100% | 100% | 100% | 100% | 100% | 100% | 100% | 100% | 100% | 100% |
| 688 | 60S ribosomal protein L19 OS=Homo sapiens GN=RPL19 PE=1 SV=1 | RL19 | 23 | 0.75 | 100% | 100% | 100% | 100% | 100% | 100% | 100% | 100% | 100% | 100% | 100% | 100% |
| 689 | 40S ribosomal protein S18 OS=Homo sapiens GN=RPS18 PE=1 SV=3 | RS18 | 18 | 0.97 | 100% | 100% | 100% | 100% | 100% | 100% | 100% | 100% | 100% | 100% | 100% | 100% |
| 690 | Heat shock protein beta-1 OS=Homo sapiens GN=HSPB1 PE=1 SV=2 | HSPB1 | 23 | 0.083 | 100% | 100% | 100% | 100% | 100% | 100% | 100% | 100% | 100% | 100% | 100% | 100% |
| 691 | 60S ribosomal protein L18 OS=Homo sapiens GN=RPL18 PE=1 SV=2 | RL18 | 22 | 0.15 | 100% | 100% | 100% | 100% | 100% | 100% | 100% | 100% | 100% | 100% | 100% | 100% |
| 692 | Ras-related protein R-Ras OS=Homo sapiens GN=RRAS PE=1 SV=1 | RRAS | 23 | 0.00094 | 100% | 100% | 100% | 100% | 100% | 100% | 100% | 100% | 100% | 100% | 100% | 100% |
| 693 | 40S ribosomal protein S19 OS=Homo sapiens GN=RPS19 PE=1 SV=2 | RS19 | 16 | 0.48 | 100% | 100% | 100% | 100% | 100% | 100% | 100% | 100% | 100% | 100% | 100% | 100% |
| 694 | Peptidyl-prolyl cis-trans isomerase A OS=Homo sapiens GN=PPIA PE=1 SV=2 | PPIA | 18 | 0.071 | 100% | 100% | 100% | 100% | 100% | 100% | 100% | 100% | 100% | 100% | 100% | 100% |
| 695 | 40S ribosomal protein S16 OS=Homo sapiens GN=RPS16 PE=1 SV=2 | RS16 | 16 | 0.4 | 100% | 100% | 100% | 100% | 100% | 100% | 100% | 100% | 100% | 100% | 100% | 100% |
| 696 | Basement membrane-specific heparan sulfate proteoglycan core protein OS=Homo sapiens GN=HSPG2 PE=1 SV=4 | PGBM | 469 | < 0.00010 | 100% | 100% | 100% | 100% | 100% | 100% | 100% | 100% | 100% | 100% | 100% | 100% |
| 697 | 60S ribosomal protein L23 OS=Homo sapiens GN=RPL23 PE=1 SV=1 | RL23 | 15 | 0.27 | 100% | 100% | 100% | 100% | 100% | 100% | 100% | 100% | 100% | 100% | 100% | 100% |
| 698 | 60S ribosomal protein L22 OS=Homo sapiens GN=RPL22 PE=1 SV=2 | RL22 | 15 | 0.086 | 100% | 100% | 100% | 100% | 100% | 100% | 100% | 100% | 100% | 100% | 100% | 100% |
| 699 | 60S ribosomal protein L30 OS=Homo sapiens GN=RPL30 PE=1 SV=2 | RL30 | 13 | 0.019 | 100% | 100% | 100% | 100% | 100% | 100% | 100% | 100% | 100% | 100% | 100% | 100% |
| 700 | 60S acidic ribosomal protein P2 OS=Homo sapiens GN=RPLP2 PE=1 SV=1 | RLA2 | 12 | 0.013 | 100% | 100% | 100% | 100% | 100% | 100% | 100% | 100% | 100% | 100% | 100% | 100% |
| 701 | Hypoxia up-regulated protein 1 OS=Homo sapiens GN=HYOU1 PE=1 SV=1 | HYOU1 | 111 | 0.00091 | 100% | 100% | 100% | 100% | 100% | 100% | 0 | 97% | 95% | 100% | 100% | 100% |
| 702 | Tenascin OS=Homo sapiens GN=TNC PE=1 SV=3 | TENA | 241 | < 0.00010 | 100% | 100% | 100% | 100% | 0 | 95% | 100% | 100% | 100% | 0 | 0 | 0 |
| 703 | 40S ribosomal protein S5 OS=Homo sapiens GN=RPS5 PE=1 SV=4 | RS5 | 23 | 0.0067 | 100% | 100% | 100% | 100% | 100% | 100% | 100% | 100% | 100% | 100% | 100% | 100% |
| 704 | 60S ribosomal protein L31 OS=Homo sapiens GN=RPL31 PE=1 SV=1 | RL31 | 14 | 0.39 | 100% | 100% | 100% | 100% | 100% | 100% | 100% | 100% | 100% | 100% | 100% | 100% |
| 705 | Ras GTPase-activating protein-binding protein 1 OS=Homo sapiens GN=G3BP1 PE=1 SV=1 | G3BP1 | 52 | 0.012 | 100% | 100% | 100% | 100% | 100% | 100% | 100% | 100% | 100% | 100% | 100% | 100% |
| 706 | Rho-related GTP-binding protein RhoG OS=Homo sapiens GN=RHOG PE=1 SV=1 | RHOG | 21 | 0.56 | 100% | 100% | 100% | 100% | 100% | 100% | 100% | 100% | 100% | 100% | 100% | 100% |
| 707 | 40S ribosomal protein S15 OS=Homo sapiens GN=RPS15 PE=1 SV=2 | RS15 | 17 | 0.12 | 99% | 95% | 100% | 100% | 100% | 100% | 100% | 100% | 100% | 100% | 100% | 100% |
| 708 | Very-long-chain (3R)-3-hydroxyacyl-[acyl-carrier protein] dehydratase 3 OS=Homo sapiens GN=PTPLAD1 PE=1 SV=2 | HACD3 | 43 | 0.24 | 100% | 100% | 100% | 100% | 100% | 100% | 100% | 100% | 31% | 100% | 100% | 100% |
| 709 | 60S acidic ribosomal protein P1 OS=Homo sapiens GN=RPLP1 PE=1 SV=1 | RLA1 | 12 | 0.24 | 95% | 95% | 95% | 100% | 100% | 95% | 95% | 100% | 100% | 100% | 100% | 100% |
| 710 | Ragulator complex protein LAMTOR3 OS=Homo sapiens GN=LAMTOR3 PE=1 SV=1 | LTOR3 | 14 | 0.0017 | 95% | 100% | 100% | 95% | 0 | 95% | 100% | 100% | 100% | 100% | 100% | 100% |
| 711 | 40S ribosomal protein S10 OS=Homo sapiens GN=RPS10 PE=1 SV=1 | RS10 | 19 | 0.0081 | 95% | 100% | 95% | 95% | 100% | 100% | 100% | 100% | 100% | 100% | 100% | 100% |
| 712 | Disintegrin and metalloproteinase domain-containing protein 10 OS=Homo sapiens GN=ADAM10 PE=1 SV=1 | ADA10 | 84 | 0.29 | 65% | 100% | 100% | 95% | 100% | 100% | 95% | 94% | 100% | 100% | 100% | 100% |
| 713 | Ras suppressor protein 1 OS=Homo sapiens GN=RSU1 PE=1 SV=3 | RSU1 | 32 | 0.089 | 95% | 100% | 100% | 100% | 84% | 10% | 100% | 100% | 100% | 100% | 100% | 100% |
| 714 | Ras-related protein R-Ras2 OS=Homo sapiens GN=RRAS2 PE=1 SV=1 | RRAS2 | 23 | 0.1 | 100% | 100% | 100% | 100% | 100% | 100% | 100% | 100% | 99% | 98% | 98% | 99% |
| 715 | Syntaxin-16 OS=Homo sapiens GN=STX16 PE=1 SV=3 | STX16 | 37 | 0.011 | 100% | 100% | 100% | 100% | 100% | 100% | 100% | 100% | 100% | 100% | 100% | 95% |
| 716 | Myristoylated alanine-rich C-kinase substrate OS=Homo sapiens GN=MARCKS PE=1 SV=4 | MARCS | 32 | 0.53 | 12% | 100% | 100% | 99% | 95% | 95% | 100% | 100% | 100% | 100% | 100% | 95% |
| 717 | Four and a half LIM domains protein 1 OS=Homo sapiens GN=FHL1 PE=1 SV=4 | FHL1 | 36 | 0.011 | 100% | 100% | 100% | 95% | 99% | 100% | 100% | 100% | 100% | 95% | 0 | 0 |
| 718 | Catalase OS=Homo sapiens GN=CAT PE=1 SV=3 | CATA | 60 | 0.0015 | 100% | 100% | 100% | 0 | 100% | 99% | 0 | 0 | 0 | 100% | 100% | 100% |
| 719 | 60S ribosomal protein L38 OS=Homo sapiens GN=RPL38 PE=1 SV=2 | RL38 | 8 | 0.052 | 95% | 100% | 100% | 95% | 95% | 95% | 100% | 99% | 100% | 100% | 100% | 100% |
| 720 | Calpain-1 catalytic subunit OS=Homo sapiens GN=CAPN1 PE=1 SV=1 | CAN1 | 82 | 0.28 | 93% | 100% | 100% | 0 | 95% | 95% | 99% | 18% | 9% | 100% | 100% | 100% |
| 721 | LIM and SH3 domain protein 1 OS=Homo sapiens GN=LASP1 PE=1 SV=2 | LASP1 | 30 | 0.3 | 100% | 100% | 95% | 95% | 95% | 100% | 100% | 100% | 100% | 100% | 99% | 100% |
| 722 | V-type proton ATPase 116 kDa subunit a isoform 2 OS=Homo sapiens GN=ATP6V0A2 PE=1 SV=2 | VPP2 | 98 | 0.063 | 95% | 0 | 100% | 100% | 99% | 95% | 0 | 100% | 0 | 98% | 100% | 100% |
| 723 | Actin-related protein 2 OS=Homo sapiens GN=ACTR2 PE=1 SV=1 | ARP2 | 45 | 0.1 | 95% | 99% | 95% | 95% | 100% | 95% | 0 | 100% | 100% | 15% | 16% | 67% |
| 724 | 60S ribosomal protein L37a OS=Homo sapiens GN=RPL37A PE=1 SV=2 | RL37A | 10 | 0.071 | 100% | 100% | 95% | 23% | 95% | 0 | 100% | 95% | 100% | 100% | 100% | 95% |
| 725 | A-kinase anchor protein 12 OS=Homo sapiens GN=AKAP12 PE=1 SV=4 | AKA12 | 191 | 0.006 | 19% | 100% | 100% | 0 | 0 | 0 | 100% | 100% | 100% | 0 | 0 | 0 |
| 726 | Actin-related protein 2/3 complex subunit 1B OS=Homo sapiens GN=ARPC1B PE=1 SV=3 | ARC1B | 41 | 0.97 | 95% | 100% | 95% | 100% | 100% | 95% | 100% | 100% | 0 | 100% | 95% | 95% |
| 727 | 40S ribosomal protein S29 OS=Homo sapiens GN=RPS29 PE=1 SV=2 | RS29 | 7 | 0.26 | 43% | 63% | 41% | 0 | 61% | 95% | 45% | 95% | 29% | 81% | 100% | 100% |
| 728 | Cluster of Tensin-1 OS=Homo sapiens GN=TNS1 PE=1 SV=2 (TENS1_HUMAN) | TENS1 [3] | 186 | 0.0072 | 100% | 100% | 100% | 0 | 15% | 0 | 0 | 18% | 0 | 95% | 95% | 0 |
| 729 | Cluster of Adenylyl cyclase-associated protein 1 OS=Homo sapiens GN=CAP1 PE=1 SV=5 (CAP1_HUMAN) | CAP1 [2] | 52 | 0.036 | 0 | 100% | 95% | 0 | 0 | 41% | 100% | 100% | 100% | 95% | 100% | 95% |
| 730 | Actin-related protein 2/3 complex subunit 3 OS=Homo sapiens GN=ARPC3 PE=1 SV=3 | ARPC3 | 21 | 0.2 | 95% | 0 | 0 | 95% | 99% | 0 | 100% | 95% | 95% | 100% | 13% | 0 |
| 731 | Actin-related protein 2/3 complex subunit 5 OS=Homo sapiens GN=ARPC5 PE=1 SV=3 | ARPC5 | 16 | 0.099 | 0 | 0 | 100% | 95% | 15% | 54% | 100% | 97% | 95% | 0 | 0 | 0 |
| 732 | TRIO and F-actin-binding protein OS=Homo sapiens GN=TRIOBP PE=1 SV=3 | TARA | 261 | 0.44 | 0 | 6% | 0 | 0 | 100% | 0 | 79% | 72% | 0 | 8% | 0 | 0 |
| 733 | High mobility group protein HMG-I/HMG-Y OS=Homo sapiens GN=HMGA1 PE=1 SV=3 | HMGA1 | 12 | < 0.00010 | 98% | 95% | 95% | 0 | 0 | 0 | 0 | 0 | 0 | 95% | 95% | 95% |
| 734 | Protein phosphatase 1 regulatory subunit 12A OS=Homo sapiens GN=PPP1R12A PE=1 SV=1 | MYPT1 | 115 | 0.43 | 0 | 95% | 95% | 0 | 0 | 95% | 100% | 95% | 95% | 0 | 98% | 95% |
| 735 | Dedicator of cytokinesis protein 7 OS=Homo sapiens GN=DOCK7 PE=1 SV=4 | DOCK7 | 243 | 0.096 | 0 | 0 | 0 | 46% | 100% | 100% | 0 | 0 | 0 | 0 | 0 | 0 |
| 736 | Calponin-1 OS=Homo sapiens GN=CNN1 PE=1 SV=2 | CNN1 | 33 | 0.0048 | 0 | 0 | 0 | 89% | 100% | 95% | 0 | 95% | 100% | 0 | 0 | 0 |
| 737 | Cysteine and glycine-rich protein 1 OS=Homo sapiens GN=CSRP1 PE=1 SV=3 | CSRP1 | 21 | 0.54 | 0 | 100% | 0 | 0 | 95% | 0 | 95% | 95% | 95% | 0 | 95% | 0 |
| 738 | Protein flightless-1 homolog OS=Homo sapiens GN=FLII PE=1 SV=2 | FLII | 145 | 0.53 | 0 | 0 | 0 | 95% | 0 | 0 | 95% | 13% | 95% | 0 | 0 | 95% |
| 739 | Vinexin OS=Homo sapiens GN=SORBS3 PE=1 SV=2 | VINEX | 75 | 0.28 | 0 | 100% | 95% | 0 | 0 | 0 | 0 | 100% | 0 | 0 | 0 | 0 |
| 740 | Protein enabled homolog OS=Homo sapiens GN=ENAH PE=1 SV=2 | ENAH | 67 | 0.44 | 0 | 0 | 0 | 0 | 0 | 0 | 100% | 0 | 0 | 0 | 0 | 0 |
| 741 | Signal recognition particle subunit SRP68 OS=Homo sapiens GN=SRP68 PE=1 SV=2 | SRP68 | 71 | 0.44 | 0 | 99% | 0 | 0 | 0 | 0 | 0 | 0 | 22% | 0 | 95% | 0 |
| 742 | Protein AATF OS=Homo sapiens GN=AATF PE=1 SV=1 | AATF | 63 | 0.44 | 0 | 0 | 0 | 0 | 0 | 0 | 0 | 0 | 0 | 0 | 0 | 95% |
| 743 | Calcineurin B homologous protein 1 OS=Homo sapiens GN=CHP1 PE=1 SV=3 | CHP1 | 22 | 0.6 | 0 | 0 | 0 | 0 | 95% | 0 | 95% | 14% | 0 | 0 | 0 | 0 |
| 744 | Four and a half LIM domains protein 2 OS=Homo sapiens GN=FHL2 PE=1 SV=3 | FHL2 | 32 | 0.44 | 63% | 0 | 95% | 0 | 0 | 0 | 0 | 0 | 0 | 0 | 0 | 0 |
| 745 | Kinesin-like protein KIF23 OS=Homo sapiens GN=KIF23 PE=1 SV=3 | KIF23 | 110 | 0.44 | 0 | 0 | 0 | 0 | 95% | 0 | 0 | 0 | 0 | 0 | 0 | 0 |
| 746 | V-type proton ATPase 16 kDa proteolipid subunit OS=Homo sapiens GN=ATP6V0C PE=1 SV=1 | VATL | 16 | 0.44 | 0 | 0 | 0 | 0 | 0 | 0 | 0 | 0 | 0 | 95% | 0 | 0 |
| 747 | Cluster of Histone H2B type 1-C/E/F/G/I OS=Homo sapiens GN=HIST1H2BC PE=1 SV=4 (H2B1C_HUMAN) | H2B1C [13] | 14 | 0.1 | 100% | 100% | 100% | 100% | 100% | 100% | 100% | 100% | 100% | 100% | 100% | 100% |
| 748 | Protein S100-A10 OS=Homo sapiens GN=S100A10 PE=1 SV=2 | S10AA | 11 | < 0.00010 | 100% | 100% | 100% | 100% | 100% | 100% | 100% | 100% | 100% | 100% | 100% | 100% |
| 749 | Tyrosine-protein kinase JAK1 OS=Homo sapiens GN=JAK1 PE=1 SV=2 | JAK1 | 133 | 0.84 | 0 | 100% | 100% | 13% | 100% | 100% | 100% | 0 | 100% | 100% | 95% | 63% |
| 750 | Glia-derived nexin OS=Homo sapiens GN=SERPINE2 PE=1 SV=1 | GDN | 44 | 0.078 | 100% | 100% | 100% | 95% | 0 | 96% | 5% | 95% | 0 | 0 | 95% | 0 |
| 751 | Calreticulin OS=Homo sapiens GN=CALR PE=1 SV=1 | CALR | 48 | 0.092 | 100% | 100% | 100% | 100% | 100% | 100% | 100% | 100% | 100% | 100% | 100% | 100% |
| 752 | Endoglin OS=Homo sapiens GN=ENG PE=1 SV=2 | EGLN | 71 | 0.028 | 100% | 100% | 100% | 100% | 100% | 100% | 100% | 100% | 100% | 100% | 100% | 100% |
| 753 | Annexin A5 OS=Homo sapiens GN=ANXA5 PE=1 SV=2 | ANXA5 | 36 | < 0.00010 | 100% | 100% | 100% | 100% | 100% | 100% | 100% | 100% | 100% | 100% | 100% | 100% |
| 754 | Cluster of Thrombospondin-1 OS=Homo sapiens GN=THBS1 PE=1 SV=2 (TSP1_HUMAN) | TSP1 [2] | 129 | < 0.00010 | 100% | 100% | 100% | 100% | 100% | 100% | 100% | 100% | 100% | 100% | 100% | 100% |
| 755 | Endothelin-converting enzyme 1 OS=Homo sapiens GN=ECE1 PE=1 SV=2 | ECE1 | 87 | 0.14 | 100% | 100% | 100% | 100% | 100% | 100% | 100% | 100% | 100% | 100% | 100% | 100% |
| 756 | Lactadherin OS=Homo sapiens GN=MFGE8 PE=1 SV=2 | MFGM | 43 | 0.00049 | 100% | 100% | 100% | 96% | 100% | 100% | 100% | 100% | 100% | 100% | 100% | 100% |
| 757 | Cell surface glycoprotein MUC18 OS=Homo sapiens GN=MCAM PE=1 SV=2 | MUC18 | 72 | < 0.00010 | 75% | 100% | 100% | 100% | 100% | 100% | 43% | 0 | 94% | 100% | 100% | 100% |
| 758 | Cleft lip and palate transmembrane protein 1 OS=Homo sapiens GN=CLPTM1 PE=1 SV=1 | CLPT1 | 76 | 0.051 | 100% | 100% | 100% | 100% | 100% | 100% | 100% | 100% | 100% | 100% | 100% | 100% |
| 759 | CD276 antigen OS=Homo sapiens GN=CD276 PE=1 SV=1 | CD276 | 57 | 0.16 | 100% | 100% | 100% | 100% | 100% | 100% | 100% | 100% | 100% | 100% | 100% | 100% |
| 760 | Renin receptor OS=Homo sapiens GN=ATP6AP2 PE=1 SV=2 | RENR | 39 | 0.25 | 87% | 95% | 100% | 100% | 100% | 100% | 100% | 11% | 100% | 100% | 100% | 100% |
| 761 | Vascular cell adhesion protein 1 OS=Homo sapiens GN=VCAM1 PE=1 SV=1 | VCAM1 | 81 | 0.065 | 100% | 95% | 100% | 0 | 0 | 0 | 0 | 95% | 100% | 0 | 0 | 95% |
| 762 | Ubiquitin-like modifier-activating enzyme 1 OS=Homo sapiens GN=UBA1 PE=1 SV=3 | UBA1 | 118 | 0.068 | 100% | 100% | 100% | 100% | 100% | 100% | 100% | 100% | 100% | 100% | 100% | 100% |
| 763 | Desmoglein-3 OS=Homo sapiens GN=DSG3 PE=1 SV=2 | DSG3 | 108 | 0.00034 | 100% | 100% | 100% | 0 | 0 | 0 | 0 | 0 | 0 | 8% | 0 | 0 |
| 764 | Low-density lipoprotein receptor-related protein 10 OS=Homo sapiens GN=LRP10 PE=1 SV=2 | LRP10 | 76 | 0.5 | 100% | 100% | 100% | 100% | 100% | 100% | 95% | 100% | 81% | 0 | 100% | 100% |
| 765 | SH3 domain-binding protein 4 OS=Homo sapiens GN=SH3BP4 PE=1 SV=1 | SH3B4 | 107 | 0.8 | 0 | 95% | 0 | 0 | 0 | 95% | 0 | 0 | 95% | 0 | 0 | 0 |
| 766 | Filamin-A OS=Homo sapiens GN=FLNA PE=1 SV=4 | FLNA | 281 | 0.0002 | 100% | 100% | 100% | 100% | 100% | 100% | 100% | 100% | 100% | 100% | 100% | 100% |
| 767 | Ras-related protein Rab-10 OS=Homo sapiens GN=RAB10 PE=1 SV=1 | RAB10 | 23 | 0.16 | 100% | 100% | 100% | 100% | 100% | 100% | 100% | 100% | 100% | 100% | 100% | 100% |
| 768 | Cluster of Actin-related protein 3 OS=Homo sapiens GN=ACTR3 PE=1 SV=3 (ARP3_HUMAN) | ARP3 [3] | 47 | 0.25 | 100% | 100% | 100% | 100% | 100% | 100% | 100% | 100% | 100% | 100% | 100% | 100% |
| 769 | Cluster of Serine/threonine-protein phosphatase PP1-alpha catalytic subunit OS=Homo sapiens GN=PPP1CA PE=1 SV=1 (PP1A_HUMAN) | PP1A [2] | 38 | 0.9 | 100% | 100% | 100% | 100% | 100% | 100% | 100% | 100% | 100% | 100% | 100% | 100% |
| 770 | Pinin OS=Homo sapiens GN=PNN PE=1 SV=4 | PININ | 82 | 0.0011 | 14% | 100% | 100% | 100% | 100% | 100% | 100% | 0 | 100% | 100% | 100% | 100% |
| 771 | WD repeat-containing protein 1 OS=Homo sapiens GN=WDR1 PE=1 SV=4 | WDR1 | 66 | 0.33 | 100% | 100% | 100% | 100% | 100% | 100% | 100% | 100% | 100% | 100% | 100% | 100% |
| 772 | PDZ and LIM domain protein 7 OS=Homo sapiens GN=PDLIM7 PE=1 SV=1 | PDLI7 | 50 | 0.023 | 95% | 100% | 100% | 100% | 98% | 95% | 100% | 100% | 100% | 100% | 95% | 100% |
| 773 | Cluster of Protocadherin gamma-A3 OS=Homo sapiens GN=PCDHGA3 PE=2 SV=2 (PCDG3_HUMAN) | PCDG3 [8] | 101 | 0.65 | 6% | 100% | 100% | 0 | 95% | 0 | 6% | 100% | 100% | 0 | 95% | 100% |
| 774 | Serine/threonine-protein kinase MRCK alpha OS=Homo sapiens GN=CDC42BPA PE=1 SV=1 | MRCKA | 197 | 0.0071 | 100% | 0 | 7% | 99% | 100% | 100% | 100% | 99% | 98% | 0 | 13% | 0 |
| 775 | Tropomodulin-3 OS=Homo sapiens GN=TMOD3 PE=1 SV=1 | TMOD3 | 40 | 0.1 | 0 | 95% | 95% | 95% | 100% | 100% | 0 | 28% | 100% | 100% | 100% | 100% |
| 776 | Gamma-adducin OS=Homo sapiens GN=ADD3 PE=1 SV=1 | ADDG | 79 | 0.024 | 100% | 100% | 100% | 0 | 0 | 95% | 0 | 0 | 0 | 100% | 100% | 100% |
| 777 | Serologically defined colon cancer antigen 8 OS=Homo sapiens GN=SDCCAG8 PE=1 SV=1 | SDCG8 | 83 | 0.44 | 0 | 96% | 84% | 0 | 0 | 0 | 0 | 9% | 81% | 0 | 0 | 0 |
| 778 | Fascin OS=Homo sapiens GN=FSCN1 PE=1 SV=3 | FSCN1 | 55 | 0.12 | 95% | 31% | 100% | 15% | 0 | 0 | 95% | 95% | 95% | 0 | 0 | 95% |
| 779 | Reticulon-4 OS=Homo sapiens GN=RTN4 PE=1 SV=2 | RTN4 | 130 | 0.014 | 100% | 100% | 100% | 100% | 100% | 100% | 100% | 100% | 100% | 100% | 100% | 100% |
| 780 | Cluster of Ras-related protein Rab-1B OS=Homo sapiens GN=RAB1B PE=1 SV=1 (RAB1B_HUMAN) | RAB1B [2] | 22 | 0.001 | 100% | 100% | 100% | 100% | 100% | 100% | 100% | 100% | 100% | 100% | 100% | 100% |
| 781 | Cluster of Fructose-bisphosphate aldolase A OS=Homo sapiens GN=ALDOA PE=1 SV=2 (ALDOA_HUMAN) | ALDOA [3] | 39 | 0.00031 | 100% | 100% | 100% | 100% | 100% | 100% | 100% | 100% | 100% | 100% | 100% | 100% |
| 782 | Spectrin alpha chain, non-erythrocytic 1 OS=Homo sapiens GN=SPTAN1 PE=1 SV=3 | SPTN1 | 285 | 0.0022 | 100% | 100% | 100% | 100% | 100% | 100% | 100% | 100% | 100% | 100% | 100% | 100% |
| 783 | 60S ribosomal protein L6 OS=Homo sapiens GN=RPL6 PE=1 SV=3 | RL6 | 33 | 0.19 | 100% | 100% | 100% | 100% | 100% | 100% | 100% | 100% | 100% | 100% | 100% | 100% |
| 784 | 60S ribosomal protein L7a OS=Homo sapiens GN=RPL7A PE=1 SV=2 | RL7A | 30 | 0.0097 | 100% | 100% | 100% | 100% | 100% | 100% | 100% | 100% | 100% | 100% | 100% | 100% |
| 785 | Elongation factor 2 OS=Homo sapiens GN=EEF2 PE=1 SV=4 | EF2 | 95 | 0.00032 | 100% | 100% | 100% | 100% | 100% | 100% | 100% | 100% | 100% | 100% | 100% | 100% |
| 786 | Leucine-rich repeat-containing protein 59 OS=Homo sapiens GN=LRRC59 PE=1 SV=1 | LRC59 | 35 | 0.0068 | 100% | 100% | 100% | 100% | 100% | 100% | 100% | 100% | 100% | 100% | 100% | 100% |
| 787 | Caldesmon OS=Homo sapiens GN=CALD1 PE=1 SV=3 | CALD1 | 93 | 0.0029 | 100% | 100% | 100% | 100% | 100% | 100% | 100% | 100% | 100% | 100% | 100% | 100% |
| 788 | Cluster of Histone H3.2 OS=Homo sapiens GN=HIST2H3A PE=1 SV=3 (H32_HUMAN) | H32 [3] | 15 | 0.43 | 100% | 100% | 100% | 100% | 100% | 100% | 100% | 100% | 100% | 100% | 100% | 100% |
| 789 | Staphylococcal nuclease domain-containing protein 1 OS=Homo sapiens GN=SND1 PE=1 SV=1 | SND1 | 102 | 0.26 | 100% | 100% | 100% | 100% | 100% | 100% | 100% | 100% | 100% | 100% | 100% | 100% |
| 790 | Golgin subfamily A member 2 OS=Homo sapiens GN=GOLGA2 PE=1 SV=3 | GOGA2 | 113 | 0.059 | 100% | 100% | 100% | 100% | 100% | 100% | 100% | 100% | 100% | 100% | 100% | 100% |
| 791 | Cluster of ATP-dependent RNA helicase DDX3X OS=Homo sapiens GN=DDX3X PE=1 SV=3 (DDX3X_HUMAN) | DDX3X [2] | 73 | 0.021 | 100% | 100% | 100% | 100% | 100% | 100% | 100% | 100% | 100% | 100% | 100% | 100% |
| 792 | 40S ribosomal protein S2 OS=Homo sapiens GN=RPS2 PE=1 SV=2 | RS2 | 31 | 0.048 | 100% | 100% | 100% | 100% | 100% | 100% | 100% | 100% | 100% | 100% | 100% | 100% |
| 793 | 60S ribosomal protein L14 OS=Homo sapiens GN=RPL14 PE=1 SV=4 | RL14 | 23 | 0.0062 | 100% | 100% | 100% | 100% | 100% | 100% | 100% | 100% | 100% | 100% | 100% | 100% |
| 794 | 60S ribosomal protein L15 OS=Homo sapiens GN=RPL15 PE=1 SV=2 | RL15 | 24 | 0.0021 | 100% | 100% | 100% | 100% | 100% | 100% | 100% | 100% | 100% | 100% | 100% | 100% |
| 795 | 60S ribosomal protein L23a OS=Homo sapiens GN=RPL23A PE=1 SV=1 | RL23A | 18 | 0.16 | 100% | 100% | 100% | 100% | 100% | 100% | 100% | 100% | 100% | 100% | 100% | 100% |
| 796 | 60S ribosomal protein L24 OS=Homo sapiens GN=RPL24 PE=1 SV=1 | RL24 | 18 | 0.082 | 100% | 100% | 100% | 100% | 100% | 100% | 100% | 100% | 100% | 100% | 100% | 100% |
| 797 | Cluster of Poly(rC)-binding protein 1 OS=Homo sapiens GN=PCBP1 PE=1 SV=2 (PCBP1_HUMAN) | PCBP1 [3] | 37 | 0.25 | 100% | 100% | 100% | 100% | 100% | 100% | 100% | 100% | 100% | 100% | 100% | 100% |
| 798 | T-complex protein 1 subunit theta OS=Homo sapiens GN=CCT8 PE=1 SV=4 | TCPQ | 60 | 0.56 | 100% | 100% | 100% | 100% | 100% | 100% | 100% | 100% | 100% | 100% | 100% | 100% |
| 799 | Ribosomal L1 domain-containing protein 1 OS=Homo sapiens GN=RSL1D1 PE=1 SV=3 | RL1D1 | 55 | 0.13 | 100% | 100% | 100% | 100% | 100% | 100% | 100% | 100% | 100% | 100% | 100% | 100% |
| 800 | Nucleolar protein 56 OS=Homo sapiens GN=NOP56 PE=1 SV=4 | NOP56 | 66 | 0.24 | 100% | 100% | 100% | 100% | 100% | 100% | 100% | 100% | 100% | 100% | 100% | 100% |
| 801 | Vesicle-associated membrane protein-associated protein B/C OS=Homo sapiens GN=VAPB PE=1 SV=3 | VAPB | 27 | 0.096 | 100% | 100% | 100% | 100% | 100% | 100% | 100% | 100% | 100% | 100% | 100% | 100% |
| 802 | Elongation factor 1-gamma OS=Homo sapiens GN=EEF1G PE=1 SV=3 | EF1G | 50 | 0.0088 | 100% | 100% | 100% | 100% | 100% | 100% | 100% | 100% | 100% | 100% | 100% | 100% |
| 803 | 60S ribosomal protein L29 OS=Homo sapiens GN=RPL29 PE=1 SV=2 | RL29 | 18 | 0.37 | 100% | 100% | 100% | 100% | 100% | 100% | 100% | 100% | 100% | 100% | 100% | 100% |
| 804 | Protein S100-A11 OS=Homo sapiens GN=S100A11 PE=1 SV=2 | S10AB | 12 | 0.26 | 100% | 100% | 100% | 100% | 100% | 100% | 100% | 100% | 100% | 100% | 100% | 100% |
| 805 | Eukaryotic translation initiation factor 2 subunit 3 OS=Homo sapiens GN=EIF2S3 PE=1 SV=3 | IF2G | 51 | 0.00094 | 100% | 100% | 100% | 100% | 100% | 100% | 100% | 100% | 100% | 100% | 100% | 100% |
| 806 | Vigilin OS=Homo sapiens GN=HDLBP PE=1 SV=2 | VIGLN | 141 | 0.3 | 95% | 100% | 100% | 100% | 95% | 100% | 100% | 100% | 100% | 100% | 100% | 100% |
| 807 | Septin-9 OS=Homo sapiens GN=SEPT9 PE=1 SV=2 | SEPT9 | 65 | 0.12 | 100% | 100% | 100% | 100% | 100% | 100% | 100% | 100% | 100% | 100% | 100% | 100% |
| 808 | Cluster of Kinesin-1 heavy chain OS=Homo sapiens GN=KIF5B PE=1 SV=1 (KINH_HUMAN) | KINH [3] | 110 | 0.024 | 0 | 100% | 100% | 89% | 100% | 100% | 100% | 100% | 100% | 100% | 100% | 100% |
| 809 | Zinc finger CCCH-type antiviral protein 1 OS=Homo sapiens GN=ZC3HAV1 PE=1 SV=3 | ZCCHV | 101 | 0.0075 | 100% | 100% | 100% | 100% | 100% | 100% | 100% | 100% | 100% | 100% | 100% | 100% |
| 810 | Keratin, type I cytoskeletal 18 OS=Homo sapiens GN=KRT18 PE=1 SV=2 | K1C18 | 48 | 0.00075 | 0 | 0 | 64% | 100% | 100% | 100% | 0 | 100% | 100% | 100% | 100% | 100% |
| 811 | Elongation factor 1-delta OS=Homo sapiens GN=EEF1D PE=1 SV=5 | EF1D | 31 | < 0.00010 | 100% | 100% | 95% | 100% | 100% | 100% | 100% | 100% | 100% | 100% | 100% | 100% |
| 812 | 40S ribosomal protein S26 OS=Homo sapiens GN=RPS26 PE=1 SV=3 | RS26 | 13 | 0.82 | 100% | 100% | 100% | 100% | 100% | 100% | 100% | 100% | 100% | 100% | 100% | 100% |
| 813 | Histone H1x OS=Homo sapiens GN=H1FX PE=1 SV=1 | H1X | 22 | 0.081 | 100% | 100% | 100% | 98% | 100% | 100% | 100% | 100% | 100% | 100% | 100% | 100% |
| 814 | F-actin-capping protein subunit beta OS=Homo sapiens GN=CAPZB PE=1 SV=4 | CAPZB | 31 | 0.51 | 100% | 100% | 100% | 100% | 100% | 100% | 100% | 100% | 100% | 100% | 95% | 100% |
| 815 | Profilin-1 OS=Homo sapiens GN=PFN1 PE=1 SV=2 | PROF1 | 15 | 0.0014 | 100% | 100% | 100% | 100% | 100% | 100% | 100% | 100% | 100% | 100% | 100% | 100% |
| 816 | Perilipin-3 OS=Homo sapiens GN=PLIN3 PE=1 SV=3 | PLIN3 | 47 | 0.018 | 100% | 100% | 100% | 100% | 0 | 100% | 100% | 95% | 100% | 100% | 100% | 100% |
| 817 | RuvB-like 1 OS=Homo sapiens GN=RUVBL1 PE=1 SV=1 | RUVB1 | 50 | 0.14 | 90% | 100% | 100% | 100% | 100% | 100% | 100% | 100% | 100% | 100% | 100% | 100% |
| 818 | 60S ribosomal protein L34 OS=Homo sapiens GN=RPL34 PE=1 SV=3 | RL34 | 13 | 0.022 | 95% | 100% | 100% | 100% | 46% | 100% | 100% | 100% | 100% | 100% | 100% | 100% |
| 819 | Tyrosine-protein phosphatase non-receptor type 1 OS=Homo sapiens GN=PTPN1 PE=1 SV=1 | PTN1 | 50 | 0.47 | 100% | 100% | 100% | 100% | 100% | 100% | 100% | 100% | 100% | 100% | 100% | 100% |
| 820 | Emerin OS=Homo sapiens GN=EMD PE=1 SV=1 | EMD | 29 | 0.93 | 95% | 100% | 100% | 100% | 100% | 93% | 100% | 95% | 100% | 100% | 100% | 100% |
| 821 | Signal transducer and activator of transcription 1-alpha/beta OS=Homo sapiens GN=STAT1 PE=1 SV=2 | STAT1 | 87 | 0.89 | 100% | 100% | 100% | 100% | 100% | 100% | 100% | 100% | 95% | 100% | 100% | 99% |
| 822 | Poly(U)-binding-splicing factor PUF60 OS=Homo sapiens GN=PUF60 PE=1 SV=1 | PUF60 | 60 | 0.041 | 95% | 100% | 100% | 95% | 100% | 100% | 100% | 100% | 100% | 100% | 100% | 100% |
| 823 | Syntaxin-5 OS=Homo sapiens GN=STX5 PE=1 SV=2 | STX5 | 40 | 0.72 | 100% | 100% | 100% | 100% | 100% | 100% | 100% | 100% | 95% | 100% | 100% | 95% |
| 824 | Eukaryotic translation initiation factor 4 gamma 1 OS=Homo sapiens GN=EIF4G1 PE=1 SV=4 | IF4G1 | 175 | 0.076 | 73% | 0 | 100% | 100% | 97% | 100% | 100% | 100% | 100% | 100% | 100% | 100% |
| 825 | Eukaryotic translation initiation factor 3 subunit E OS=Homo sapiens GN=EIF3E PE=1 SV=1 | EIF3E | 52 | 0.67 | 95% | 100% | 100% | 100% | 15% | 71% | 99% | 100% | 100% | 100% | 100% | 100% |
| 826 | Transgelin-2 OS=Homo sapiens GN=TAGLN2 PE=1 SV=3 | TAGL2 | 22 | 0.12 | 100% | 95% | 100% | 0 | 100% | 0 | 99% | 100% | 100% | 100% | 100% | 100% |
| 827 | Transmembrane protein 2 OS=Homo sapiens GN=TMEM2 PE=1 SV=1 | TMEM2 | 154 | 0.39 | 0 | 95% | 0 | 0 | 100% | 100% | 95% | 28% | 95% | 100% | 100% | 97% |
| 828 | Rho GTPase-activating protein 1 OS=Homo sapiens GN=ARHGAP1 PE=1 SV=1 | RHG01 | 50 | 0.013 | 95% | 100% | 100% | 95% | 95% | 0 | 100% | 100% | 100% | 100% | 100% | 95% |
| 829 | Endophilin-B1 OS=Homo sapiens GN=SH3GLB1 PE=1 SV=1 | SHLB1 | 41 | 0.1 | 99% | 100% | 100% | 98% | 100% | 97% | 95% | 95% | 0 | 100% | 100% | 100% |
| 830 | F-actin-capping protein subunit alpha-1 OS=Homo sapiens GN=CAPZA1 PE=1 SV=3 | CAZA1 | 33 | 0.9 | 100% | 100% | 100% | 100% | 100% | 100% | 100% | 100% | 6% | 6% | 100% | 6% |
| 831 | Cluster of Calponin-2 OS=Homo sapiens GN=CNN2 PE=1 SV=4 (CNN2_HUMAN) | CNN2 [2] | 34 | 0.52 | 0 | 11% | 95% | 95% | 0 | 100% | 95% | 100% | 95% | 0 | 100% | 100% |
| 832 | Probable ATP-dependent RNA helicase DDX6 OS=Homo sapiens GN=DDX6 PE=1 SV=2 | DDX6 | 54 | 0.38 | 0 | 16% | 98% | 0 | 100% | 69% | 100% | 46% | 100% | 0 | 84% | 0 |
| 833 | MAGUK p55 subfamily member 7 OS=Homo sapiens GN=MPP7 PE=1 SV=1 | MPP7 | 66 | 0.0027 | 0 | 0 | 0 | 100% | 100% | 100% | 0 | 0 | 0 | 0 | 0 | 100% |
| 834 | IST1 homolog OS=Homo sapiens GN=IST1 PE=1 SV=1 | IST1 | 40 | 0.17 | 0 | 0 | 81% | 15% | 0 | 14% | 85% | 0 | 91% | 100% | 81% | 100% |
| 835 | Arginine--tRNA ligase, cytoplasmic OS=Homo sapiens GN=RARS PE=1 SV=2 | SYRC | 75 | 0.47 | 0 | 100% | 0 | 100% | 100% | 98% | 100% | 0 | 95% | 0 | 0 | 5% |
| 836 | Fatty acid synthase OS=Homo sapiens GN=FASN PE=1 SV=3 | FAS | 273 | 0.34 | 0 | 0 | 6% | 0 | 0 | 0 | 100% | 64% | 0 | 100% | 0 | 100% |
| 837 | Clathrin interactor 1 OS=Homo sapiens GN=CLINT1 PE=1 SV=1 | EPN4 | 68 | < 0.00010 | 0 | 0 | 98% | 0 | 0 | 0 | 0 | 0 | 0 | 100% | 100% | 100% |
| 838 | Peroxiredoxin-6 OS=Homo sapiens GN=PRDX6 PE=1 SV=3 | PRDX6 | 25 | 0.072 | 95% | 95% | 100% | 0 | 0 | 0 | 0 | 100% | 68% | 95% | 95% | 95% |
| 839 | Niban-like protein 1 OS=Homo sapiens GN=FAM129B PE=1 SV=3 | NIBL1 | 84 | 0.87 | 95% | 0 | 95% | 0 | 0 | 95% | 42% | 0 | 76% | 95% | 95% | 100% |
| 840 | Coronin-1B OS=Homo sapiens GN=CORO1B PE=1 SV=1 | COR1B | 54 | 0.89 | 95% | 95% | 15% | 0 | 95% | 14% | 95% | 0 | 0 | 0 | 95% | 8% |
| 841 | GTP-binding nuclear protein Ran OS=Homo sapiens GN=RAN PE=1 SV=3 | RAN | 24 | 0.12 | 0 | 100% | 100% | 0 | 0 | 0 | 0 | 0 | 0 | 25% | 100% | 56% |
| 842 | E3 ubiquitin/ISG15 ligase TRIM25 OS=Homo sapiens GN=TRIM25 PE=1 SV=2 | TRI25 | 71 | 0.59 | 0 | 63% | 95% | 0 | 0 | 0 | 27% | 0 | 6% | 65% | 100% | 0 |
| 843 | Exocyst complex component 3 OS=Homo sapiens GN=EXOC3 PE=1 SV=2 | EXOC3 | 87 | 0.44 | 0 | 13% | 0 | 62% | 0 | 100% | 0 | 0 | 0 | 10% | 6% | 0 |
| 844 | Multifunctional protein ADE2 OS=Homo sapiens GN=PAICS PE=1 SV=3 | PUR6 | 47 | 0.58 | 0 | 0 | 100% | 0 | 0 | 0 | 0 | 9% | 95% | 0 | 0 | 0 |
| 845 | Afadin OS=Homo sapiens GN=MLLT4 PE=1 SV=3 | AFAD | 207 | 0.44 | 6% | 0 | 0 | 0 | 0 | 0 | 0 | 100% | 0 | 0 | 0 | 0 |
| 846 | General vesicular transport factor p115 OS=Homo sapiens GN=USO1 PE=1 SV=2 | USO1 | 108 | 0.44 | 18% | 0 | 0 | 0 | 0 | 53% | 0 | 0 | 0 | 0 | 100% | 0 |
| 847 | Cytoskeleton-associated protein 5 OS=Homo sapiens GN=CKAP5 PE=1 SV=3 | CKAP5 | 226 | 0.24 | 0 | 0 | 0 | 0 | 0 | 95% | 0 | 0 | 0 | 95% | 0 | 99% |
| 848 | Cluster of La-related protein 1 OS=Homo sapiens GN=LARP1 PE=1 SV=2 (LARP1_HUMAN) | LARP1 [2] | 124 | 0.44 | 0 | 0 | 0 | 0 | 0 | 0 | 0 | 0 | 0 | 93% | 0 | 0 |
| 849 | Wiskott-Aldrich syndrome protein family member 2 OS=Homo sapiens GN=WASF2 PE=1 SV=3 | WASF2 | 54 | 0.7 | 0 | 0 | 95% | 0 | 100% | 0 | 0 | 0 | 0 | 0 | 95% | 0 |
| 850 | Proteasome subunit beta type-6 OS=Homo sapiens GN=PSMB6 PE=1 SV=4 | PSB6 | 25 | 0.44 | 0 | 10% | 0 | 0 | 0 | 0 | 95% | 86% | 0 | 0 | 0 | 0 |
| 851 | Zinc finger CCCH domain-containing protein 15 OS=Homo sapiens GN=ZC3H15 PE=1 SV=1 | ZC3HF | 49 | 0.44 | 0 | 0 | 64% | 0 | 95% | 0 | 0 | 0 | 0 | 12% | 0 | 0 |
| 852 | Basic leucine zipper and W2 domain-containing protein 1 OS=Homo sapiens GN=BZW1 PE=1 SV=1 | BZW1 | 48 | 0.59 | 0 | 0 | 95% | 0 | 0 | 0 | 0 | 95% | 0 | 0 | 0 | 0 |
| 853 | Cdc42 effector protein 1 OS=Homo sapiens GN=CDC42EP1 PE=1 SV=1 | BORG5 | 40 | 0.44 | 0 | 0 | 0 | 0 | 0 | 0 | 0 | 0 | 0 | 95% | 0 | 0 |
| 854 | Basic leucine zipper and W2 domain-containing protein 2 OS=Homo sapiens GN=BZW2 PE=1 SV=1 | BZW2 | 48 | 0.44 | 0 | 0 | 0 | 0 | 0 | 0 | 95% | 0 | 0 | 0 | 0 | 0 |
| 855 | Heterogeneous nuclear ribonucleoprotein K OS=Homo sapiens GN=HNRNPK PE=1 SV=1 | HNRPK | 51 | 0.029 | 100% | 100% | 100% | 100% | 100% | 100% | 100% | 100% | 100% | 100% | 100% | 100% |
| 856 | Band 4.1-like protein 2 OS=Homo sapiens GN=EPB41L2 PE=1 SV=1 | E41L2 | 113 | 0.0054 | 100% | 100% | 100% | 100% | 100% | 100% | 100% | 98% | 100% | 100% | 100% | 100% |
| 857 | Beta-2-syntrophin OS=Homo sapiens GN=SNTB2 PE=1 SV=1 | SNTB2 | 58 | 0.058 | 100% | 100% | 100% | 100% | 100% | 100% | 100% | 100% | 100% | 100% | 100% | 100% |
| 858 | Cluster of Cytoplasmic FMR1-interacting protein 1 OS=Homo sapiens GN=CYFIP1 PE=1 SV=1 (CYFP1_HUMAN) | CYFP1 [2] | 145 | 0.076 | 100% | 100% | 100% | 100% | 100% | 100% | 100% | 100% | 100% | 100% | 100% | 100% |
| 859 | Secretory carrier-associated membrane protein 1 OS=Homo sapiens GN=SCAMP1 PE=1 SV=2 | SCAM1 | 38 | 0.017 | 100% | 100% | 100% | 100% | 100% | 100% | 100% | 100% | 100% | 100% | 100% | 100% |
| 860 | Pre-mRNA-splicing factor SPF27 OS=Homo sapiens GN=BCAS2 PE=1 SV=1 | SPF27 | 26 | 0.016 | 95% | 0 | 89% | 0 | 0 | 95% | 95% | 98% | 95% | 100% | 100% | 100% |
| 861 | Torsin-1A OS=Homo sapiens GN=TOR1A PE=1 SV=1 | TOR1A | 38 | 0.65 | 100% | 0 | 100% | 95% | 100% | 95% | 99% | 100% | 100% | 100% | 0 | 0 |
| 862 | Prenylated Rab acceptor protein 1 OS=Homo sapiens GN=RABAC1 PE=1 SV=1 | PRAF1 | 21 | 0.81 | 0 | 99% | 34% | 33% | 100% | 60% | 100% | 0 | 100% | 97% | 0 | 0 |
| 863 | Spectrin beta chain, non-erythrocytic 2 OS=Homo sapiens GN=SPTBN2 PE=1 SV=3 | SPTN2 | 271 | 0.72 | 0 | 0 | 96% | 0 | 46% | 0 | 72% | 0 | 95% | 0 | 100% | 0 |
| 864 | Hemicentin-2 OS=Homo sapiens GN=HMCN2 PE=2 SV=2 | HMCN2 | 543 | 0.44 | 0 | 0 | 83% | 0 | 0 | 0 | 0 | 0 | 0 | 8% | 8% | 95% |
| 865 | Leucine-rich repeat-containing protein 4B OS=Homo sapiens GN=LRRC4B PE=2 SV=3 | LRC4B | 76 | 0.2 | 0 | 0 | 0 | 39% | 95% | 95% | 0 | 0 | 0 | 95% | 15% | 53% |
| 866 | Transmembrane and ubiquitin-like domain-containing protein 1 OS=Homo sapiens GN=TMUB1 PE=1 SV=1 | TMUB1 | 26 | < 0.00010 | 0 | 0 | 0 | 100% | 100% | 95% | 0 | 0 | 0 | 0 | 15% | 0 |
| 867 | CD99 antigen-like protein 2 OS=Homo sapiens GN=CD99L2 PE=1 SV=1 | C99L2 | 28 | 0.0016 | 0 | 95% | 95% | 95% | 95% | 95% | 0 | 0 | 0 | 0 | 0 | 0 |
| 868 | Mitochondrial fission factor OS=Homo sapiens GN=MFF PE=1 SV=1 | MFF | 38 | 0.052 | 0 | 0 | 0 | 0 | 0 | 0 | 0 | 0 | 0 | 95% | 95% | 0 |
| 869 | Cluster of SH3 and PX domain-containing protein 2A OS=Homo sapiens GN=SH3PXD2A PE=1 SV=1 (SPD2A_HUMAN) | SPD2A [2] | 125 | 0.44 | 0 | 0 | 0 | 0 | 0 | 0 | 100% | 0 | 0 | 0 | 0 | 0 |
| 870 | Structure-specific endonuclease subunit SLX4 OS=Homo sapiens GN=SLX4 PE=1 SV=3 | SLX4 | 200 | 0.44 | 0 | 0 | 0 | 0 | 0 | 0 | 0 | 99% | 0 | 0 | 0 | 14% |
| 871 | Glycylpeptide N-tetradecanoyltransferase 1 OS=Homo sapiens GN=NMT1 PE=1 SV=2 | NMT1 | 57 | 0.44 | 0 | 0 | 0 | 0 | 95% | 0 | 0 | 0 | 0 | 0 | 0 | 0 |
| 872 | Transmembrane protein 230 OS=Homo sapiens GN=TMEM230 PE=1 SV=1 | TM230 | 13 | 0.44 | 0 | 0 | 95% | 0 | 0 | 0 | 0 | 0 | 0 | 0 | 0 | 0 |
| 873 | Prostacyclin synthase OS=Homo sapiens GN=PTGIS PE=1 SV=1 | PTGIS | 57 | < 0.00010 | 100% | 100% | 100% | 100% | 100% | 100% | 100% | 100% | 100% | 100% | 100% | 100% |
| 874 | Protein kinase C delta-binding protein OS=Homo sapiens GN=PRKCDBP PE=1 SV=3 | PRDBP | 28 | 0.0045 | 100% | 100% | 100% | 95% | 100% | 95% | 100% | 100% | 100% | 100% | 100% | 100% |
| 875 | Serum deprivation-response protein OS=Homo sapiens GN=SDPR PE=1 SV=3 | SDPR | 47 | 0.0079 | 0 | 0 | 0 | 100% | 100% | 100% | 0 | 0 | 74% | 100% | 0 | 100% |
| 876 | Battenin OS=Homo sapiens GN=CLN3 PE=1 SV=1 | CLN3 | 48 | 0.0012 | 0 | 0 | 0 | 0 | 0 | 0 | 95% | 100% | 95% | 0 | 0 | 0 |
| 877 | Cytochrome b reductase 1 OS=Homo sapiens GN=CYBRD1 PE=1 SV=1 | CYBR1 | 32 | 0.15 | 100% | 100% | 100% | 100% | 100% | 100% | 100% | 100% | 100% | 95% | 95% | 95% |
| 878 | Major facilitator superfamily domain-containing protein 10 OS=Homo sapiens GN=MFSD10 PE=2 SV=1 | MFS10 | 48 | 0.14 | 95% | 95% | 75% | 95% | 0 | 95% | 100% | 0 | 95% | 0 | 0 | 0 |
| 879 | Unconventional myosin-Id OS=Homo sapiens GN=MYO1D PE=1 SV=2 | MYO1D | 116 | < 0.00010 | 100% | 100% | 100% | 0 | 100% | 95% | 100% | 100% | 100% | 0 | 32% | 0 |
| 880 | Fibronectin OS=Homo sapiens GN=FN1 PE=1 SV=4 | FINC | 263 | < 0.00010 | 100% | 100% | 100% | 100% | 100% | 100% | 100% | 100% | 100% | 100% | 100% | 100% |
| 881 | V-type proton ATPase subunit d 1 OS=Homo sapiens GN=ATP6V0D1 PE=1 SV=1 | VA0D1 | 40 | 0.00028 | 100% | 100% | 100% | 100% | 100% | 100% | 100% | 100% | 100% | 100% | 100% | 100% |
| 882 | V-type proton ATPase subunit E 1 OS=Homo sapiens GN=ATP6V1E1 PE=1 SV=1 | VATE1 | 26 | 0.65 | 100% | 100% | 100% | 100% | 100% | 100% | 100% | 100% | 100% | 100% | 100% | 100% |
| 883 | Cluster of Cell cycle control protein 50A OS=Homo sapiens GN=TMEM30A PE=1 SV=1 (CC50A_HUMAN) | CC50A [2] | 41 | 0.05 | 100% | 100% | 100% | 100% | 100% | 100% | 100% | 100% | 100% | 100% | 100% | 100% |
| 884 | 5'-AMP-activated protein kinase catalytic subunit alpha-1 OS=Homo sapiens GN=PRKAA1 PE=1 SV=4 | AAPK1 | 64 | 0.091 | 0 | 0 | 95% | 0 | 0 | 95% | 95% | 99% | 95% | 95% | 95% | 80% |
| 885 | Ras-related protein Rab-27A OS=Homo sapiens GN=RAB27A PE=1 SV=3 | RB27A | 25 | 0.02 | 75% | 83% | 0 | 99% | 0 | 0 | 99% | 98% | 99% | 0 | 0 | 0 |
| 886 | FERM domain-containing protein 6 OS=Homo sapiens GN=FRMD6 PE=1 SV=1 | FRMD6 | 72 | 0.44 | 0 | 19% | 0 | 0 | 0 | 0 | 0 | 0 | 0 | 15% | 0 | 95% |
| 887 | CD59 glycoprotein OS=Homo sapiens GN=CD59 PE=1 SV=1 | CD59 | 14 | 0.0038 | 100% | 100% | 100% | 100% | 100% | 100% | 100% | 100% | 100% | 100% | 100% | 100% |
| 888 | Gamma-glutamyltransferase 7 OS=Homo sapiens GN=GGT7 PE=1 SV=2 | GGT7 | 70 | 0.052 | 0 | 0 | 0 | 0 | 95% | 97% | 0 | 0 | 0 | 0 | 0 | 0 |
| 889 | Phosphatidylinositide phosphatase SAC1 OS=Homo sapiens GN=SACM1L PE=1 SV=2 | SAC1 | 67 | 0.073 | 100% | 100% | 100% | 100% | 100% | 100% | 100% | 100% | 100% | 100% | 100% | 100% |
| 890 | Monoacylglycerol lipase ABHD12 OS=Homo sapiens GN=ABHD12 PE=1 SV=2 | ABD12 | 45 | 0.2 | 100% | 100% | 100% | 100% | 100% | 100% | 100% | 100% | 100% | 100% | 100% | 100% |
| 891 | Nexilin OS=Homo sapiens GN=NEXN PE=1 SV=1 | NEXN | 81 | 0.16 | 0 | 0 | 0 | 0 | 100% | 100% | 6% | 0 | 0 | 0 | 95% | 95% |
| 892 | Vezatin OS=Homo sapiens GN=VEZT PE=1 SV=3 | VEZA | 89 | 0.6 | 0 | 0 | 0 | 0 | 95% | 0 | 0 | 90% | 0 | 0 | 0 | 0 |
| 893 | Protein AHNAK2 OS=Homo sapiens GN=AHNAK2 PE=1 SV=2 | AHNK2 | 617 | < 0.00010 | 100% | 100% | 100% | 100% | 100% | 100% | 100% | 100% | 100% | 100% | 100% | 100% |
| 894 | Voltage-dependent calcium channel subunit alpha-2/delta-1 OS=Homo sapiens GN=CACNA2D1 PE=1 SV=3 | CA2D1 | 125 | 0.022 | 100% | 100% | 100% | 100% | 100% | 100% | 100% | 100% | 100% | 100% | 100% | 100% |
| 895 | Dysferlin OS=Homo sapiens GN=DYSF PE=1 SV=1 | DYSF | 237 | 0.00019 | 0 | 45% | 0 | 100% | 100% | 100% | 0 | 0 | 0 | 100% | 100% | 100% |
| 896 | Zinc transporter 1 OS=Homo sapiens GN=SLC30A1 PE=1 SV=3 | ZNT1 | 55 | 0.023 | 0 | 23% | 95% | 100% | 100% | 100% | 13% | 0 | 86% | 93% | 13% | 0 |
| 897 | Major vault protein OS=Homo sapiens GN=MVP PE=1 SV=4 | MVP | 99 | < 0.00010 | 100% | 100% | 100% | 100% | 100% | 100% | 100% | 100% | 100% | 100% | 100% | 100% |
| 898 | Prelamin-A/C OS=Homo sapiens GN=LMNA PE=1 SV=1 | LMNA | 74 | 0.00056 | 100% | 100% | 100% | 100% | 100% | 100% | 100% | 100% | 100% | 100% | 100% | 100% |
| 899 | Heterogeneous nuclear ribonucleoproteins A2/B1 OS=Homo sapiens GN=HNRNPA2B1 PE=1 SV=2 | ROA2 | 37 | 0.00025 | 100% | 100% | 100% | 100% | 100% | 100% | 100% | 100% | 100% | 100% | 100% | 100% |
| 900 | Cytoplasmic dynein 1 heavy chain 1 OS=Homo sapiens GN=DYNC1H1 PE=1 SV=5 | DYHC1 | 532 | < 0.00010 | 100% | 100% | 100% | 100% | 100% | 100% | 100% | 100% | 100% | 100% | 100% | 100% |
| 901 | Collagen alpha-1(I) chain OS=Homo sapiens GN=COL1A1 PE=1 SV=5 | CO1A1 | 139 | < 0.00010 | 100% | 100% | 100% | 100% | 100% | 100% | 100% | 100% | 100% | 100% | 100% | 100% |
| 902 | Dolichyl-diphosphooligosaccharide--protein glycosyltransferase subunit 1 OS=Homo sapiens GN=RPN1 PE=1 SV=1 | RPN1 | 69 | 0.063 | 100% | 100% | 100% | 100% | 100% | 100% | 100% | 100% | 100% | 100% | 100% | 100% |
| 903 | DNA-dependent protein kinase catalytic subunit OS=Homo sapiens GN=PRKDC PE=1 SV=3 | PRKDC | 469 | 0.00092 | 100% | 100% | 100% | 100% | 100% | 100% | 100% | 100% | 100% | 100% | 100% | 100% |
| 904 | Collagen alpha-1(XII) chain OS=Homo sapiens GN=COL12A1 PE=1 SV=2 | COCA1 | 333 | < 0.00010 | 100% | 100% | 100% | 100% | 100% | 100% | 100% | 100% | 100% | 100% | 100% | 100% |
| 905 | Cluster of Histone H2A type 2-C OS=Homo sapiens GN=HIST2H2AC PE=1 SV=4 (H2A2C_HUMAN) | H2A2C [6] | 14 | 0.00079 | 100% | 100% | 100% | 100% | 100% | 100% | 100% | 100% | 100% | 100% | 100% | 100% |
| 906 | Histone H4 OS=Homo sapiens GN=HIST1H4A PE=1 SV=2 | H4 | 11 | 0.073 | 100% | 100% | 100% | 100% | 100% | 100% | 100% | 100% | 100% | 100% | 100% | 100% |
| 907 | Cluster of Heterogeneous nuclear ribonucleoprotein A1 OS=Homo sapiens GN=HNRNPA1 PE=1 SV=5 (ROA1_HUMAN) | ROA1 [2] | 39 | 0.12 | 100% | 100% | 100% | 100% | 100% | 100% | 100% | 100% | 100% | 100% | 100% | 100% |
| 908 | Serpin H1 OS=Homo sapiens GN=SERPINH1 PE=1 SV=2 | SERPH | 46 | 0.0033 | 100% | 100% | 100% | 100% | 100% | 100% | 100% | 100% | 100% | 100% | 100% | 100% |
| 909 | NADH-cytochrome b5 reductase 3 OS=Homo sapiens GN=CYB5R3 PE=1 SV=3 | NB5R3 | 34 | 0.0027 | 100% | 100% | 100% | 100% | 100% | 100% | 100% | 100% | 100% | 100% | 100% | 100% |
| 910 | Collagen alpha-2(I) chain OS=Homo sapiens GN=COL1A2 PE=1 SV=7 | CO1A2 | 129 | 0.00026 | 100% | 100% | 100% | 100% | 100% | 100% | 100% | 100% | 100% | 100% | 100% | 100% |
| 911 | Heterogeneous nuclear ribonucleoprotein U OS=Homo sapiens GN=HNRNPU PE=1 SV=6 | HNRPU | 91 | 0.00013 | 100% | 100% | 100% | 100% | 100% | 100% | 100% | 100% | 100% | 100% | 100% | 100% |
| 912 | Cluster of Heterogeneous nuclear ribonucleoproteins C1/C2 OS=Homo sapiens GN=HNRNPC PE=1 SV=4 (HNRPC_HUMAN) | HNRPC [2] | 34 | 0.22 | 100% | 100% | 100% | 100% | 100% | 100% | 100% | 100% | 100% | 100% | 100% | 100% |
| 913 | Nucleolin OS=Homo sapiens GN=NCL PE=1 SV=3 | NUCL | 77 | 0.0093 | 100% | 100% | 100% | 100% | 100% | 100% | 100% | 100% | 100% | 100% | 100% | 100% |
| 914 | Atlastin-3 OS=Homo sapiens GN=ATL3 PE=1 SV=1 | ATLA3 | 61 | 0.05 | 100% | 100% | 100% | 100% | 100% | 100% | 100% | 100% | 100% | 100% | 100% | 100% |
| 915 | Interleukin enhancer-binding factor 3 OS=Homo sapiens GN=ILF3 PE=1 SV=3 | ILF3 | 95 | 0.0099 | 100% | 100% | 100% | 100% | 100% | 100% | 100% | 100% | 100% | 100% | 100% | 100% |
| 916 | ATP-dependent RNA helicase A OS=Homo sapiens GN=DHX9 PE=1 SV=4 | DHX9 | 141 | 0.00028 | 100% | 100% | 100% | 100% | 100% | 100% | 100% | 100% | 100% | 100% | 100% | 100% |
| 917 | Protein ERGIC-53 OS=Homo sapiens GN=LMAN1 PE=1 SV=2 | LMAN1 | 58 | 0.022 | 100% | 100% | 100% | 100% | 100% | 100% | 100% | 100% | 100% | 100% | 100% | 100% |
| 918 | Heterogeneous nuclear ribonucleoprotein L OS=Homo sapiens GN=HNRNPL PE=1 SV=2 | HNRPL | 64 | 0.044 | 100% | 100% | 100% | 100% | 100% | 100% | 100% | 100% | 100% | 100% | 100% | 100% |
| 919 | Polypeptide N-acetylgalactosaminyltransferase 2 OS=Homo sapiens GN=GALNT2 PE=1 SV=1 | GALT2 | 65 | 0.081 | 100% | 100% | 100% | 100% | 100% | 100% | 100% | 100% | 100% | 100% | 100% | 100% |
| 920 | Neutral alpha-glucosidase AB OS=Homo sapiens GN=GANAB PE=1 SV=3 | GANAB | 107 | 0.47 | 100% | 100% | 100% | 100% | 100% | 100% | 100% | 100% | 100% | 100% | 100% | 100% |
| 921 | Cluster of RNA-binding motif protein, X chromosome OS=Homo sapiens GN=RBMX PE=1 SV=3 (RBMX_HUMAN) | RBMX [3] | 42 | 0.21 | 100% | 100% | 100% | 100% | 100% | 100% | 100% | 100% | 100% | 100% | 100% | 100% |
| 922 | Pre-mRNA-processing-splicing factor 8 OS=Homo sapiens GN=PRPF8 PE=1 SV=2 | PRP8 | 274 | < 0.00010 | 100% | 100% | 100% | 100% | 100% | 100% | 100% | 100% | 100% | 100% | 100% | 100% |
| 923 | Dolichyl-diphosphooligosaccharide--protein glycosyltransferase subunit 2 OS=Homo sapiens GN=RPN2 PE=1 SV=3 | RPN2 | 69 | 0.03 | 100% | 100% | 100% | 100% | 100% | 100% | 100% | 100% | 100% | 100% | 100% | 100% |
| 924 | Matrin-3 OS=Homo sapiens GN=MATR3 PE=1 SV=2 | MATR3 | 95 | 0.0066 | 100% | 100% | 100% | 100% | 100% | 100% | 100% | 100% | 100% | 100% | 100% | 100% |
| 925 | Heterogeneous nuclear ribonucleoprotein A3 OS=Homo sapiens GN=HNRNPA3 PE=1 SV=2 | ROA3 | 40 | 0.0029 | 100% | 100% | 100% | 100% | 100% | 100% | 100% | 100% | 100% | 100% | 100% | 100% |
| 926 | Cluster of 60S ribosomal protein L10 OS=Homo sapiens GN=RPL10 PE=1 SV=4 (RL10_HUMAN) | RL10 [2] | 25 | 0.1 | 100% | 100% | 100% | 100% | 100% | 100% | 100% | 100% | 100% | 100% | 100% | 100% |
| 927 | Procollagen-lysine,2-oxoglutarate 5-dioxygenase 2 OS=Homo sapiens GN=PLOD2 PE=1 SV=2 | PLOD2 | 85 | < 0.00010 | 100% | 100% | 100% | 100% | 100% | 100% | 100% | 100% | 100% | 100% | 100% | 100% |
| 928 | Galectin-1 OS=Homo sapiens GN=LGALS1 PE=1 SV=2 | LEG1 | 15 | 0.098 | 100% | 100% | 100% | 100% | 100% | 100% | 100% | 100% | 100% | 100% | 100% | 100% |
| 929 | DnaJ homolog subfamily C member 13 OS=Homo sapiens GN=DNAJC13 PE=1 SV=5 | DJC13 | 254 | 0.57 | 100% | 100% | 100% | 100% | 100% | 100% | 100% | 100% | 100% | 100% | 100% | 100% |
| 930 | U5 small nuclear ribonucleoprotein 200 kDa helicase OS=Homo sapiens GN=SNRNP200 PE=1 SV=2 | U520 | 245 | 0.0036 | 100% | 100% | 100% | 100% | 100% | 100% | 100% | 100% | 100% | 100% | 100% | 100% |
| 931 | Transmembrane protein 43 OS=Homo sapiens GN=TMEM43 PE=1 SV=1 | TMM43 | 45 | 0.34 | 100% | 100% | 100% | 100% | 100% | 100% | 100% | 100% | 100% | 100% | 100% | 100% |
| 932 | Adipocyte plasma membrane-associated protein OS=Homo sapiens GN=APMAP PE=1 SV=2 | APMAP | 46 | 0.26 | 100% | 100% | 100% | 100% | 100% | 100% | 100% | 100% | 100% | 100% | 100% | 100% |
| 933 | 116 kDa U5 small nuclear ribonucleoprotein component OS=Homo sapiens GN=EFTUD2 PE=1 SV=1 | U5S1 | 109 | 0.011 | 100% | 100% | 100% | 100% | 100% | 100% | 100% | 100% | 100% | 100% | 100% | 100% |
| 934 | Interleukin enhancer-binding factor 2 OS=Homo sapiens GN=ILF2 PE=1 SV=2 | ILF2 | 43 | 0.0066 | 100% | 100% | 100% | 100% | 100% | 100% | 100% | 100% | 100% | 100% | 100% | 100% |
| 935 | Synaptic vesicle membrane protein VAT-1 homolog OS=Homo sapiens GN=VAT1 PE=1 SV=2 | VAT1 | 42 | 0.0045 | 100% | 100% | 100% | 100% | 100% | 100% | 100% | 100% | 100% | 100% | 100% | 100% |
| 936 | Epoxide hydrolase 1 OS=Homo sapiens GN=EPHX1 PE=1 SV=1 | HYEP | 53 | 0.39 | 100% | 100% | 100% | 100% | 100% | 100% | 100% | 100% | 100% | 100% | 100% | 100% |
| 937 | Vesicle-trafficking protein SEC22b OS=Homo sapiens GN=SEC22B PE=1 SV=4 | SC22B | 25 | 0.0011 | 100% | 100% | 100% | 100% | 100% | 100% | 100% | 100% | 100% | 100% | 100% | 100% |
| 938 | Cluster of Protein transport protein Sec23A OS=Homo sapiens GN=SEC23A PE=1 SV=2 (SC23A_HUMAN) | SC23A [2] | 86 | 0.00046 | 100% | 100% | 100% | 100% | 100% | 100% | 100% | 100% | 100% | 100% | 100% | 100% |
| 939 | Polypyrimidine tract-binding protein 1 OS=Homo sapiens GN=PTBP1 PE=1 SV=1 | PTBP1 | 57 | 0.0031 | 100% | 100% | 100% | 100% | 100% | 100% | 100% | 100% | 100% | 100% | 100% | 100% |
| 940 | X-ray repair cross-complementing protein 6 OS=Homo sapiens GN=XRCC6 PE=1 SV=2 | XRCC6 | 70 | 0.27 | 100% | 100% | 100% | 100% | 100% | 100% | 100% | 100% | 100% | 100% | 100% | 100% |
| 941 | Splicing factor 3B subunit 1 OS=Homo sapiens GN=SF3B1 PE=1 SV=3 | SF3B1 | 146 | 0.00077 | 100% | 100% | 100% | 100% | 100% | 100% | 100% | 100% | 100% | 100% | 100% | 100% |
| 942 | Neutral cholesterol ester hydrolase 1 OS=Homo sapiens GN=NCEH1 PE=1 SV=3 | NCEH1 | 46 | 0.0031 | 100% | 100% | 100% | 100% | 100% | 100% | 100% | 100% | 100% | 100% | 100% | 100% |
| 943 | Heterogeneous nuclear ribonucleoprotein U-like protein 2 OS=Homo sapiens GN=HNRNPUL2 PE=1 SV=1 | HNRL2 | 85 | 0.1 | 100% | 100% | 100% | 100% | 100% | 100% | 100% | 100% | 100% | 100% | 100% | 100% |
| 944 | Ribosome-binding protein 1 OS=Homo sapiens GN=RRBP1 PE=1 SV=4 | RRBP1 | 152 | 0.55 | 100% | 100% | 100% | 100% | 100% | 100% | 100% | 100% | 100% | 100% | 100% | 100% |
| 945 | Long-chain-fatty-acid--CoA ligase 3 OS=Homo sapiens GN=ACSL3 PE=1 SV=3 | ACSL3 | 80 | 0.0017 | 100% | 100% | 100% | 100% | 100% | 100% | 100% | 100% | 100% | 100% | 100% | 100% |
| 946 | Ras-related protein Rab-7a OS=Homo sapiens GN=RAB7A PE=1 SV=1 | RAB7A | 23 | 0.0068 | 100% | 100% | 100% | 100% | 100% | 100% | 100% | 100% | 100% | 100% | 100% | 100% |
| 947 | Keratin, type I cytoskeletal 9 OS=Homo sapiens GN=KRT9 PE=1 SV=3 | K1C9 | 62 | 0.00077 | 100% | 100% | 100% | 100% | 100% | 100% | 100% | 100% | 100% | 100% | 100% | 100% |
| 948 | Heterogeneous nuclear ribonucleoprotein R OS=Homo sapiens GN=HNRNPR PE=1 SV=1 | HNRPR | 71 | 0.33 | 100% | 100% | 100% | 100% | 100% | 100% | 100% | 100% | 100% | 100% | 100% | 100% |
| 949 | Acetolactate synthase-like protein OS=Homo sapiens GN=ILVBL PE=1 SV=2 | ILVBL | 68 | 0.032 | 100% | 100% | 100% | 100% | 100% | 100% | 100% | 100% | 100% | 100% | 100% | 100% |
| 950 | NADPH--cytochrome P450 reductase OS=Homo sapiens GN=POR PE=1 SV=2 | NCPR | 77 | 0.77 | 100% | 100% | 100% | 100% | 100% | 100% | 100% | 100% | 100% | 100% | 100% | 100% |
| 951 | 60S ribosomal protein L13 OS=Homo sapiens GN=RPL13 PE=1 SV=4 | RL13 | 24 | 0.23 | 100% | 100% | 100% | 100% | 100% | 100% | 100% | 100% | 100% | 100% | 100% | 100% |
| 952 | Procollagen-lysine,2-oxoglutarate 5-dioxygenase 1 OS=Homo sapiens GN=PLOD1 PE=1 SV=2 | PLOD1 | 84 | 0.0021 | 100% | 100% | 100% | 100% | 100% | 100% | 100% | 100% | 100% | 100% | 100% | 100% |
| 953 | Dolichyl-diphosphooligosaccharide--protein glycosyltransferase 48 kDa subunit OS=Homo sapiens GN=DDOST PE=1 SV=4 | OST48 | 51 | 0.011 | 100% | 100% | 100% | 100% | 100% | 100% | 100% | 100% | 100% | 100% | 100% | 100% |
| 954 | Surfeit locus protein 4 OS=Homo sapiens GN=SURF4 PE=1 SV=3 | SURF4 | 30 | 0.081 | 100% | 100% | 100% | 100% | 100% | 100% | 100% | 100% | 100% | 100% | 100% | 100% |
| 955 | 40S ribosomal protein S6 OS=Homo sapiens GN=RPS6 PE=1 SV=1 | RS6 | 29 | 0.034 | 100% | 100% | 100% | 100% | 100% | 100% | 100% | 100% | 100% | 100% | 100% | 100% |
| 956 | Splicing factor, proline- and glutamine-rich OS=Homo sapiens GN=SFPQ PE=1 SV=2 | SFPQ | 76 | 0.021 | 100% | 100% | 100% | 100% | 100% | 100% | 100% | 100% | 100% | 100% | 100% | 100% |
| 957 | Heterogeneous nuclear ribonucleoprotein Q OS=Homo sapiens GN=SYNCRIP PE=1 SV=2 | HNRPQ | 70 | 0.24 | 100% | 100% | 100% | 100% | 100% | 100% | 100% | 100% | 100% | 100% | 100% | 100% |
| 958 | Mannosyl-oligosaccharide glucosidase OS=Homo sapiens GN=MOGS PE=1 SV=5 | MOGS | 92 | 0.0087 | 100% | 100% | 100% | 100% | 100% | 100% | 100% | 100% | 100% | 100% | 100% | 100% |
| 959 | Cathepsin D OS=Homo sapiens GN=CTSD PE=1 SV=1 | CATD | 45 | 0.21 | 100% | 100% | 100% | 100% | 100% | 100% | 100% | 100% | 100% | 100% | 100% | 100% |
| 960 | Alpha-mannosidase 2 OS=Homo sapiens GN=MAN2A1 PE=1 SV=2 | MA2A1 | 131 | 0.024 | 100% | 100% | 100% | 100% | 100% | 100% | 100% | 100% | 100% | 100% | 100% | 100% |
| 961 | Estradiol 17-beta-dehydrogenase 12 OS=Homo sapiens GN=HSD17B12 PE=1 SV=2 | DHB12 | 34 | 0.45 | 100% | 100% | 100% | 100% | 100% | 100% | 100% | 100% | 100% | 100% | 100% | 100% |
| 962 | E3 UFM1-protein ligase 1 OS=Homo sapiens GN=UFL1 PE=1 SV=2 | UFL1 | 90 | 0.086 | 100% | 100% | 100% | 100% | 100% | 100% | 100% | 100% | 100% | 100% | 100% | 100% |
| 963 | 60S ribosomal protein L17 OS=Homo sapiens GN=RPL17 PE=1 SV=3 | RL17 | 21 | 0.35 | 100% | 100% | 100% | 100% | 100% | 100% | 100% | 100% | 100% | 100% | 100% | 100% |
| 964 | Membrane-associated progesterone receptor component 1 OS=Homo sapiens GN=PGRMC1 PE=1 SV=3 | PGRC1 | 22 | 0.52 | 100% | 100% | 100% | 100% | 100% | 100% | 100% | 100% | 100% | 100% | 100% | 100% |
| 965 | Cluster of Heterogeneous nuclear ribonucleoprotein H OS=Homo sapiens GN=HNRNPH1 PE=1 SV=4 (HNRH1_HUMAN) | HNRH1 [2] | 49 | 0.62 | 100% | 100% | 100% | 100% | 100% | 100% | 100% | 100% | 100% | 100% | 100% | 100% |
| 966 | Prolyl 4-hydroxylase subunit alpha-1 OS=Homo sapiens GN=P4HA1 PE=1 SV=2 | P4HA1 | 61 | 0.067 | 100% | 100% | 100% | 100% | 100% | 100% | 100% | 100% | 100% | 100% | 100% | 100% |
| 967 | Prohibitin-2 OS=Homo sapiens GN=PHB2 PE=1 SV=2 | PHB2 | 33 | < 0.00010 | 100% | 100% | 100% | 100% | 100% | 100% | 100% | 100% | 100% | 100% | 100% | 100% |
| 968 | Glucosylceramidase OS=Homo sapiens GN=GBA PE=1 SV=3 | GLCM | 60 | 0.0058 | 100% | 100% | 100% | 100% | 100% | 100% | 100% | 100% | 100% | 100% | 100% | 100% |
| 969 | Transmembrane protein 165 OS=Homo sapiens GN=TMEM165 PE=1 SV=1 | TM165 | 35 | 0.00034 | 100% | 100% | 100% | 100% | 100% | 100% | 100% | 100% | 100% | 100% | 100% | 100% |
| 970 | Splicing factor 3B subunit 3 OS=Homo sapiens GN=SF3B3 PE=1 SV=4 | SF3B3 | 136 | 0.064 | 100% | 100% | 100% | 100% | 100% | 100% | 100% | 100% | 100% | 100% | 100% | 100% |
| 971 | RNA-binding protein Raly OS=Homo sapiens GN=RALY PE=1 SV=1 | RALY | 32 | 0.00062 | 100% | 100% | 100% | 100% | 100% | 100% | 100% | 100% | 100% | 100% | 100% | 100% |
| 972 | KH domain-containing, RNA-binding, signal transduction-associated protein 1 OS=Homo sapiens GN=KHDRBS1 PE=1 SV=1 | KHDR1 | 48 | 0.05 | 100% | 100% | 100% | 100% | 100% | 100% | 100% | 100% | 100% | 100% | 100% | 100% |
| 973 | Histone H1.5 OS=Homo sapiens GN=HIST1H1B PE=1 SV=3 | H15 | 23 | 0.0038 | 100% | 100% | 100% | 100% | 100% | 100% | 100% | 100% | 100% | 100% | 100% | 100% |
| 974 | Cluster of 60S ribosomal protein L26 OS=Homo sapiens GN=RPL26 PE=1 SV=1 (RL26_HUMAN) | RL26 [2] | 17 | 0.46 | 100% | 100% | 100% | 100% | 100% | 100% | 100% | 100% | 100% | 100% | 100% | 100% |
| 975 | Non-POU domain-containing octamer-binding protein OS=Homo sapiens GN=NONO PE=1 SV=4 | NONO | 54 | 0.018 | 100% | 100% | 100% | 100% | 100% | 100% | 100% | 100% | 100% | 100% | 100% | 100% |
| 976 | Putative pre-mRNA-splicing factor ATP-dependent RNA helicase DHX15 OS=Homo sapiens GN=DHX15 PE=1 SV=2 | DHX15 | 91 | 0.0086 | 100% | 100% | 100% | 100% | 100% | 100% | 100% | 100% | 100% | 100% | 100% | 100% |
| 977 | Eukaryotic translation initiation factor 3 subunit A OS=Homo sapiens GN=EIF3A PE=1 SV=1 | EIF3A | 167 | 0.0088 | 100% | 100% | 100% | 100% | 100% | 100% | 100% | 100% | 100% | 100% | 100% | 100% |
| 978 | Trifunctional enzyme subunit alpha, mitochondrial OS=Homo sapiens GN=HADHA PE=1 SV=2 | ECHA | 83 | 0.00036 | 100% | 100% | 100% | 100% | 100% | 100% | 100% | 100% | 100% | 100% | 100% | 100% |
| 979 | Transmembrane emp24 domain-containing protein 9 OS=Homo sapiens GN=TMED9 PE=1 SV=2 | TMED9 | 27 | 0.0021 | 100% | 100% | 100% | 100% | 100% | 100% | 100% | 100% | 100% | 100% | 100% | 100% |
| 980 | Heterogeneous nuclear ribonucleoprotein D0 OS=Homo sapiens GN=HNRNPD PE=1 SV=1 | HNRPD | 38 | 0.13 | 100% | 100% | 100% | 100% | 100% | 100% | 100% | 100% | 100% | 100% | 100% | 100% |
| 981 | Splicing factor 3B subunit 2 OS=Homo sapiens GN=SF3B2 PE=1 SV=2 | SF3B2 | 100 | 0.2 | 100% | 100% | 100% | 100% | 100% | 100% | 100% | 100% | 100% | 100% | 100% | 100% |
| 982 | Lanosterol synthase OS=Homo sapiens GN=LSS PE=1 SV=1 | ERG7 | 83 | 0.01 | 100% | 100% | 100% | 100% | 100% | 100% | 100% | 100% | 100% | 100% | 100% | 100% |
| 983 | Double-stranded RNA-specific adenosine deaminase OS=Homo sapiens GN=ADAR PE=1 SV=4 | DSRAD | 136 | 0.046 | 100% | 100% | 100% | 100% | 100% | 100% | 100% | 100% | 100% | 100% | 100% | 100% |
| 984 | Protein transport protein Sec61 subunit alpha isoform 1 OS=Homo sapiens GN=SEC61A1 PE=1 SV=2 | S61A1 | 52 | 0.29 | 100% | 100% | 100% | 100% | 100% | 100% | 100% | 100% | 100% | 100% | 100% | 100% |
| 985 | Nodal modulator 2 OS=Homo sapiens GN=NOMO2 PE=1 SV=1 | NOMO2 (+1) | 139 | 0.0089 | 100% | 100% | 100% | 100% | 100% | 100% | 100% | 100% | 100% | 100% | 100% | 100% |
| 986 | Nucleolar RNA helicase 2 OS=Homo sapiens GN=DDX21 PE=1 SV=5 | DDX21 | 87 | 0.00022 | 100% | 100% | 100% | 100% | 100% | 100% | 100% | 100% | 100% | 100% | 100% | 100% |
| 987 | 60S ribosomal protein L21 OS=Homo sapiens GN=RPL21 PE=1 SV=2 | RL21 | 19 | 0.28 | 100% | 100% | 100% | 100% | 100% | 100% | 100% | 100% | 100% | 100% | 100% | 100% |
| 988 | Signal recognition particle receptor subunit alpha OS=Homo sapiens GN=SRPR PE=1 SV=2 | SRPR | 70 | < 0.00010 | 100% | 100% | 100% | 100% | 100% | 100% | 100% | 100% | 100% | 100% | 100% | 100% |
| 989 | Eukaryotic initiation factor 4A-III OS=Homo sapiens GN=EIF4A3 PE=1 SV=4 | IF4A3 | 47 | 0.009 | 100% | 100% | 100% | 100% | 100% | 100% | 100% | 100% | 100% | 100% | 100% | 100% |
| 990 | Nucleobindin-1 OS=Homo sapiens GN=NUCB1 PE=1 SV=4 | NUCB1 | 54 | 0.022 | 100% | 100% | 100% | 100% | 100% | 100% | 100% | 100% | 100% | 100% | 100% | 100% |
| 991 | Serine/arginine-rich splicing factor 3 OS=Homo sapiens GN=SRSF3 PE=1 SV=1 | SRSF3 | 19 | 0.5 | 100% | 100% | 100% | 100% | 100% | 100% | 100% | 100% | 100% | 100% | 100% | 100% |
| 992 | Golgi integral membrane protein 4 OS=Homo sapiens GN=GOLIM4 PE=1 SV=1 | GOLI4 | 82 | 0.0073 | 100% | 100% | 100% | 100% | 100% | 100% | 100% | 100% | 100% | 100% | 100% | 100% |
| 993 | Sulfide:quinone oxidoreductase, mitochondrial OS=Homo sapiens GN=SQRDL PE=1 SV=1 | SQRD | 50 | < 0.00010 | 100% | 100% | 100% | 100% | 100% | 100% | 100% | 100% | 100% | 100% | 100% | 100% |
| 994 | Endonuclease domain-containing 1 protein OS=Homo sapiens GN=ENDOD1 PE=1 SV=2 | ENDD1 | 55 | 0.54 | 100% | 100% | 100% | 100% | 100% | 100% | 100% | 100% | 100% | 100% | 100% | 100% |
| 995 | Collagen alpha-1(V) chain OS=Homo sapiens GN=COL5A1 PE=1 SV=3 | CO5A1 | 184 | 0.079 | 100% | 100% | 100% | 100% | 100% | 100% | 100% | 100% | 100% | 100% | 100% | 100% |
| 996 | Heterogeneous nuclear ribonucleoprotein H3 OS=Homo sapiens GN=HNRNPH3 PE=1 SV=2 | HNRH3 | 37 | 0.039 | 100% | 100% | 100% | 100% | 100% | 100% | 100% | 100% | 100% | 100% | 100% | 100% |
| 997 | T-complex protein 1 subunit eta OS=Homo sapiens GN=CCT7 PE=1 SV=2 | TCPH | 59 | 0.15 | 100% | 100% | 100% | 100% | 100% | 100% | 100% | 100% | 100% | 100% | 100% | 100% |
| 998 | ER membrane protein complex subunit 1 OS=Homo sapiens GN=EMC1 PE=1 SV=1 | EMC1 | 112 | 0.46 | 100% | 100% | 100% | 100% | 100% | 100% | 100% | 100% | 100% | 100% | 100% | 100% |
| 999 | Cluster of Histone H1.3 OS=Homo sapiens GN=HIST1H1D PE=1 SV=2 (H13_HUMAN) | H13 [4] | 22 | 0.0056 | 100% | 100% | 100% | 100% | 100% | 100% | 100% | 100% | 100% | 100% | 100% | 100% |
| 1000 | Endoplasmic reticulum resident protein 44 OS=Homo sapiens GN=ERP44 PE=1 SV=1 | ERP44 | 47 | 0.0051 | 100% | 100% | 100% | 100% | 100% | 100% | 100% | 100% | 100% | 100% | 100% | 100% |
| 1001 | Syntaxin-12 OS=Homo sapiens GN=STX12 PE=1 SV=1 | STX12 | 32 | 0.0021 | 100% | 100% | 100% | 100% | 100% | 100% | 100% | 100% | 100% | 100% | 100% | 100% |
| 1002 | Cluster of Ras-related protein Rab-6A OS=Homo sapiens GN=RAB6A PE=1 SV=3 (RAB6A_HUMAN) | RAB6A [2] | 24 | 0.05 | 100% | 100% | 100% | 100% | 100% | 100% | 100% | 100% | 100% | 100% | 100% | 100% |
| 1003 | Nicalin OS=Homo sapiens GN=NCLN PE=1 SV=2 | NCLN | 63 | 0.27 | 100% | 100% | 100% | 100% | 100% | 100% | 100% | 100% | 100% | 100% | 100% | 100% |
| 1004 | 60S ribosomal protein L28 OS=Homo sapiens GN=RPL28 PE=1 SV=3 | RL28 | 16 | 0.87 | 100% | 100% | 100% | 100% | 100% | 100% | 100% | 100% | 100% | 100% | 100% | 100% |
| 1005 | 60S ribosomal protein L18a OS=Homo sapiens GN=RPL18A PE=1 SV=2 | RL18A | 21 | 0.43 | 100% | 100% | 100% | 100% | 100% | 100% | 100% | 100% | 100% | 100% | 100% | 100% |
| 1006 | T-complex protein 1 subunit delta OS=Homo sapiens GN=CCT4 PE=1 SV=4 | TCPD | 58 | 0.016 | 100% | 100% | 100% | 100% | 100% | 100% | 100% | 100% | 100% | 100% | 100% | 100% |
| 1007 | Endoplasmic reticulum-Golgi intermediate compartment protein 1 OS=Homo sapiens GN=ERGIC1 PE=1 SV=1 | ERGI1 | 33 | 0.7 | 100% | 100% | 100% | 100% | 100% | 100% | 100% | 100% | 100% | 100% | 100% | 100% |
| 1008 | Glutamate dehydrogenase 1, mitochondrial OS=Homo sapiens GN=GLUD1 PE=1 SV=2 | DHE3 | 61 | 0.0096 | 100% | 100% | 100% | 100% | 100% | 100% | 100% | 100% | 100% | 100% | 100% | 100% |
| 1009 | Splicing factor 3A subunit 1 OS=Homo sapiens GN=SF3A1 PE=1 SV=1 | SF3A1 | 89 | 0.00092 | 100% | 100% | 100% | 100% | 100% | 100% | 100% | 100% | 100% | 100% | 100% | 100% |
| 1010 | Coiled-coil domain-containing protein 47 OS=Homo sapiens GN=CCDC47 PE=1 SV=1 | CCD47 | 56 | 0.36 | 100% | 100% | 100% | 100% | 100% | 100% | 100% | 100% | 100% | 100% | 100% | 100% |
| 1011 | Protein disulfide-isomerase A4 OS=Homo sapiens GN=PDIA4 PE=1 SV=2 | PDIA4 | 73 | 0.11 | 100% | 100% | 100% | 100% | 100% | 100% | 100% | 100% | 100% | 100% | 100% | 100% |
| 1012 | Cluster of ADP-ribosylation factor-like protein 8B OS=Homo sapiens GN=ARL8B PE=1 SV=1 (ARL8B_HUMAN) | ARL8B [2] | 22 | 0.0031 | 100% | 100% | 100% | 100% | 100% | 100% | 100% | 100% | 100% | 100% | 100% | 100% |
| 1013 | Cluster of Scaffold attachment factor B1 OS=Homo sapiens GN=SAFB PE=1 SV=4 (SAFB1_HUMAN) | SAFB1 [2] | 103 | 0.14 | 100% | 100% | 100% | 100% | 100% | 100% | 100% | 100% | 100% | 100% | 100% | 100% |
| 1014 | Receptor expression-enhancing protein 5 OS=Homo sapiens GN=REEP5 PE=1 SV=3 | REEP5 | 21 | 0.11 | 100% | 100% | 100% | 100% | 100% | 100% | 100% | 100% | 100% | 100% | 100% | 100% |
| 1015 | Protein lunapark OS=Homo sapiens GN=LNP PE=1 SV=2 | LNP | 48 | 0.51 | 100% | 100% | 100% | 100% | 100% | 100% | 100% | 100% | 100% | 100% | 100% | 100% |
| 1016 | Cluster of Alpha-2-macroglobulin OS=Homo sapiens GN=A2M PE=1 SV=3 (A2MG_HUMAN) | A2MG [2] | 163 | 0.0013 | 100% | 100% | 100% | 100% | 100% | 100% | 100% | 100% | 100% | 100% | 100% | 100% |
| 1017 | Pre-mRNA-processing factor 19 OS=Homo sapiens GN=PRPF19 PE=1 SV=1 | PRP19 | 55 | 0.0061 | 100% | 100% | 100% | 100% | 100% | 100% | 100% | 100% | 100% | 100% | 100% | 100% |
| 1018 | Wolframin OS=Homo sapiens GN=WFS1 PE=1 SV=2 | WFS1 | 100 | 0.00029 | 100% | 100% | 100% | 100% | 100% | 100% | 100% | 100% | 100% | 100% | 100% | 100% |
| 1019 | Dolichyl-diphosphooligosaccharide--protein glycosyltransferase subunit STT3A OS=Homo sapiens GN=STT3A PE=1 SV=2 | STT3A | 81 | 0.053 | 100% | 100% | 100% | 100% | 100% | 100% | 100% | 100% | 100% | 100% | 100% | 100% |
| 1020 | ATP-dependent RNA helicase DDX1 OS=Homo sapiens GN=DDX1 PE=1 SV=2 | DDX1 | 82 | 0.033 | 100% | 100% | 100% | 100% | 100% | 100% | 100% | 100% | 100% | 100% | 100% | 100% |
| 1021 | Probable ATP-dependent RNA helicase DDX5 OS=Homo sapiens GN=DDX5 PE=1 SV=1 | DDX5 | 69 | 0.23 | 100% | 100% | 100% | 100% | 100% | 100% | 100% | 100% | 100% | 100% | 100% | 100% |
| 1022 | Procollagen-lysine,2-oxoglutarate 5-dioxygenase 3 OS=Homo sapiens GN=PLOD3 PE=1 SV=1 | PLOD3 | 85 | 0.049 | 100% | 100% | 100% | 100% | 100% | 100% | 100% | 100% | 100% | 100% | 100% | 100% |
| 1023 | Cluster of ELAV-like protein 1 OS=Homo sapiens GN=ELAVL1 PE=1 SV=2 (ELAV1_HUMAN) | ELAV1 [2] | 36 | 0.33 | 100% | 100% | 100% | 100% | 100% | 100% | 100% | 100% | 100% | 100% | 100% | 100% |
| 1024 | Transmembrane emp24 domain-containing protein 7 OS=Homo sapiens GN=TMED7 PE=1 SV=2 | TMED7 | 25 | < 0.00010 | 100% | 100% | 100% | 100% | 100% | 100% | 100% | 100% | 100% | 100% | 100% | 100% |
| 1025 | Saccharopine dehydrogenase-like oxidoreductase OS=Homo sapiens GN=SCCPDH PE=1 SV=1 | SCPDL | 47 | 0.32 | 100% | 100% | 100% | 100% | 100% | 100% | 100% | 100% | 100% | 100% | 100% | 100% |
| 1026 | Cluster of Myosin light polypeptide 6 OS=Homo sapiens GN=MYL6 PE=1 SV=2 (MYL6_HUMAN) | MYL6 [2] | 17 | 0.82 | 100% | 100% | 100% | 100% | 100% | 100% | 100% | 100% | 100% | 100% | 100% | 100% |
| 1027 | Secretory carrier-associated membrane protein 3 OS=Homo sapiens GN=SCAMP3 PE=1 SV=3 | SCAM3 | 38 | 0.026 | 100% | 100% | 100% | 100% | 100% | 100% | 100% | 100% | 100% | 100% | 100% | 100% |
| 1028 | Signal recognition particle receptor subunit beta OS=Homo sapiens GN=SRPRB PE=1 SV=3 | SRPRB | 30 | 0.12 | 100% | 100% | 100% | 100% | 100% | 100% | 100% | 100% | 100% | 100% | 100% | 100% |
| 1029 | Alpha-1,3-mannosyl-glycoprotein 2-beta-N-acetylglucosaminyltransferase OS=Homo sapiens GN=MGAT1 PE=2 SV=2 | MGAT1 | 51 | 0.0029 | 100% | 100% | 100% | 100% | 100% | 100% | 100% | 100% | 100% | 100% | 100% | 100% |
| 1030 | Membrane-associated progesterone receptor component 2 OS=Homo sapiens GN=PGRMC2 PE=1 SV=1 | PGRC2 | 24 | 0.071 | 100% | 100% | 100% | 100% | 100% | 100% | 100% | 100% | 100% | 100% | 100% | 100% |
| 1031 | 60S ribosomal protein L27a OS=Homo sapiens GN=RPL27A PE=1 SV=2 | RL27A | 17 | 0.64 | 100% | 100% | 100% | 100% | 100% | 100% | 100% | 100% | 100% | 100% | 100% | 100% |
| 1032 | Annexin A11 OS=Homo sapiens GN=ANXA11 PE=1 SV=1 | ANX11 | 54 | 0.00048 | 100% | 100% | 100% | 100% | 100% | 100% | 100% | 100% | 100% | 100% | 100% | 100% |
| 1033 | Golgin subfamily A member 5 OS=Homo sapiens GN=GOLGA5 PE=1 SV=3 | GOGA5 | 83 | 0.2 | 100% | 100% | 100% | 100% | 100% | 100% | 100% | 100% | 100% | 100% | 100% | 100% |
| 1034 | Fragile X mental retardation syndrome-related protein 1 OS=Homo sapiens GN=FXR1 PE=1 SV=3 | FXR1 | 70 | 0.0052 | 100% | 100% | 100% | 100% | 100% | 100% | 100% | 100% | 100% | 100% | 100% | 100% |
| 1035 | Inositol monophosphatase 3 OS=Homo sapiens GN=IMPAD1 PE=1 SV=1 | IMPA3 | 39 | 0.089 | 100% | 100% | 100% | 100% | 100% | 100% | 100% | 100% | 100% | 100% | 100% | 100% |
| 1036 | Myb-binding protein 1A OS=Homo sapiens GN=MYBBP1A PE=1 SV=2 | MBB1A | 149 | 0.00047 | 100% | 100% | 100% | 100% | 100% | 100% | 100% | 100% | 100% | 100% | 100% | 100% |
| 1037 | Thioredoxin domain-containing protein 5 OS=Homo sapiens GN=TXNDC5 PE=1 SV=2 | TXND5 | 48 | 0.38 | 100% | 100% | 100% | 100% | 100% | 100% | 100% | 100% | 100% | 100% | 100% | 100% |
| 1038 | Transformer-2 protein homolog beta OS=Homo sapiens GN=TRA2B PE=1 SV=1 | TRA2B | 34 | 0.085 | 100% | 100% | 100% | 100% | 100% | 100% | 100% | 100% | 100% | 100% | 100% | 100% |
| 1039 | BAG family molecular chaperone regulator 2 OS=Homo sapiens GN=BAG2 PE=1 SV=1 | BAG2 | 24 | 0.054 | 100% | 100% | 100% | 100% | 100% | 100% | 100% | 100% | 100% | 100% | 100% | 100% |
| 1040 | Erlin-1 OS=Homo sapiens GN=ERLIN1 PE=1 SV=1 | ERLN1 | 39 | 0.0028 | 100% | 100% | 100% | 100% | 100% | 100% | 100% | 100% | 100% | 100% | 100% | 100% |
| 1041 | Cluster of Heat shock 70 kDa protein 1A/1B OS=Homo sapiens GN=HSPA1A PE=1 SV=5 (HSP71_HUMAN) | HSP71 [4] | 70 | 0.0029 | 100% | 100% | 100% | 100% | 100% | 100% | 100% | 100% | 100% | 100% | 100% | 100% |
| 1042 | NAD(P) transhydrogenase, mitochondrial OS=Homo sapiens GN=NNT PE=1 SV=3 | NNTM | 114 | < 0.00010 | 100% | 100% | 100% | 100% | 100% | 100% | 0 | 95% | 87% | 100% | 100% | 100% |
| 1043 | Serine/arginine-rich splicing factor 1 OS=Homo sapiens GN=SRSF1 PE=1 SV=2 | SRSF1 | 28 | 0.04 | 100% | 100% | 100% | 100% | 100% | 100% | 100% | 100% | 100% | 100% | 100% | 100% |
| 1044 | Transmembrane 9 superfamily member 3 OS=Homo sapiens GN=TM9SF3 PE=1 SV=2 | TM9S3 | 68 | 0.037 | 100% | 100% | 100% | 100% | 100% | 100% | 100% | 100% | 100% | 100% | 100% | 100% |
| 1045 | Protein FAM3C OS=Homo sapiens GN=FAM3C PE=1 SV=1 | FAM3C | 25 | 0.024 | 100% | 100% | 100% | 100% | 100% | 100% | 100% | 100% | 100% | 100% | 100% | 100% |
| 1046 | Antigen peptide transporter 1 OS=Homo sapiens GN=TAP1 PE=1 SV=2 | TAP1 | 87 | 0.00083 | 100% | 100% | 100% | 100% | 100% | 100% | 100% | 100% | 100% | 100% | 100% | 100% |
| 1047 | Tapasin OS=Homo sapiens GN=TAPBP PE=1 SV=1 | TPSN | 48 | 0.042 | 100% | 100% | 100% | 100% | 100% | 100% | 100% | 100% | 100% | 100% | 100% | 100% |
| 1048 | Cathepsin B OS=Homo sapiens GN=CTSB PE=1 SV=3 | CATB | 38 | 0.001 | 100% | 100% | 100% | 100% | 100% | 100% | 100% | 100% | 100% | 100% | 100% | 100% |
| 1049 | Phosphoglycerate kinase 1 OS=Homo sapiens GN=PGK1 PE=1 SV=3 | PGK1 | 45 | 0.00022 | 100% | 100% | 100% | 88% | 100% | 100% | 100% | 100% | 100% | 100% | 100% | 100% |
| 1050 | UDP-glucose:glycoprotein glucosyltransferase 1 OS=Homo sapiens GN=UGGT1 PE=1 SV=3 | UGGG1 | 177 | 0.012 | 100% | 100% | 100% | 100% | 100% | 100% | 100% | 100% | 100% | 100% | 100% | 100% |
| 1051 | FAS-associated factor 2 OS=Homo sapiens GN=FAF2 PE=1 SV=2 | FAF2 | 53 | 0.058 | 100% | 100% | 100% | 100% | 100% | 100% | 100% | 100% | 100% | 100% | 100% | 100% |
| 1052 | Golgi-specific brefeldin A-resistance guanine nucleotide exchange factor 1 OS=Homo sapiens GN=GBF1 PE=1 SV=2 | GBF1 | 206 | 0.021 | 100% | 100% | 100% | 100% | 100% | 100% | 100% | 100% | 100% | 100% | 100% | 100% |
| 1053 | Heterogeneous nuclear ribonucleoprotein A/B OS=Homo sapiens GN=HNRNPAB PE=1 SV=2 | ROAA | 36 | 0.0054 | 100% | 100% | 100% | 100% | 100% | 100% | 100% | 100% | 100% | 100% | 100% | 100% |
| 1054 | Cluster of Hexokinase-1 OS=Homo sapiens GN=HK1 PE=1 SV=3 (HXK1_HUMAN) | HXK1 [4] | 102 | < 0.00010 | 100% | 100% | 100% | 100% | 100% | 100% | 0 | 100% | 31% | 100% | 100% | 100% |
| 1055 | Transmembrane 9 superfamily member 4 OS=Homo sapiens GN=TM9SF4 PE=1 SV=2 | TM9S4 | 75 | 0.015 | 100% | 100% | 100% | 100% | 100% | 100% | 100% | 100% | 100% | 100% | 100% | 100% |
| 1056 | Calumenin OS=Homo sapiens GN=CALU PE=1 SV=2 | CALU | 37 | 0.15 | 100% | 100% | 100% | 100% | 100% | 100% | 100% | 100% | 100% | 100% | 100% | 100% |
| 1057 | 60S ribosomal protein L35a OS=Homo sapiens GN=RPL35A PE=1 SV=2 | RL35A | 13 | 0.0085 | 100% | 100% | 100% | 100% | 100% | 100% | 100% | 100% | 100% | 100% | 100% | 100% |
| 1058 | Cluster of 2-oxoglutarate dehydrogenase, mitochondrial OS=Homo sapiens GN=OGDH PE=1 SV=3 (ODO1_HUMAN) | ODO1 [2] | 116 | 0.019 | 71% | 100% | 100% | 100% | 100% | 100% | 100% | 100% | 100% | 100% | 100% | 100% |
| 1059 | Golgin subfamily B member 1 OS=Homo sapiens GN=GOLGB1 PE=1 SV=2 | GOGB1 | 376 | 0.13 | 100% | 100% | 100% | 100% | 100% | 100% | 100% | 100% | 100% | 100% | 100% | 100% |
| 1060 | Heterogeneous nuclear ribonucleoprotein D-like OS=Homo sapiens GN=HNRNPDL PE=1 SV=3 | HNRDL | 46 | 0.024 | 100% | 100% | 100% | 100% | 100% | 100% | 100% | 100% | 100% | 100% | 100% | 100% |
| 1061 | Thyroid hormone receptor-associated protein 3 OS=Homo sapiens GN=THRAP3 PE=1 SV=2 | TR150 | 109 | 0.0053 | 100% | 100% | 100% | 100% | 100% | 100% | 100% | 100% | 100% | 100% | 100% | 100% |
| 1062 | Neuroblastoma-amplified sequence OS=Homo sapiens GN=NBAS PE=1 SV=2 | NBAS | 269 | 0.86 | 100% | 100% | 100% | 100% | 100% | 100% | 100% | 100% | 100% | 100% | 100% | 100% |
| 1063 | Serine/arginine-rich splicing factor 7 OS=Homo sapiens GN=SRSF7 PE=1 SV=1 | SRSF7 | 27 | 0.33 | 100% | 100% | 100% | 100% | 100% | 100% | 100% | 100% | 100% | 100% | 100% | 100% |
| 1064 | Coatomer subunit alpha OS=Homo sapiens GN=COPA PE=1 SV=2 | COPA | 138 | 0.17 | 100% | 100% | 100% | 100% | 100% | 100% | 100% | 100% | 100% | 100% | 100% | 100% |
| 1065 | CAAX prenyl protease 1 homolog OS=Homo sapiens GN=ZMPSTE24 PE=1 SV=2 | FACE1 | 55 | 0.13 | 100% | 100% | 100% | 100% | 100% | 100% | 100% | 100% | 100% | 100% | 100% | 100% |
| 1066 | Neuropathy target esterase OS=Homo sapiens GN=PNPLA6 PE=1 SV=2 | PLPL6 | 150 | 0.087 | 100% | 100% | 100% | 100% | 100% | 100% | 100% | 100% | 100% | 100% | 100% | 100% |
| 1067 | T-complex protein 1 subunit zeta OS=Homo sapiens GN=CCT6A PE=1 SV=3 | TCPZ | 58 | 0.63 | 100% | 100% | 100% | 100% | 100% | 100% | 100% | 100% | 100% | 100% | 100% | 100% |
| 1068 | Sterol-4-alpha-carboxylate 3-dehydrogenase, decarboxylating OS=Homo sapiens GN=NSDHL PE=1 SV=2 | NSDHL | 42 | 0.088 | 100% | 100% | 100% | 100% | 100% | 100% | 100% | 100% | 100% | 100% | 100% | 100% |
| 1069 | 60S ribosomal protein L11 OS=Homo sapiens GN=RPL11 PE=1 SV=2 | RL11 | 20 | 0.006 | 100% | 100% | 100% | 100% | 100% | 100% | 100% | 100% | 100% | 100% | 100% | 100% |
| 1070 | DDRGK domain-containing protein 1 OS=Homo sapiens GN=DDRGK1 PE=1 SV=2 | DDRGK | 36 | 0.21 | 100% | 100% | 100% | 100% | 100% | 100% | 100% | 100% | 100% | 100% | 100% | 100% |
| 1071 | Peroxisomal multifunctional enzyme type 2 OS=Homo sapiens GN=HSD17B4 PE=1 SV=3 | DHB4 | 80 | 0.0037 | 100% | 100% | 100% | 100% | 100% | 100% | 100% | 100% | 100% | 100% | 100% | 100% |
| 1072 | Adenosine 3'-phospho 5'-phosphosulfate transporter 1 OS=Homo sapiens GN=SLC35B2 PE=1 SV=1 | S35B2 | 48 | 0.44 | 100% | 100% | 100% | 100% | 100% | 100% | 100% | 100% | 100% | 100% | 100% | 100% |
| 1073 | Serine palmitoyltransferase 1 OS=Homo sapiens GN=SPTLC1 PE=1 SV=1 | SPTC1 | 53 | 0.52 | 100% | 100% | 100% | 100% | 100% | 100% | 100% | 100% | 100% | 100% | 100% | 100% |
| 1074 | Splicing factor 3A subunit 3 OS=Homo sapiens GN=SF3A3 PE=1 SV=1 | SF3A3 | 59 | 0.037 | 100% | 100% | 100% | 100% | 100% | 100% | 100% | 100% | 100% | 100% | 100% | 100% |
| 1075 | Thioredoxin-related transmembrane protein 1 OS=Homo sapiens GN=TMX1 PE=1 SV=1 | TMX1 | 32 | 0.76 | 100% | 100% | 100% | 100% | 100% | 100% | 100% | 100% | 100% | 100% | 100% | 100% |
| 1076 | Eukaryotic translation initiation factor 3 subunit C OS=Homo sapiens GN=EIF3C PE=1 SV=1 | EIF3C (+1) | 105 | 0.53 | 100% | 100% | 100% | 100% | 100% | 100% | 100% | 100% | 100% | 100% | 100% | 100% |
| 1077 | Vesicle transport protein GOT1B OS=Homo sapiens GN=GOLT1B PE=1 SV=1 | GOT1B | 15 | 0.99 | 100% | 100% | 100% | 100% | 100% | 100% | 100% | 100% | 100% | 100% | 100% | 100% |
| 1078 | Inhibitor of nuclear factor kappa-B kinase-interacting protein OS=Homo sapiens GN=IKBIP PE=1 SV=1 | IKIP | 39 | 0.17 | 100% | 100% | 100% | 100% | 100% | 100% | 100% | 100% | 100% | 100% | 100% | 100% |
| 1079 | Trifunctional enzyme subunit beta, mitochondrial OS=Homo sapiens GN=HADHB PE=1 SV=3 | ECHB | 51 | 0.017 | 100% | 100% | 100% | 100% | 100% | 100% | 100% | 100% | 100% | 100% | 100% | 100% |
| 1080 | 60S ribosomal protein L32 OS=Homo sapiens GN=RPL32 PE=1 SV=2 | RL32 | 16 | 0.027 | 100% | 100% | 100% | 100% | 100% | 100% | 100% | 100% | 100% | 100% | 100% | 100% |
| 1081 | Translocon-associated protein subunit delta OS=Homo sapiens GN=SSR4 PE=1 SV=1 | SSRD | 19 | 0.86 | 100% | 100% | 100% | 100% | 100% | 100% | 100% | 100% | 100% | 100% | 100% | 100% |
| 1082 | Nuclease-sensitive element-binding protein 1 OS=Homo sapiens GN=YBX1 PE=1 SV=3 | YBOX1 | 36 | 0.11 | 100% | 100% | 100% | 100% | 100% | 100% | 100% | 100% | 100% | 100% | 100% | 100% |
| 1083 | Probable ATP-dependent RNA helicase DDX23 OS=Homo sapiens GN=DDX23 PE=1 SV=3 | DDX23 | 96 | 0.74 | 100% | 100% | 100% | 100% | 100% | 100% | 100% | 100% | 100% | 100% | 100% | 100% |
| 1084 | Elongation factor Tu, mitochondrial OS=Homo sapiens GN=TUFM PE=1 SV=2 | EFTU | 50 | 0.0023 | 100% | 100% | 100% | 100% | 100% | 100% | 100% | 100% | 100% | 100% | 100% | 100% |
| 1085 | Heterochromatin protein 1-binding protein 3 OS=Homo sapiens GN=HP1BP3 PE=1 SV=1 | HP1B3 | 61 | 0.096 | 100% | 100% | 100% | 100% | 100% | 100% | 100% | 100% | 100% | 100% | 100% | 100% |
| 1086 | 40S ribosomal protein S17-like OS=Homo sapiens GN=RPS17L PE=1 SV=1 | RS17L (+1) | 16 | 0.048 | 100% | 100% | 100% | 100% | 100% | 100% | 100% | 100% | 100% | 100% | 100% | 100% |
| 1087 | Signal peptidase complex subunit 2 OS=Homo sapiens GN=SPCS2 PE=1 SV=3 | SPCS2 | 25 | 0.092 | 100% | 100% | 100% | 100% | 100% | 100% | 100% | 100% | 100% | 100% | 100% | 100% |
| 1088 | Procollagen galactosyltransferase 1 OS=Homo sapiens GN=COLGALT1 PE=1 SV=1 | GT251 | 72 | 0.8 | 100% | 100% | 100% | 100% | 100% | 100% | 100% | 100% | 100% | 100% | 100% | 100% |
| 1089 | Titin OS=Homo sapiens GN=TTN PE=1 SV=4 | TITIN | 3816 | 0.25 | 100% | 95% | 100% | 100% | 96% | 100% | 100% | 99% | 100% | 43% | 93% | 100% |
| 1090 | Endoplasmic reticulum resident protein 29 OS=Homo sapiens GN=ERP29 PE=1 SV=4 | ERP29 | 29 | 0.00044 | 100% | 100% | 100% | 100% | 100% | 100% | 100% | 100% | 100% | 100% | 100% | 100% |
| 1091 | Probable ATP-dependent RNA helicase DDX17 OS=Homo sapiens GN=DDX17 PE=1 SV=2 | DDX17 | 80 | 0.0092 | 99% | 100% | 100% | 100% | 100% | 100% | 100% | 100% | 100% | 100% | 100% | 100% |
| 1092 | Regulator of nonsense transcripts 1 OS=Homo sapiens GN=UPF1 PE=1 SV=2 | RENT1 | 124 | 0.13 | 100% | 100% | 100% | 100% | 100% | 100% | 100% | 100% | 100% | 100% | 100% | 100% |
| 1093 | 40S ribosomal protein S15a OS=Homo sapiens GN=RPS15A PE=1 SV=2 | RS15A | 15 | 0.027 | 100% | 100% | 100% | 100% | 100% | 100% | 100% | 100% | 100% | 100% | 100% | 100% |
| 1094 | 60S ribosomal protein L35 OS=Homo sapiens GN=RPL35 PE=1 SV=2 | RL35 | 15 | 0.048 | 100% | 100% | 100% | 100% | 100% | 100% | 100% | 100% | 100% | 100% | 100% | 100% |
| 1095 | Protein transport protein Sec24C OS=Homo sapiens GN=SEC24C PE=1 SV=3 | SC24C | 118 | 0.17 | 100% | 100% | 100% | 100% | 100% | 100% | 100% | 100% | 100% | 100% | 100% | 100% |
| 1096 | Translocation protein SEC63 homolog OS=Homo sapiens GN=SEC63 PE=1 SV=2 | SEC63 | 88 | 0.76 | 100% | 100% | 100% | 100% | 100% | 100% | 100% | 100% | 100% | 100% | 100% | 100% |
| 1097 | Cell division cycle 5-like protein OS=Homo sapiens GN=CDC5L PE=1 SV=2 | CDC5L | 92 | 0.026 | 95% | 100% | 100% | 100% | 100% | 100% | 100% | 100% | 100% | 100% | 100% | 100% |
| 1098 | UBX domain-containing protein 4 OS=Homo sapiens GN=UBXN4 PE=1 SV=2 | UBXN4 | 57 | 0.11 | 100% | 100% | 100% | 100% | 100% | 100% | 100% | 100% | 100% | 100% | 100% | 100% |
| 1099 | Fatty acid desaturase 3 OS=Homo sapiens GN=FADS3 PE=2 SV=1 | FADS3 | 51 | 0.034 | 100% | 100% | 100% | 100% | 100% | 100% | 100% | 100% | 100% | 100% | 100% | 100% |
| 1100 | Lamin-B2 OS=Homo sapiens GN=LMNB2 PE=1 SV=3 | LMNB2 | 68 | 0.098 | 100% | 100% | 100% | 100% | 100% | 100% | 100% | 100% | 100% | 100% | 100% | 100% |
| 1101 | Poly [ADP-ribose] polymerase 4 OS=Homo sapiens GN=PARP4 PE=1 SV=3 | PARP4 | 193 | 0.0012 | 100% | 100% | 100% | 100% | 95% | 95% | 100% | 100% | 100% | 100% | 100% | 100% |
| 1102 | Triosephosphate isomerase OS=Homo sapiens GN=TPI1 PE=1 SV=3 | TPIS | 31 | 0.0076 | 100% | 100% | 100% | 100% | 100% | 95% | 100% | 100% | 100% | 100% | 100% | 100% |
| 1103 | Probable glutathione peroxidase 8 OS=Homo sapiens GN=GPX8 PE=1 SV=2 | GPX8 | 24 | 0.26 | 100% | 100% | 100% | 100% | 100% | 100% | 100% | 100% | 100% | 100% | 100% | 100% |
| 1104 | Very-long-chain enoyl-CoA reductase OS=Homo sapiens GN=TECR PE=1 SV=1 | TECR | 36 | 0.014 | 100% | 100% | 100% | 100% | 100% | 100% | 100% | 100% | 86% | 100% | 100% | 100% |
| 1105 | Lipase maturation factor 2 OS=Homo sapiens GN=LMF2 PE=1 SV=2 | LMF2 | 80 | 0.0047 | 100% | 100% | 100% | 100% | 100% | 100% | 100% | 100% | 100% | 100% | 100% | 100% |
| 1106 | Cluster of Serine/arginine-rich splicing factor 6 OS=Homo sapiens GN=SRSF6 PE=1 SV=2 (SRSF6_HUMAN) | SRSF6 [2] | 40 | 0.49 | 100% | 100% | 100% | 100% | 100% | 100% | 100% | 100% | 100% | 100% | 100% | 100% |
| 1107 | Peptidyl-prolyl cis-trans isomerase FKBP8 OS=Homo sapiens GN=FKBP8 PE=1 SV=2 | FKBP8 | 45 | 0.021 | 100% | 100% | 100% | 100% | 100% | 100% | 100% | 100% | 100% | 100% | 100% | 100% |
| 1108 | Retinol dehydrogenase 11 OS=Homo sapiens GN=RDH11 PE=1 SV=2 | RDH11 | 35 | 0.45 | 100% | 100% | 100% | 100% | 100% | 100% | 100% | 100% | 100% | 100% | 100% | 100% |
| 1109 | Bcl-2-associated transcription factor 1 OS=Homo sapiens GN=BCLAF1 PE=1 SV=2 | BCLF1 | 106 | 0.79 | 99% | 100% | 100% | 94% | 100% | 100% | 100% | 100% | 100% | 100% | 100% | 100% |
| 1110 | Eukaryotic translation initiation factor 2 subunit 1 OS=Homo sapiens GN=EIF2S1 PE=1 SV=3 | IF2A | 36 | 0.18 | 100% | 100% | 100% | 100% | 100% | 100% | 100% | 100% | 100% | 100% | 100% | 100% |
| 1111 | Putative ribosomal RNA methyltransferase NOP2 OS=Homo sapiens GN=NOP2 PE=1 SV=2 | NOP2 | 89 | 0.0003 | 100% | 100% | 100% | 100% | 100% | 100% | 100% | 100% | 100% | 100% | 100% | 100% |
| 1112 | Dolichol-phosphate mannosyltransferase subunit 1 OS=Homo sapiens GN=DPM1 PE=1 SV=1 | DPM1 | 30 | 0.4 | 100% | 100% | 100% | 100% | 100% | 100% | 100% | 100% | 100% | 100% | 100% | 100% |
| 1113 | Far upstream element-binding protein 2 OS=Homo sapiens GN=KHSRP PE=1 SV=4 | FUBP2 | 73 | 0.14 | 100% | 100% | 100% | 100% | 100% | 100% | 100% | 100% | 100% | 100% | 100% | 100% |
| 1114 | Endoplasmic reticulum mannosyl-oligosaccharide 1,2-alpha-mannosidase OS=Homo sapiens GN=MAN1B1 PE=1 SV=2 | MA1B1 | 80 | 0.0049 | 100% | 100% | 100% | 100% | 100% | 100% | 100% | 100% | 100% | 100% | 100% | 100% |
| 1115 | Golgi SNAP receptor complex member 2 OS=Homo sapiens GN=GOSR2 PE=1 SV=2 | GOSR2 | 25 | 0.092 | 100% | 100% | 100% | 100% | 100% | 100% | 100% | 100% | 100% | 100% | 100% | 100% |
| 1116 | Uncharacterized protein KIAA2013 OS=Homo sapiens GN=KIAA2013 PE=2 SV=1 | K2013 | 69 | 0.0079 | 100% | 100% | 100% | 100% | 100% | 100% | 100% | 100% | 79% | 100% | 100% | 100% |
| 1117 | Glycerol-3-phosphate dehydrogenase, mitochondrial OS=Homo sapiens GN=GPD2 PE=1 SV=3 | GPDM | 81 | < 0.00010 | 100% | 100% | 100% | 100% | 100% | 100% | 98% | 98% | 0 | 100% | 100% | 100% |
| 1118 | Zinc finger protein-like 1 OS=Homo sapiens GN=ZFPL1 PE=1 SV=2 | ZFPL1 | 34 | 0.23 | 100% | 100% | 100% | 100% | 100% | 100% | 100% | 100% | 100% | 100% | 100% | 100% |
| 1119 | Cluster of RNA-binding protein 39 OS=Homo sapiens GN=RBM39 PE=1 SV=2 (RBM39_HUMAN) | RBM39 [2] | 59 | 0.0021 | 100% | 100% | 100% | 100% | 100% | 100% | 100% | 100% | 100% | 100% | 100% | 100% |
| 1120 | Far upstream element-binding protein 3 OS=Homo sapiens GN=FUBP3 PE=1 SV=2 | FUBP3 | 62 | 0.021 | 96% | 100% | 100% | 97% | 100% | 100% | 100% | 100% | 100% | 100% | 100% | 100% |
| 1121 | Lysosomal acid phosphatase OS=Homo sapiens GN=ACP2 PE=1 SV=3 | PPAL | 48 | 0.00042 | 100% | 100% | 100% | 100% | 100% | 100% | 100% | 100% | 100% | 100% | 100% | 100% |
| 1122 | 40S ribosomal protein S24 OS=Homo sapiens GN=RPS24 PE=1 SV=1 | RS24 | 15 | 0.021 | 100% | 100% | 100% | 100% | 100% | 100% | 100% | 100% | 100% | 100% | 100% | 100% |
| 1123 | Collagen alpha-1(III) chain OS=Homo sapiens GN=COL3A1 PE=1 SV=4 | CO3A1 | 139 | 0.11 | 100% | 100% | 100% | 100% | 100% | 100% | 100% | 100% | 100% | 100% | 100% | 100% |
| 1124 | Core histone macro-H2A.1 OS=Homo sapiens GN=H2AFY PE=1 SV=4 | H2AY | 40 | 0.012 | 100% | 100% | 100% | 100% | 100% | 100% | 100% | 100% | 100% | 100% | 100% | 100% |
| 1125 | GPI transamidase component PIG-S OS=Homo sapiens GN=PIGS PE=1 SV=3 | PIGS | 62 | 0.14 | 100% | 100% | 100% | 100% | 100% | 100% | 100% | 100% | 100% | 100% | 100% | 100% |
| 1126 | Fragile X mental retardation syndrome-related protein 2 OS=Homo sapiens GN=FXR2 PE=1 SV=2 | FXR2 | 74 | 0.14 | 100% | 100% | 100% | 100% | 100% | 100% | 100% | 100% | 100% | 100% | 100% | 100% |
| 1127 | Prolyl 4-hydroxylase subunit alpha-2 OS=Homo sapiens GN=P4HA2 PE=1 SV=1 | P4HA2 | 61 | 0.2 | 100% | 100% | 100% | 100% | 100% | 100% | 100% | 100% | 100% | 100% | 100% | 100% |
| 1128 | Protein disulfide-isomerase A5 OS=Homo sapiens GN=PDIA5 PE=1 SV=1 | PDIA5 | 60 | 0.55 | 100% | 100% | 100% | 100% | 100% | 100% | 100% | 100% | 100% | 100% | 100% | 100% |
| 1129 | Beta-hexosaminidase subunit beta OS=Homo sapiens GN=HEXB PE=1 SV=3 | HEXB | 63 | < 0.00010 | 100% | 100% | 100% | 95% | 100% | 100% | 100% | 95% | 95% | 100% | 100% | 100% |
| 1130 | Pre-B-cell leukemia transcription factor-interacting protein 1 OS=Homo sapiens GN=PBXIP1 PE=1 SV=1 | PBIP1 | 81 | 0.0023 | 100% | 100% | 100% | 95% | 100% | 100% | 100% | 100% | 100% | 100% | 100% | 100% |
| 1131 | Dolichyl-diphosphooligosaccharide--protein glycosyltransferase subunit STT3B OS=Homo sapiens GN=STT3B PE=1 SV=1 | STT3B | 94 | 0.0025 | 100% | 100% | 100% | 100% | 100% | 100% | 99% | 100% | 84% | 100% | 100% | 100% |
| 1132 | Heterogeneous nuclear ribonucleoprotein F OS=Homo sapiens GN=HNRNPF PE=1 SV=3 | HNRPF | 46 | 0.3 | 100% | 100% | 100% | 100% | 100% | 100% | 100% | 100% | 100% | 100% | 100% | 100% |
| 1133 | Poliovirus receptor-related protein 2 OS=Homo sapiens GN=PVRL2 PE=1 SV=1 | PVRL2 | 58 | 0.12 | 100% | 100% | 100% | 100% | 100% | 100% | 100% | 100% | 100% | 100% | 100% | 100% |
| 1134 | Antigen peptide transporter 2 OS=Homo sapiens GN=TAP2 PE=1 SV=1 | TAP2 | 76 | 0.001 | 100% | 100% | 100% | 100% | 100% | 100% | 100% | 100% | 100% | 100% | 100% | 100% |
| 1135 | Torsin-1A-interacting protein 2 OS=Homo sapiens GN=TOR1AIP2 PE=1 SV=1 | TOIP2 | 51 | 0.12 | 95% | 100% | 100% | 100% | 100% | 100% | 100% | 100% | 100% | 100% | 100% | 100% |
| 1136 | Long-chain-fatty-acid--CoA ligase 4 OS=Homo sapiens GN=ACSL4 PE=1 SV=2 | ACSL4 | 79 | 0.51 | 100% | 100% | 100% | 100% | 100% | 100% | 100% | 100% | 100% | 100% | 100% | 100% |
| 1137 | Chondroitin sulfate synthase 2 OS=Homo sapiens GN=CHPF PE=1 SV=2 | CHSS2 | 85 | 0.057 | 0 | 100% | 100% | 100% | 100% | 100% | 100% | 100% | 100% | 100% | 100% | 100% |
| 1138 | Serine/arginine-rich splicing factor 9 OS=Homo sapiens GN=SRSF9 PE=1 SV=1 | SRSF9 | 26 | 0.51 | 96% | 100% | 100% | 100% | 100% | 100% | 100% | 100% | 100% | 100% | 100% | 100% |
| 1139 | Carnitine O-palmitoyltransferase 1, liver isoform OS=Homo sapiens GN=CPT1A PE=1 SV=2 | CPT1A | 88 | 0.16 | 100% | 100% | 100% | 100% | 100% | 100% | 100% | 100% | 100% | 100% | 100% | 100% |
| 1140 | CDK5 regulatory subunit-associated protein 3 OS=Homo sapiens GN=CDK5RAP3 PE=1 SV=2 | CK5P3 | 57 | 0.99 | 0 | 100% | 100% | 100% | 100% | 100% | 100% | 100% | 100% | 100% | 100% | 100% |
| 1141 | Laminin subunit gamma-1 OS=Homo sapiens GN=LAMC1 PE=1 SV=3 | LAMC1 | 178 | 0.29 | 100% | 100% | 100% | 100% | 100% | 100% | 100% | 100% | 100% | 100% | 100% | 100% |
| 1142 | U2 small nuclear ribonucleoprotein A' OS=Homo sapiens GN=SNRPA1 PE=1 SV=2 | RU2A | 28 | 0.001 | 100% | 100% | 100% | 100% | 100% | 100% | 100% | 100% | 100% | 100% | 100% | 100% |
| 1143 | SUN domain-containing protein 2 OS=Homo sapiens GN=SUN2 PE=1 SV=3 | SUN2 | 80 | 0.039 | 99% | 100% | 100% | 100% | 100% | 100% | 95% | 99% | 100% | 100% | 100% | 100% |
| 1144 | Translocating chain-associated membrane protein 1 OS=Homo sapiens GN=TRAM1 PE=1 SV=3 | TRAM1 | 43 | 0.075 | 100% | 100% | 100% | 80% | 100% | 100% | 0 | 100% | 100% | 100% | 100% | 100% |
| 1145 | Splicing factor U2AF 65 kDa subunit OS=Homo sapiens GN=U2AF2 PE=1 SV=4 | U2AF2 | 54 | 0.0014 | 100% | 100% | 100% | 100% | 100% | 100% | 100% | 100% | 100% | 100% | 100% | 100% |
| 1146 | Cytochrome P450 1B1 OS=Homo sapiens GN=CYP1B1 PE=1 SV=2 | CP1B1 | 61 | 0.2 | 100% | 100% | 100% | 100% | 100% | 100% | 100% | 100% | 100% | 100% | 100% | 100% |
| 1147 | Eukaryotic translation initiation factor 3 subunit F OS=Homo sapiens GN=EIF3F PE=1 SV=1 | EIF3F | 38 | 0.24 | 100% | 100% | 100% | 100% | 100% | 100% | 100% | 100% | 100% | 100% | 100% | 100% |
| 1148 | N-acetylgalactosaminyltransferase 7 OS=Homo sapiens GN=GALNT7 PE=1 SV=1 | GALT7 | 75 | 0.018 | 0 | 57% | 100% | 100% | 100% | 100% | 94% | 14% | 47% | 100% | 100% | 100% |
| 1149 | U2 snRNP-associated SURP motif-containing protein OS=Homo sapiens GN=U2SURP PE=1 SV=2 | SR140 | 118 | 0.22 | 100% | 100% | 100% | 100% | 100% | 100% | 100% | 100% | 100% | 100% | 100% | 100% |
| 1150 | Transmembrane emp24 domain-containing protein 4 OS=Homo sapiens GN=TMED4 PE=1 SV=1 | TMED4 | 26 | 0.14 | 100% | 100% | 100% | 100% | 100% | 100% | 100% | 100% | 100% | 100% | 100% | 100% |
| 1151 | Vesicle transport through interaction with t-SNAREs homolog 1B OS=Homo sapiens GN=VTI1B PE=1 SV=3 | VTI1B | 27 | 0.45 | 99% | 100% | 100% | 100% | 100% | 100% | 100% | 100% | 100% | 100% | 100% | 100% |
| 1152 | Fibronectin type III domain-containing protein 3B OS=Homo sapiens GN=FNDC3B PE=1 SV=2 | FND3B | 133 | 0.0041 | 95% | 100% | 100% | 100% | 100% | 100% | 100% | 100% | 100% | 100% | 100% | 100% |
| 1153 | Translational activator GCN1 OS=Homo sapiens GN=GCN1L1 PE=1 SV=6 | GCN1L | 293 | 0.021 | 100% | 100% | 100% | 100% | 100% | 100% | 100% | 100% | 100% | 100% | 100% | 100% |
| 1154 | Nucleolar GTP-binding protein 1 OS=Homo sapiens GN=GTPBP4 PE=1 SV=3 | NOG1 | 74 | 0.068 | 0 | 100% | 100% | 95% | 100% | 100% | 100% | 100% | 100% | 100% | 100% | 100% |
| 1155 | Cluster of Coatomer subunit gamma-1 OS=Homo sapiens GN=COPG1 PE=1 SV=1 (COPG1_HUMAN) | COPG1 [2] | 98 | 0.0031 | 100% | 98% | 100% | 100% | 100% | 100% | 100% | 100% | 100% | 100% | 100% | 100% |
| 1156 | UPF0568 protein C14orf166 OS=Homo sapiens GN=C14orf166 PE=1 SV=1 | CN166 | 28 | 0.032 | 100% | 100% | 100% | 100% | 100% | 100% | 100% | 100% | 100% | 100% | 100% | 100% |
| 1157 | LETM1 and EF-hand domain-containing protein 1, mitochondrial OS=Homo sapiens GN=LETM1 PE=1 SV=1 | LETM1 | 83 | 0.0066 | 6% | 100% | 100% | 95% | 100% | 100% | 0 | 14% | 75% | 100% | 100% | 100% |
| 1158 | Serine/arginine-rich splicing factor 10 OS=Homo sapiens GN=SRSF10 PE=1 SV=1 | SRS10 | 31 | 0.2 | 100% | 100% | 100% | 100% | 100% | 100% | 100% | 100% | 100% | 100% | 100% | 100% |
| 1159 | Malectin OS=Homo sapiens GN=MLEC PE=1 SV=1 | MLEC | 32 | 0.14 | 100% | 100% | 100% | 100% | 100% | 100% | 100% | 100% | 95% | 100% | 100% | 100% |
| 1160 | Mitochondrial carrier homolog 2 OS=Homo sapiens GN=MTCH2 PE=1 SV=1 | MTCH2 | 33 | 0.0027 | 100% | 100% | 100% | 100% | 100% | 100% | 100% | 14% | 95% | 100% | 100% | 100% |
| 1161 | Small nuclear ribonucleoprotein Sm D1 OS=Homo sapiens GN=SNRPD1 PE=1 SV=1 | SMD1 | 13 | 0.28 | 100% | 100% | 100% | 100% | 100% | 100% | 100% | 100% | 100% | 100% | 100% | 100% |
| 1162 | Transmembrane and TPR repeat-containing protein 3 OS=Homo sapiens GN=TMTC3 PE=1 SV=2 | TMTC3 | 104 | 0.14 | 100% | 100% | 100% | 100% | 100% | 100% | 100% | 100% | 100% | 100% | 100% | 100% |
| 1163 | Tripeptidyl-peptidase 1 OS=Homo sapiens GN=TPP1 PE=1 SV=2 | TPP1 | 61 | 0.42 | 100% | 100% | 100% | 100% | 100% | 100% | 100% | 100% | 100% | 100% | 100% | 100% |
| 1164 | Apolipoprotein L2 OS=Homo sapiens GN=APOL2 PE=1 SV=1 | APOL2 | 37 | 0.03 | 100% | 100% | 100% | 100% | 100% | 100% | 100% | 100% | 100% | 100% | 100% | 100% |
| 1165 | Mitochondrial inner membrane protein OS=Homo sapiens GN=IMMT PE=1 SV=1 | IMMT | 84 | < 0.00010 | 95% | 100% | 100% | 100% | 100% | 100% | 0 | 0 | 5% | 100% | 100% | 100% |
| 1166 | Lysophosphatidylcholine acyltransferase 2 OS=Homo sapiens GN=LPCAT2 PE=1 SV=1 | PCAT2 | 60 | 0.15 | 100% | 100% | 100% | 100% | 100% | 100% | 100% | 100% | 100% | 100% | 100% | 100% |
| 1167 | Transgelin OS=Homo sapiens GN=TAGLN PE=1 SV=4 | TAGL | 23 | 0.0048 | 100% | 100% | 100% | 97% | 100% | 100% | 100% | 100% | 100% | 100% | 100% | 100% |
| 1168 | Transmembrane protein 109 OS=Homo sapiens GN=TMEM109 PE=1 SV=1 | TM109 | 26 | 0.033 | 100% | 100% | 100% | 100% | 100% | 100% | 100% | 95% | 100% | 100% | 100% | 100% |
| 1169 | Polypeptide N-acetylgalactosaminyltransferase 3 OS=Homo sapiens GN=GALNT3 PE=1 SV=2 | GALT3 | 73 | 0.00032 | 0 | 0 | 0 | 100% | 100% | 100% | 0 | 0 | 0 | 100% | 100% | 100% |
| 1170 | Glucosidase 2 subunit beta OS=Homo sapiens GN=PRKCSH PE=1 SV=2 | GLU2B | 59 | 0.36 | 100% | 100% | 100% | 100% | 100% | 100% | 83% | 100% | 100% | 100% | 100% | 100% |
| 1171 | Guanine nucleotide-binding protein-like 3 OS=Homo sapiens GN=GNL3 PE=1 SV=2 | GNL3 | 62 | 0.035 | 100% | 100% | 100% | 95% | 100% | 100% | 100% | 100% | 100% | 100% | 100% | 100% |
| 1172 | Lysophospholipid acyltransferase 7 OS=Homo sapiens GN=MBOAT7 PE=1 SV=2 | MBOA7 | 53 | 0.036 | 100% | 100% | 100% | 100% | 100% | 100% | 100% | 100% | 94% | 100% | 100% | 100% |
| 1173 | Reticulocalbin-1 OS=Homo sapiens GN=RCN1 PE=1 SV=1 | RCN1 | 39 | 0.12 | 100% | 100% | 100% | 100% | 100% | 100% | 100% | 95% | 100% | 100% | 100% | 100% |
| 1174 | Heterogeneous nuclear ribonucleoprotein A0 OS=Homo sapiens GN=HNRNPA0 PE=1 SV=1 | ROA0 | 31 | 0.22 | 100% | 100% | 100% | 99% | 100% | 97% | 100% | 100% | 100% | 100% | 100% | 100% |
| 1175 | Reticulon-1 OS=Homo sapiens GN=RTN1 PE=1 SV=1 | RTN1 | 84 | < 0.00010 | 0 | 11% | 15% | 100% | 100% | 100% | 11% | 0 | 0 | 100% | 100% | 100% |
| 1176 | Transmembrane protein 214 OS=Homo sapiens GN=TMEM214 PE=1 SV=2 | TM214 | 77 | 0.24 | 100% | 100% | 100% | 100% | 100% | 100% | 100% | 100% | 100% | 100% | 100% | 100% |
| 1177 | Alpha-2-HS-glycoprotein OS=Homo sapiens GN=AHSG PE=1 SV=1 | FETUA | 39 | 0.032 | 100% | 100% | 100% | 99% | 100% | 100% | 100% | 100% | 100% | 100% | 100% | 100% |
| 1178 | DnaJ homolog subfamily C member 10 OS=Homo sapiens GN=DNAJC10 PE=1 SV=2 | DJC10 | 91 | 0.05 | 100% | 100% | 100% | 100% | 100% | 100% | 100% | 100% | 100% | 100% | 100% | 100% |
| 1179 | Peptidyl-prolyl cis-trans isomerase FKBP11 OS=Homo sapiens GN=FKBP11 PE=1 SV=1 | FKB11 | 22 | < 0.00010 | 100% | 100% | 100% | 54% | 13% | 95% | 100% | 100% | 100% | 100% | 100% | 100% |
| 1180 | tRNA-splicing ligase RtcB homolog OS=Homo sapiens GN=RTCB PE=1 SV=1 | RTCB | 55 | 0.0051 | 100% | 100% | 100% | 100% | 100% | 100% | 100% | 100% | 100% | 100% | 100% | 100% |
| 1181 | Sec1 family domain-containing protein 2 OS=Homo sapiens GN=SCFD2 PE=1 SV=2 | SCFD2 | 75 | 0.11 | 100% | 100% | 100% | 100% | 100% | 100% | 100% | 100% | 100% | 100% | 100% | 100% |
| 1182 | Vacuolar protein sorting-associated protein 45 OS=Homo sapiens GN=VPS45 PE=1 SV=1 | VPS45 | 65 | 0.95 | 100% | 100% | 100% | 100% | 100% | 100% | 100% | 100% | 100% | 100% | 100% | 100% |
| 1183 | rRNA 2'-O-methyltransferase fibrillarin OS=Homo sapiens GN=FBL PE=1 SV=2 | FBRL | 34 | 0.0046 | 95% | 100% | 100% | 100% | 100% | 100% | 100% | 100% | 100% | 100% | 100% | 100% |
| 1184 | 40S ribosomal protein S20 OS=Homo sapiens GN=RPS20 PE=1 SV=1 | RS20 | 13 | 0.59 | 100% | 100% | 100% | 100% | 100% | 100% | 100% | 100% | 100% | 100% | 100% | 100% |
| 1185 | All-trans-retinol 13,14-reductase OS=Homo sapiens GN=RETSAT PE=1 SV=2 | RETST | 67 | 0.016 | 100% | 100% | 100% | 100% | 100% | 100% | 95% | 95% | 100% | 100% | 100% | 100% |
| 1186 | 60S ribosomal protein L36 OS=Homo sapiens GN=RPL36 PE=1 SV=3 | RL36 | 12 | 0.99 | 100% | 100% | 100% | 100% | 100% | 95% | 100% | 100% | 0 | 100% | 100% | 100% |
| 1187 | Syntaxin-18 OS=Homo sapiens GN=STX18 PE=1 SV=1 | STX18 | 39 | 0.55 | 100% | 100% | 100% | 100% | 100% | 100% | 95% | 100% | 100% | 100% | 100% | 100% |
| 1188 | TAR DNA-binding protein 43 OS=Homo sapiens GN=TARDBP PE=1 SV=1 | TADBP | 45 | 0.5 | 100% | 100% | 100% | 100% | 100% | 100% | 100% | 100% | 100% | 100% | 100% | 100% |
| 1189 | ADP-ribosylation factor-like protein 6-interacting protein 1 OS=Homo sapiens GN=ARL6IP1 PE=1 SV=2 | AR6P1 | 23 | 0.12 | 100% | 100% | 100% | 100% | 100% | 100% | 100% | 100% | 100% | 100% | 100% | 100% |
| 1190 | Matrix-remodeling-associated protein 7 OS=Homo sapiens GN=MXRA7 PE=1 SV=1 | MXRA7 | 21 | 0.26 | 100% | 100% | 100% | 100% | 100% | 100% | 100% | 100% | 100% | 100% | 100% | 100% |
| 1191 | Oxysterol-binding protein-related protein 8 OS=Homo sapiens GN=OSBPL8 PE=1 SV=3 | OSBL8 | 101 | 0.35 | 100% | 100% | 100% | 100% | 100% | 100% | 0 | 100% | 100% | 100% | 100% | 100% |
| 1192 | Proline-, glutamic acid- and leucine-rich protein 1 OS=Homo sapiens GN=PELP1 PE=1 SV=2 | PELP1 | 120 | 0.013 | 95% | 100% | 100% | 100% | 100% | 100% | 100% | 100% | 100% | 100% | 100% | 100% |
| 1193 | Translocation protein SEC62 OS=Homo sapiens GN=SEC62 PE=1 SV=1 | SEC62 | 46 | 0.56 | 100% | 100% | 100% | 100% | 100% | 100% | 100% | 100% | 100% | 100% | 100% | 100% |
| 1194 | Transducin beta-like protein 2 OS=Homo sapiens GN=TBL2 PE=1 SV=1 | TBL2 | 50 | 0.25 | 100% | 100% | 100% | 100% | 100% | 100% | 100% | 100% | 100% | 100% | 100% | 100% |
| 1195 | Tricarboxylate transport protein, mitochondrial OS=Homo sapiens GN=SLC25A1 PE=1 SV=2 | TXTP | 34 | 0.22 | 100% | 100% | 100% | 100% | 100% | 100% | 100% | 100% | 100% | 100% | 100% | 100% |
| 1196 | Small nuclear ribonucleoprotein-associated proteins B and B' OS=Homo sapiens GN=SNRPB PE=1 SV=2 | RSMB (+1) | 25 | 0.71 | 100% | 100% | 100% | 100% | 100% | 100% | 100% | 100% | 100% | 100% | 100% | 100% |
| 1197 | Protein YIF1B OS=Homo sapiens GN=YIF1B PE=1 SV=1 | YIF1B | 34 | 0.29 | 100% | 100% | 100% | 100% | 100% | 100% | 100% | 99% | 100% | 100% | 95% | 100% |
| 1198 | Endoplasmic reticulum-Golgi intermediate compartment protein 3 OS=Homo sapiens GN=ERGIC3 PE=1 SV=1 | ERGI3 | 43 | 0.57 | 100% | 100% | 100% | 98% | 100% | 100% | 100% | 100% | 100% | 100% | 59% | 94% |
| 1199 | Aggrecan core protein OS=Homo sapiens GN=ACAN PE=1 SV=2 | PGCA | 250 | < 0.00010 | 0 | 0 | 0 | 0 | 0 | 0 | 100% | 100% | 100% | 0 | 0 | 0 |
| 1200 | Transformer-2 protein homolog alpha OS=Homo sapiens GN=TRA2A PE=1 SV=1 | TRA2A | 33 | 0.092 | 100% | 100% | 100% | 0 | 100% | 100% | 100% | 100% | 100% | 100% | 100% | 100% |
| 1201 | Twinkle protein, mitochondrial OS=Homo sapiens GN=PEO1 PE=1 SV=1 | PEO1 | 77 | 0.0096 | 91% | 51% | 0 | 95% | 19% | 95% | 100% | 95% | 100% | 0 | 0 | 0 |
| 1202 | Very long-chain specific acyl-CoA dehydrogenase, mitochondrial OS=Homo sapiens GN=ACADVL PE=1 SV=1 | ACADV | 70 | 0.19 | 95% | 100% | 100% | 100% | 100% | 100% | 100% | 100% | 100% | 100% | 100% | 100% |
| 1203 | ADP-dependent glucokinase OS=Homo sapiens GN=ADPGK PE=1 SV=1 | ADPGK | 54 | 0.0093 | 100% | 100% | 100% | 100% | 100% | 100% | 100% | 100% | 95% | 100% | 100% | 100% |
| 1204 | Peptidyl-prolyl cis-trans isomerase FKBP10 OS=Homo sapiens GN=FKBP10 PE=1 SV=1 | FKB10 | 64 | 0.55 | 100% | 57% | 100% | 95% | 95% | 95% | 100% | 100% | 100% | 100% | 100% | 100% |
| 1205 | PRA1 family protein 2 OS=Homo sapiens GN=PRAF2 PE=1 SV=1 | PRAF2 | 19 | 0.24 | 100% | 100% | 100% | 100% | 100% | 100% | 100% | 100% | 100% | 100% | 100% | 100% |
| 1206 | 40S ribosomal protein S25 OS=Homo sapiens GN=RPS25 PE=1 SV=1 | RS25 | 14 | 0.058 | 100% | 100% | 100% | 100% | 100% | 100% | 100% | 100% | 100% | 100% | 100% | 100% |
| 1207 | Small nuclear ribonucleoprotein Sm D3 OS=Homo sapiens GN=SNRPD3 PE=1 SV=1 | SMD3 | 14 | 0.00096 | 95% | 100% | 100% | 100% | 100% | 100% | 100% | 100% | 100% | 100% | 100% | 100% |
| 1208 | 60S ribosomal protein L36a-like OS=Homo sapiens GN=RPL36AL PE=1 SV=3 | RL36L | 12 | 0.6 | 100% | 100% | 100% | 100% | 100% | 100% | 100% | 100% | 100% | 86% | 100% | 100% |
| 1209 | Cytochrome b5 type B OS=Homo sapiens GN=CYB5B PE=1 SV=2 | CYB5B | 16 | 0.011 | 100% | 100% | 100% | 100% | 100% | 100% | 100% | 100% | 100% | 100% | 100% | 100% |
| 1210 | Mannosyl-oligosaccharide 1,2-alpha-mannosidase IA OS=Homo sapiens GN=MAN1A1 PE=1 SV=3 | MA1A1 | 73 | 0.021 | 100% | 100% | 100% | 95% | 100% | 100% | 100% | 100% | 100% | 100% | 100% | 100% |
| 1211 | Peptidyl-tRNA hydrolase 2, mitochondrial OS=Homo sapiens GN=PTRH2 PE=1 SV=1 | PTH2 | 19 | 0.0053 | 100% | 100% | 100% | 100% | 100% | 100% | 100% | 100% | 100% | 100% | 100% | 100% |
| 1212 | Putative sodium-coupled neutral amino acid transporter 10 OS=Homo sapiens GN=SLC38A10 PE=1 SV=2 | S38AA | 120 | 0.28 | 95% | 99% | 95% | 100% | 100% | 100% | 100% | 98% | 100% | 100% | 100% | 100% |
| 1213 | Transmembrane protein 33 OS=Homo sapiens GN=TMEM33 PE=1 SV=2 | TMM33 | 28 | 0.022 | 100% | 100% | 100% | 100% | 100% | 100% | 100% | 100% | 100% | 100% | 100% | 100% |
| 1214 | Centromere/kinetochore protein zw10 homolog OS=Homo sapiens GN=ZW10 PE=1 SV=3 | ZW10 | 89 | 0.032 | 0 | 100% | 100% | 100% | 100% | 100% | 100% | 100% | 100% | 100% | 100% | 100% |
| 1215 | Ribosome biogenesis protein BRX1 homolog OS=Homo sapiens GN=BRIX1 PE=1 SV=2 | BRX1 | 41 | 0.35 | 21% | 100% | 100% | 100% | 99% | 100% | 100% | 100% | 100% | 100% | 99% | 100% |
| 1216 | Endosialin OS=Homo sapiens GN=CD248 PE=1 SV=1 | CD248 | 81 | < 0.00010 | 100% | 100% | 100% | 0 | 0 | 0 | 100% | 100% | 100% | 0 | 0 | 0 |
| 1217 | Collagen alpha-2(V) chain OS=Homo sapiens GN=COL5A2 PE=1 SV=3 | CO5A2 | 145 | 0.54 | 100% | 100% | 100% | 72% | 99% | 100% | 100% | 100% | 100% | 100% | 100% | 100% |
| 1218 | 2,4-dienoyl-CoA reductase, mitochondrial OS=Homo sapiens GN=DECR1 PE=1 SV=1 | DECR | 36 | 0.88 | 100% | 100% | 100% | 100% | 100% | 100% | 100% | 100% | 100% | 100% | 100% | 100% |
| 1219 | EGF-like repeat and discoidin I-like domain-containing protein 3 OS=Homo sapiens GN=EDIL3 PE=1 SV=1 | EDIL3 | 54 | 0.2 | 100% | 100% | 100% | 100% | 100% | 100% | 100% | 100% | 100% | 100% | 100% | 100% |
| 1220 | Protein-lysine 6-oxidase OS=Homo sapiens GN=LOX PE=1 SV=2 | LYOX | 47 | 0.1 | 100% | 100% | 100% | 100% | 100% | 100% | 100% | 100% | 100% | 100% | 100% | 100% |
| 1221 | Nucleolar protein 58 OS=Homo sapiens GN=NOP58 PE=1 SV=1 | NOP58 | 60 | 0.019 | 100% | 100% | 100% | 95% | 100% | 100% | 100% | 100% | 100% | 100% | 100% | 100% |
| 1222 | RNA-binding protein 14 OS=Homo sapiens GN=RBM14 PE=1 SV=2 | RBM14 | 69 | 0.0081 | 91% | 100% | 100% | 100% | 100% | 100% | 100% | 99% | 100% | 100% | 100% | 100% |
| 1223 | Sideroflexin-3 OS=Homo sapiens GN=SFXN3 PE=1 SV=2 | SFXN3 | 36 | < 0.00010 | 6% | 100% | 91% | 100% | 100% | 100% | 6% | 0 | 6% | 100% | 100% | 100% |
| 1224 | Sterol O-acyltransferase 1 OS=Homo sapiens GN=SOAT1 PE=1 SV=3 | SOAT1 | 65 | 0.12 | 100% | 100% | 100% | 100% | 100% | 100% | 100% | 95% | 53% | 100% | 100% | 100% |
| 1225 | DNA topoisomerase 1 OS=Homo sapiens GN=TOP1 PE=1 SV=2 | TOP1 | 91 | 0.0018 | 0 | 100% | 98% | 100% | 100% | 100% | 100% | 100% | 100% | 100% | 100% | 100% |
| 1226 | Chitobiosyldiphosphodolichol beta-mannosyltransferase OS=Homo sapiens GN=ALG1 PE=1 SV=2 | ALG1 | 53 | 0.93 | 100% | 100% | 100% | 100% | 100% | 100% | 100% | 100% | 100% | 100% | 100% | 100% |
| 1227 | DnaJ homolog subfamily B member 11 OS=Homo sapiens GN=DNAJB11 PE=1 SV=1 | DJB11 | 41 | 0.0026 | 100% | 100% | 100% | 100% | 100% | 100% | 100% | 0 | 100% | 100% | 100% | 100% |
| 1228 | Eukaryotic translation initiation factor 3 subunit B OS=Homo sapiens GN=EIF3B PE=1 SV=3 | EIF3B | 92 | 0.0048 | 95% | 100% | 95% | 100% | 100% | 100% | 100% | 100% | 100% | 100% | 100% | 100% |
| 1229 | Polypeptide N-acetylgalactosaminyltransferase 5 OS=Homo sapiens GN=GALNT5 PE=1 SV=1 | GALT5 | 106 | 0.035 | 100% | 100% | 100% | 100% | 100% | 100% | 100% | 100% | 100% | 100% | 100% | 100% |
| 1230 | Glycosyltransferase 8 domain-containing protein 1 OS=Homo sapiens GN=GLT8D1 PE=1 SV=2 | GL8D1 | 42 | 0.14 | 100% | 100% | 100% | 100% | 100% | 100% | 100% | 100% | 100% | 100% | 100% | 100% |
| 1231 | Bifunctional 3'-phosphoadenosine 5'-phosphosulfate synthase 2 OS=Homo sapiens GN=PAPSS2 PE=1 SV=2 | PAPS2 | 70 | 0.095 | 97% | 100% | 95% | 100% | 100% | 100% | 100% | 100% | 100% | 100% | 100% | 100% |
| 1232 | pre-rRNA processing protein FTSJ3 OS=Homo sapiens GN=FTSJ3 PE=1 SV=2 | SPB1 | 97 | 0.0087 | 0 | 0 | 100% | 100% | 100% | 100% | 100% | 100% | 100% | 100% | 100% | 100% |
| 1233 | Cell cycle and apoptosis regulator protein 2 OS=Homo sapiens GN=CCAR2 PE=1 SV=2 | CCAR2 | 103 | 0.0025 | 0 | 0 | 100% | 95% | 0 | 64% | 100% | 100% | 100% | 100% | 100% | 100% |
| 1234 | Dolichyl-diphosphooligosaccharide--protein glycosyltransferase subunit DAD1 OS=Homo sapiens GN=DAD1 PE=1 SV=3 | DAD1 | 12 | 0.36 | 100% | 100% | 100% | 100% | 100% | 100% | 100% | 100% | 100% | 100% | 100% | 100% |
| 1235 | Heterogeneous nuclear ribonucleoprotein U-like protein 1 OS=Homo sapiens GN=HNRNPUL1 PE=1 SV=2 | HNRL1 | 96 | 0.033 | 99% | 100% | 100% | 95% | 100% | 95% | 100% | 100% | 100% | 100% | 100% | 100% |
| 1236 | Insulin-like growth factor-binding protein 7 OS=Homo sapiens GN=IGFBP7 PE=1 SV=1 | IBP7 | 29 | 0.00026 | 95% | 100% | 100% | 95% | 100% | 100% | 100% | 100% | 100% | 100% | 100% | 100% |
| 1237 | Insulin-like growth factor 2 mRNA-binding protein 2 OS=Homo sapiens GN=IGF2BP2 PE=1 SV=2 | IF2B2 | 66 | 0.16 | 87% | 100% | 100% | 100% | 100% | 100% | 100% | 100% | 100% | 100% | 100% | 100% |
| 1238 | Lamina-associated polypeptide 2, isoforms beta/gamma OS=Homo sapiens GN=TMPO PE=1 SV=2 | LAP2B | 51 | 0.0018 | 95% | 100% | 100% | 100% | 100% | 100% | 100% | 95% | 95% | 100% | 100% | 100% |
| 1239 | Alpha-1,6-mannosyl-glycoprotein 2-beta-N-acetylglucosaminyltransferase OS=Homo sapiens GN=MGAT2 PE=1 SV=1 | MGAT2 | 52 | 0.0019 | 100% | 100% | 100% | 100% | 100% | 100% | 100% | 98% | 95% | 100% | 100% | 100% |
| 1240 | Nuclear mitotic apparatus protein 1 OS=Homo sapiens GN=NUMA1 PE=1 SV=2 | NUMA1 | 238 | 0.58 | 100% | 100% | 100% | 92% | 100% | 100% | 100% | 100% | 100% | 100% | 100% | 100% |
| 1241 | Polyadenylate-binding protein 2 OS=Homo sapiens GN=PABPN1 PE=1 SV=3 | PABP2 | 33 | 0.0049 | 100% | 100% | 100% | 100% | 95% | 0 | 100% | 100% | 100% | 100% | 100% | 100% |
| 1242 | Signal peptidase complex catalytic subunit SEC11A OS=Homo sapiens GN=SEC11A PE=1 SV=1 | SC11A | 21 | 0.34 | 100% | 100% | 100% | 95% | 100% | 100% | 100% | 100% | 100% | 100% | 100% | 8% |
| 1243 | Sphingosine-1-phosphate lyase 1 OS=Homo sapiens GN=SGPL1 PE=1 SV=3 | SGPL1 | 64 | 0.0041 | 0 | 100% | 95% | 95% | 100% | 100% | 100% | 100% | 100% | 100% | 100% | 100% |
| 1244 | SNW domain-containing protein 1 OS=Homo sapiens GN=SNW1 PE=1 SV=1 | SNW1 | 61 | 0.0038 | 100% | 100% | 100% | 0 | 0 | 100% | 100% | 100% | 100% | 100% | 100% | 100% |
| 1245 | Transmembrane emp24 domain-containing protein 2 OS=Homo sapiens GN=TMED2 PE=1 SV=1 | TMED2 | 23 | 0.015 | 100% | 100% | 100% | 100% | 100% | 100% | 100% | 100% | 100% | 0 | 100% | 100% |
| 1246 | Voltage-dependent anion-selective channel protein 3 OS=Homo sapiens GN=VDAC3 PE=1 SV=1 | VDAC3 | 31 | 0.17 | 100% | 100% | 100% | 100% | 100% | 100% | 100% | 100% | 100% | 100% | 100% | 100% |
| 1247 | Alkyldihydroxyacetonephosphate synthase, peroxisomal OS=Homo sapiens GN=AGPS PE=1 SV=1 | ADAS | 73 | 0.066 | 43% | 100% | 100% | 99% | 100% | 100% | 0 | 0 | 100% | 100% | 100% | 100% |
| 1248 | Eukaryotic translation initiation factor 3 subunit H OS=Homo sapiens GN=EIF3H PE=1 SV=1 | EIF3H | 40 | 0.0078 | 95% | 100% | 100% | 95% | 100% | 95% | 100% | 100% | 100% | 100% | 100% | 100% |
| 1249 | U4/U6.U5 tri-snRNP-associated protein 2 OS=Homo sapiens GN=USP39 PE=1 SV=2 | SNUT2 | 65 | 0.24 | 100% | 100% | 100% | 95% | 100% | 100% | 100% | 100% | 100% | 100% | 100% | 100% |
| 1250 | Solute carrier family 35 member F6 OS=Homo sapiens GN=SLC35F6 PE=1 SV=1 | S35F6 | 40 | 0.47 | 100% | 100% | 100% | 95% | 95% | 100% | 100% | 95% | 100% | 100% | 100% | 99% |
| 1251 | Cluster of 40S ribosomal protein S27 OS=Homo sapiens GN=RPS27 PE=1 SV=3 (RS27_HUMAN) | RS27 [2] | 9 | 0.51 | 100% | 100% | 100% | 100% | 100% | 100% | 100% | 100% | 100% | 100% | 100% | 100% |
| 1252 | Cluster of Eukaryotic initiation factor 4A-I OS=Homo sapiens GN=EIF4A1 PE=1 SV=1 (IF4A1_HUMAN) | IF4A1 [2] | 46 | 0.024 | 100% | 100% | 100% | 100% | 100% | 100% | 100% | 100% | 100% | 100% | 100% | 100% |
| 1253 | Cluster of Protein CASP OS=Homo sapiens GN=CUX1 PE=1 SV=2 (CASP_HUMAN) | CASP [2] | 77 | 0.017 | 99% | 100% | 95% | 95% | 100% | 100% | 100% | 100% | 100% | 100% | 100% | 99% |
| 1254 | Cluster of Chromodomain-helicase-DNA-binding protein 4 OS=Homo sapiens GN=CHD4 PE=1 SV=2 (CHD4_HUMAN) | CHD4 [3] | 218 | 0.062 | 0 | 86% | 100% | 100% | 100% | 59% | 100% | 100% | 100% | 100% | 100% | 100% |
| 1255 | Cytochrome c oxidase subunit 2 OS=Homo sapiens GN=MT-CO2 PE=1 SV=1 | COX2 | 26 | 0.00021 | 100% | 100% | 100% | 100% | 100% | 100% | 95% | 100% | 95% | 100% | 100% | 100% |
| 1256 | Protein transport protein Sec24B OS=Homo sapiens GN=SEC24B PE=1 SV=2 | SC24B | 137 | 0.49 | 98% | 95% | 100% | 88% | 100% | 100% | 100% | 100% | 100% | 57% | 100% | 100% |
| 1257 | Mitochondrial import receptor subunit TOM70 OS=Homo sapiens GN=TOMM70A PE=1 SV=1 | TOM70 | 67 | 0.17 | 95% | 100% | 100% | 100% | 100% | 100% | 65% | 95% | 100% | 100% | 100% | 100% |
| 1258 | Beta-galactosidase OS=Homo sapiens GN=GLB1 PE=1 SV=2 | BGAL | 76 | 0.026 | 100% | 100% | 100% | 0 | 100% | 92% | 100% | 100% | 100% | 100% | 100% | 100% |
| 1259 | ER membrane protein complex subunit 7 OS=Homo sapiens GN=EMC7 PE=1 SV=1 | EMC7 | 26 | 0.0033 | 100% | 100% | 100% | 100% | 100% | 100% | 100% | 95% | 95% | 100% | 100% | 100% |
| 1260 | Importin-5 OS=Homo sapiens GN=IPO5 PE=1 SV=4 | IPO5 | 124 | 0.074 | 0 | 100% | 99% | 100% | 100% | 100% | 100% | 100% | 100% | 100% | 100% | 100% |
| 1261 | Microtubule-associated protein 1A OS=Homo sapiens GN=MAP1A PE=1 SV=6 | MAP1A | 305 | 0.15 | 0 | 100% | 100% | 100% | 100% | 0 | 100% | 100% | 100% | 100% | 100% | 100% |
| 1262 | U4/U6.U5 tri-snRNP-associated protein 1 OS=Homo sapiens GN=SART1 PE=1 SV=1 | SNUT1 | 90 | 0.44 | 95% | 100% | 100% | 99% | 100% | 100% | 100% | 100% | 100% | 100% | 100% | 100% |
| 1263 | Mannosyl-oligosaccharide 1,2-alpha-mannosidase IB OS=Homo sapiens GN=MAN1A2 PE=1 SV=1 | MA1A2 | 73 | 0.001 | 100% | 100% | 95% | 100% | 100% | 100% | 100% | 95% | 100% | 100% | 100% | 100% |
| 1264 | Secretory carrier-associated membrane protein 2 OS=Homo sapiens GN=SCAMP2 PE=1 SV=2 | SCAM2 | 37 | 0.0067 | 0 | 100% | 100% | 100% | 100% | 100% | 100% | 100% | 100% | 100% | 100% | 100% |
| 1265 | Vesicle transport through interaction with t-SNAREs homolog 1A OS=Homo sapiens GN=VTI1A PE=1 SV=2 | VTI1A | 25 | 0.046 | 100% | 100% | 100% | 95% | 100% | 95% | 100% | 99% | 100% | 100% | 100% | 100% |
| 1266 | Eukaryotic translation initiation factor 3 subunit D OS=Homo sapiens GN=EIF3D PE=1 SV=1 | EIF3D | 64 | 0.29 | 100% | 100% | 88% | 95% | 100% | 100% | 100% | 100% | 100% | 100% | 100% | 100% |
| 1267 | N-acetylglucosamine-6-sulfatase OS=Homo sapiens GN=GNS PE=1 SV=3 | GNS | 62 | 0.11 | 100% | 100% | 100% | 100% | 100% | 100% | 96% | 0 | 100% | 100% | 100% | 100% |
| 1268 | Lipid phosphate phosphohydrolase 3 OS=Homo sapiens GN=PPAP2B PE=1 SV=1 | LPP3 | 35 | < 0.00010 | 100% | 100% | 100% | 0 | 0 | 0 | 95% | 0 | 0 | 93% | 100% | 100% |
| 1269 | Peroxidasin homolog OS=Homo sapiens GN=PXDN PE=1 SV=2 | PXDN | 165 | 0.014 | 99% | 95% | 100% | 100% | 100% | 100% | 100% | 100% | 100% | 100% | 100% | 100% |
| 1270 | Tripeptidyl-peptidase 2 OS=Homo sapiens GN=TPP2 PE=1 SV=4 | TPP2 | 138 | 0.31 | 100% | 100% | 66% | 92% | 100% | 100% | 100% | 53% | 95% | 100% | 100% | 100% |
| 1271 | Glycosaminoglycan xylosylkinase OS=Homo sapiens GN=FAM20B PE=1 SV=1 | XYLK | 46 | 0.35 | 100% | 100% | 100% | 100% | 100% | 100% | 100% | 100% | 100% | 100% | 100% | 100% |
| 1272 | Zinc transporter 7 OS=Homo sapiens GN=SLC30A7 PE=2 SV=1 | ZNT7 | 42 | 0.15 | 100% | 99% | 100% | 95% | 100% | 100% | 100% | 100% | 100% | 86% | 99% | 100% |
| 1273 | U1 small nuclear ribonucleoprotein 70 kDa OS=Homo sapiens GN=SNRNP70 PE=1 SV=2 | RU17 | 52 | 1 | 100% | 98% | 100% | 29% | 99% | 100% | 0 | 99% | 100% | 98% | 100% | 100% |
| 1274 | Citrate synthase, mitochondrial OS=Homo sapiens GN=CS PE=1 SV=2 | CISY | 52 | 0.33 | 100% | 100% | 100% | 100% | 95% | 100% | 100% | 100% | 100% | 100% | 100% | 100% |
| 1275 | Cartilage-associated protein OS=Homo sapiens GN=CRTAP PE=1 SV=1 | CRTAP | 47 | 0.13 | 63% | 99% | 66% | 100% | 100% | 100% | 100% | 99% | 100% | 100% | 100% | 100% |
| 1276 | Cytochrome b5 OS=Homo sapiens GN=CYB5A PE=1 SV=2 | CYB5 | 15 | 0.02 | 100% | 100% | 100% | 100% | 100% | 100% | 95% | 0 | 95% | 100% | 100% | 100% |
| 1277 | Cytoplasmic dynein 1 intermediate chain 2 OS=Homo sapiens GN=DYNC1I2 PE=1 SV=3 | DC1I2 | 71 | 0.024 | 98% | 100% | 95% | 100% | 100% | 100% | 100% | 100% | 100% | 95% | 100% | 95% |
| 1278 | 7-dehydrocholesterol reductase OS=Homo sapiens GN=DHCR7 PE=1 SV=1 | DHCR7 | 54 | 0.08 | 93% | 100% | 95% | 100% | 97% | 100% | 100% | 100% | 100% | 100% | 100% | 100% |
| 1279 | H/ACA ribonucleoprotein complex subunit 4 OS=Homo sapiens GN=DKC1 PE=1 SV=3 | DKC1 | 58 | 0.00016 | 0 | 97% | 99% | 95% | 100% | 100% | 100% | 100% | 100% | 100% | 100% | 100% |
| 1280 | Eukaryotic translation initiation factor 3 subunit L OS=Homo sapiens GN=EIF3L PE=1 SV=1 | EIF3L | 67 | 0.27 | 100% | 99% | 100% | 0 | 100% | 95% | 100% | 100% | 100% | 100% | 100% | 100% |
| 1281 | Lamin-B1 OS=Homo sapiens GN=LMNB1 PE=1 SV=2 | LMNB1 | 66 | 0.00031 | 0 | 100% | 100% | 100% | 100% | 100% | 100% | 100% | 77% | 100% | 100% | 100% |
| 1282 | Protein transport protein Sec61 subunit beta OS=Homo sapiens GN=SEC61B PE=1 SV=2 | SC61B | 10 | 0.77 | 100% | 100% | 100% | 100% | 100% | 100% | 100% | 100% | 100% | 100% | 100% | 100% |
| 1283 | Protein sel-1 homolog 1 OS=Homo sapiens GN=SEL1L PE=1 SV=3 | SE1L1 | 89 | 0.13 | 100% | 100% | 100% | 100% | 100% | 100% | 95% | 100% | 100% | 100% | 100% | 0 |
| 1284 | Acid ceramidase OS=Homo sapiens GN=ASAH1 PE=1 SV=5 | ASAH1 | 45 | 0.72 | 0 | 0 | 100% | 95% | 100% | 100% | 100% | 100% | 100% | 100% | 100% | 100% |
| 1285 | ER membrane protein complex subunit 2 OS=Homo sapiens GN=EMC2 PE=1 SV=1 | EMC2 | 35 | 0.81 | 100% | 100% | 100% | 95% | 95% | 100% | 100% | 100% | 100% | 100% | 100% | 100% |
| 1286 | GPI transamidase component PIG-T OS=Homo sapiens GN=PIGT PE=1 SV=1 | PIGT | 66 | 0.02 | 100% | 100% | 100% | 100% | 100% | 100% | 0 | 95% | 0 | 100% | 100% | 100% |
| 1287 | Sideroflexin-1 OS=Homo sapiens GN=SFXN1 PE=1 SV=4 | SFXN1 | 36 | 0.021 | 0 | 100% | 100% | 100% | 100% | 100% | 100% | 95% | 100% | 100% | 100% | 100% |
| 1288 | WD40 repeat-containing protein SMU1 OS=Homo sapiens GN=SMU1 PE=1 SV=2 | SMU1 | 58 | 0.0028 | 100% | 100% | 100% | 15% | 100% | 82% | 100% | 100% | 100% | 100% | 100% | 100% |
| 1289 | Signal recognition particle 14 kDa protein OS=Homo sapiens GN=SRP14 PE=1 SV=2 | SRP14 | 15 | 0.45 | 95% | 100% | 100% | 0 | 100% | 100% | 100% | 98% | 100% | 100% | 100% | 100% |
| 1290 | 40S ribosomal protein S30 OS=Homo sapiens GN=FAU PE=1 SV=1 | RS30 | 7 | 0.16 | 95% | 100% | 100% | 100% | 100% | 100% | 100% | 100% | 100% | 100% | 100% | 100% |
| 1291 | Pituitary tumor-transforming gene 1 protein-interacting protein OS=Homo sapiens GN=PTTG1IP PE=1 SV=1 | PTTG | 20 | 0.0079 | 100% | 95% | 100% | 100% | 100% | 95% | 100% | 100% | 100% | 95% | 95% | 95% |
| 1292 | Protein CASC4 OS=Homo sapiens GN=CASC4 PE=1 SV=1 | CASC4 | 49 | 0.83 | 100% | 100% | 100% | 100% | 100% | 100% | 100% | 95% | 100% | 100% | 0 | 100% |
| 1293 | ATP-dependent RNA helicase DDX18 OS=Homo sapiens GN=DDX18 PE=1 SV=2 | DDX18 | 75 | 0.32 | 95% | 100% | 100% | 100% | 100% | 100% | 100% | 100% | 100% | 100% | 100% | 100% |
| 1294 | Importin subunit beta-1 OS=Homo sapiens GN=KPNB1 PE=1 SV=2 | IMB1 | 97 | 0.0071 | 100% | 100% | 100% | 95% | 100% | 100% | 0 | 100% | 25% | 100% | 100% | 100% |
| 1295 | Matrix-remodeling-associated protein 8 OS=Homo sapiens GN=MXRA8 PE=2 SV=1 | MXRA8 | 49 | 0.0064 | 100% | 100% | 100% | 100% | 100% | 100% | 100% | 100% | 100% | 100% | 0 | 95% |
| 1296 | Ribosome biogenesis regulatory protein homolog OS=Homo sapiens GN=RRS1 PE=1 SV=2 | RRS1 | 41 | 0.031 | 95% | 0 | 100% | 95% | 100% | 100% | 100% | 100% | 100% | 100% | 100% | 100% |
| 1297 | DBIRD complex subunit ZNF326 OS=Homo sapiens GN=ZNF326 PE=1 SV=2 | ZN326 | 66 | 0.0068 | 100% | 100% | 100% | 95% | 0 | 95% | 100% | 95% | 95% | 100% | 100% | 100% |
| 1298 | Translocon-associated protein subunit alpha OS=Homo sapiens GN=SSR1 PE=1 SV=3 | SSRA | 32 | 0.44 | 100% | 63% | 100% | 100% | 95% | 100% | 100% | 100% | 100% | 100% | 100% | 100% |
| 1299 | Ancient ubiquitous protein 1 OS=Homo sapiens GN=AUP1 PE=1 SV=1 | AUP1 | 53 | 0.26 | 100% | 15% | 100% | 95% | 100% | 100% | 100% | 100% | 95% | 100% | 100% | 100% |
| 1300 | Insulin-like growth factor 2 mRNA-binding protein 3 OS=Homo sapiens GN=IGF2BP3 PE=1 SV=2 | IF2B3 | 64 | 0.00013 | 74% | 100% | 100% | 100% | 100% | 100% | 55% | 0 | 0 | 100% | 100% | 100% |
| 1301 | Protein-methionine sulfoxide oxidase MICAL2 OS=Homo sapiens GN=MICAL2 PE=1 SV=1 | MICA2 | 127 | 0.0025 | 100% | 100% | 0 | 84% | 100% | 100% | 100% | 100% | 100% | 16% | 95% | 9% |
| 1302 | Prolactin regulatory element-binding protein OS=Homo sapiens GN=PREB PE=1 SV=2 | PREB | 45 | 0.93 | 100% | 100% | 100% | 100% | 95% | 100% | 100% | 100% | 100% | 100% | 100% | 100% |
| 1303 | Type 2 phosphatidylinositol 4,5-bisphosphate 4-phosphatase OS=Homo sapiens GN=TMEM55A PE=1 SV=1 | TM55A | 28 | 0.15 | 0 | 100% | 100% | 95% | 95% | 95% | 100% | 100% | 100% | 95% | 95% | 95% |
| 1304 | Peroxiredoxin-4 OS=Homo sapiens GN=PRDX4 PE=1 SV=1 | PRDX4 | 31 | 0.15 | 100% | 100% | 100% | 100% | 100% | 100% | 100% | 100% | 100% | 100% | 100% | 100% |
| 1305 | Putative RNA-binding protein 3 OS=Homo sapiens GN=RBM3 PE=1 SV=1 | RBM3 | 17 | 0.089 | 95% | 100% | 100% | 95% | 95% | 100% | 100% | 95% | 95% | 100% | 100% | 100% |
| 1306 | Serine palmitoyltransferase 2 OS=Homo sapiens GN=SPTLC2 PE=1 SV=1 | SPTC2 | 63 | 0.064 | 54% | 100% | 95% | 100% | 100% | 100% | 100% | 100% | 100% | 100% | 13% | 100% |
| 1307 | Atlastin-1 OS=Homo sapiens GN=ATL1 PE=1 SV=1 | ATLA1 | 64 | 0.43 | 95% | 100% | 100% | 100% | 100% | 100% | 100% | 100% | 100% | 100% | 100% | 100% |
| 1308 | Cleavage and polyadenylation specificity factor subunit 6 OS=Homo sapiens GN=CPSF6 PE=1 SV=2 | CPSF6 | 59 | 0.53 | 95% | 100% | 100% | 100% | 100% | 100% | 100% | 100% | 100% | 100% | 0 | 100% |
| 1309 | Ras GTPase-activating protein-binding protein 2 OS=Homo sapiens GN=G3BP2 PE=1 SV=2 | G3BP2 | 54 | 0.68 | 100% | 100% | 100% | 99% | 100% | 100% | 100% | 100% | 100% | 100% | 99% | 100% |
| 1310 | Growth arrest-specific protein 6 OS=Homo sapiens GN=GAS6 PE=1 SV=2 | GAS6 | 80 | 0.39 | 99% | 95% | 100% | 100% | 100% | 95% | 100% | 95% | 100% | 100% | 100% | 95% |
| 1311 | N-acetyltransferase 10 OS=Homo sapiens GN=NAT10 PE=1 SV=2 | NAT10 | 116 | 0.057 | 100% | 100% | 100% | 97% | 100% | 100% | 100% | 100% | 100% | 100% | 11% | 100% |
| 1312 | Phenylalanine--tRNA ligase beta subunit OS=Homo sapiens GN=FARSB PE=1 SV=3 | SYFB | 66 | 0.2 | 0 | 100% | 83% | 100% | 100% | 100% | 100% | 100% | 22% | 100% | 100% | 100% |
| 1313 | Protein SON OS=Homo sapiens GN=SON PE=1 SV=4 | SON | 264 | 0.28 | 0 | 100% | 100% | 100% | 100% | 100% | 98% | 99% | 100% | 100% | 100% | 100% |
| 1314 | Actin-related protein 2/3 complex subunit 4 OS=Homo sapiens GN=ARPC4 PE=1 SV=3 | ARPC4 | 20 | 0.63 | 95% | 100% | 100% | 40% | 65% | 100% | 100% | 100% | 100% | 100% | 95% | 100% |
| 1315 | Cleft lip and palate transmembrane protein 1-like protein OS=Homo sapiens GN=CLPTM1L PE=1 SV=1 | CLP1L | 62 | 0.075 | 0 | 100% | 95% | 98% | 100% | 100% | 98% | 100% | 24% | 100% | 100% | 100% |
| 1316 | Enhancer of mRNA-decapping protein 4 OS=Homo sapiens GN=EDC4 PE=1 SV=1 | EDC4 | 152 | 0.069 | 100% | 100% | 100% | 95% | 100% | 100% | 100% | 100% | 100% | 100% | 100% | 0 |
| 1317 | UPF0577 protein KIAA1324-like OS=Homo sapiens GN=KIAA1324L PE=2 SV=2 | K132L | 114 | 0.0079 | 95% | 100% | 100% | 100% | 100% | 100% | 0 | 0 | 0 | 100% | 100% | 100% |
| 1318 | Protein LAP2 OS=Homo sapiens GN=ERBB2IP PE=1 SV=2 | LAP2 | 158 | 0.55 | 43% | 100% | 100% | 5% | 100% | 100% | 95% | 100% | 95% | 100% | 100% | 100% |
| 1319 | Lamin-B receptor OS=Homo sapiens GN=LBR PE=1 SV=2 | LBR | 71 | 0.093 | 0 | 100% | 100% | 100% | 100% | 100% | 0 | 7% | 79% | 78% | 100% | 100% |
| 1320 | Pescadillo homolog OS=Homo sapiens GN=PES1 PE=1 SV=1 | PESC | 68 | 0.071 | 0 | 100% | 53% | 76% | 100% | 95% | 100% | 95% | 100% | 100% | 98% | 100% |
| 1321 | Calcium-binding mitochondrial carrier protein SCaMC-1 OS=Homo sapiens GN=SLC25A24 PE=1 SV=2 | SCMC1 | 53 | 0.0013 | 0 | 0 | 100% | 100% | 100% | 100% | 0 | 0 | 0 | 100% | 100% | 100% |
| 1322 | Polypeptide N-acetylgalactosaminyltransferase 1 OS=Homo sapiens GN=GALNT1 PE=1 SV=1 | GALT1 | 64 | 0.16 | 0 | 13% | 100% | 100% | 100% | 100% | 100% | 0 | 100% | 100% | 100% | 100% |
| 1323 | U4/U6 small nuclear ribonucleoprotein Prp4 OS=Homo sapiens GN=PRPF4 PE=1 SV=2 | PRP4 | 58 | 0.0047 | 0 | 100% | 100% | 0 | 100% | 0 | 100% | 100% | 100% | 100% | 100% | 100% |
| 1324 | Rab11 family-interacting protein 5 OS=Homo sapiens GN=RAB11FIP5 PE=1 SV=1 | RFIP5 | 70 | 0.0036 | 100% | 100% | 100% | 100% | 100% | 100% | 100% | 95% | 95% | 100% | 100% | 100% |
| 1325 | Ribosome production factor 2 homolog OS=Homo sapiens GN=RPF2 PE=1 SV=2 | RPF2 | 36 | 0.0066 | 0 | 0 | 100% | 0 | 0 | 0 | 100% | 100% | 100% | 100% | 100% | 100% |
| 1326 | 40S ribosomal protein S12 OS=Homo sapiens GN=RPS12 PE=1 SV=3 | RS12 | 15 | 0.038 | 95% | 95% | 95% | 100% | 100% | 100% | 92% | 95% | 95% | 100% | 100% | 100% |
| 1327 | 40S ribosomal protein S23 OS=Homo sapiens GN=RPS23 PE=1 SV=3 | RS23 | 16 | 0.11 | 100% | 100% | 100% | 95% | 100% | 0 | 100% | 95% | 100% | 100% | 95% | 100% |
| 1328 | U5 small nuclear ribonucleoprotein 40 kDa protein OS=Homo sapiens GN=SNRNP40 PE=1 SV=1 | SNR40 | 39 | 0.022 | 0 | 95% | 100% | 100% | 95% | 99% | 95% | 100% | 95% | 100% | 100% | 100% |
| 1329 | N-sulphoglucosamine sulphohydrolase OS=Homo sapiens GN=SGSH PE=1 SV=1 | SPHM | 57 | 0.014 | 95% | 100% | 95% | 95% | 100% | 100% | 100% | 100% | 100% | 100% | 100% | 100% |
| 1330 | Protein-tyrosine sulfotransferase 2 OS=Homo sapiens GN=TPST2 PE=1 SV=1 | TPST2 | 42 | 0.0035 | 100% | 99% | 95% | 95% | 0 | 100% | 100% | 100% | 100% | 99% | 95% | 0 |
| 1331 | Transmembrane and coiled-coil domain-containing protein 1 OS=Homo sapiens GN=TMCO1 PE=1 SV=1 | TMCO1 | 21 | 0.99 | 95% | 100% | 100% | 95% | 100% | 100% | 100% | 94% | 100% | 100% | 100% | 100% |
| 1332 | Elongation of very long chain fatty acids protein 1 OS=Homo sapiens GN=ELOVL1 PE=1 SV=1 | ELOV1 | 33 | 0.58 | 95% | 95% | 95% | 95% | 95% | 95% | 95% | 100% | 100% | 95% | 95% | 95% |
| 1333 | Zinc transporter SLC39A7 OS=Homo sapiens GN=SLC39A7 PE=1 SV=2 | S39A7 | 50 | 0.12 | 98% | 100% | 100% | 100% | 100% | 100% | 100% | 95% | 0 | 100% | 100% | 100% |
| 1334 | Cluster of 6-phosphofructokinase type C OS=Homo sapiens GN=PFKP PE=1 SV=2 (K6PP_HUMAN) | K6PP [3] | 86 | 0.019 | 100% | 100% | 100% | 99% | 100% | 0 | 100% | 100% | 100% | 58% | 0 | 95% |
| 1335 | Endoplasmic reticulum lectin 1 OS=Homo sapiens GN=ERLEC1 PE=1 SV=1 | ERLEC | 55 | 0.016 | 99% | 100% | 100% | 95% | 100% | 100% | 100% | 63% | 100% | 100% | 100% | 100% |
| 1336 | Guanine nucleotide-binding protein subunit beta-2-like 1 OS=Homo sapiens GN=GNB2L1 PE=1 SV=3 | GBLP | 35 | 0.00024 | 95% | 0 | 95% | 100% | 95% | 68% | 100% | 100% | 100% | 100% | 100% | 100% |
| 1337 | Eukaryotic translation initiation factor 2 subunit 2 OS=Homo sapiens GN=EIF2S2 PE=1 SV=2 | IF2B | 38 | 0.011 | 0 | 99% | 0 | 0 | 100% | 97% | 100% | 100% | 100% | 100% | 100% | 100% |
| 1338 | Lysophospholipid acyltransferase LPCAT4 OS=Homo sapiens GN=LPCAT4 PE=1 SV=1 | LPCT4 | 57 | 0.92 | 100% | 100% | 100% | 95% | 100% | 100% | 100% | 100% | 100% | 100% | 100% | 95% |
| 1339 | Ragulator complex protein LAMTOR2 OS=Homo sapiens GN=LAMTOR2 PE=1 SV=1 | LTOR2 | 14 | 0.015 | 95% | 100% | 100% | 0 | 0 | 95% | 100% | 100% | 100% | 100% | 100% | 100% |
| 1340 | Melanoma inhibitory activity protein 3 OS=Homo sapiens GN=MIA3 PE=1 SV=1 | MIA3 | 214 | 0.017 | 6% | 100% | 100% | 93% | 100% | 100% | 100% | 0 | 0 | 100% | 100% | 100% |
| 1341 | Proliferation-associated protein 2G4 OS=Homo sapiens GN=PA2G4 PE=1 SV=3 | PA2G4 | 44 | 0.066 | 95% | 100% | 95% | 95% | 100% | 99% | 100% | 100% | 100% | 10% | 0 | 99% |
| 1342 | Poly [ADP-ribose] polymerase 1 OS=Homo sapiens GN=PARP1 PE=1 SV=4 | PARP1 | 113 | 0.053 | 100% | 100% | 0 | 100% | 100% | 100% | 100% | 100% | 95% | 100% | 100% | 100% |
| 1343 | Lysophosphatidylcholine acyltransferase 1 OS=Homo sapiens GN=LPCAT1 PE=1 SV=2 | PCAT1 | 59 | 0.47 | 100% | 100% | 100% | 95% | 100% | 95% | 100% | 100% | 95% | 100% | 100% | 100% |
| 1344 | Putative phospholipase B-like 2 OS=Homo sapiens GN=PLBD2 PE=1 SV=2 | PLBL2 | 65 | 0.055 | 100% | 100% | 100% | 0 | 0 | 95% | 100% | 100% | 14% | 100% | 100% | 100% |
| 1345 | Histone deacetylase complex subunit SAP18 OS=Homo sapiens GN=SAP18 PE=1 SV=1 | SAP18 | 18 | 0.14 | 100% | 100% | 100% | 0 | 100% | 100% | 0 | 100% | 100% | 100% | 100% | 100% |
| 1346 | Isoleucine--tRNA ligase, cytoplasmic OS=Homo sapiens GN=IARS PE=1 SV=2 | SYIC | 145 | 0.53 | 0 | 100% | 100% | 94% | 100% | 100% | 100% | 100% | 100% | 100% | 100% | 100% |
| 1347 | Zinc finger RNA-binding protein OS=Homo sapiens GN=ZFR PE=1 SV=2 | ZFR | 117 | 0.0031 | 95% | 100% | 100% | 0 | 0 | 95% | 100% | 0 | 100% | 100% | 100% | 100% |
| 1348 | Motile sperm domain-containing protein 2 OS=Homo sapiens GN=MOSPD2 PE=1 SV=1 | MSPD2 | 60 | 0.00012 | 100% | 100% | 100% | 100% | 100% | 100% | 100% | 100% | 100% | 98% | 99% | 99% |
| 1349 | ATP-dependent RNA helicase DDX24 OS=Homo sapiens GN=DDX24 PE=1 SV=1 | DDX24 | 96 | 0.0018 | 0 | 95% | 5% | 100% | 97% | 100% | 100% | 100% | 100% | 95% | 99% | 100% |
| 1350 | GPI-anchor transamidase OS=Homo sapiens GN=PIGK PE=1 SV=2 | GPI8 | 45 | 0.049 | 100% | 100% | 100% | 0 | 100% | 95% | 0 | 95% | 100% | 100% | 100% | 100% |
| 1351 | Tumor protein p53-inducible protein 11 OS=Homo sapiens GN=TP53I11 PE=1 SV=2 | P5I11 | 21 | 0.034 | 100% | 100% | 100% | 95% | 100% | 100% | 86% | 87% | 99% | 95% | 95% | 100% |
| 1352 | THO complex subunit 4 OS=Homo sapiens GN=ALYREF PE=1 SV=3 | THOC4 | 27 | 0.9 | 100% | 95% | 100% | 0 | 100% | 100% | 100% | 100% | 95% | 100% | 100% | 100% |
| 1353 | Torsin-1A-interacting protein 1 OS=Homo sapiens GN=TOR1AIP1 PE=1 SV=2 | TOIP1 | 66 | 0.0017 | 100% | 100% | 100% | 100% | 100% | 100% | 100% | 95% | 95% | 95% | 95% | 0 |
| 1354 | Splicing factor U2AF 35 kDa subunit OS=Homo sapiens GN=U2AF1 PE=1 SV=3 | U2AF1 | 28 | 0.03 | 100% | 100% | 95% | 100% | 100% | 100% | 100% | 100% | 100% | 100% | 100% | 100% |
| 1355 | Type 1 phosphatidylinositol 4,5-bisphosphate 4-phosphatase OS=Homo sapiens GN=TMEM55B PE=1 SV=1 | TM55B | 29 | 0.63 | 100% | 100% | 100% | 0 | 100% | 100% | 100% | 100% | 100% | 95% | 100% | 100% |
| 1356 | Hornerin OS=Homo sapiens GN=HRNR PE=1 SV=2 | HORN | 282 | 0.24 | 24% | 100% | 100% | 95% | 100% | 100% | 100% | 97% | 100% | 99% | 99% | 99% |
| 1357 | Translocon-associated protein subunit gamma OS=Homo sapiens GN=SSR3 PE=1 SV=1 | SSRG | 21 | 0.12 | 95% | 95% | 100% | 95% | 95% | 100% | 95% | 95% | 95% | 0 | 100% | 0 |
| 1358 | GA-binding protein subunit beta-2 OS=Homo sapiens GN=GABPB2 PE=2 SV=1 | GABP2 | 49 | 0.59 | 0 | 35% | 26% | 63% | 80% | 35% | 48% | 61% | 27% | 95% | 25% | 42% |
| 1359 | Dynactin subunit 2 OS=Homo sapiens GN=DCTN2 PE=1 SV=4 | DCTN2 | 44 | 0.012 | 0 | 75% | 100% | 100% | 100% | 100% | 100% | 100% | 100% | 0 | 28% | 0 |
| 1360 | Mesoderm-specific transcript homolog protein OS=Homo sapiens GN=MEST PE=2 SV=2 | MEST | 39 | 0.001 | 100% | 100% | 100% | 0 | 37% | 95% | 0 | 0 | 95% | 100% | 100% | 100% |
| 1361 | SEC23-interacting protein OS=Homo sapiens GN=SEC23IP PE=1 SV=1 | S23IP | 111 | 0.4 | 100% | 100% | 98% | 100% | 100% | 97% | 100% | 100% | 100% | 100% | 100% | 100% |
| 1362 | Protein spinster homolog 1 OS=Homo sapiens GN=SPNS1 PE=1 SV=1 | SPNS1 | 57 | 0.26 | 100% | 100% | 95% | 100% | 100% | 100% | 100% | 100% | 100% | 100% | 95% | 95% |
| 1363 | V-type proton ATPase subunit D OS=Homo sapiens GN=ATP6V1D PE=1 SV=1 | VATD | 28 | 0.12 | 100% | 100% | 95% | 19% | 100% | 100% | 100% | 100% | 100% | 100% | 100% | 95% |
| 1364 | Transcriptional activator protein Pur-alpha OS=Homo sapiens GN=PURA PE=1 SV=2 | PURA | 35 | 0.021 | 95% | 100% | 100% | 100% | 100% | 100% | 100% | 100% | 100% | 100% | 100% | 100% |
| 1365 | Exportin-2 OS=Homo sapiens GN=CSE1L PE=1 SV=3 | XPO2 | 110 | 0.34 | 95% | 65% | 0 | 95% | 87% | 0 | 0 | 0 | 0 | 36% | 0 | 100% |
| 1366 | ATP-binding cassette sub-family E member 1 OS=Homo sapiens GN=ABCE1 PE=1 SV=1 | ABCE1 | 67 | 0.26 | 95% | 95% | 100% | 7% | 95% | 95% | 95% | 95% | 95% | 100% | 100% | 100% |
| 1367 | Cytochrome c oxidase subunit 5B, mitochondrial OS=Homo sapiens GN=COX5B PE=1 SV=2 | COX5B | 14 | 0.028 | 0 | 100% | 100% | 100% | 100% | 100% | 0 | 0 | 0 | 100% | 100% | 100% |
| 1368 | Probable ATP-dependent RNA helicase DDX27 OS=Homo sapiens GN=DDX27 PE=1 SV=2 | DDX27 | 90 | 0.19 | 100% | 95% | 95% | 0 | 100% | 100% | 100% | 100% | 100% | 100% | 95% | 100% |
| 1369 | Eukaryotic translation initiation factor 3 subunit I OS=Homo sapiens GN=EIF3I PE=1 SV=1 | EIF3I | 37 | 0.69 | 100% | 100% | 100% | 0 | 100% | 78% | 100% | 100% | 100% | 100% | 100% | 100% |
| 1370 | Insulin-like growth factor 2 mRNA-binding protein 1 OS=Homo sapiens GN=IGF2BP1 PE=1 SV=2 | IF2B1 | 63 | 0.049 | 40% | 99% | 99% | 100% | 100% | 100% | 99% | 99% | 98% | 100% | 100% | 100% |
| 1371 | Procollagen C-endopeptidase enhancer 1 OS=Homo sapiens GN=PCOLCE PE=1 SV=2 | PCOC1 | 48 | 0.04 | 100% | 100% | 100% | 0 | 0 | 95% | 95% | 95% | 100% | 100% | 100% | 100% |
| 1372 | Lysosomal protective protein OS=Homo sapiens GN=CTSA PE=1 SV=2 | PPGB | 54 | 0.13 | 100% | 100% | 100% | 0 | 0 | 15% | 100% | 0 | 100% | 99% | 0 | 100% |
| 1373 | Protein Red OS=Homo sapiens GN=IK PE=1 SV=3 | RED | 66 | 0.0068 | 95% | 100% | 100% | 0 | 100% | 99% | 100% | 100% | 61% | 100% | 100% | 100% |
| 1374 | Kinase D-interacting substrate of 220 kDa OS=Homo sapiens GN=KIDINS220 PE=1 SV=3 | KDIS | 197 | 0.2 | 0 | 98% | 100% | 99% | 100% | 100% | 100% | 100% | 95% | 95% | 100% | 100% |
| 1375 | Prolyl 3-hydroxylase 1 OS=Homo sapiens GN=LEPRE1 PE=1 SV=2 | P3H1 | 83 | 0.68 | 100% | 99% | 100% | 95% | 100% | 100% | 100% | 100% | 100% | 95% | 95% | 86% |
| 1376 | Ceramide synthase 2 OS=Homo sapiens GN=CERS2 PE=1 SV=1 | CERS2 | 45 | 0.015 | 95% | 100% | 100% | 100% | 100% | 100% | 95% | 100% | 100% | 95% | 56% | 95% |
| 1377 | Eukaryotic translation initiation factor 6 OS=Homo sapiens GN=EIF6 PE=1 SV=1 | IF6 | 27 | 0.075 | 95% | 95% | 95% | 95% | 95% | 95% | 100% | 100% | 100% | 100% | 100% | 100% |
| 1378 | Vesicle transport protein USE1 OS=Homo sapiens GN=USE1 PE=1 SV=2 | USE1 | 29 | 0.37 | 99% | 100% | 100% | 100% | 100% | 100% | 100% | 90% | 100% | 100% | 100% | 100% |
| 1379 | Small nuclear ribonucleoprotein E OS=Homo sapiens GN=SNRPE PE=1 SV=1 | RUXE | 11 | 0.14 | 95% | 100% | 95% | 95% | 99% | 95% | 95% | 100% | 95% | 100% | 98% | 100% |
| 1380 | ATP-dependent RNA helicase DDX54 OS=Homo sapiens GN=DDX54 PE=1 SV=2 | DDX54 | 99 | 0.37 | 97% | 0 | 100% | 100% | 100% | 100% | 100% | 100% | 100% | 9% | 100% | 100% |
| 1381 | Testis-expressed sequence 10 protein OS=Homo sapiens GN=TEX10 PE=1 SV=2 | TEX10 | 106 | 0.0032 | 0 | 100% | 100% | 0 | 0 | 0 | 100% | 95% | 100% | 100% | 100% | 100% |
| 1382 | TLD domain-containing protein 1 OS=Homo sapiens GN=TLDC1 PE=1 SV=2 | TLDC1 | 51 | 0.6 | 58% | 100% | 100% | 100% | 100% | 100% | 100% | 100% | 100% | 100% | 100% | 100% |
| 1383 | Pre-mRNA-processing factor 6 OS=Homo sapiens GN=PRPF6 PE=1 SV=1 | PRP6 | 107 | 0.18 | 97% | 100% | 100% | 100% | 99% | 0 | 100% | 0 | 100% | 100% | 100% | 99% |
| 1384 | Chromatin target of PRMT1 protein OS=Homo sapiens GN=CHTOP PE=1 SV=2 | CHTOP | 26 | 0.055 | 100% | 95% | 100% | 99% | 99% | 100% | 98% | 97% | 95% | 100% | 100% | 100% |
| 1385 | Prosaposin OS=Homo sapiens GN=PSAP PE=1 SV=2 | SAP | 58 | 0.78 | 100% | 100% | 100% | 100% | 0 | 100% | 95% | 100% | 100% | 100% | 100% | 95% |
| 1386 | Endoplasmic reticulum-Golgi intermediate compartment protein 2 OS=Homo sapiens GN=ERGIC2 PE=1 SV=2 | ERGI2 | 43 | 0.69 | 97% | 100% | 100% | 100% | 100% | 100% | 95% | 100% | 100% | 95% | 100% | 95% |
| 1387 | Translocator protein OS=Homo sapiens GN=TSPO PE=1 SV=3 | TSPOA | 19 | 0.054 | 95% | 6% | 100% | 95% | 95% | 95% | 95% | 100% | 6% | 100% | 100% | 100% |
| 1388 | SPRY domain-containing protein 7 OS=Homo sapiens GN=SPRYD7 PE=1 SV=2 | SPRY7 | 22 | 0.71 | 99% | 86% | 100% | 18% | 100% | 100% | 100% | 0 | 100% | 60% | 80% | 100% |
| 1389 | Cluster of Spliceosome RNA helicase DDX39B OS=Homo sapiens GN=DDX39B PE=1 SV=1 (DX39B_HUMAN) | DX39B [2] | 49 | 0.35 | 100% | 100% | 100% | 0 | 19% | 100% | 95% | 100% | 78% | 100% | 100% | 100% |
| 1390 | Cluster of DNA topoisomerase 2-beta OS=Homo sapiens GN=TOP2B PE=1 SV=3 (TOP2B_HUMAN) | TOP2B [2] | 183 | 0.028 | 95% | 100% | 100% | 100% | 100% | 100% | 0 | 100% | 95% | 0 | 10% | 100% |
| 1391 | Apoptosis-inducing factor 1, mitochondrial OS=Homo sapiens GN=AIFM1 PE=1 SV=1 | AIFM1 | 67 | 0.0031 | 0 | 95% | 95% | 100% | 100% | 100% | 0 | 0 | 0 | 100% | 100% | 100% |
| 1392 | Cytochrome c oxidase subunit 4 isoform 1, mitochondrial OS=Homo sapiens GN=COX4I1 PE=1 SV=1 | COX41 | 20 | 0.0017 | 95% | 97% | 95% | 95% | 100% | 95% | 95% | 95% | 95% | 100% | 100% | 100% |
| 1393 | Glycosylphosphatidylinositol anchor attachment 1 protein OS=Homo sapiens GN=GPAA1 PE=1 SV=3 | GPAA1 | 68 | 0.059 | 95% | 100% | 100% | 99% | 100% | 100% | 100% | 100% | 100% | 29% | 0 | 35% |
| 1394 | Protein RRP5 homolog OS=Homo sapiens GN=PDCD11 PE=1 SV=3 | RRP5 | 209 | < 0.00010 | 0 | 13% | 94% | 12% | 100% | 0 | 14% | 0 | 91% | 100% | 100% | 100% |
| 1395 | Solute carrier family 35 member E1 OS=Homo sapiens GN=SLC35E1 PE=1 SV=2 | S35E1 | 45 | 0.077 | 95% | 100% | 100% | 100% | 100% | 100% | 100% | 11% | 99% | 95% | 95% | 95% |
| 1396 | Leucine-rich PPR motif-containing protein, mitochondrial OS=Homo sapiens GN=LRPPRC PE=1 SV=3 | LPPRC | 158 | 0.28 | 0 | 100% | 6% | 94% | 0 | 97% | 100% | 93% | 100% | 100% | 100% | 99% |
| 1397 | Mitotic checkpoint protein BUB3 OS=Homo sapiens GN=BUB3 PE=1 SV=1 | BUB3 | 37 | 0.82 | 95% | 95% | 100% | 100% | 100% | 99% | 100% | 100% | 100% | 100% | 100% | 100% |
| 1398 | Dynein heavy chain domain-containing protein 1 OS=Homo sapiens GN=DNHD1 PE=2 SV=2 | DNHD1 | 534 | 0.44 | 75% | 9% | 95% | 16% | 33% | 0 | 99% | 92% | 98% | 7% | 99% | 82% |
| 1399 | Non-histone chromosomal protein HMG-17 OS=Homo sapiens GN=HMGN2 PE=1 SV=3 | HMGN2 | 9 | 0.048 | 95% | 95% | 95% | 95% | 20% | 95% | 95% | 95% | 0 | 95% | 95% | 95% |
| 1400 | Delta-1-pyrroline-5-carboxylate synthase OS=Homo sapiens GN=ALDH18A1 PE=1 SV=2 | P5CS | 87 | 0.00088 | 0 | 95% | 0 | 0 | 95% | 99% | 100% | 100% | 100% | 95% | 100% | 100% |
| 1401 | Protein CYR61 OS=Homo sapiens GN=CYR61 PE=1 SV=1 | CYR61 | 42 | 0.49 | 0 | 0 | 0 | 100% | 99% | 88% | 100% | 100% | 89% | 97% | 96% | 100% |
| 1402 | Microtubule-associated proteins 1A/1B light chain 3B OS=Homo sapiens GN=MAP1LC3B PE=1 SV=3 | MLP3B (+1) | 15 | 0.024 | 100% | 100% | 100% | 15% | 100% | 14% | 100% | 86% | 100% | 0 | 0 | 95% |
| 1403 | Golgi SNAP receptor complex member 1 OS=Homo sapiens GN=GOSR1 PE=1 SV=1 | GOSR1 | 29 | 0.2 | 0 | 100% | 95% | 100% | 100% | 100% | 0 | 100% | 100% | 100% | 97% | 100% |
| 1404 | WD repeat and FYVE domain-containing protein 1 OS=Homo sapiens GN=WDFY1 PE=1 SV=1 | WDFY1 | 46 | 0.19 | 95% | 100% | 100% | 100% | 95% | 100% | 100% | 100% | 98% | 100% | 100% | 100% |
| 1405 | TATA-binding protein-associated factor 2N OS=Homo sapiens GN=TAF15 PE=1 SV=1 | RBP56 | 62 | 0.0079 | 0 | 100% | 95% | 0 | 100% | 100% | 92% | 100% | 100% | 100% | 100% | 100% |
| 1406 | Protein YIPF5 OS=Homo sapiens GN=YIPF5 PE=1 SV=1 | YIPF5 | 28 | 0.027 | 95% | 100% | 95% | 100% | 100% | 100% | 100% | 100% | 100% | 100% | 100% | 100% |
| 1407 | Cluster of Myosin regulatory light chain 12A OS=Homo sapiens GN=MYL12A PE=1 SV=2 (ML12A_HUMAN) | ML12A [3] | 20 | 0.59 | 100% | 100% | 95% | 100% | 100% | 100% | 100% | 95% | 100% | 100% | 94% | 95% |
| 1408 | Cluster of ATPase family AAA domain-containing protein 3A OS=Homo sapiens GN=ATAD3A PE=1 SV=2 (ATD3A_HUMAN) | ATD3A [2] | 71 | 0.0066 | 0 | 89% | 95% | 100% | 100% | 100% | 0 | 0 | 0 | 100% | 100% | 100% |
| 1409 | ATP synthase subunit g, mitochondrial OS=Homo sapiens GN=ATP5L PE=1 SV=3 | ATP5L | 11 | 0.08 | 50% | 100% | 0 | 95% | 100% | 100% | 95% | 100% | 100% | 100% | 100% | 100% |
| 1410 | Coatomer subunit delta OS=Homo sapiens GN=ARCN1 PE=1 SV=1 | COPD | 57 | 0.3 | 0 | 32% | 100% | 100% | 95% | 95% | 100% | 100% | 100% | 98% | 100% | 95% |
| 1411 | Phosphoglycerate mutase 1 OS=Homo sapiens GN=PGAM1 PE=1 SV=2 | PGAM1 | 29 | 0.0078 | 100% | 100% | 100% | 95% | 100% | 95% | 77% | 0 | 0 | 100% | 100% | 100% |
| 1412 | Sulfhydryl oxidase 1 OS=Homo sapiens GN=QSOX1 PE=1 SV=3 | QSOX1 | 83 | 0.22 | 95% | 100% | 100% | 0 | 100% | 100% | 100% | 100% | 100% | 95% | 95% | 0 |
| 1413 | Double-stranded RNA-specific editase 1 OS=Homo sapiens GN=ADARB1 PE=1 SV=1 | RED1 | 81 | < 0.00010 | 100% | 100% | 100% | 0 | 93% | 0 | 0 | 0 | 95% | 100% | 95% | 0 |
| 1414 | Protein transport protein Sec24D OS=Homo sapiens GN=SEC24D PE=1 SV=2 | SC24D | 113 | 0.16 | 0 | 100% | 0 | 95% | 92% | 100% | 100% | 100% | 100% | 100% | 100% | 50% |
| 1415 | ATP synthase subunit d, mitochondrial OS=Homo sapiens GN=ATP5H PE=1 SV=3 | ATP5H | 18 | 0.055 | 95% | 5% | 0 | 95% | 100% | 100% | 100% | 59% | 8% | 100% | 100% | 100% |
| 1416 | Developmentally-regulated GTP-binding protein 1 OS=Homo sapiens GN=DRG1 PE=1 SV=1 | DRG1 | 41 | 0.27 | 100% | 100% | 100% | 100% | 100% | 95% | 100% | 95% | 100% | 100% | 100% | 100% |
| 1417 | ATP-binding cassette sub-family D member 3 OS=Homo sapiens GN=ABCD3 PE=1 SV=1 | ABCD3 | 75 | 0.032 | 95% | 13% | 100% | 100% | 100% | 100% | 0 | 95% | 95% | 100% | 100% | 100% |
| 1418 | Signal peptidase complex subunit 3 OS=Homo sapiens GN=SPCS3 PE=1 SV=1 | SPCS3 | 20 | 0.67 | 41% | 100% | 100% | 97% | 100% | 100% | 95% | 100% | 100% | 100% | 95% | 100% |
| 1419 | Laminin subunit alpha-5 OS=Homo sapiens GN=LAMA5 PE=1 SV=8 | LAMA5 | 400 | < 0.00010 | 0 | 0 | 0 | 0 | 0 | 0 | 100% | 100% | 100% | 79% | 100% | 100% |
| 1420 | Microsomal glutathione S-transferase 1 OS=Homo sapiens GN=MGST1 PE=1 SV=1 | MGST1 | 18 | 0.0038 | 100% | 100% | 100% | 0 | 0 | 0 | 100% | 100% | 100% | 100% | 95% | 99% |
| 1421 | UDP-glucose 6-dehydrogenase OS=Homo sapiens GN=UGDH PE=1 SV=1 | UGDH | 55 | 0.049 | 95% | 100% | 100% | 0 | 34% | 95% | 100% | 100% | 100% | 0 | 95% | 0 |
| 1422 | Cleavage and polyadenylation specificity factor subunit 1 OS=Homo sapiens GN=CPSF1 PE=1 SV=2 | CPSF1 | 161 | 0.7 | 95% | 100% | 0 | 100% | 100% | 100% | 100% | 95% | 95% | 100% | 100% | 95% |
| 1423 | Coatomer subunit beta' OS=Homo sapiens GN=COPB2 PE=1 SV=2 | COPB2 | 102 | 0.26 | 95% | 95% | 100% | 13% | 100% | 100% | 100% | 99% | 100% | 0 | 95% | 95% |
| 1424 | ER lumen protein retaining receptor 3 OS=Homo sapiens GN=KDELR3 PE=2 SV=1 | ERD23 | 25 | 0.014 | 13% | 100% | 100% | 14% | 15% | 100% | 100% | 100% | 100% | 97% | 100% | 89% |
| 1425 | DnaJ homolog subfamily C member 3 OS=Homo sapiens GN=DNAJC3 PE=1 SV=1 | DNJC3 | 58 | 0.2 | 0 | 100% | 59% | 100% | 100% | 99% | 100% | 100% | 100% | 20% | 100% | 95% |
| 1426 | Bcl-2-like protein 13 OS=Homo sapiens GN=BCL2L13 PE=1 SV=1 | B2L13 | 53 | 0.0023 | 95% | 95% | 95% | 100% | 100% | 100% | 95% | 95% | 95% | 98% | 95% | 100% |
| 1427 | Charged multivesicular body protein 6 OS=Homo sapiens GN=CHMP6 PE=1 SV=3 | CHMP6 | 23 | 0.16 | 95% | 100% | 100% | 95% | 0 | 6% | 100% | 96% | 100% | 97% | 82% | 14% |
| 1428 | Prolyl 3-hydroxylase 3 OS=Homo sapiens GN=LEPREL2 PE=1 SV=1 | P3H3 | 82 | 0.15 | 95% | 100% | 95% | 0 | 95% | 99% | 100% | 100% | 100% | 60% | 62% | 100% |
| 1429 | Serine/arginine-rich splicing factor 11 OS=Homo sapiens GN=SRSF11 PE=1 SV=1 | SRS11 | 54 | 0.03 | 0 | 0 | 0 | 100% | 0 | 100% | 100% | 100% | 100% | 100% | 100% | 100% |
| 1430 | Transmembrane protein 87A OS=Homo sapiens GN=TMEM87A PE=1 SV=3 | TM87A | 63 | 0.054 | 95% | 95% | 100% | 0 | 100% | 100% | 100% | 100% | 95% | 0 | 95% | 100% |
| 1431 | AP-3 complex subunit beta-1 OS=Homo sapiens GN=AP3B1 PE=1 SV=3 | AP3B1 | 121 | 0.021 | 0 | 100% | 100% | 99% | 0 | 100% | 100% | 100% | 100% | 100% | 8% | 99% |
| 1432 | Histone H1.0 OS=Homo sapiens GN=H1F0 PE=1 SV=3 | H10 | 21 | 0.012 | 100% | 100% | 100% | 0 | 0 | 0 | 100% | 100% | 100% | 100% | 0 | 100% |
| 1433 | SAFB-like transcription modulator OS=Homo sapiens GN=SLTM PE=1 SV=2 | SLTM | 117 | 0.068 | 98% | 100% | 100% | 9% | 100% | 95% | 87% | 100% | 16% | 0 | 100% | 99% |
| 1434 | Alpha-1,3-mannosyl-glycoprotein 4-beta-N-acetylglucosaminyltransferase B OS=Homo sapiens GN=MGAT4B PE=1 SV=1 | MGT4B | 63 | 0.17 | 0 | 100% | 100% | 0 | 100% | 0 | 100% | 100% | 95% | 100% | 0 | 75% |
| 1435 | Serine/threonine-protein phosphatase 2A 65 kDa regulatory subunit A alpha isoform OS=Homo sapiens GN=PPP2R1A PE=1 SV=4 | 2AAA | 65 | 0.016 | 0 | 0 | 100% | 100% | 100% | 100% | 95% | 100% | 0 | 98% | 95% | 100% |
| 1436 | V-type proton ATPase subunit S1 OS=Homo sapiens GN=ATP6AP1 PE=1 SV=2 | VAS1 | 52 | 0.076 | 0 | 99% | 99% | 95% | 10% | 0 | 94% | 97% | 96% | 100% | 100% | 100% |
| 1437 | Protein jagunal homolog 1 OS=Homo sapiens GN=JAGN1 PE=1 SV=1 | JAGN1 | 21 | 0.094 | 21% | 100% | 100% | 95% | 100% | 0 | 95% | 98% | 95% | 100% | 100% | 100% |
| 1438 | Rab-like protein 3 OS=Homo sapiens GN=RABL3 PE=1 SV=1 | RABL3 | 26 | 0.12 | 0 | 100% | 100% | 100% | 100% | 100% | 95% | 0 | 20% | 100% | 0 | 95% |
| 1439 | Acyl-CoA:lysophosphatidylglycerol acyltransferase 1 OS=Homo sapiens GN=LPGAT1 PE=2 SV=1 | LGAT1 | 43 | 0.42 | 95% | 100% | 0 | 0 | 100% | 100% | 95% | 100% | 0 | 100% | 100% | 100% |
| 1440 | Metallo-beta-lactamase domain-containing protein 2 OS=Homo sapiens GN=MBLAC2 PE=1 SV=3 | MBLC2 | 31 | 0.046 | 95% | 0 | 100% | 95% | 100% | 100% | 0 | 0 | 0 | 100% | 100% | 100% |
| 1441 | SID1 transmembrane family member 2 OS=Homo sapiens GN=SIDT2 PE=1 SV=2 | SIDT2 | 94 | 0.0002 | 95% | 100% | 100% | 0 | 0 | 0 | 100% | 100% | 100% | 100% | 32% | 100% |
| 1442 | SPATS2-like protein OS=Homo sapiens GN=SPATS2L PE=1 SV=2 | SPS2L | 62 | 0.26 | 84% | 100% | 100% | 0 | 95% | 95% | 95% | 0 | 100% | 100% | 100% | 100% |
| 1443 | Serine/arginine repetitive matrix protein 1 OS=Homo sapiens GN=SRRM1 PE=1 SV=2 | SRRM1 | 102 | 0.047 | 0 | 94% | 95% | 0 | 0 | 95% | 99% | 100% | 99% | 100% | 100% | 100% |
| 1444 | Interferon-induced, double-stranded RNA-activated protein kinase OS=Homo sapiens GN=EIF2AK2 PE=1 SV=2 | E2AK2 | 62 | 0.18 | 92% | 95% | 95% | 100% | 95% | 95% | 100% | 95% | 97% | 95% | 100% | 99% |
| 1445 | Protein transport protein Sec24A OS=Homo sapiens GN=SEC24A PE=1 SV=2 | SC24A | 120 | 0.32 | 67% | 95% | 100% | 100% | 92% | 100% | 100% | 100% | 100% | 100% | 83% | 0 |
| 1446 | Derlin-1 OS=Homo sapiens GN=DERL1 PE=1 SV=1 | DERL1 | 29 | 0.2 | 100% | 95% | 95% | 95% | 95% | 100% | 100% | 100% | 100% | 95% | 100% | 100% |
| 1447 | Protein PRRC1 OS=Homo sapiens GN=PRRC1 PE=1 SV=1 | PRRC1 | 47 | 0.95 | 0 | 99% | 100% | 52% | 100% | 9% | 100% | 86% | 100% | 100% | 100% | 0 |
| 1448 | Enhancer of rudimentary homolog OS=Homo sapiens GN=ERH PE=1 SV=1 | ERH | 12 | 0.72 | 12% | 99% | 100% | 0 | 91% | 0 | 100% | 13% | 0 | 100% | 40% | 0 |
| 1449 | MLN64 N-terminal domain homolog OS=Homo sapiens GN=STARD3NL PE=1 SV=1 | MENTO | 27 | 0.068 | 100% | 100% | 95% | 52% | 100% | 100% | 100% | 100% | 100% | 43% | 0 | 0 |
| 1450 | Peptidyl-prolyl cis-trans isomerase C OS=Homo sapiens GN=PPIC PE=1 SV=1 | PPIC | 23 | 0.88 | 100% | 100% | 95% | 95% | 100% | 100% | 95% | 100% | 0 | 95% | 95% | 95% |
| 1451 | Nesprin-1 OS=Homo sapiens GN=SYNE1 PE=1 SV=4 | SYNE1 | 1011 | 0.59 | 61% | 56% | 100% | 0 | 10% | 100% | 94% | 47% | 89% | 43% | 60% | 95% |
| 1452 | Immediate early response 3-interacting protein 1 OS=Homo sapiens GN=IER3IP1 PE=1 SV=1 | IR3IP | 9 | 0.083 | 95% | 28% | 100% | 95% | 95% | 95% | 95% | 100% | 100% | 95% | 95% | 95% |
| 1453 | Dehydrogenase/reductase SDR family member 7 OS=Homo sapiens GN=DHRS7 PE=1 SV=1 | DHRS7 | 38 | 0.041 | 100% | 100% | 100% | 95% | 95% | 0 | 100% | 95% | 95% | 100% | 100% | 100% |
| 1454 | Lysosomal alpha-mannosidase OS=Homo sapiens GN=MAN2B1 PE=1 SV=3 | MA2B1 | 114 | 0.0072 | 95% | 95% | 59% | 95% | 0 | 95% | 0 | 100% | 0 | 100% | 100% | 100% |
| 1455 | Protein O-linked-mannose beta-1,2-N-acetylglucosaminyltransferase 1 OS=Homo sapiens GN=POMGNT1 PE=1 SV=2 | PMGT1 | 75 | 0.018 | 44% | 0 | 0 | 100% | 100% | 100% | 100% | 0 | 95% | 100% | 100% | 100% |
| 1456 | Retinol dehydrogenase 10 OS=Homo sapiens GN=RDH10 PE=1 SV=1 | RDH10 | 38 | 0.00064 | 100% | 100% | 100% | 0 | 0 | 0 | 100% | 52% | 65% | 0 | 0 | 0 |
| 1457 | Testis-expressed sequence 264 protein OS=Homo sapiens GN=TEX264 PE=1 SV=1 | TX264 | 34 | 0.54 | 95% | 100% | 95% | 0 | 0 | 100% | 100% | 99% | 100% | 95% | 100% | 100% |
| 1458 | Alpha-1,3/1,6-mannosyltransferase ALG2 OS=Homo sapiens GN=ALG2 PE=1 SV=1 | ALG2 | 47 | 0.81 | 95% | 95% | 100% | 95% | 100% | 100% | 95% | 100% | 100% | 95% | 100% | 100% |
| 1459 | Golgi-associated plant pathogenesis-related protein 1 OS=Homo sapiens GN=GLIPR2 PE=1 SV=3 | GAPR1 | 17 | 0.2 | 0 | 98% | 100% | 95% | 95% | 100% | 100% | 100% | 100% | 95% | 95% | 99% |
| 1460 | NHP2-like protein 1 OS=Homo sapiens GN=NHP2L1 PE=1 SV=3 | NH2L1 | 14 | 0.0078 | 18% | 95% | 100% | 0 | 95% | 0 | 100% | 100% | 100% | 95% | 100% | 100% |
| 1461 | Retinol dehydrogenase 14 OS=Homo sapiens GN=RDH14 PE=1 SV=1 | RDH14 | 37 | 0.057 | 0 | 100% | 63% | 7% | 65% | 100% | 63% | 98% | 100% | 100% | 100% | 100% |
| 1462 | Glycine--tRNA ligase OS=Homo sapiens GN=GARS PE=1 SV=3 | SYG | 83 | 0.74 | 25% | 0 | 100% | 95% | 95% | 99% | 100% | 95% | 100% | 100% | 25% | 100% |
| 1463 | Nucleolar protein 6 OS=Homo sapiens GN=NOL6 PE=1 SV=2 | NOL6 | 128 | 0.21 | 100% | 95% | 95% | 99% | 100% | 100% | 100% | 0 | 99% | 100% | 99% | 98% |
| 1464 | Nucleolar complex protein 2 homolog OS=Homo sapiens GN=NOC2L PE=1 SV=4 | NOC2L | 85 | 0.6 | 7% | 82% | 100% | 0 | 100% | 95% | 26% | 100% | 60% | 100% | 60% | 100% |
| 1465 | RNA-binding protein 25 OS=Homo sapiens GN=RBM25 PE=1 SV=3 | RBM25 | 100 | 0.053 | 0 | 95% | 95% | 30% | 0 | 89% | 100% | 100% | 100% | 98% | 98% | 100% |
| 1466 | H(+)/Cl(-) exchange transporter 7 OS=Homo sapiens GN=CLCN7 PE=1 SV=2 | CLCN7 | 89 | 0.34 | 100% | 100% | 100% | 31% | 95% | 100% | 0 | 95% | 0 | 100% | 99% | 100% |
| 1467 | Dehydrogenase/reductase SDR family member 7B OS=Homo sapiens GN=DHRS7B PE=1 SV=2 | DRS7B | 35 | 0.98 | 100% | 99% | 95% | 95% | 0 | 100% | 95% | 95% | 95% | 100% | 95% | 95% |
| 1468 | Q8N446-DECOY | Q8N446-DECOY | ? | 0.6 | 88% | 21% | 16% | 0 | 17% | 68% | 6% | 31% | 8% | 23% | 13% | 14% |
| 1469 | Probable rRNA-processing protein EBP2 OS=Homo sapiens GN=EBNA1BP2 PE=1 SV=2 | EBP2 | 35 | 0.0086 | 0 | 0 | 0 | 0 | 95% | 95% | 100% | 23% | 99% | 100% | 100% | 100% |
| 1470 | Golgi resident protein GCP60 OS=Homo sapiens GN=ACBD3 PE=1 SV=4 | GCP60 | 61 | 0.044 | 95% | 100% | 100% | 95% | 95% | 95% | 100% | 100% | 100% | 95% | 95% | 100% |
| 1471 | Exostosin-2 OS=Homo sapiens GN=EXT2 PE=1 SV=1 | EXT2 | 82 | 0.013 | 0 | 74% | 0 | 100% | 100% | 100% | 0 | 9% | 37% | 98% | 100% | 95% |
| 1472 | U4/U6 small nuclear ribonucleoprotein Prp3 OS=Homo sapiens GN=PRPF3 PE=1 SV=2 | PRPF3 | 78 | 0.0088 | 0 | 13% | 15% | 0 | 15% | 14% | 84% | 99% | 81% | 100% | 100% | 100% |
| 1473 | Peptidyl-prolyl cis-trans isomerase FKBP9 OS=Homo sapiens GN=FKBP9 PE=1 SV=2 | FKBP9 | 63 | 0.076 | 95% | 100% | 100% | 100% | 100% | 100% | 95% | 95% | 100% | 10% | 95% | 92% |
| 1474 | CDGSH iron-sulfur domain-containing protein 2 OS=Homo sapiens GN=CISD2 PE=1 SV=1 | CISD2 | 15 | 0.46 | 97% | 100% | 100% | 47% | 0 | 100% | 92% | 66% | 0 | 12% | 98% | 20% |
| 1475 | Lysosomal-associated transmembrane protein 4A OS=Homo sapiens GN=LAPTM4A PE=1 SV=1 | LAP4A | 27 | 0.085 | 0 | 0 | 100% | 0 | 0 | 95% | 100% | 100% | 100% | 100% | 100% | 95% |
| 1476 | Palmitoyltransferase ZDHHC13 OS=Homo sapiens GN=ZDHHC13 PE=1 SV=3 | ZDH13 | 71 | 0.16 | 99% | 95% | 95% | 100% | 0 | 95% | 99% | 100% | 100% | 95% | 86% | 95% |
| 1477 | Oxysterol-binding protein-related protein 5 OS=Homo sapiens GN=OSBPL5 PE=1 SV=1 | OSBL5 | 99 | 0.65 | 97% | 100% | 100% | 95% | 95% | 95% | 100% | 100% | 95% | 95% | 95% | 95% |
| 1478 | Vitamin K epoxide reductase complex subunit 1-like protein 1 OS=Homo sapiens GN=VKORC1L1 PE=1 SV=2 | VKORL | 20 | 0.061 | 100% | 98% | 100% | 100% | 100% | 95% | 98% | 80% | 100% | 10% | 99% | 8% |
| 1479 | P49750-DECOY | P49750-DECOY | ? | 0.6 | 45% | 0 | 0 | 0 | 74% | 97% | 5% | 42% | 0 | 81% | 99% | 90% |
| 1480 | Protein cornichon homolog 4 OS=Homo sapiens GN=CNIH4 PE=1 SV=1 | CNIH4 | 16 | 0.011 | 95% | 95% | 95% | 95% | 95% | 95% | 95% | 95% | 95% | 0 | 0 | 0 |
| 1481 | Abhydrolase domain-containing protein 16A OS=Homo sapiens GN=ABHD16A PE=1 SV=3 | ABHGA | 63 | 0.37 | 0 | 100% | 0 | 66% | 100% | 100% | 0 | 0 | 0 | 95% | 100% | 100% |
| 1482 | Dolichyl-phosphate beta-glucosyltransferase OS=Homo sapiens GN=ALG5 PE=1 SV=1 | ALG5 | 37 | 0.023 | 0 | 7% | 100% | 0 | 0 | 0 | 0 | 95% | 0 | 100% | 100% | 100% |
| 1483 | Gamma-glutamyl hydrolase OS=Homo sapiens GN=GGH PE=1 SV=2 | GGH | 36 | 0.0056 | 0 | 100% | 0 | 0 | 95% | 0 | 67% | 0 | 0 | 100% | 100% | 100% |
| 1484 | Leiomodin-1 OS=Homo sapiens GN=LMOD1 PE=1 SV=3 | LMOD1 | 67 | 0.0012 | 95% | 0 | 95% | 0 | 0 | 0 | 100% | 100% | 100% | 95% | 0 | 14% |
| 1485 | Protein THEM6 OS=Homo sapiens GN=THEM6 PE=1 SV=2 | THEM6 | 24 | 0.33 | 0 | 100% | 100% | 0 | 100% | 100% | 63% | 100% | 100% | 60% | 62% | 100% |
| 1486 | Thioredoxin-related transmembrane protein 2 OS=Homo sapiens GN=TMX2 PE=1 SV=1 | TMX2 | 34 | 0.015 | 100% | 100% | 100% | 100% | 100% | 100% | 95% | 0 | 95% | 95% | 0 | 0 |
| 1487 | Fibrillin-1 OS=Homo sapiens GN=FBN1 PE=1 SV=3 | FBN1 | 312 | 0.21 | 99% | 0 | 0 | 99% | 100% | 0 | 0 | 95% | 100% | 100% | 100% | 100% |
| 1488 | Galactosylgalactosylxylosylprotein 3-beta-glucuronosyltransferase 3 OS=Homo sapiens GN=B3GAT3 PE=1 SV=2 | B3GA3 | 37 | 0.94 | 95% | 100% | 95% | 0 | 100% | 100% | 95% | 100% | 57% | 0 | 100% | 100% |
| 1489 | Polypeptide N-acetylgalactosaminyltransferase 10 OS=Homo sapiens GN=GALNT10 PE=1 SV=2 | GLT10 | 69 | 0.42 | 0 | 26% | 0 | 95% | 0 | 100% | 0 | 100% | 100% | 97% | 0 | 100% |
| 1490 | Vacuolar protein sorting-associated protein 35 OS=Homo sapiens GN=VPS35 PE=1 SV=2 | VPS35 | 92 | 0.22 | 0 | 95% | 100% | 87% | 95% | 100% | 100% | 100% | 100% | 23% | 95% | 0 |
| 1491 | Serine/arginine repetitive matrix protein 2 OS=Homo sapiens GN=SRRM2 PE=1 SV=2 | SRRM2 | 300 | < 0.00010 | 0 | 53% | 100% | 0 | 0 | 0 | 0 | 0 | 0 | 100% | 98% | 100% |
| 1492 | U1 small nuclear ribonucleoprotein A OS=Homo sapiens GN=SNRPA PE=1 SV=3 | SNRPA | 31 | 0.94 | 10% | 100% | 100% | 0 | 100% | 94% | 86% | 100% | 10% | 99% | 100% | 89% |
| 1493 | 1-acyl-sn-glycerol-3-phosphate acyltransferase alpha OS=Homo sapiens GN=AGPAT1 PE=1 SV=2 | PLCA | 32 | 0.45 | 95% | 95% | 95% | 95% | 95% | 95% | 98% | 0 | 100% | 95% | 100% | 95% |
| 1494 | ER membrane protein complex subunit 3 OS=Homo sapiens GN=EMC3 PE=1 SV=3 | EMC3 | 30 | 0.48 | 95% | 100% | 95% | 95% | 100% | 0 | 98% | 95% | 0 | 100% | 100% | 100% |
| 1495 | Cleavage and polyadenylation specificity factor subunit 5 OS=Homo sapiens GN=NUDT21 PE=1 SV=1 | CPSF5 | 26 | 0.26 | 0 | 97% | 100% | 0 | 0 | 0 | 100% | 100% | 100% | 5% | 100% | 0 |
| 1496 | Phospholipase D3 OS=Homo sapiens GN=PLD3 PE=1 SV=1 | PLD3 | 55 | 0.71 | 95% | 95% | 100% | 0 | 100% | 100% | 95% | 100% | 95% | 95% | 95% | 100% |
| 1497 | E3 ubiquitin-protein ligase synoviolin OS=Homo sapiens GN=SYVN1 PE=1 SV=2 | SYVN1 | 68 | 0.12 | 0 | 100% | 100% | 15% | 98% | 0 | 99% | 98% | 95% | 100% | 98% | 90% |
| 1498 | Transmembrane protein 115 OS=Homo sapiens GN=TMEM115 PE=1 SV=1 | TM115 | 38 | 0.6 | 95% | 100% | 95% | 95% | 100% | 95% | 95% | 100% | 95% | 100% | 95% | 90% |
| 1499 | Transmembrane protein 205 OS=Homo sapiens GN=TMEM205 PE=1 SV=1 | TM205 | 21 | 0.5 | 95% | 100% | 100% | 100% | 100% | 95% | 0 | 95% | 95% | 95% | 100% | 100% |
| 1500 | DNA-directed RNA polymerases I and III subunit RPAC1 OS=Homo sapiens GN=POLR1C PE=1 SV=1 | RPAC1 | 39 | 0.81 | 95% | 95% | 95% | 95% | 95% | 95% | 95% | 95% | 95% | 0 | 95% | 100% |
| 1501 | Eukaryotic translation initiation factor 4B OS=Homo sapiens GN=EIF4B PE=1 SV=2 | IF4B | 69 | 0.67 | 0 | 95% | 100% | 0 | 95% | 95% | 0 | 100% | 99% | 95% | 95% | 97% |
| 1502 | Very-long-chain (3R)-3-hydroxyacyl-[acyl-carrier protein] dehydratase 2 OS=Homo sapiens GN=PTPLB PE=1 SV=1 | HACD2 | 28 | 0.053 | 0 | 95% | 95% | 0 | 34% | 95% | 0 | 0 | 0 | 99% | 95% | 95% |
| 1503 | Beta-1,4-galactosyltransferase 4 OS=Homo sapiens GN=B4GALT4 PE=1 SV=1 | B4GT4 | 40 | 0.56 | 95% | 100% | 100% | 95% | 95% | 100% | 95% | 99% | 95% | 95% | 95% | 99% |
| 1504 | Coiled-coil domain-containing protein 80 OS=Homo sapiens GN=CCDC80 PE=1 SV=1 | CCD80 | 108 | 0.042 | 100% | 100% | 100% | 0 | 0 | 0 | 99% | 0 | 0 | 100% | 95% | 100% |
| 1505 | Carbohydrate sulfotransferase 14 OS=Homo sapiens GN=CHST14 PE=1 SV=2 | CHSTE | 43 | 0.5 | 95% | 77% | 95% | 100% | 99% | 100% | 100% | 100% | 0 | 0 | 100% | 99% |
| 1506 | Pre-mRNA-processing factor 40 homolog A OS=Homo sapiens GN=PRPF40A PE=1 SV=2 | PR40A | 109 | 0.18 | 95% | 95% | 100% | 95% | 40% | 0 | 100% | 100% | 95% | 100% | 10% | 95% |
| 1507 | RNA-binding protein with serine-rich domain 1 OS=Homo sapiens GN=RNPS1 PE=1 SV=1 | RNPS1 | 34 | 0.31 | 100% | 100% | 95% | 0 | 0 | 0 | 100% | 95% | 100% | 100% | 95% | 0 |
| 1508 | Ribonuclease inhibitor OS=Homo sapiens GN=RNH1 PE=1 SV=2 | RINI | 50 | 0.67 | 6% | 100% | 100% | 0 | 0 | 95% | 95% | 100% | 75% | 95% | 100% | 0 |
| 1509 | YLP motif-containing protein 1 OS=Homo sapiens GN=YLPM1 PE=1 SV=3 | YLPM1 | 220 | 0.47 | 10% | 100% | 100% | 0 | 0 | 95% | 95% | 99% | 0 | 100% | 100% | 0 |
| 1510 | Pyruvate dehydrogenase E1 component subunit beta, mitochondrial OS=Homo sapiens GN=PDHB PE=1 SV=3 | ODPB | 39 | 0.029 | 95% | 95% | 95% | 95% | 0 | 95% | 100% | 95% | 100% | 100% | 100% | 95% |
| 1511 | 3-ketodihydrosphingosine reductase OS=Homo sapiens GN=KDSR PE=1 SV=1 | KDSR | 36 | 0.0057 | 0 | 95% | 100% | 95% | 100% | 100% | 95% | 95% | 100% | 0 | 0 | 0 |
| 1512 | Importin subunit alpha-1 OS=Homo sapiens GN=KPNA2 PE=1 SV=1 | IMA1 | 58 | 0.00051 | 0 | 0 | 0 | 100% | 100% | 100% | 100% | 100% | 100% | 100% | 100% | 95% |
| 1513 | Transmembrane 9 superfamily member 1 OS=Homo sapiens GN=TM9SF1 PE=2 SV=2 | TM9S1 | 69 | 0.95 | 95% | 95% | 95% | 95% | 100% | 0 | 0 | 98% | 100% | 100% | 100% | 95% |
| 1514 | Chloride channel CLIC-like protein 1 OS=Homo sapiens GN=CLCC1 PE=1 SV=1 | CLCC1 | 62 | 0.64 | 0 | 90% | 0 | 68% | 100% | 66% | 100% | 0 | 0 | 100% | 100% | 0 |
| 1515 | Histone H2A.V OS=Homo sapiens GN=H2AFV PE=1 SV=3 | H2AV | 14 | 0.064 | 100% | 100% | 100% | 80% | 100% | 100% | 0 | 0 | 100% | 100% | 100% | 100% |
| 1516 | Uveal autoantigen with coiled-coil domains and ankyrin repeats OS=Homo sapiens GN=UACA PE=1 SV=2 | UACA | 163 | 0.32 | 100% | 100% | 0 | 0 | 0 | 0 | 100% | 0 | 95% | 0 | 0 | 99% |
| 1517 | Splicing factor 3B subunit 6 OS=Homo sapiens GN=SF3B6 PE=1 SV=1 | SF3B6 | 15 | 0.95 | 95% | 0 | 100% | 0 | 95% | 95% | 95% | 95% | 100% | 100% | 19% | 100% |
| 1518 | Peroxisomal acyl-coenzyme A oxidase 3 OS=Homo sapiens GN=ACOX3 PE=1 SV=2 | ACOX3 | 78 | 0.078 | 95% | 12% | 95% | 0 | 95% | 0 | 100% | 100% | 100% | 95% | 95% | 100% |
| 1519 | mRNA turnover protein 4 homolog OS=Homo sapiens GN=MRTO4 PE=1 SV=2 | MRT4 | 28 | 0.12 | 86% | 95% | 0 | 98% | 0 | 95% | 100% | 100% | 100% | 100% | 100% | 100% |
| 1520 | Small integral membrane protein 13 OS=Homo sapiens GN=SMIM13 PE=3 SV=1 | SIM13 | 10 | 0.19 | 0 | 100% | 100% | 0 | 0 | 13% | 95% | 100% | 100% | 91% | 99% | 100% |
| 1521 | Ubiquitin carboxyl-terminal hydrolase 49 OS=Homo sapiens GN=USP49 PE=1 SV=1 | UBP49 | 79 | 0.7 | 22% | 100% | 0 | 30% | 20% | 95% | 10% | 5% | 0 | 95% | 14% | 82% |
| 1522 | P35527-DECOY | P35527-DECOY | ? | 0.53 | 95% | 95% | 95% | 95% | 95% | 99% | 95% | 95% | 95% | 95% | 0 | 95% |
| 1523 | Small nuclear ribonucleoprotein G-like protein OS=Homo sapiens PE=3 SV=2 | RUXGL (+1) | 9 | 0.76 | 97% | 95% | 95% | 95% | 95% | 95% | 0 | 95% | 95% | 95% | 95% | 95% |
| 1524 | Thioredoxin-related transmembrane protein 4 OS=Homo sapiens GN=TMX4 PE=1 SV=1 | TMX4 | 39 | 0.2 | 95% | 95% | 95% | 95% | 95% | 95% | 95% | 95% | 0 | 95% | 95% | 95% |
| 1525 | Arylsulfatase A OS=Homo sapiens GN=ARSA PE=1 SV=3 | ARSA | 54 | 0.92 | 95% | 100% | 100% | 0 | 95% | 95% | 95% | 95% | 0 | 100% | 100% | 95% |
| 1526 | Glycerol-3-phosphate acyltransferase 4 OS=Homo sapiens GN=AGPAT6 PE=1 SV=1 | GPAT4 | 52 | 0.11 | 0 | 0 | 0 | 0 | 94% | 0 | 100% | 100% | 0 | 98% | 98% | 0 |
| 1527 | HEAT repeat-containing protein 1 OS=Homo sapiens GN=HEATR1 PE=1 SV=3 | HEAT1 | 242 | < 0.00010 | 0 | 0 | 16% | 0 | 0 | 0 | 97% | 73% | 99% | 100% | 100% | 100% |
| 1528 | Lysosomal alpha-glucosidase OS=Homo sapiens GN=GAA PE=1 SV=4 | LYAG | 105 | 0.016 | 95% | 100% | 100% | 0 | 0 | 0 | 0 | 100% | 84% | 0 | 95% | 0 |
| 1529 | NADH dehydrogenase [ubiquinone] iron-sulfur protein 2, mitochondrial OS=Homo sapiens GN=NDUFS2 PE=1 SV=2 | NDUS2 | 53 | 0.0015 | 0 | 0 | 87% | 100% | 100% | 100% | 0 | 0 | 0 | 100% | 100% | 100% |
| 1530 | Reticulocalbin-3 OS=Homo sapiens GN=RCN3 PE=1 SV=1 | RCN3 | 37 | 0.35 | 0 | 100% | 100% | 0 | 0 | 0 | 99% | 100% | 95% | 0 | 0 | 0 |
| 1531 | Carbohydrate sulfotransferase 3 OS=Homo sapiens GN=CHST3 PE=1 SV=3 | CHST3 | 55 | 0.013 | 0 | 0 | 6% | 100% | 100% | 100% | 100% | 95% | 100% | 0 | 0 | 95% |
| 1532 | Zinc finger CCCH domain-containing protein 14 OS=Homo sapiens GN=ZC3H14 PE=1 SV=1 | ZC3HE | 83 | 0.043 | 0 | 100% | 0 | 0 | 0 | 0 | 0 | 95% | 95% | 100% | 100% | 100% |
| 1533 | Cytochrome P450 20A1 OS=Homo sapiens GN=CYP20A1 PE=2 SV=1 | CP20A | 52 | 0.064 | 0 | 23% | 100% | 0 | 11% | 52% | 0 | 100% | 14% | 100% | 100% | 95% |
| 1534 | THO complex subunit 2 OS=Homo sapiens GN=THOC2 PE=1 SV=2 | THOC2 | 183 | 0.14 | 100% | 95% | 100% | 0 | 77% | 0 | 95% | 95% | 100% | 100% | 100% | 100% |
| 1535 | Ribosome biogenesis protein BOP1 OS=Homo sapiens GN=BOP1 PE=1 SV=2 | BOP1 | 84 | 0.048 | 0 | 95% | 100% | 0 | 0 | 95% | 100% | 100% | 100% | 95% | 55% | 0 |
| 1536 | Squalene synthase OS=Homo sapiens GN=FDFT1 PE=1 SV=1 | FDFT | 48 | 0.0029 | 0 | 0 | 0 | 0 | 95% | 100% | 100% | 100% | 100% | 0 | 100% | 95% |
| 1537 | Cleavage and polyadenylation specificity factor subunit 7 OS=Homo sapiens GN=CPSF7 PE=1 SV=1 | CPSF7 | 52 | 0.79 | 0 | 95% | 100% | 100% | 95% | 95% | 95% | 100% | 100% | 100% | 0 | 100% |
| 1538 | cTAGE family member 5 OS=Homo sapiens GN=CTAGE5 PE=1 SV=4 | CTGE5 | 91 | 0.11 | 100% | 100% | 100% | 95% | 100% | 95% | 0 | 98% | 0 | 0 | 75% | 100% |
| 1539 | Bifunctional glutamate/proline--tRNA ligase OS=Homo sapiens GN=EPRS PE=1 SV=5 | SYEP | 171 | 0.6 | 0 | 93% | 100% | 0 | 92% | 0 | 10% | 95% | 80% | 60% | 62% | 100% |
| 1540 | Transaldolase OS=Homo sapiens GN=TALDO1 PE=1 SV=2 | TALDO | 38 | 0.0089 | 11% | 95% | 85% | 0 | 0 | 0 | 0 | 0 | 0 | 95% | 99% | 100% |
| 1541 | Nebulin OS=Homo sapiens GN=NEB PE=1 SV=5 | NEBU | 773 | 0.44 | 50% | 93% | 56% | 0 | 0 | 0 | 0 | 0 | 71% | 0 | 23% | 100% |
| 1542 | Q86XK2-DECOY | Q86XK2-DECOY | ? | 0.44 | 33% | 75% | 50% | 0 | 0 | 75% | 95% | 20% | 82% | 34% | 0 | 13% |
| 1543 | ATPase ASNA1 OS=Homo sapiens GN=ASNA1 PE=1 SV=2 | ASNA | 39 | 0.16 | 0 | 0 | 100% | 95% | 100% | 67% | 100% | 100% | 100% | 58% | 95% | 0 |
| 1544 | Chitinase domain-containing protein 1 OS=Homo sapiens GN=CHID1 PE=1 SV=1 | CHID1 | 45 | 0.0021 | 0 | 95% | 95% | 0 | 81% | 100% | 0 | 0 | 0 | 100% | 100% | 100% |
| 1545 | Superkiller viralicidic activity 2-like 2 OS=Homo sapiens GN=SKIV2L2 PE=1 SV=3 | SK2L2 | 118 | 0.3 | 0 | 95% | 95% | 0 | 95% | 97% | 100% | 100% | 95% | 98% | 95% | 95% |
| 1546 | Non-specific lipid-transfer protein OS=Homo sapiens GN=SCP2 PE=1 SV=2 | NLTP | 59 | 0.2 | 0 | 19% | 98% | 0 | 92% | 100% | 0 | 0 | 0 | 100% | 100% | 100% |
| 1547 | E3 ubiquitin-protein ligase AMFR OS=Homo sapiens GN=AMFR PE=1 SV=2 | AMFR | 73 | 0.00058 | 0 | 0 | 0 | 100% | 100% | 100% | 0 | 0 | 0 | 99% | 100% | 100% |
| 1548 | Alpha-N-acetylglucosaminidase OS=Homo sapiens GN=NAGLU PE=1 SV=2 | ANAG | 82 | 0.016 | 100% | 100% | 95% | 95% | 0 | 0 | 0 | 0 | 0 | 100% | 100% | 100% |
| 1549 | RNA-binding protein FUS OS=Homo sapiens GN=FUS PE=1 SV=1 | FUS | 53 | 0.49 | 100% | 100% | 0 | 0 | 5% | 100% | 32% | 0 | 100% | 100% | 11% | 11% |
| 1550 | LEM domain-containing protein 2 OS=Homo sapiens GN=LEMD2 PE=1 SV=1 | LEMD2 | 57 | 0.0005 | 0 | 100% | 0 | 100% | 100% | 100% | 0 | 100% | 0 | 0 | 0 | 0 |
| 1551 | Heat shock 70 kDa protein 13 OS=Homo sapiens GN=HSPA13 PE=1 SV=1 | HSP13 | 52 | 0.0048 | 9% | 95% | 95% | 95% | 100% | 100% | 0 | 0 | 0 | 100% | 100% | 95% |
| 1552 | Tumor protein D54 OS=Homo sapiens GN=TPD52L2 PE=1 SV=2 | TPD54 | 22 | 0.021 | 0 | 100% | 95% | 95% | 95% | 100% | 100% | 99% | 98% | 0 | 0 | 0 |
| 1553 | Mammalian ependymin-related protein 1 OS=Homo sapiens GN=EPDR1 PE=1 SV=2 | EPDR1 | 25 | 0.027 | 7% | 95% | 100% | 0 | 95% | 95% | 100% | 13% | 88% | 100% | 100% | 100% |
| 1554 | Mitochondrial fission 1 protein OS=Homo sapiens GN=FIS1 PE=1 SV=2 | FIS1 | 17 | 0.0057 | 95% | 100% | 82% | 95% | 95% | 95% | 81% | 55% | 23% | 100% | 100% | 100% |
| 1555 | Ubiquitin-associated domain-containing protein 2 OS=Homo sapiens GN=UBAC2 PE=2 SV=1 | UBAC2 | 39 | 0.0071 | 0 | 95% | 95% | 95% | 100% | 100% | 100% | 0 | 0 | 95% | 21% | 0 |
| 1556 | Xylosyltransferase 2 OS=Homo sapiens GN=XYLT2 PE=2 SV=2 | XYLT2 | 97 | 0.23 | 95% | 80% | 99% | 21% | 95% | 88% | 26% | 95% | 100% | 100% | 97% | 98% |
| 1557 | Fibroblast growth factor 2 OS=Homo sapiens GN=FGF2 PE=1 SV=3 | FGF2 | 31 | 0.041 | 100% | 100% | 100% | 100% | 98% | 93% | 0 | 0 | 0 | 72% | 81% | 0 |
| 1558 | Signal-induced proliferation-associated protein 1 OS=Homo sapiens GN=SIPA1 PE=1 SV=1 | SIPA1 | 112 | 0.6 | 0 | 0 | 0 | 0 | 100% | 13% | 100% | 6% | 26% | 0 | 80% | 11% |
| 1559 | Protein Daple OS=Homo sapiens GN=CCDC88C PE=1 SV=3 | DAPLE | 228 | 0.44 | 5% | 63% | 40% | 99% | 0 | 11% | 0 | 7% | 0 | 11% | 93% | 18% |
| 1560 | Derlin-2 OS=Homo sapiens GN=DERL2 PE=1 SV=1 | DERL2 | 28 | 0.26 | 95% | 95% | 95% | 95% | 95% | 95% | 95% | 95% | 95% | 96% | 95% | 95% |
| 1561 | Charged multivesicular body protein 1b OS=Homo sapiens GN=CHMP1B PE=1 SV=1 | CHM1B | 22 | 0.59 | 24% | 92% | 43% | 0 | 34% | 49% | 22% | 25% | 95% | 95% | 34% | 21% |
| 1562 | Cleavage stimulation factor subunit 3 OS=Homo sapiens GN=CSTF3 PE=1 SV=1 | CSTF3 | 83 | 0.0091 | 0 | 0 | 0 | 0 | 0 | 0 | 95% | 0 | 100% | 100% | 100% | 100% |
| 1563 | Polynucleotide 5'-hydroxyl-kinase NOL9 OS=Homo sapiens GN=NOL9 PE=1 SV=1 | NOL9 | 79 | 0.0064 | 0 | 0 | 0 | 12% | 0 | 0 | 100% | 100% | 100% | 100% | 100% | 95% |
| 1564 | Periostin OS=Homo sapiens GN=POSTN PE=1 SV=2 | POSTN | 93 | < 0.00010 | 0 | 0 | 0 | 0 | 0 | 0 | 100% | 100% | 100% | 0 | 0 | 0 |
| 1565 | Sequestosome-1 OS=Homo sapiens GN=SQSTM1 PE=1 SV=1 | SQSTM | 48 | 0.064 | 95% | 95% | 100% | 0 | 95% | 0 | 100% | 100% | 95% | 95% | 0 | 0 |
| 1566 | Cytochrome c1, heme protein, mitochondrial OS=Homo sapiens GN=CYC1 PE=1 SV=3 | CY1 | 35 | 0.00063 | 95% | 0 | 100% | 100% | 100% | 100% | 0 | 0 | 0 | 95% | 0 | 0 |
| 1567 | Heparan sulfate 2-O-sulfotransferase 1 OS=Homo sapiens GN=HS2ST1 PE=1 SV=1 | HS2ST | 42 | 0.34 | 100% | 0 | 100% | 43% | 0 | 95% | 8% | 51% | 10% | 100% | 0 | 95% |
| 1568 | Galectin-3-binding protein OS=Homo sapiens GN=LGALS3BP PE=1 SV=1 | LG3BP | 65 | 0.042 | 95% | 100% | 95% | 95% | 0 | 0 | 0 | 0 | 0 | 100% | 100% | 100% |
| 1569 | Splicing factor 3A subunit 2 OS=Homo sapiens GN=SF3A2 PE=1 SV=2 | SF3A2 | 49 | 0.48 | 0 | 100% | 0 | 0 | 0 | 0 | 98% | 100% | 0 | 100% | 100% | 0 |
| 1570 | Mannose-P-dolichol utilization defect 1 protein OS=Homo sapiens GN=MPDU1 PE=1 SV=2 | MPU1 | 27 | 0.18 | 95% | 95% | 95% | 95% | 0 | 95% | 95% | 97% | 95% | 95% | 100% | 100% |
| 1571 | Transcriptional activator protein Pur-beta OS=Homo sapiens GN=PURB PE=1 SV=3 | PURB | 33 | 0.22 | 0 | 99% | 100% | 0 | 0 | 0 | 100% | 37% | 98% | 100% | 100% | 100% |
| 1572 | 5'-3' exoribonuclease 2 OS=Homo sapiens GN=XRN2 PE=1 SV=1 | XRN2 | 109 | 0.43 | 95% | 95% | 95% | 0 | 0 | 100% | 45% | 100% | 95% | 100% | 100% | 95% |
| 1573 | Glycosyltransferase 8 domain-containing protein 2 OS=Homo sapiens GN=GLT8D2 PE=2 SV=1 | GL8D2 | 40 | < 0.00010 | 0 | 0 | 0 | 100% | 100% | 100% | 0 | 0 | 0 | 100% | 100% | 95% |
| 1574 | Protein MAK16 homolog OS=Homo sapiens GN=MAK16 PE=1 SV=2 | MAK16 | 35 | 0.37 | 0 | 95% | 95% | 0 | 100% | 95% | 95% | 95% | 100% | 100% | 95% | 95% |
| 1575 | Phosphatidylserine synthase 1 OS=Homo sapiens GN=PTDSS1 PE=1 SV=1 | PTSS1 | 56 | 0.036 | 0 | 95% | 0 | 95% | 100% | 100% | 100% | 100% | 100% | 95% | 0 | 10% |
| 1576 | F-actin-capping protein subunit alpha-2 OS=Homo sapiens GN=CAPZA2 PE=1 SV=3 | CAZA2 | 33 | 0.029 | 63% | 100% | 100% | 100% | 100% | 96% | 100% | 100% | 100% | 0 | 0 | 98% |
| 1577 | Delta(24)-sterol reductase OS=Homo sapiens GN=DHCR24 PE=1 SV=2 | DHC24 | 60 | 0.02 | 0 | 0 | 0 | 100% | 95% | 0 | 92% | 95% | 100% | 97% | 97% | 95% |
| 1578 | Elongation factor 1-beta OS=Homo sapiens GN=EEF1B2 PE=1 SV=3 | EF1B | 25 | 0.00055 | 0 | 56% | 49% | 0 | 12% | 0 | 100% | 95% | 100% | 7% | 88% | 53% |
| 1579 | THO complex subunit 6 homolog OS=Homo sapiens GN=THOC6 PE=1 SV=1 | THOC6 | 38 | 0.14 | 95% | 95% | 95% | 95% | 0 | 0 | 95% | 100% | 100% | 95% | 0 | 100% |
| 1580 | Serine/threonine-protein phosphatase PGAM5, mitochondrial OS=Homo sapiens GN=PGAM5 PE=1 SV=2 | PGAM5 | 32 | 0.44 | 0 | 25% | 95% | 7% | 0 | 0 | 0 | 0 | 0 | 100% | 100% | 89% |
| 1581 | WD repeat-containing protein 18 OS=Homo sapiens GN=WDR18 PE=1 SV=2 | WDR18 | 47 | 1 | 0 | 94% | 100% | 49% | 99% | 95% | 95% | 100% | 0 | 32% | 95% | 6% |
| 1582 | Galectin-8 OS=Homo sapiens GN=LGALS8 PE=1 SV=4 | LEG8 | 36 | 0.0088 | 23% | 15% | 52% | 0 | 100% | 100% | 0 | 0 | 0 | 100% | 97% | 95% |
| 1583 | Lupus La protein OS=Homo sapiens GN=SSB PE=1 SV=2 | LA | 47 | 0.34 | 95% | 95% | 88% | 99% | 95% | 95% | 95% | 14% | 95% | 95% | 95% | 95% |
| 1584 | Class E basic helix-loop-helix protein 22 OS=Homo sapiens GN=BHLHE22 PE=2 SV=1 | BHE22 | 37 | 0.44 | 0 | 0 | 0 | 0 | 0 | 98% | 56% | 0 | 0 | 0 | 0 | 0 |
| 1585 | Cluster of Ras-related GTP-binding protein C OS=Homo sapiens GN=RRAGC PE=1 SV=1 (RRAGC_HUMAN) | RRAGC [2] | 44 | 0.014 | 0 | 95% | 100% | 0 | 0 | 0 | 100% | 100% | 100% | 100% | 93% | 99% |
| 1586 | Cleavage and polyadenylation specificity factor subunit 2 OS=Homo sapiens GN=CPSF2 PE=1 SV=2 | CPSF2 | 88 | 0.048 | 0 | 95% | 0 | 0 | 0 | 0 | 0 | 95% | 0 | 98% | 100% | 100% |
| 1587 | Poly [ADP-ribose] polymerase 14 OS=Homo sapiens GN=PARP14 PE=1 SV=3 | PAR14 | 203 | 0.33 | 0 | 99% | 95% | 0 | 95% | 0 | 0 | 81% | 0 | 32% | 100% | 100% |
| 1588 | Serine/threonine-protein kinase 11-interacting protein OS=Homo sapiens GN=STK11IP PE=1 SV=3 | S11IP | 121 | 0.00077 | 0 | 0 | 95% | 0 | 0 | 0 | 100% | 100% | 100% | 0 | 0 | 99% |
| 1589 | Protein OS-9 OS=Homo sapiens GN=OS9 PE=1 SV=1 | OS9 | 76 | 0.0028 | 100% | 100% | 100% | 15% | 95% | 14% | 0 | 0 | 0 | 0 | 13% | 14% |
| 1590 | Gamma-interferon-inducible protein 16 OS=Homo sapiens GN=IFI16 PE=1 SV=3 | IF16 | 88 | 0.94 | 100% | 86% | 95% | 0 | 95% | 100% | 95% | 66% | 95% | 95% | 0 | 95% |
| 1591 | RNA-binding protein 10 OS=Homo sapiens GN=RBM10 PE=1 SV=3 | RBM10 | 104 | 0.2 | 0 | 95% | 95% | 95% | 0 | 0 | 100% | 100% | 95% | 100% | 0 | 100% |
| 1592 | Soluble calcium-activated nucleotidase 1 OS=Homo sapiens GN=CANT1 PE=1 SV=1 | CANT1 | 45 | 0.0093 | 6% | 100% | 0 | 0 | 0 | 0 | 100% | 100% | 100% | 0 | 0 | 95% |
| 1593 | U4/U6 small nuclear ribonucleoprotein Prp31 OS=Homo sapiens GN=PRPF31 PE=1 SV=2 | PRP31 | 55 | 0.061 | 95% | 99% | 95% | 0 | 0 | 0 | 100% | 100% | 0 | 26% | 0 | 25% |
| 1594 | Serine/arginine-rich splicing factor 5 OS=Homo sapiens GN=SRSF5 PE=1 SV=1 | SRSF5 | 31 | 0.56 | 77% | 100% | 100% | 0 | 56% | 99% | 60% | 100% | 0 | 100% | 100% | 100% |
| 1595 | Electron transfer flavoprotein-ubiquinone oxidoreductase, mitochondrial OS=Homo sapiens GN=ETFDH PE=1 SV=2 | ETFD | 68 | 0.0029 | 0 | 0 | 0 | 0 | 0 | 0 | 14% | 0 | 0 | 100% | 100% | 100% |
| 1596 | Protein mago nashi homolog 2 OS=Homo sapiens GN=MAGOHB PE=1 SV=1 | MGN2 (+1) | 17 | 0.11 | 0 | 100% | 100% | 0 | 0 | 0 | 15% | 0 | 0 | 100% | 0 | 100% |
| 1597 | ATP-binding cassette sub-family F member 2 OS=Homo sapiens GN=ABCF2 PE=1 SV=2 | ABCF2 | 71 | 0.41 | 0 | 0 | 95% | 95% | 26% | 95% | 95% | 100% | 80% | 100% | 99% | 95% |
| 1598 | DNA damage-binding protein 1 OS=Homo sapiens GN=DDB1 PE=1 SV=1 | DDB1 | 127 | 0.19 | 80% | 95% | 100% | 0 | 0 | 95% | 100% | 95% | 100% | 100% | 95% | 0 |
| 1599 | Serine hydroxymethyltransferase, mitochondrial OS=Homo sapiens GN=SHMT2 PE=1 SV=3 | GLYM | 56 | 0.41 | 0 | 100% | 95% | 95% | 95% | 9% | 13% | 15% | 29% | 49% | 95% | 100% |
| 1600 | Xyloside xylosyltransferase 1 OS=Homo sapiens GN=XXYLT1 PE=1 SV=1 | XXLT1 | 44 | 0.44 | 0 | 95% | 95% | 0 | 95% | 95% | 0 | 95% | 95% | 100% | 100% | 100% |
| 1601 | Nucleosome assembly protein 1-like 1 OS=Homo sapiens GN=NAP1L1 PE=1 SV=1 | NP1L1 | 45 | 0.56 | 0 | 100% | 0 | 95% | 95% | 100% | 95% | 100% | 95% | 95% | 95% | 95% |
| 1602 | Arylsulfatase B OS=Homo sapiens GN=ARSB PE=1 SV=1 | ARSB | 60 | 0.49 | 100% | 100% | 95% | 0 | 58% | 75% | 100% | 0 | 95% | 0 | 100% | 100% |
| 1603 | Prenylcysteine oxidase-like OS=Homo sapiens GN=PCYOX1L PE=1 SV=2 | PCYXL | 55 | 0.61 | 0 | 0 | 95% | 0 | 16% | 100% | 0 | 100% | 0 | 100% | 100% | 100% |
| 1604 | KDEL motif-containing protein 2 OS=Homo sapiens GN=KDELC2 PE=1 SV=2 | KDEL2 | 59 | 0.51 | 95% | 0 | 95% | 0 | 100% | 95% | 95% | 95% | 95% | 0 | 100% | 7% |
| 1605 | U6 snRNA-associated Sm-like protein LSm3 OS=Homo sapiens GN=LSM3 PE=1 SV=2 | LSM3 | 12 | 0.35 | 0 | 0 | 99% | 0 | 0 | 100% | 63% | 95% | 100% | 100% | 95% | 100% |
| 1606 | Probable ATP-dependent RNA helicase DDX46 OS=Homo sapiens GN=DDX46 PE=1 SV=2 | DDX46 | 117 | 0.45 | 99% | 0 | 0 | 0 | 0 | 99% | 100% | 95% | 95% | 100% | 100% | 100% |
| 1607 | Tumor necrosis factor alpha-induced protein 2 OS=Homo sapiens GN=TNFAIP2 PE=1 SV=2 | TNAP2 | 73 | 0.074 | 95% | 95% | 95% | 95% | 95% | 95% | 95% | 95% | 95% | 100% | 98% | 95% |
| 1608 | Vitamin K epoxide reductase complex subunit 1 OS=Homo sapiens GN=VKORC1 PE=1 SV=1 | VKOR1 | 18 | 0.44 | 95% | 95% | 95% | 95% | 95% | 95% | 95% | 95% | 95% | 95% | 95% | 0 |
| 1609 | Fibronectin type-III domain-containing protein 3A OS=Homo sapiens GN=FNDC3A PE=1 SV=4 | FND3A | 132 | 0.049 | 0 | 94% | 9% | 0 | 43% | 10% | 0 | 0 | 0 | 100% | 95% | 100% |
| 1610 | KH domain-containing, RNA-binding, signal transduction-associated protein 3 OS=Homo sapiens GN=KHDRBS3 PE=1 SV=1 | KHDR3 | 39 | 0.026 | 0 | 97% | 0 | 98% | 96% | 28% | 0 | 0 | 0 | 100% | 100% | 100% |
| 1611 | Lysosomal protein NCU-G1 OS=Homo sapiens GN=C1orf85 PE=2 SV=1 | NCUG1 | 44 | 0.0093 | 95% | 95% | 95% | 0 | 12% | 0 | 100% | 100% | 100% | 0 | 0 | 100% |
| 1612 | SWI/SNF complex subunit SMARCC2 OS=Homo sapiens GN=SMARCC2 PE=1 SV=1 | SMRC2 | 133 | < 0.00010 | 25% | 0 | 0 | 0 | 0 | 0 | 0 | 0 | 0 | 100% | 100% | 100% |
| 1613 | Glutaminyl-peptide cyclotransferase-like protein OS=Homo sapiens GN=QPCTL PE=1 SV=2 | QPCTL | 43 | 0.00079 | 0 | 0 | 0 | 15% | 99% | 0 | 0 | 0 | 0 | 100% | 99% | 100% |
| 1614 | 3-ketoacyl-CoA thiolase, peroxisomal OS=Homo sapiens GN=ACAA1 PE=1 SV=2 | THIK | 44 | 0.011 | 95% | 95% | 95% | 100% | 100% | 100% | 0 | 0 | 0 | 0 | 0 | 95% |
| 1615 | Acetyl-CoA acetyltransferase, mitochondrial OS=Homo sapiens GN=ACAT1 PE=1 SV=1 | THIL | 45 | 0.31 | 0 | 63% | 100% | 0 | 57% | 0 | 0 | 16% | 95% | 98% | 100% | 0 |
| 1616 | A-kinase anchor protein 2 OS=Homo sapiens GN=AKAP2 PE=1 SV=3 | AKAP2 | 95 | 0.00027 | 95% | 95% | 100% | 95% | 100% | 100% | 0 | 0 | 0 | 0 | 0 | 0 |
| 1617 | GH3 domain-containing protein OS=Homo sapiens GN=GHDC PE=1 SV=2 | GHDC | 58 | 0.0056 | 100% | 100% | 100% | 0 | 65% | 0 | 0 | 0 | 0 | 75% | 62% | 56% |
| 1618 | MAP7 domain-containing protein 1 OS=Homo sapiens GN=MAP7D1 PE=1 SV=1 | MA7D1 | 93 | 0.51 | 0 | 0 | 0 | 28% | 100% | 0 | 99% | 100% | 0 | 0 | 99% | 44% |
| 1619 | Follistatin-related protein 1 OS=Homo sapiens GN=FSTL1 PE=1 SV=1 | FSTL1 | 35 | 0.015 | 0 | 0 | 95% | 0 | 95% | 99% | 0 | 0 | 0 | 100% | 100% | 100% |
| 1620 | Suppressor of SWI4 1 homolog OS=Homo sapiens GN=PPAN PE=1 SV=1 | SSF1 | 53 | 0.0018 | 0 | 0 | 0 | 95% | 95% | 95% | 95% | 95% | 95% | 100% | 99% | 95% |
| 1621 | THO complex subunit 1 OS=Homo sapiens GN=THOC1 PE=1 SV=1 | THOC1 | 76 | 0.57 | 0 | 0 | 100% | 0 | 0 | 11% | 0 | 13% | 14% | 90% | 100% | 0 |
| 1622 | Small nuclear ribonucleoprotein Sm D2 OS=Homo sapiens GN=SNRPD2 PE=1 SV=1 | SMD2 | 14 | 0.0025 | 0 | 53% | 100% | 0 | 0 | 40% | 95% | 95% | 95% | 100% | 100% | 95% |
| 1623 | ADP-ribosylation factor-like protein 1 OS=Homo sapiens GN=ARL1 PE=1 SV=1 | ARL1 | 20 | 0.17 | 95% | 95% | 95% | 29% | 95% | 100% | 100% | 95% | 100% | 95% | 0 | 0 |
| 1624 | Dynamin-like 120 kDa protein, mitochondrial OS=Homo sapiens GN=OPA1 PE=1 SV=3 | OPA1 | 112 | 0.11 | 0 | 95% | 21% | 20% | 0 | 100% | 0 | 0 | 0 | 7% | 100% | 100% |
| 1625 | Vesicle transport protein SFT2C OS=Homo sapiens GN=SFT2D3 PE=2 SV=1 | SFT2C | 22 | 0.14 | 95% | 0 | 0 | 100% | 100% | 100% | 0 | 100% | 100% | 95% | 0 | 95% |
| 1626 | Mitochondrial import receptor subunit TOM22 homolog OS=Homo sapiens GN=TOMM22 PE=1 SV=3 | TOM22 | 16 | 0.0014 | 95% | 0 | 95% | 95% | 100% | 95% | 0 | 0 | 0 | 100% | 100% | 95% |
| 1627 | Protein S100-A4 OS=Homo sapiens GN=S100A4 PE=1 SV=1 | S10A4 | 12 | 0.16 | 13% | 100% | 100% | 0 | 0 | 0 | 100% | 100% | 100% | 0 | 0 | 0 |
| 1628 | UPF0554 protein C2orf43 OS=Homo sapiens GN=C2orf43 PE=1 SV=1 | CB043 | 37 | 0.086 | 47% | 95% | 98% | 95% | 95% | 95% | 100% | 80% | 77% | 23% | 0 | 95% |
| 1629 | Protein YIF1A OS=Homo sapiens GN=YIF1A PE=1 SV=2 | YIF1A | 32 | 0.13 | 100% | 95% | 100% | 0 | 100% | 0 | 0 | 40% | 0 | 0 | 95% | 0 |
| 1630 | Cytochrome c oxidase subunit 6C OS=Homo sapiens GN=COX6C PE=1 SV=2 | COX6C | 9 | 0.034 | 0 | 35% | 66% | 95% | 65% | 64% | 34% | 0 | 0 | 100% | 100% | 100% |
| 1631 | Lysocardiolipin acyltransferase 1 OS=Homo sapiens GN=LCLAT1 PE=1 SV=1 | LCLT1 | 49 | 0.7 | 0 | 0 | 100% | 0 | 100% | 100% | 100% | 95% | 65% | 57% | 95% | 0 |
| 1632 | Protein FAM134A OS=Homo sapiens GN=FAM134A PE=1 SV=3 | F134A | 58 | 0.42 | 0 | 95% | 0 | 0 | 7% | 92% | 0 | 100% | 100% | 15% | 100% | 0 |
| 1633 | Pre-mRNA-splicing factor 38A OS=Homo sapiens GN=PRPF38A PE=1 SV=1 | PR38A | 37 | 0.59 | 0 | 95% | 95% | 0 | 65% | 95% | 0 | 99% | 100% | 98% | 100% | 65% |
| 1634 | Serrate RNA effector molecule homolog OS=Homo sapiens GN=SRRT PE=1 SV=1 | SRRT | 101 | 0.75 | 0 | 95% | 0 | 0 | 95% | 0 | 7% | 95% | 95% | 0 | 100% | 100% |
| 1635 | Tubulin gamma-1 chain OS=Homo sapiens GN=TUBG1 PE=1 SV=2 | TBG1 (+1) | 51 | 0.4 | 93% | 44% | 95% | 26% | 40% | 100% | 0 | 0 | 0 | 98% | 100% | 11% |
| 1636 | Autophagy-related protein 9A OS=Homo sapiens GN=ATG9A PE=1 SV=3 | ATG9A | 94 | 0.64 | 0 | 0 | 95% | 0 | 95% | 95% | 80% | 100% | 100% | 95% | 55% | 95% |
| 1637 | von Willebrand factor A domain-containing protein 8 OS=Homo sapiens GN=VWA8 PE=1 SV=2 | VWA8 | 215 | 0.12 | 95% | 99% | 95% | 0 | 100% | 100% | 26% | 16% | 0 | 0 | 0 | 0 |
| 1638 | ATP-dependent RNA helicase DHX36 OS=Homo sapiens GN=DHX36 PE=1 SV=2 | DHX36 | 115 | 0.33 | 0 | 0 | 95% | 95% | 95% | 95% | 95% | 0 | 95% | 95% | 95% | 100% |
| 1639 | Fanconi anemia group M protein OS=Homo sapiens GN=FANCM PE=1 SV=2 | FANCM | 232 | 0.26 | 0 | 99% | 100% | 0 | 0 | 18% | 100% | 6% | 0 | 10% | 0 | 0 |
| 1640 | Nucleolar complex protein 3 homolog OS=Homo sapiens GN=NOC3L PE=1 SV=1 | NOC3L | 93 | 0.059 | 0 | 95% | 95% | 0 | 0 | 0 | 100% | 99% | 0 | 100% | 95% | 100% |
| 1641 | Long-chain fatty acid transport protein 3 OS=Homo sapiens GN=SLC27A3 PE=2 SV=3 | S27A3 | 79 | 0.00057 | 100% | 100% | 100% | 0 | 0 | 0 | 95% | 0 | 95% | 0 | 95% | 0 |
| 1642 | Synaptogyrin-2 OS=Homo sapiens GN=SYNGR2 PE=1 SV=1 | SNG2 | 25 | 0.11 | 0 | 0 | 0 | 95% | 100% | 0 | 0 | 0 | 0 | 100% | 95% | 100% |
| 1643 | Exocyst complex component 6B OS=Homo sapiens GN=EXOC6B PE=1 SV=3 | EXC6B | 94 | 0.026 | 0 | 95% | 95% | 95% | 34% | 98% | 0 | 0 | 0 | 100% | 95% | 100% |
| 1644 | N-acylneuraminate cytidylyltransferase OS=Homo sapiens GN=CMAS PE=1 SV=2 | NEUA | 48 | 0.0089 | 0 | 77% | 0 | 0 | 0 | 0 | 0 | 0 | 0 | 95% | 100% | 100% |
| 1645 | DNA-(apurinic or apyrimidinic site) lyase OS=Homo sapiens GN=APEX1 PE=1 SV=2 | APEX1 | 36 | 0.19 | 0 | 0 | 100% | 0 | 0 | 0 | 95% | 100% | 40% | 100% | 100% | 18% |
| 1646 | Protein odr-4 homolog OS=Homo sapiens GN=ODR4 PE=2 SV=1 | ODR4 | 51 | 0.0097 | 0 | 0 | 0 | 0 | 100% | 95% | 100% | 100% | 95% | 8% | 0 | 62% |
| 1647 | Mitochondrial antiviral-signaling protein OS=Homo sapiens GN=MAVS PE=1 SV=2 | MAVS | 57 | 0.00085 | 100% | 100% | 100% | 100% | 100% | 95% | 0 | 0 | 0 | 0 | 0 | 0 |
| 1648 | ATP synthase subunit f, mitochondrial OS=Homo sapiens GN=ATP5J2 PE=1 SV=3 | ATPK | 11 | 0.99 | 95% | 100% | 0 | 0 | 95% | 100% | 95% | 95% | 95% | 95% | 95% | 95% |
| 1649 | Nucleolar protein 16 OS=Homo sapiens GN=NOP16 PE=1 SV=2 | NOP16 | 21 | 0.68 | 0 | 100% | 0 | 0 | 100% | 95% | 95% | 0 | 100% | 95% | 98% | 0 |
| 1650 | Calcium uniporter protein, mitochondrial OS=Homo sapiens GN=MCU PE=1 SV=1 | MCU | 40 | 0.002 | 0 | 0 | 0 | 100% | 100% | 95% | 0 | 0 | 0 | 100% | 95% | 95% |
| 1651 | Acyl-coenzyme A thioesterase 9, mitochondrial OS=Homo sapiens GN=ACOT9 PE=1 SV=2 | ACOT9 | 50 | 0.12 | 0 | 0 | 0 | 0 | 96% | 18% | 0 | 95% | 5% | 100% | 100% | 95% |
| 1652 | Triple functional domain protein OS=Homo sapiens GN=TRIO PE=1 SV=2 | TRIO | 347 | 0.031 | 85% | 100% | 95% | 0 | 7% | 0 | 0 | 11% | 79% | 95% | 95% | 0 |
| 1653 | TATA element modulatory factor OS=Homo sapiens GN=TMF1 PE=1 SV=2 | TMF1 | 123 | 0.0032 | 0 | 0 | 0 | 100% | 95% | 100% | 0 | 0 | 0 | 98% | 97% | 0 |
| 1654 | UPF0556 protein C19orf10 OS=Homo sapiens GN=C19orf10 PE=1 SV=1 | CS010 | 19 | 0.031 | 0 | 99% | 20% | 100% | 95% | 100% | 0 | 0 | 0 | 95% | 95% | 0 |
| 1655 | Periodic tryptophan protein 1 homolog OS=Homo sapiens GN=PWP1 PE=1 SV=1 | PWP1 | 56 | 0.32 | 0 | 95% | 86% | 0 | 7% | 30% | 100% | 8% | 95% | 95% | 95% | 9% |
| 1656 | Signal peptide, CUB and EGF-like domain-containing protein 3 OS=Homo sapiens GN=SCUBE3 PE=1 SV=1 | SCUB3 | 109 | 0.0017 | 0 | 95% | 95% | 0 | 0 | 0 | 100% | 95% | 95% | 0 | 0 | 0 |
| 1657 | Structural maintenance of chromosomes protein 1A OS=Homo sapiens GN=SMC1A PE=1 SV=2 | SMC1A | 143 | 0.44 | 79% | 0 | 63% | 0 | 59% | 94% | 0 | 0 | 100% | 0 | 0 | 0 |
| 1658 | Protein PRRC2C OS=Homo sapiens GN=PRRC2C PE=1 SV=4 | PRC2C | 317 | 0.59 | 0 | 21% | 100% | 0 | 7% | 0 | 0 | 23% | 68% | 98% | 90% | 0 |
| 1659 | Oligosaccharyltransferase complex subunit OSTC OS=Homo sapiens GN=OSTC PE=1 SV=1 | OSTC | 17 | 0.29 | 0 | 0 | 95% | 95% | 0 | 95% | 0 | 100% | 100% | 0 | 0 | 8% |
| 1660 | Proteasome subunit beta type-2 OS=Homo sapiens GN=PSMB2 PE=1 SV=1 | PSB2 | 23 | 0.1 | 95% | 95% | 84% | 0 | 83% | 77% | 54% | 58% | 0 | 97% | 44% | 0 |
| 1661 | GPI ethanolamine phosphate transferase 2 OS=Homo sapiens GN=PIGG PE=1 SV=1 | PIGG | 108 | 0.24 | 95% | 0 | 0 | 96% | 11% | 95% | 95% | 0 | 95% | 95% | 95% | 95% |
| 1662 | Transmembrane protein 179B OS=Homo sapiens GN=TMEM179B PE=1 SV=1 | T179B | 24 | 0.074 | 95% | 95% | 95% | 95% | 95% | 95% | 95% | 95% | 95% | 95% | 95% | 95% |
| 1663 | Glutamate decarboxylase-like protein 1 OS=Homo sapiens GN=GADL1 PE=1 SV=4 | GADL1 | 59 | 0.44 | 42% | 66% | 67% | 38% | 91% | 30% | 58% | 0 | 0 | 8% | 8% | 23% |
| 1664 | Golgi reassembly-stacking protein 2 OS=Homo sapiens GN=GORASP2 PE=1 SV=3 | GORS2 | 47 | 0.0027 | 0 | 0 | 0 | 0 | 0 | 0 | 100% | 100% | 100% | 95% | 0 | 0 |
| 1665 | Heat shock protein beta-6 OS=Homo sapiens GN=HSPB6 PE=1 SV=2 | HSPB6 | 17 | 0.0068 | 0 | 74% | 26% | 0 | 0 | 0 | 100% | 100% | 100% | 0 | 0 | 0 |
| 1666 | RNA-binding protein with multiple splicing OS=Homo sapiens GN=RBPMS PE=1 SV=1 | RBPMS | 22 | 0.00087 | 0 | 0 | 0 | 0 | 0 | 0 | 100% | 100% | 100% | 0 | 0 | 0 |
| 1667 | Protein DEK OS=Homo sapiens GN=DEK PE=1 SV=1 | DEK | 43 | 0.00014 | 0 | 11% | 0 | 0 | 95% | 0 | 0 | 0 | 0 | 100% | 100% | 100% |
| 1668 | Laminin subunit beta-2 OS=Homo sapiens GN=LAMB2 PE=1 SV=2 | LAMB2 | 196 | 0.03 | 0 | 95% | 100% | 0 | 0 | 0 | 100% | 100% | 95% | 0 | 0 | 0 |
| 1669 | Pumilio domain-containing protein KIAA0020 OS=Homo sapiens GN=KIAA0020 PE=1 SV=3 | K0020 | 74 | 0.0077 | 0 | 0 | 0 | 0 | 0 | 0 | 95% | 0 | 100% | 100% | 100% | 95% |
| 1670 | KN motif and ankyrin repeat domain-containing protein 2 OS=Homo sapiens GN=KANK2 PE=1 SV=1 | KANK2 | 91 | 0.22 | 12% | 99% | 100% | 0 | 0 | 0 | 10% | 19% | 10% | 0 | 0 | 99% |
| 1671 | Paraspeckle component 1 OS=Homo sapiens GN=PSPC1 PE=1 SV=1 | PSPC1 | 59 | 0.047 | 0 | 0 | 0 | 98% | 18% | 98% | 99% | 100% | 97% | 72% | 98% | 95% |
| 1672 | ER membrane protein complex subunit 10 OS=Homo sapiens GN=EMC10 PE=1 SV=1 | EMC10 | 27 | 0.01 | 95% | 0 | 100% | 95% | 100% | 100% | 0 | 0 | 0 | 95% | 95% | 95% |
| 1673 | Golgi pH regulator A OS=Homo sapiens GN=GPR89A PE=1 SV=2 | GPHRA (+2) | 53 | 0.31 | 95% | 95% | 49% | 0 | 95% | 0 | 95% | 95% | 0 | 95% | 95% | 100% |
| 1674 | Protein MANBAL OS=Homo sapiens GN=MANBAL PE=1 SV=1 | MANBL | 9 | 0.033 | 95% | 95% | 95% | 0 | 0 | 0 | 0 | 95% | 95% | 100% | 100% | 90% |
| 1675 | Interferon-inducible double-stranded RNA-dependent protein kinase activator A OS=Homo sapiens GN=PRKRA PE=1 SV=1 | PRKRA | 34 | 0.37 | 95% | 100% | 95% | 0 | 95% | 95% | 0 | 95% | 0 | 95% | 100% | 0 |
| 1676 | 26S protease regulatory subunit 6A OS=Homo sapiens GN=PSMC3 PE=1 SV=3 | PRS6A | 49 | 0.012 | 0 | 0 | 12% | 0 | 0 | 0 | 100% | 95% | 100% | 0 | 0 | 95% |
| 1677 | Beta-hexosaminidase subunit alpha OS=Homo sapiens GN=HEXA PE=1 SV=2 | HEXA | 61 | 0.067 | 99% | 0 | 50% | 0 | 0 | 0 | 99% | 100% | 0 | 100% | 100% | 100% |
| 1678 | Transcription intermediary factor 1-beta OS=Homo sapiens GN=TRIM28 PE=1 SV=5 | TIF1B | 89 | 0.0016 | 0 | 95% | 0 | 8% | 0 | 0 | 100% | 100% | 100% | 0 | 0 | 0 |
| 1679 | 39S ribosomal protein L15, mitochondrial OS=Homo sapiens GN=MRPL15 PE=1 SV=1 | RM15 | 33 | 0.014 | 0 | 0 | 18% | 0 | 0 | 100% | 0 | 0 | 0 | 100% | 100% | 100% |
| 1680 | Phosphatidylserine synthase 2 OS=Homo sapiens GN=PTDSS2 PE=1 SV=1 | PTSS2 | 56 | 0.88 | 9% | 95% | 95% | 0 | 100% | 95% | 0 | 63% | 95% | 95% | 14% | 95% |
| 1681 | Nuclear receptor coactivator 5 OS=Homo sapiens GN=NCOA5 PE=1 SV=2 | NCOA5 | 66 | 0.6 | 0 | 98% | 0 | 11% | 0 | 95% | 6% | 71% | 0 | 0 | 100% | 0 |
| 1682 | Aldehyde dehydrogenase family 16 member A1 OS=Homo sapiens GN=ALDH16A1 PE=1 SV=2 | A16A1 | 85 | 0.12 | 95% | 95% | 95% | 95% | 95% | 79% | 100% | 13% | 0 | 0 | 0 | 0 |
| 1683 | Mitochondrial import inner membrane translocase subunit TIM50 OS=Homo sapiens GN=TIMM50 PE=1 SV=2 | TIM50 | 40 | 0.12 | 0 | 0 | 0 | 79% | 100% | 99% | 0 | 5% | 0 | 95% | 95% | 0 |
| 1684 | Myelin expression factor 2 OS=Homo sapiens GN=MYEF2 PE=1 SV=3 | MYEF2 | 64 | 0.0038 | 0 | 0 | 0 | 95% | 95% | 95% | 100% | 95% | 95% | 0 | 0 | 100% |
| 1685 | Hemoglobin subunit delta OS=Homo sapiens GN=HBD PE=1 SV=2 | HBD | 16 | 0.12 | 95% | 95% | 100% | 0 | 35% | 9% | 34% | 95% | 95% | 0 | 95% | 75% |
| 1686 | Transmembrane protein 120A OS=Homo sapiens GN=TMEM120A PE=2 SV=1 | T120A | 41 | 0.096 | 95% | 79% | 0 | 66% | 95% | 95% | 0 | 98% | 0 | 95% | 100% | 95% |
| 1687 | Protein FAM134C OS=Homo sapiens GN=FAM134C PE=1 SV=1 | F134C | 51 | 0.083 | 0 | 0 | 0 | 95% | 14% | 95% | 95% | 95% | 95% | 0 | 0 | 99% |
| 1688 | Polyadenylate-binding protein 5 OS=Homo sapiens GN=PABPC5 PE=2 SV=1 | PABP5 | 43 | 0.82 | 94% | 0 | 0 | 22% | 51% | 98% | 0 | 87% | 49% | 41% | 43% | 94% |
| 1689 | Lipid phosphate phosphohydrolase 1 OS=Homo sapiens GN=PPAP2A PE=1 SV=1 | LPP1 | 32 | 0.00014 | 95% | 95% | 0 | 95% | 95% | 95% | 95% | 95% | 95% | 0 | 0 | 0 |
| 1690 | Signal peptidase complex subunit 1 OS=Homo sapiens GN=SPCS1 PE=1 SV=4 | SPCS1 | 12 | 0.74 | 0 | 95% | 95% | 0 | 95% | 95% | 0 | 0 | 95% | 16% | 95% | 95% |
| 1691 | Stomatin-like protein 1 OS=Homo sapiens GN=STOML1 PE=1 SV=1 | STML1 | 43 | 0.62 | 95% | 95% | 95% | 0 | 95% | 95% | 95% | 95% | 95% | 95% | 95% | 95% |
| 1692 | Cluster of Transcription initiation factor TFIID subunit 1-like OS=Homo sapiens GN=TAF1L PE=1 SV=1 (TAF1L_HUMAN) | TAF1L [2] | 207 | 0.44 | 23% | 33% | 42% | 0 | 99% | 43% | 42% | 10% | 33% | 0 | 0 | 22% |
| 1693 | Squalene monooxygenase OS=Homo sapiens GN=SQLE PE=1 SV=3 | ERG1 | 64 | 0.15 | 0 | 0 | 10% | 0 | 0 | 0 | 0 | 100% | 100% | 0 | 0 | 0 |
| 1694 | Protein ITFG3 OS=Homo sapiens GN=ITFG3 PE=1 SV=1 | ITFG3 | 60 | < 0.00010 | 100% | 100% | 100% | 0 | 0 | 95% | 0 | 0 | 0 | 0 | 0 | 0 |
| 1695 | Dehydrogenase/reductase SDR family member 1 OS=Homo sapiens GN=DHRS1 PE=1 SV=1 | DHRS1 | 34 | 0.43 | 10% | 95% | 100% | 95% | 61% | 0 | 0 | 0 | 0 | 0 | 95% | 95% |
| 1696 | Transmembrane emp24 domain-containing protein 5 OS=Homo sapiens GN=TMED5 PE=1 SV=1 | TMED5 | 26 | 0.62 | 95% | 95% | 100% | 95% | 0 | 95% | 0 | 100% | 0 | 0 | 0 | 95% |
| 1697 | NADH dehydrogenase [ubiquinone] 1 alpha subcomplex subunit 5 OS=Homo sapiens GN=NDUFA5 PE=1 SV=3 | NDUA5 | 13 | 0.0028 | 0 | 0 | 0 | 0 | 45% | 0 | 0 | 0 | 0 | 100% | 100% | 100% |
| 1698 | Sulfatase-modifying factor 2 OS=Homo sapiens GN=SUMF2 PE=1 SV=2 | SUMF2 | 34 | 0.058 | 0 | 95% | 99% | 0 | 0 | 95% | 0 | 0 | 0 | 95% | 100% | 100% |
| 1699 | Collagen alpha-1(II) chain OS=Homo sapiens GN=COL2A1 PE=1 SV=3 | CO2A1 | 142 | 0.34 | 0 | 53% | 100% | 0 | 36% | 50% | 16% | 100% | 100% | 0 | 0 | 0 |
| 1700 | Alpha/beta hydrolase domain-containing protein 17B OS=Homo sapiens GN=ABHD17B PE=2 SV=1 | AB17B | 32 | 0.17 | 0 | 92% | 29% | 0 | 0 | 0 | 80% | 100% | 100% | 0 | 0 | 0 |
| 1701 | Brain protein I3 OS=Homo sapiens GN=BRI3 PE=2 SV=1 | BRI3 | 14 | 0.022 | 0 | 0 | 95% | 0 | 95% | 0 | 100% | 100% | 95% | 95% | 95% | 95% |
| 1702 | Phosphatidate cytidylyltransferase 2 OS=Homo sapiens GN=CDS2 PE=1 SV=1 | CDS2 | 51 | 0.12 | 0 | 95% | 95% | 95% | 100% | 0 | 100% | 95% | 95% | 0 | 0 | 0 |
| 1703 | Alpha-(1,6)-fucosyltransferase OS=Homo sapiens GN=FUT8 PE=1 SV=2 | FUT8 | 67 | 0.013 | 95% | 95% | 95% | 95% | 0 | 0 | 7% | 0 | 0 | 100% | 100% | 95% |
| 1704 | Gamma-tubulin complex component 2 OS=Homo sapiens GN=TUBGCP2 PE=1 SV=2 | GCP2 | 103 | 0.19 | 0 | 0 | 0 | 19% | 100% | 100% | 0 | 7% | 0 | 98% | 0 | 95% |
| 1705 | NADH dehydrogenase [ubiquinone] 1 alpha subcomplex subunit 9, mitochondrial OS=Homo sapiens GN=NDUFA9 PE=1 SV=2 | NDUA9 | 43 | 0.39 | 99% | 100% | 0 | 95% | 0 | 0 | 0 | 0 | 0 | 95% | 100% | 95% |
| 1706 | NADH dehydrogenase [ubiquinone] 1 alpha subcomplex subunit 13 OS=Homo sapiens GN=NDUFA13 PE=1 SV=3 | NDUAD | 17 | 0.075 | 95% | 79% | 0 | 0 | 95% | 95% | 0 | 0 | 0 | 100% | 100% | 100% |
| 1707 | Zinc transporter 6 OS=Homo sapiens GN=SLC30A6 PE=1 SV=2 | ZNT6 | 51 | 0.22 | 0 | 0 | 0 | 0 | 95% | 95% | 95% | 100% | 0 | 100% | 95% | 95% |
| 1708 | RNA-binding protein 4 OS=Homo sapiens GN=RBM4 PE=1 SV=1 | RBM4 | 40 | 0.0037 | 0 | 0 | 0 | 0 | 95% | 0 | 0 | 0 | 0 | 100% | 100% | 100% |
| 1709 | Single-stranded DNA-binding protein, mitochondrial OS=Homo sapiens GN=SSBP1 PE=1 SV=1 | SSBP | 17 | 0.017 | 95% | 99% | 100% | 0 | 0 | 0 | 95% | 95% | 100% | 0 | 74% | 0 |
| 1710 | Dynein heavy chain 1, axonemal OS=Homo sapiens GN=DNAH1 PE=2 SV=4 | DYH1 | 494 | 0.44 | 0 | 0 | 0 | 0 | 100% | 0 | 0 | 0 | 0 | 0 | 0 | 96% |
| 1711 | Calcium homeostasis endoplasmic reticulum protein OS=Homo sapiens GN=CHERP PE=1 SV=3 | CHERP | 104 | 0.0097 | 0 | 0 | 0 | 0 | 0 | 0 | 95% | 0 | 0 | 100% | 100% | 100% |
| 1712 | Transportin-1 OS=Homo sapiens GN=TNPO1 PE=1 SV=2 | TNPO1 | 102 | 0.21 | 0 | 0 | 0 | 0 | 0 | 0 | 95% | 100% | 0 | 9% | 0 | 100% |
| 1713 | Ribosomal RNA processing protein 1 homolog A OS=Homo sapiens GN=RRP1 PE=1 SV=1 | RRP1 | 53 | 0.0025 | 0 | 0 | 100% | 76% | 0 | 0 | 98% | 100% | 98% | 0 | 0 | 0 |
| 1714 | GDH/6PGL endoplasmic bifunctional protein OS=Homo sapiens GN=H6PD PE=1 SV=2 | G6PE | 89 | 0.052 | 0 | 0 | 0 | 0 | 0 | 0 | 5% | 0 | 0 | 100% | 5% | 100% |
| 1715 | Protein PRRC2A OS=Homo sapiens GN=PRRC2A PE=1 SV=3 | PRC2A | 229 | 0.0032 | 0 | 0 | 0 | 100% | 90% | 95% | 0 | 0 | 0 | 100% | 99% | 0 |
| 1716 | PHD finger-like domain-containing protein 5A OS=Homo sapiens GN=PHF5A PE=1 SV=1 | PHF5A | 12 | 0.44 | 0 | 100% | 0 | 0 | 86% | 21% | 0 | 0 | 0 | 12% | 13% | 81% |
| 1717 | Ubiquitin conjugation factor E4 A OS=Homo sapiens GN=UBE4A PE=1 SV=2 | UBE4A | 123 | 0.055 | 0 | 0 | 0 | 95% | 0 | 99% | 0 | 0 | 0 | 0 | 99% | 0 |
| 1718 | Legumain OS=Homo sapiens GN=LGMN PE=1 SV=1 | LGMN | 49 | 0.62 | 0 | 95% | 0 | 95% | 0 | 0 | 99% | 95% | 95% | 0 | 0 | 95% |
| 1719 | Dynein heavy chain 2, axonemal OS=Homo sapiens GN=DNAH2 PE=2 SV=3 | DYH2 | 508 | 0.44 | 0 | 13% | 0 | 0 | 0 | 84% | 0 | 0 | 0 | 0 | 7% | 99% |
| 1720 | VIP36-like protein OS=Homo sapiens GN=LMAN2L PE=1 SV=1 | LMA2L | 40 | 0.44 | 0 | 95% | 19% | 98% | 9% | 98% | 0 | 0 | 17% | 14% | 0 | 0 |
| 1721 | Ethanolaminephosphotransferase 1 OS=Homo sapiens GN=EPT1 PE=1 SV=3 | EPT1 | 45 | 0.045 | 0 | 8% | 95% | 95% | 95% | 95% | 95% | 83% | 95% | 0 | 95% | 0 |
| 1722 | Peptidyl-prolyl cis-trans isomerase FKBP2 OS=Homo sapiens GN=FKBP2 PE=1 SV=2 | FKBP2 | 16 | 0.26 | 0 | 95% | 95% | 95% | 95% | 95% | 95% | 95% | 95% | 95% | 0 | 95% |
| 1723 | Integral membrane protein GPR180 OS=Homo sapiens GN=GPR180 PE=2 SV=1 | GP180 | 49 | 0.44 | 95% | 0 | 95% | 95% | 95% | 95% | 95% | 95% | 0 | 95% | 95% | 71% |
| 1724 | Protein-S-isoprenylcysteine O-methyltransferase OS=Homo sapiens GN=ICMT PE=1 SV=1 | ICMT | 32 | 0.44 | 18% | 95% | 15% | 0 | 0 | 23% | 42% | 26% | 20% | 29% | 28% | 0 |
| 1725 | Protein S100-A13 OS=Homo sapiens GN=S100A13 PE=1 SV=1 | S10AD | 11 | 0.44 | 0 | 95% | 10% | 15% | 15% | 0 | 14% | 13% | 0 | 0 | 13% | 14% |
| 1726 | Structural maintenance of chromosomes protein 3 OS=Homo sapiens GN=SMC3 PE=1 SV=2 | SMC3 | 142 | 0.6 | 0 | 0 | 0 | 88% | 0 | 0 | 67% | 95% | 79% | 0 | 95% | 0 |
| 1727 | Transmembrane protein 41B OS=Homo sapiens GN=TMEM41B PE=1 SV=1 | TM41B | 33 | 0.24 | 0 | 95% | 0 | 0 | 95% | 95% | 95% | 0 | 0 | 95% | 95% | 95% |
| 1728 | Trafficking protein particle complex subunit 3 OS=Homo sapiens GN=TRAPPC3 PE=1 SV=1 | TPPC3 | 20 | 0.44 | 20% | 63% | 66% | 6% | 65% | 64% | 63% | 63% | 0 | 60% | 95% | 0 |
| 1729 | Vacuolar ATPase assembly integral membrane protein VMA21 OS=Homo sapiens GN=VMA21 PE=1 SV=1 | VMA21 | 11 | 0.98 | 0 | 95% | 95% | 0 | 95% | 95% | 95% | 0 | 95% | 95% | 95% | 0 |
| 1730 | Aladin OS=Homo sapiens GN=AAAS PE=1 SV=1 | AAAS | 60 | 0.0057 | 0 | 0 | 0 | 100% | 100% | 100% | 0 | 0 | 0 | 95% | 0 | 95% |
| 1731 | Liver carboxylesterase 1 OS=Homo sapiens GN=CES1 PE=1 SV=2 | EST1 | 63 | 0.0029 | 0 | 0 | 0 | 0 | 0 | 0 | 0 | 0 | 0 | 95% | 100% | 100% |
| 1732 | Bifunctional heparan sulfate N-deacetylase/N-sulfotransferase 1 OS=Homo sapiens GN=NDST1 PE=1 SV=1 | NDST1 | 101 | 0.46 | 0 | 0 | 16% | 0 | 0 | 100% | 0 | 0 | 0 | 100% | 9% | 100% |
| 1733 | NADH dehydrogenase [ubiquinone] flavoprotein 1, mitochondrial OS=Homo sapiens GN=NDUFV1 PE=1 SV=4 | NDUV1 | 51 | 0.063 | 0 | 0 | 0 | 95% | 95% | 95% | 0 | 0 | 0 | 95% | 90% | 100% |
| 1734 | Protein eva-1 homolog B OS=Homo sapiens GN=EVA1B PE=1 SV=1 | EVA1B | 18 | 0.48 | 0 | 100% | 95% | 0 | 0 | 95% | 95% | 0 | 0 | 0 | 0 | 0 |
| 1735 | Glutathione S-transferase kappa 1 OS=Homo sapiens GN=GSTK1 PE=1 SV=3 | GSTK1 | 25 | 0.38 | 0 | 100% | 100% | 0 | 0 | 0 | 0 | 0 | 0 | 100% | 0 | 0 |
| 1736 | Calcium signal-modulating cyclophilin ligand OS=Homo sapiens GN=CAMLG PE=1 SV=1 | CAMLG | 33 | 0.028 | 100% | 95% | 100% | 0 | 0 | 0 | 100% | 0 | 95% | 0 | 0 | 0 |
| 1737 | Cytoplasmic dynein 1 light intermediate chain 2 OS=Homo sapiens GN=DYNC1LI2 PE=1 SV=1 | DC1L2 | 54 | 0.045 | 0 | 0 | 0 | 0 | 60% | 95% | 100% | 95% | 95% | 95% | 65% | 0 |
| 1738 | Protein kish-A OS=Homo sapiens GN=TMEM167A PE=1 SV=1 | KISHA | 8 | 0.0023 | 0 | 95% | 0 | 0 | 0 | 0 | 100% | 100% | 95% | 95% | 95% | 95% |
| 1739 | Ribosomal biogenesis protein LAS1L OS=Homo sapiens GN=LAS1L PE=1 SV=2 | LAS1L | 83 | 0.35 | 10% | 0 | 0 | 0 | 97% | 0 | 100% | 100% | 0 | 0 | 95% | 0 |
| 1740 | Transmembrane protein 106B OS=Homo sapiens GN=TMEM106B PE=1 SV=2 | T106B | 31 | 0.0055 | 0 | 95% | 95% | 0 | 0 | 0 | 100% | 100% | 100% | 0 | 0 | 0 |
| 1741 | Thioredoxin domain-containing protein 15 OS=Homo sapiens GN=TXNDC15 PE=1 SV=1 | TXD15 | 40 | 0.047 | 0 | 0 | 95% | 0 | 100% | 95% | 0 | 0 | 0 | 100% | 95% | 95% |
| 1742 | Vitamin K-dependent gamma-carboxylase OS=Homo sapiens GN=GGCX PE=1 SV=2 | VKGC | 88 | 0.25 | 0 | 95% | 0 | 95% | 100% | 0 | 0 | 26% | 41% | 0 | 100% | 67% |
| 1743 | Putative helicase MOV-10 OS=Homo sapiens GN=MOV10 PE=1 SV=2 | MOV10 | 114 | < 0.00010 | 0 | 0 | 0 | 0 | 0 | 18% | 33% | 58% | 0 | 100% | 95% | 100% |
| 1744 | Transmembrane protein 63A OS=Homo sapiens GN=TMEM63A PE=2 SV=3 | TM63A | 92 | 0.063 | 0 | 95% | 0 | 0 | 0 | 100% | 100% | 100% | 100% | 0 | 0 | 0 |
| 1745 | Intron-binding protein aquarius OS=Homo sapiens GN=AQR PE=1 SV=4 | AQR | 171 | 0.082 | 0 | 0 | 0 | 0 | 95% | 0 | 95% | 95% | 100% | 0 | 0 | 95% |
| 1746 | Cysteine-rich with EGF-like domain protein 1 OS=Homo sapiens GN=CRELD1 PE=1 SV=3 | CREL1 | 45 | 0.0088 | 0 | 0 | 0 | 0 | 95% | 95% | 0 | 0 | 0 | 100% | 100% | 95% |
| 1747 | Small nuclear ribonucleoprotein F OS=Homo sapiens GN=SNRPF PE=1 SV=1 | RUXF | 10 | 0.0037 | 0 | 0 | 0 | 0 | 0 | 0 | 63% | 100% | 95% | 95% | 95% | 95% |
| 1748 | UAP56-interacting factor OS=Homo sapiens GN=FYTTD1 PE=1 SV=3 | UIF | 36 | 0.31 | 0 | 0 | 95% | 0 | 0 | 0 | 0 | 95% | 97% | 95% | 100% | 77% |
| 1749 | Transcription activator BRG1 OS=Homo sapiens GN=SMARCA4 PE=1 SV=2 | SMCA4 | 185 | 0.23 | 0 | 26% | 0 | 0 | 0 | 0 | 100% | 0 | 6% | 95% | 95% | 0 |
| 1750 | Heparan-alpha-glucosaminide N-acetyltransferase OS=Homo sapiens GN=HGSNAT PE=1 SV=2 | HGNAT | 73 | 0.21 | 95% | 0 | 0 | 100% | 0 | 95% | 95% | 95% | 95% | 0 | 95% | 0 |
| 1751 | UTP--glucose-1-phosphate uridylyltransferase OS=Homo sapiens GN=UGP2 PE=1 SV=5 | UGPA | 57 | 0.013 | 0 | 18% | 0 | 0 | 0 | 0 | 0 | 95% | 7% | 100% | 86% | 100% |
| 1752 | Inactive rhomboid protein 1 OS=Homo sapiens GN=RHBDF1 PE=1 SV=2 | RHDF1 | 97 | 0.59 | 0 | 100% | 15% | 0 | 15% | 0 | 0 | 0 | 0 | 100% | 13% | 96% |
| 1753 | Cathepsin L1 OS=Homo sapiens GN=CTSL PE=1 SV=2 | CATL1 | 38 | 0.44 | 0 | 10% | 65% | 0 | 0 | 0 | 0 | 0 | 0 | 12% | 100% | 84% |
| 1754 | NADH dehydrogenase [ubiquinone] 1 beta subcomplex subunit 4 OS=Homo sapiens GN=NDUFB4 PE=1 SV=3 | NDUB4 | 15 | 0.2 | 0 | 0 | 0 | 29% | 94% | 91% | 0 | 0 | 0 | 53% | 8% | 100% |
| 1755 | Stereocilin OS=Homo sapiens GN=STRC PE=2 SV=1 | STRC | 193 | 0.44 | 0 | 0 | 40% | 0 | 100% | 17% | 17% | 91% | 0 | 0 | 15% | 0 |
| 1756 | Protein RFT1 homolog OS=Homo sapiens GN=RFT1 PE=1 SV=1 | RFT1 | 60 | 0.44 | 0 | 0 | 23% | 0 | 54% | 0 | 100% | 0 | 23% | 25% | 0 | 0 |
| 1757 | Receptor expression-enhancing protein 3 OS=Homo sapiens GN=REEP3 PE=1 SV=1 | REEP3 | 29 | 0.12 | 0 | 83% | 0 | 0 | 0 | 80% | 100% | 95% | 8% | 5% | 100% | 100% |
| 1758 | Splicing factor 1 OS=Homo sapiens GN=SF1 PE=1 SV=4 | SF01 | 68 | 0.44 | 0 | 12% | 43% | 0 | 100% | 0 | 0 | 12% | 8% | 0 | 55% | 0 |
| 1759 | 6-phosphogluconate dehydrogenase, decarboxylating OS=Homo sapiens GN=PGD PE=1 SV=3 | 6PGD | 53 | 0.0039 | 9% | 100% | 0 | 0 | 0 | 0 | 0 | 0 | 0 | 95% | 95% | 100% |
| 1760 | Vesicle-associated membrane protein 4 OS=Homo sapiens GN=VAMP4 PE=1 SV=2 | VAMP4 | 16 | 0.39 | 92% | 19% | 95% | 0 | 95% | 12% | 95% | 100% | 0 | 0 | 0 | 0 |
| 1761 | NADH dehydrogenase [ubiquinone] 1 beta subcomplex subunit 10 OS=Homo sapiens GN=NDUFB10 PE=1 SV=3 | NDUBA | 21 | 0.36 | 93% | 95% | 95% | 27% | 71% | 95% | 0 | 0 | 0 | 0 | 100% | 0 |
| 1762 | Carboxypeptidase Q OS=Homo sapiens GN=CPQ PE=1 SV=1 | CBPQ | 52 | 0.35 | 100% | 0 | 95% | 0 | 0 | 0 | 0 | 73% | 99% | 95% | 0 | 0 |
| 1763 | Cleavage and polyadenylation specificity factor subunit 3 OS=Homo sapiens GN=CPSF3 PE=1 SV=1 | CPSF3 | 77 | 0.12 | 0 | 0 | 0 | 10% | 24% | 0 | 33% | 83% | 99% | 95% | 95% | 0 |
| 1764 | Transducin beta-like protein 3 OS=Homo sapiens GN=TBL3 PE=1 SV=2 | TBL3 | 89 | 0.42 | 0 | 0 | 0 | 95% | 0 | 99% | 0 | 0 | 95% | 98% | 84% | 0 |
| 1765 | 39S ribosomal protein L4, mitochondrial OS=Homo sapiens GN=MRPL4 PE=1 SV=1 | RM04 | 35 | 0.13 | 0 | 74% | 95% | 95% | 95% | 95% | 0 | 0 | 0 | 98% | 95% | 0 |
| 1766 | Choline/ethanolaminephosphotransferase 1 OS=Homo sapiens GN=CEPT1 PE=1 SV=1 | CEPT1 | 47 | 0.052 | 95% | 50% | 0 | 95% | 72% | 95% | 0 | 17% | 0 | 95% | 22% | 10% |
| 1767 | P39900-DECOY | P39900-DECOY (+1) | ? | 0.56 | 0 | 60% | 66% | 95% | 0 | 64% | 95% | 0 | 65% | 60% | 95% | 95% |
| 1768 | Vesicle transport protein SEC20 OS=Homo sapiens GN=BNIP1 PE=1 SV=3 | SEC20 | 26 | 0.31 | 0 | 95% | 95% | 95% | 0 | 0 | 95% | 95% | 95% | 95% | 95% | 95% |
| 1769 | Mitochondrial import receptor subunit TOM40 homolog OS=Homo sapiens GN=TOMM40 PE=1 SV=1 | TOM40 | 38 | 0.22 | 95% | 0 | 20% | 95% | 91% | 24% | 0 | 0 | 0 | 95% | 0 | 95% |
| 1770 | DNA dC->dU-editing enzyme APOBEC-3C OS=Homo sapiens GN=APOBEC3C PE=1 SV=2 | ABC3C | 23 | 0.33 | 0 | 0 | 95% | 0 | 0 | 0 | 95% | 0 | 0 | 95% | 95% | 17% |
| 1771 | Ubiquinone biosynthesis monooxygenase COQ6 OS=Homo sapiens GN=COQ6 PE=1 SV=2 | COQ6 | 51 | 0.44 | 14% | 0 | 0 | 32% | 42% | 0 | 0 | 0 | 0 | 21% | 95% | 0 |
| 1772 | Integrator complex subunit 11 OS=Homo sapiens GN=CPSF3L PE=1 SV=2 | INT11 | 68 | 0.8 | 0 | 0 | 29% | 18% | 89% | 0 | 0 | 0 | 95% | 31% | 95% | 0 |
| 1773 | LysM and putative peptidoglycan-binding domain-containing protein 3 OS=Homo sapiens GN=LYSMD3 PE=1 SV=2 | LYSM3 | 35 | 0.031 | 0 | 95% | 50% | 92% | 79% | 95% | 12% | 17% | 0 | 91% | 0 | 0 |
| 1774 | Ribosomal RNA small subunit methyltransferase NEP1 OS=Homo sapiens GN=EMG1 PE=1 SV=4 | NEP1 | 27 | 0.16 | 0 | 95% | 0 | 0 | 95% | 45% | 95% | 0 | 0 | 95% | 94% | 95% |
| 1775 | GTP-binding protein SAR1a OS=Homo sapiens GN=SAR1A PE=1 SV=1 | SAR1A (+1) | 22 | 0.1 | 91% | 95% | 95% | 0 | 0 | 0 | 95% | 57% | 27% | 80% | 0 | 95% |
| 1776 | Serpin I2 OS=Homo sapiens GN=SERPINI2 PE=1 SV=1 | SPI2 | 46 | 0.052 | 0 | 0 | 8% | 0 | 0 | 0 | 9% | 95% | 95% | 0 | 32% | 0 |
| 1777 | Zinc finger CCHC domain-containing protein 8 OS=Homo sapiens GN=ZCCHC8 PE=1 SV=2 | ZCHC8 | 79 | 0.074 | 0 | 95% | 95% | 0 | 0 | 0 | 95% | 95% | 15% | 95% | 95% | 95% |
| 1778 | Q5W0U4-DECOY | Q5W0U4-DECOY | ? | 0.68 | 0 | 83% | 0 | 88% | 0 | 45% | 0 | 0 | 95% | 79% | 85% | 86% |
| 1779 | Cluster of Protein FAM98A OS=Homo sapiens GN=FAM98A PE=1 SV=1 (FA98A_HUMAN) | FA98A [2] | 55 | 0.31 | 0 | 0 | 95% | 0 | 0 | 0 | 99% | 100% | 0 | 95% | 95% | 0 |
| 1780 | Cluster of RNA-binding motif, single-stranded-interacting protein 2 OS=Homo sapiens GN=RBMS2 PE=1 SV=1 (RBMS2_HUMAN) | RBMS2 [2] | 44 | 0.097 | 0 | 0 | 0 | 0 | 0 | 0 | 0 | 0 | 0 | 95% | 100% | 0 |
| 1781 | EMILIN-1 OS=Homo sapiens GN=EMILIN1 PE=1 SV=2 | EMIL1 | 107 | < 0.00010 | 0 | 0 | 0 | 0 | 0 | 0 | 0 | 0 | 0 | 100% | 100% | 100% |
| 1782 | Alpha-N-acetylgalactosaminidase OS=Homo sapiens GN=NAGA PE=1 SV=2 | NAGAB | 47 | 0.13 | 0 | 95% | 95% | 0 | 0 | 0 | 0 | 0 | 0 | 100% | 0 | 100% |
| 1783 | Ras-related GTP-binding protein A OS=Homo sapiens GN=RRAGA PE=1 SV=1 | RRAGA | 37 | 0.0054 | 0 | 95% | 0 | 0 | 0 | 0 | 100% | 100% | 100% | 0 | 0 | 0 |
| 1784 | Disrupted in renal carcinoma protein 2 OS=Homo sapiens GN=DIRC2 PE=1 SV=1 | DIRC2 | 52 | 0.32 | 0 | 0 | 0 | 0 | 0 | 0 | 100% | 95% | 0 | 0 | 100% | 93% |
| 1785 | Dolichol-phosphate mannosyltransferase subunit 3 OS=Homo sapiens GN=DPM3 PE=1 SV=2 | DPM3 | 10 | 0.51 | 0 | 100% | 100% | 0 | 0 | 95% | 0 | 95% | 95% | 0 | 95% | 0 |
| 1786 | Eukaryotic translation initiation factor 3 subunit M OS=Homo sapiens GN=EIF3M PE=1 SV=1 | EIF3M | 43 | 0.0037 | 0 | 0 | 0 | 0 | 0 | 0 | 0 | 0 | 100% | 100% | 100% | 95% |
| 1787 | Glycogen [starch] synthase, muscle OS=Homo sapiens GN=GYS1 PE=1 SV=2 | GYS1 | 84 | 0.4 | 0 | 100% | 100% | 44% | 0 | 100% | 0 | 0 | 0 | 0 | 0 | 0 |
| 1788 | MKI67 FHA domain-interacting nucleolar phosphoprotein OS=Homo sapiens GN=NIFK PE=1 SV=1 | MK67I | 34 | 0.019 | 0 | 0 | 0 | 0 | 0 | 95% | 95% | 95% | 95% | 95% | 95% | 100% |
| 1789 | RAD50-interacting protein 1 OS=Homo sapiens GN=RINT1 PE=1 SV=1 | RINT1 | 91 | 0.33 | 0 | 95% | 0 | 0 | 0 | 0 | 99% | 0 | 0 | 100% | 0 | 99% |
| 1790 | Splicing factor 3B subunit 4 OS=Homo sapiens GN=SF3B4 PE=1 SV=1 | SF3B4 | 44 | 0.0011 | 0 | 0 | 0 | 0 | 0 | 0 | 95% | 100% | 95% | 95% | 95% | 95% |
| 1791 | Vacuolar protein sorting-associated protein 41 homolog OS=Homo sapiens GN=VPS41 PE=1 SV=3 | VPS41 | 99 | 0.62 | 100% | 95% | 0 | 0 | 0 | 100% | 0 | 0 | 28% | 95% | 95% | 0 |
| 1792 | E3 ubiquitin-protein ligase TRIP12 OS=Homo sapiens GN=TRIP12 PE=1 SV=1 | TRIPC | 220 | 0.44 | 0 | 0 | 0 | 67% | 100% | 0 | 0 | 0 | 0 | 0 | 54% | 0 |
| 1793 | Exostosin-1 OS=Homo sapiens GN=EXT1 PE=1 SV=2 | EXT1 | 86 | 0.19 | 0 | 0 | 0 | 15% | 69% | 52% | 0 | 100% | 99% | 18% | 0 | 0 |
| 1794 | ERO1-like protein alpha OS=Homo sapiens GN=ERO1L PE=1 SV=2 | ERO1A | 54 | 0.25 | 0 | 100% | 95% | 0 | 0 | 32% | 0 | 0 | 0 | 100% | 0 | 0 |
| 1795 | CAD protein OS=Homo sapiens GN=CAD PE=1 SV=3 | PYR1 | 243 | 0.00031 | 0 | 0 | 0 | 0 | 0 | 0 | 100% | 100% | 100% | 0 | 0 | 0 |
| 1796 | AP-1 complex subunit gamma-1 OS=Homo sapiens GN=AP1G1 PE=1 SV=5 | AP1G1 | 91 | 0.16 | 0 | 0 | 0 | 0 | 100% | 100% | 0 | 0 | 0 | 100% | 95% | 0 |
| 1797 | Deleted in autism protein 1 OS=Homo sapiens GN=C3orf58 PE=1 SV=1 | DIA1 | 49 | 0.23 | 0 | 0 | 0 | 0 | 0 | 100% | 95% | 95% | 17% | 0 | 16% | 6% |
| 1798 | Vacuolar protein sorting-associated protein 13C OS=Homo sapiens GN=VPS13C PE=1 SV=1 | VP13C | 422 | 0.052 | 5% | 0 | 0 | 0 | 0 | 0 | 0 | 95% | 100% | 0 | 0 | 0 |
| 1799 | Far upstream element-binding protein 1 OS=Homo sapiens GN=FUBP1 PE=1 SV=3 | FUBP1 | 68 | 0.032 | 0 | 0 | 0 | 0 | 10% | 100% | 0 | 0 | 100% | 99% | 40% | 100% |
| 1800 | DnaJ homolog subfamily B member 2 OS=Homo sapiens GN=DNAJB2 PE=1 SV=3 | DNJB2 | 36 | 0.0016 | 14% | 100% | 95% | 95% | 100% | 95% | 0 | 0 | 0 | 0 | 0 | 0 |
| 1801 | Ufm1-specific protease 2 OS=Homo sapiens GN=UFSP2 PE=1 SV=3 | UFSP2 | 53 | 0.098 | 0 | 0 | 0 | 0 | 95% | 0 | 100% | 95% | 0 | 100% | 95% | 95% |
| 1802 | Protein unc-93 homolog B1 OS=Homo sapiens GN=UNC93B1 PE=1 SV=2 | UN93B | 67 | 0.4 | 95% | 95% | 0 | 0 | 0 | 0 | 0 | 25% | 100% | 95% | 95% | 15% |
| 1803 | Putative ATP-dependent RNA helicase DHX30 OS=Homo sapiens GN=DHX30 PE=1 SV=1 | DHX30 | 134 | 0.33 | 0 | 0 | 98% | 0 | 0 | 95% | 0 | 0 | 0 | 95% | 100% | 0 |
| 1804 | RNA-binding protein 34 OS=Homo sapiens GN=RBM34 PE=1 SV=2 | RBM34 | 49 | 0.59 | 0 | 0 | 0 | 0 | 95% | 0 | 0 | 0 | 0 | 100% | 0 | 12% |
| 1805 | Serine/threonine-protein kinase PRP4 homolog OS=Homo sapiens GN=PRPF4B PE=1 SV=3 | PRP4B | 117 | 0.24 | 5% | 0 | 11% | 0 | 0 | 0 | 0 | 0 | 95% | 100% | 95% | 0 |
| 1806 | Dynactin subunit 1 OS=Homo sapiens GN=DCTN1 PE=1 SV=3 | DCTN1 | 142 | 0.12 | 0 | 0 | 0 | 0 | 100% | 95% | 0 | 99% | 100% | 0 | 0 | 0 |
| 1807 | Insulin-like growth factor-binding protein 5 OS=Homo sapiens GN=IGFBP5 PE=1 SV=1 | IBP5 | 31 | 0.03 | 95% | 100% | 95% | 0 | 0 | 0 | 100% | 23% | 0 | 0 | 0 | 0 |
| 1808 | Putative RNA-binding protein Luc7-like 2 OS=Homo sapiens GN=LUC7L2 PE=1 SV=2 | LC7L2 | 47 | 0.19 | 0 | 26% | 0 | 0 | 0 | 0 | 99% | 100% | 0 | 0 | 0 | 95% |
| 1809 | Sentrin-specific protease 3 OS=Homo sapiens GN=SENP3 PE=1 SV=2 | SENP3 | 65 | 0.89 | 95% | 0 | 99% | 0 | 95% | 0 | 95% | 0 | 95% | 0 | 95% | 95% |
| 1810 | LMBR1 domain-containing protein 2 OS=Homo sapiens GN=LMBRD2 PE=1 SV=1 | LMBD2 | 81 | 0.018 | 0 | 0 | 0 | 95% | 95% | 95% | 0 | 0 | 0 | 0 | 95% | 99% |
| 1811 | Torsin-1B OS=Homo sapiens GN=TOR1B PE=1 SV=2 | TOR1B | 38 | 0.17 | 0 | 45% | 95% | 0 | 94% | 99% | 0 | 53% | 0 | 0 | 0 | 0 |
| 1812 | Mitochondrial glutamate carrier 1 OS=Homo sapiens GN=SLC25A22 PE=1 SV=1 | GHC1 | 34 | 0.44 | 0 | 0 | 0 | 0 | 0 | 97% | 0 | 0 | 0 | 60% | 95% | 99% |
| 1813 | Reticulocalbin-2 OS=Homo sapiens GN=RCN2 PE=1 SV=1 | RCN2 | 37 | 0.91 | 95% | 0 | 95% | 95% | 0 | 0 | 0 | 0 | 95% | 99% | 95% | 0 |
| 1814 | Beta-1,3-galactosyltransferase 6 OS=Homo sapiens GN=B3GALT6 PE=1 SV=2 | B3GT6 | 37 | 0.8 | 0 | 0 | 95% | 7% | 95% | 0 | 22% | 46% | 0 | 0 | 8% | 99% |
| 1815 | Decorin OS=Homo sapiens GN=DCN PE=1 SV=1 | PGS2 | 40 | 0.44 | 0 | 19% | 0 | 0 | 0 | 0 | 0 | 0 | 0 | 0 | 10% | 97% |
| 1816 | DnaJ homolog subfamily B member 12 OS=Homo sapiens GN=DNAJB12 PE=1 SV=4 | DJB12 | 42 | 0.07 | 0 | 0 | 0 | 0 | 95% | 95% | 95% | 97% | 95% | 0 | 95% | 0 |
| 1817 | NADH dehydrogenase [ubiquinone] iron-sulfur protein 8, mitochondrial OS=Homo sapiens GN=NDUFS8 PE=1 SV=1 | NDUS8 | 24 | 0.61 | 95% | 95% | 95% | 95% | 0 | 0 | 0 | 86% | 0 | 95% | 95% | 51% |
| 1818 | Palmitoyl-protein thioesterase 1 OS=Homo sapiens GN=PPT1 PE=1 SV=1 | PPT1 | 34 | 0.98 | 0 | 95% | 95% | 95% | 95% | 0 | 0 | 95% | 95% | 95% | 0 | 95% |
| 1819 | Protein transport protein Sec61 subunit gamma OS=Homo sapiens GN=SEC61G PE=1 SV=1 | SC61G | 8 | 0.58 | 14% | 95% | 66% | 95% | 11% | 0 | 0 | 0 | 13% | 0 | 0 | 65% |
| 1820 | UDP-glucuronic acid decarboxylase 1 OS=Homo sapiens GN=UXS1 PE=1 SV=1 | UXS1 | 48 | 0.004 | 0 | 51% | 10% | 95% | 95% | 95% | 0 | 0 | 0 | 0 | 95% | 0 |
| 1821 | Cytochrome c oxidase subunit 7A2, mitochondrial OS=Homo sapiens GN=COX7A2 PE=1 SV=1 | CX7A2 | 9 | 0.062 | 0 | 43% | 95% | 57% | 82% | 95% | 0 | 0 | 0 | 95% | 95% | 95% |
| 1822 | Protein Hook homolog 2 OS=Homo sapiens GN=HOOK2 PE=1 SV=3 | HOOK2 | 83 | 0.53 | 0 | 0 | 85% | 13% | 0 | 95% | 0 | 95% | 0 | 95% | 95% | 0 |
| 1823 | Prostaglandin E synthase OS=Homo sapiens GN=PTGES PE=1 SV=2 | PTGES | 17 | 0.013 | 95% | 95% | 95% | 0 | 95% | 0 | 0 | 0 | 0 | 95% | 95% | 95% |
| 1824 | Ras-related protein Rab-12 OS=Homo sapiens GN=RAB12 PE=1 SV=3 | RAB12 | 27 | 0.6 | 0 | 95% | 95% | 0 | 95% | 0 | 0 | 90% | 16% | 95% | 95% | 95% |
| 1825 | RNA binding protein fox-1 homolog 2 OS=Homo sapiens GN=RBFOX2 PE=1 SV=3 | RFOX2 | 41 | 0.065 | 0 | 95% | 95% | 0 | 95% | 95% | 95% | 95% | 95% | 7% | 0 | 0 |
| 1826 | Secretory carrier-associated membrane protein 4 OS=Homo sapiens GN=SCAMP4 PE=2 SV=1 | SCAM4 | 26 | 0.11 | 0 | 0 | 0 | 0 | 0 | 95% | 95% | 0 | 42% | 95% | 80% | 95% |
| 1827 | Thioredoxin domain-containing protein 12 OS=Homo sapiens GN=TXNDC12 PE=1 SV=1 | TXD12 | 19 | 0.12 | 0 | 82% | 84% | 0 | 95% | 71% | 16% | 0 | 0 | 64% | 63% | 6% |
| 1828 | Exosome component 10 OS=Homo sapiens GN=EXOSC10 PE=1 SV=2 | EXOSX | 101 | 0.44 | 0 | 40% | 0 | 0 | 0 | 0 | 0 | 0 | 6% | 95% | 0 | 0 |
| 1829 | Pre-mRNA 3'-end-processing factor FIP1 OS=Homo sapiens GN=FIP1L1 PE=1 SV=1 | FIP1 | 67 | 0.21 | 0 | 95% | 0 | 0 | 0 | 0 | 0 | 0 | 0 | 0 | 100% | 95% |
| 1830 | Nestin OS=Homo sapiens GN=NES PE=1 SV=2 | NEST | 177 | 0.44 | 0 | 0 | 0 | 0 | 0 | 0 | 100% | 99% | 0 | 0 | 0 | 0 |
| 1831 | U3 small nucleolar RNA-interacting protein 2 OS=Homo sapiens GN=RRP9 PE=1 SV=1 | U3IP2 | 52 | 0.57 | 0 | 11% | 39% | 0 | 95% | 0 | 0 | 0 | 0 | 0 | 0 | 100% |
| 1832 | Protein MRVI1 OS=Homo sapiens GN=MRVI1 PE=1 SV=2 | MRVI1 | 96 | 0.44 | 28% | 63% | 66% | 0 | 0 | 0 | 100% | 0 | 0 | 0 | 0 | 0 |
| 1833 | Pentraxin-related protein PTX3 OS=Homo sapiens GN=PTX3 PE=1 SV=3 | PTX3 | 42 | 0.2 | 0 | 95% | 95% | 0 | 0 | 0 | 0 | 0 | 0 | 100% | 0 | 100% |
| 1834 | Protein transport protein Sec31A OS=Homo sapiens GN=SEC31A PE=1 SV=3 | SC31A | 133 | 0.4 | 0 | 48% | 0 | 0 | 0 | 0 | 95% | 0 | 95% | 100% | 0 | 52% |
| 1835 | Thioredoxin-interacting protein OS=Homo sapiens GN=TXNIP PE=1 SV=1 | TXNIP | 44 | 0.00026 | 95% | 100% | 95% | 0 | 0 | 0 | 0 | 0 | 0 | 95% | 95% | 95% |
| 1836 | Cytochrome P450 2U1 OS=Homo sapiens GN=CYP2U1 PE=1 SV=1 | CP2U1 | 62 | 0.0018 | 0 | 95% | 0 | 95% | 100% | 95% | 0 | 0 | 0 | 12% | 0 | 0 |
| 1837 | Epididymal secretory protein E1 OS=Homo sapiens GN=NPC2 PE=1 SV=1 | NPC2 | 17 | 0.26 | 0 | 100% | 95% | 0 | 0 | 0 | 0 | 0 | 0 | 27% | 0 | 100% |
| 1838 | Transforming growth factor beta-2 OS=Homo sapiens GN=TGFB2 PE=1 SV=1 | TGFB2 | 48 | 0.052 | 0 | 0 | 0 | 66% | 100% | 100% | 0 | 0 | 0 | 0 | 62% | 0 |
| 1839 | Endoplasmic reticulum metallopeptidase 1 OS=Homo sapiens GN=ERMP1 PE=1 SV=2 | ERMP1 | 100 | 0.58 | 0 | 95% | 95% | 0 | 95% | 0 | 0 | 0 | 0 | 31% | 29% | 100% |
| 1840 | SCAN domain-containing protein 3 OS=Homo sapiens GN=SCAND3 PE=2 SV=1 | SCND3 | 152 | 0.44 | 0 | 0 | 0 | 0 | 0 | 8% | 0 | 0 | 100% | 0 | 83% | 0 |
| 1841 | Microsomal glutathione S-transferase 3 OS=Homo sapiens GN=MGST3 PE=1 SV=1 | MGST3 | 17 | < 0.00010 | 100% | 95% | 95% | 0 | 0 | 0 | 95% | 95% | 95% | 0 | 0 | 0 |
| 1842 | Transcription factor A, mitochondrial OS=Homo sapiens GN=TFAM PE=1 SV=1 | TFAM | 29 | 0.28 | 0 | 95% | 100% | 0 | 0 | 100% | 0 | 0 | 0 | 0 | 0 | 0 |
| 1843 | Phosphatidylinositol 4-kinase type 2-beta OS=Homo sapiens GN=PI4K2B PE=1 SV=1 | P4K2B | 55 | 0.83 | 95% | 0 | 0 | 0 | 95% | 95% | 0 | 100% | 0 | 95% | 0 | 18% |
| 1844 | Proteasome subunit alpha type-1 OS=Homo sapiens GN=PSMA1 PE=1 SV=1 | PSA1 | 30 | 0.052 | 0 | 0 | 13% | 0 | 0 | 0 | 100% | 12% | 100% | 0 | 0 | 0 |
| 1845 | Multiple inositol polyphosphate phosphatase 1 OS=Homo sapiens GN=MINPP1 PE=1 SV=1 | MINP1 | 55 | 0.013 | 0 | 0 | 0 | 95% | 95% | 0 | 0 | 0 | 9% | 95% | 95% | 100% |
| 1846 | Non-histone chromosomal protein HMG-14 OS=Homo sapiens GN=HMGN1 PE=1 SV=3 | HMGN1 | 11 | 0.36 | 0 | 95% | 95% | 0 | 100% | 95% | 0 | 0 | 0 | 0 | 0 | 95% |
| 1847 | Cytochrome c oxidase protein 20 homolog OS=Homo sapiens GN=COX20 PE=1 SV=2 | COX20 | 13 | 0.013 | 0 | 0 | 0 | 0 | 95% | 100% | 0 | 0 | 0 | 95% | 95% | 95% |
| 1848 | Q9P2K8-DECOY | Q9P2K8-DECOY | ? | 0.44 | 0 | 0 | 0 | 0 | 0 | 9% | 6% | 0 | 0 | 11% | 100% | 0 |
| 1849 | Cullin-4A OS=Homo sapiens GN=CUL4A PE=1 SV=3 | CUL4A | 88 | 0.2 | 0 | 0 | 33% | 99% | 95% | 0 | 0 | 27% | 97% | 0 | 0 | 0 |
| 1850 | Inter-alpha-trypsin inhibitor heavy chain H3 OS=Homo sapiens GN=ITIH3 PE=1 SV=2 | ITIH3 | 100 | 0.59 | 0 | 95% | 0 | 0 | 0 | 99% | 0 | 0 | 84% | 0 | 0 | 0 |
| 1851 | Putative ciliary rootlet coiled-coil protein-like 3 protein OS=Homo sapiens PE=5 SV=2 | CROL3 | 248 | 0.44 | 0 | 53% | 40% | 0 | 0 | 0 | 0 | 99% | 0 | 0 | 0 | 0 |
| 1852 | Obscurin OS=Homo sapiens GN=OBSCN PE=1 SV=3 | OBSCN | 868 | 0.44 | 0 | 0 | 0 | 0 | 0 | 0 | 88% | 0 | 0 | 0 | 0 | 99% |
| 1853 | Atlastin-2 OS=Homo sapiens GN=ATL2 PE=1 SV=2 | ATLA2 | 66 | 0.1 | 0 | 0 | 0 | 98% | 38% | 0 | 99% | 97% | 35% | 0 | 43% | 99% |
| 1854 | Cleavage stimulation factor subunit 2 tau variant OS=Homo sapiens GN=CSTF2T PE=1 SV=1 | CSTFT | 64 | 0.052 | 0 | 0 | 0 | 0 | 0 | 0 | 0 | 0 | 0 | 93% | 6% | 99% |
| 1855 | P20585-DECOY | P20585-DECOY | ? | 0.24 | 95% | 0 | 0 | 0 | 0 | 10% | 0 | 19% | 0 | 98% | 91% | 0 |
| 1856 | Dedicator of cytokinesis protein 11 OS=Homo sapiens GN=DOCK11 PE=1 SV=2 | DOC11 | 238 | 0.44 | 0 | 8% | 0 | 98% | 0 | 6% | 0 | 0 | 5% | 0 | 0 | 0 |
| 1857 | Tail-anchored protein insertion receptor WRB OS=Homo sapiens GN=WRB PE=1 SV=2 | WRB | 20 | 0.79 | 0 | 81% | 0 | 95% | 0 | 0 | 14% | 0 | 81% | 0 | 13% | 14% |
| 1858 | BCL2/adenovirus E1B 19 kDa protein-interacting protein 3-like OS=Homo sapiens GN=BNIP3L PE=1 SV=1 | BNI3L | 24 | 0.035 | 0 | 95% | 95% | 0 | 0 | 9% | 0 | 0 | 0 | 95% | 95% | 95% |
| 1859 | Protein BUD31 homolog OS=Homo sapiens GN=BUD31 PE=1 SV=2 | BUD31 | 17 | 0.098 | 0 | 0 | 0 | 0 | 95% | 5% | 95% | 95% | 0 | 95% | 95% | 95% |
| 1860 | Protein LLP homolog OS=Homo sapiens GN=LLPH PE=2 SV=1 | LLPH | 15 | 0.41 | 0 | 95% | 95% | 0 | 0 | 0 | 9% | 0 | 95% | 95% | 0 | 95% |
| 1861 | Mesencephalic astrocyte-derived neurotrophic factor OS=Homo sapiens GN=MANF PE=1 SV=3 | MANF | 21 | 0.16 | 0 | 95% | 95% | 0 | 0 | 0 | 95% | 50% | 95% | 0 | 12% | 0 |
| 1862 | Major facilitator superfamily domain-containing protein 1 OS=Homo sapiens GN=MFSD1 PE=2 SV=2 | MFSD1 | 51 | 0.077 | 0 | 0 | 0 | 53% | 95% | 0 | 95% | 95% | 95% | 0 | 95% | 95% |
| 1863 | DNA-directed RNA polymerase II subunit RPB3 OS=Homo sapiens GN=POLR2C PE=1 SV=2 | RPB3 | 31 | 0.22 | 0 | 0 | 0 | 0 | 0 | 0 | 95% | 0 | 20% | 95% | 9% | 95% |
| 1864 | Structural maintenance of chromosomes protein 4 OS=Homo sapiens GN=SMC4 PE=1 SV=2 | SMC4 | 147 | 0.44 | 83% | 0 | 0 | 0 | 0 | 95% | 0 | 0 | 0 | 0 | 0 | 0 |
| 1865 | Ubiquitin-conjugating enzyme E2 G2 OS=Homo sapiens GN=UBE2G2 PE=1 SV=1 | UB2G2 | 19 | 0.38 | 0 | 0 | 0 | 0 | 95% | 0 | 95% | 0 | 95% | 95% | 0 | 95% |
| 1866 | Protein C19orf12 OS=Homo sapiens GN=C19orf12 PE=1 SV=3 | CS012 | 16 | 0.44 | 0 | 0 | 0 | 0 | 0 | 0 | 13% | 0 | 95% | 0 | 0 | 0 |
| 1867 | O15054-DECOY | O15054-DECOY | ? | 0.59 | 6% | 57% | 92% | 0 | 0 | 0 | 0 | 0 | 0 | 0 | 73% | 0 |
| 1868 | 60S ribosomal protein L7-like 1 OS=Homo sapiens GN=RPL7L1 PE=1 SV=1 | RL7L | 29 | 0.2 | 0 | 0 | 0 | 0 | 95% | 0 | 0 | 0 | 0 | 0 | 100% | 95% |
| 1869 | Regulator of microtubule dynamics protein 3 OS=Homo sapiens GN=RMDN3 PE=1 SV=2 | RMD3 | 52 | 0.00096 | 0 | 0 | 0 | 0 | 0 | 0 | 0 | 0 | 0 | 99% | 100% | 95% |
| 1870 | Polypyrimidine tract-binding protein 3 OS=Homo sapiens GN=PTBP3 PE=1 SV=2 | PTBP3 | 60 | 0.00021 | 21% | 0 | 0 | 0 | 22% | 0 | 25% | 16% | 20% | 100% | 100% | 100% |
| 1871 | C2 domain-containing protein 2 OS=Homo sapiens GN=C2CD2 PE=1 SV=2 | CU025 | 76 | 0.68 | 95% | 95% | 0 | 95% | 0 | 0 | 100% | 0 | 0 | 0 | 0 | 0 |
| 1872 | Immunoglobulin superfamily containing leucine-rich repeat protein OS=Homo sapiens GN=ISLR PE=1 SV=1 | ISLR | 46 | < 0.00010 | 100% | 100% | 100% | 0 | 0 | 0 | 0 | 0 | 0 | 0 | 0 | 0 |
| 1873 | Ragulator complex protein LAMTOR5 OS=Homo sapiens GN=LAMTOR5 PE=1 SV=1 | LTOR5 | 10 | 0.096 | 0 | 0 | 27% | 0 | 0 | 0 | 98% | 95% | 100% | 0 | 0 | 0 |
| 1874 | NADH dehydrogenase [ubiquinone] 1 beta subcomplex subunit 9 OS=Homo sapiens GN=NDUFB9 PE=1 SV=3 | NDUB9 | 22 | 0.053 | 0 | 0 | 0 | 95% | 100% | 0 | 0 | 0 | 0 | 95% | 95% | 95% |
| 1875 | Nucleolar complex protein 4 homolog OS=Homo sapiens GN=NOC4L PE=1 SV=1 | NOC4L | 58 | 0.57 | 0 | 0 | 0 | 0 | 0 | 95% | 0 | 0 | 0 | 100% | 66% | 84% |
| 1876 | 26S proteasome non-ATPase regulatory subunit 2 OS=Homo sapiens GN=PSMD2 PE=1 SV=3 | PSMD2 | 100 | 0.096 | 0 | 0 | 0 | 0 | 0 | 0 | 0 | 100% | 100% | 0 | 0 | 82% |
| 1877 | Solute carrier family 15 member 4 OS=Homo sapiens GN=SLC15A4 PE=2 SV=1 | S15A4 | 62 | 0.008 | 0 | 0 | 95% | 0 | 0 | 0 | 95% | 100% | 100% | 0 | 0 | 0 |
| 1878 | SWI/SNF-related matrix-associated actin-dependent regulator of chromatin subfamily E member 1 OS=Homo sapiens GN=SMARCE1 PE=1 SV=2 | SMCE1 | 47 | 0.17 | 0 | 0 | 0 | 0 | 0 | 0 | 95% | 0 | 100% | 0 | 100% | 95% |
| 1879 | FYVE and coiled-coil domain-containing protein 1 OS=Homo sapiens GN=FYCO1 PE=1 SV=3 | FYCO1 | 167 | 0.093 | 0 | 100% | 100% | 0 | 0 | 0 | 0 | 0 | 0 | 0 | 0 | 0 |
| 1880 | E3 ubiquitin-protein ligase RNF13 OS=Homo sapiens GN=RNF13 PE=1 SV=1 | RNF13 | 43 | 0.18 | 0 | 95% | 0 | 0 | 0 | 0 | 100% | 0 | 100% | 0 | 0 | 0 |
| 1881 | Lon protease homolog, mitochondrial OS=Homo sapiens GN=LONP1 PE=1 SV=2 | LONM | 106 | 0.052 | 0 | 0 | 0 | 0 | 0 | 0 | 0 | 0 | 0 | 10% | 100% | 100% |
| 1882 | RuvB-like 2 OS=Homo sapiens GN=RUVBL2 PE=1 SV=3 | RUVB2 | 51 | 0.24 | 0 | 0 | 0 | 0 | 0 | 64% | 0 | 0 | 95% | 95% | 100% | 0 |
| 1883 | Inosine-5'-monophosphate dehydrogenase 2 OS=Homo sapiens GN=IMPDH2 PE=1 SV=2 | IMDH2 | 56 | 0.052 | 0 | 0 | 0 | 0 | 0 | 0 | 100% | 63% | 100% | 0 | 0 | 0 |
| 1884 | Polymerase delta-interacting protein 3 OS=Homo sapiens GN=POLDIP3 PE=1 SV=2 | PDIP3 | 46 | 0.0031 | 0 | 95% | 95% | 0 | 0 | 0 | 0 | 0 | 0 | 100% | 78% | 95% |
| 1885 | GDP-Man:Man(3)GlcNAc(2)-PP-Dol alpha-1,2-mannosyltransferase OS=Homo sapiens GN=ALG11 PE=1 SV=2 | ALG11 | 56 | 0.44 | 0 | 0 | 0 | 0 | 100% | 0 | 0 | 13% | 84% | 0 | 0 | 0 |
| 1886 | Uncharacterized protein C17orf59 OS=Homo sapiens GN=C17orf59 PE=1 SV=2 | CQ059 | 37 | 0.12 | 0 | 100% | 95% | 0 | 0 | 0 | 95% | 95% | 8% | 0 | 0 | 0 |
| 1887 | T-cell immunomodulatory protein OS=Homo sapiens GN=ITFG1 PE=1 SV=1 | TIP | 68 | 0.18 | 0 | 0 | 100% | 15% | 0 | 0 | 89% | 95% | 0 | 12% | 0 | 0 |
| 1888 | GRAM domain-containing protein 3 OS=Homo sapiens GN=GRAMD3 PE=1 SV=1 | GRAM3 | 48 | 0.57 | 0 | 100% | 95% | 0 | 0 | 95% | 0 | 0 | 0 | 95% | 0 | 0 |
| 1889 | Transmembrane and coiled-coil domain-containing protein 3 OS=Homo sapiens GN=TMCO3 PE=2 SV=1 | TMCO3 | 76 | 0.052 | 0 | 0 | 0 | 0 | 95% | 100% | 0 | 0 | 0 | 5% | 0 | 0 |
| 1890 | Hydrocephalus-inducing protein homolog OS=Homo sapiens GN=HYDIN PE=1 SV=3 | HYDIN | 576 | 0.59 | 0 | 100% | 0 | 0 | 11% | 0 | 0 | 95% | 0 | 0 | 0 | 0 |
| 1891 | Protein PRRC2B OS=Homo sapiens GN=PRRC2B PE=1 SV=2 | PRC2B | 243 | 0.052 | 0 | 0 | 0 | 0 | 67% | 100% | 7% | 0 | 0 | 0 | 0 | 0 |
| 1892 | RNA-binding protein 26 OS=Homo sapiens GN=RBM26 PE=1 SV=3 | RBM26 | 114 | 0.19 | 0 | 95% | 0 | 0 | 0 | 0 | 0 | 0 | 0 | 0 | 99% | 100% |
| 1893 | Surfeit locus protein 6 OS=Homo sapiens GN=SURF6 PE=1 SV=3 | SURF6 | 41 | 0.44 | 0 | 0 | 0 | 0 | 0 | 0 | 0 | 0 | 0 | 100% | 0 | 0 |
| 1894 | Glutaminase kidney isoform, mitochondrial OS=Homo sapiens GN=GLS PE=1 SV=1 | GLSK | 73 | 0.44 | 0 | 0 | 0 | 0 | 0 | 100% | 19% | 36% | 10% | 0 | 0 | 0 |
| 1895 | Engulfment and cell motility protein 2 OS=Homo sapiens GN=ELMO2 PE=1 SV=2 | ELMO2 | 83 | 0.44 | 0 | 0 | 0 | 0 | 0 | 0 | 15% | 0 | 100% | 0 | 0 | 0 |
| 1896 | Armadillo repeat-containing protein 10 OS=Homo sapiens GN=ARMC10 PE=1 SV=1 | ARM10 | 38 | 0.4 | 100% | 0 | 81% | 0 | 95% | 0 | 0 | 0 | 0 | 95% | 95% | 0 |
| 1897 | Septin-10 OS=Homo sapiens GN=SEPT10 PE=1 SV=2 | SEP10 | 53 | 0.038 | 0 | 98% | 95% | 0 | 0 | 0 | 0 | 0 | 0 | 82% | 95% | 100% |
| 1898 | Alpha-mannosidase 2x OS=Homo sapiens GN=MAN2A2 PE=2 SV=3 | MA2A2 | 131 | 0.18 | 0 | 0 | 0 | 99% | 0 | 99% | 0 | 0 | 0 | 0 | 95% | 28% |
| 1899 | Q6ZQQ6-DECOY | Q6ZQQ6-DECOY | ? | 0.44 | 99% | 70% | 0 | 0 | 0 | 0 | 0 | 0 | 0 | 0 | 0 | 0 |
| 1900 | Myomesin-1 OS=Homo sapiens GN=MYOM1 PE=1 SV=2 | MYOM1 | 188 | 0.44 | 0 | 0 | 0 | 16% | 99% | 0 | 0 | 0 | 0 | 0 | 0 | 0 |
| 1901 | Ectonucleoside triphosphate diphosphohydrolase 4 OS=Homo sapiens GN=ENTPD4 PE=1 SV=1 | ENTP4 | 70 | 0.12 | 0 | 0 | 0 | 95% | 0 | 99% | 0 | 0 | 0 | 95% | 95% | 0 |
| 1902 | ADP-ribosylation factor-like protein 15 OS=Homo sapiens GN=ARL15 PE=1 SV=1 | ARL15 | 23 | 0.012 | 95% | 95% | 95% | 0 | 95% | 99% | 0 | 0 | 0 | 0 | 0 | 0 |
| 1903 | 39S ribosomal protein L39, mitochondrial OS=Homo sapiens GN=MRPL39 PE=1 SV=3 | RM39 | 39 | 0.38 | 0 | 0 | 0 | 0 | 0 | 95% | 72% | 95% | 0 | 99% | 0 | 95% |
| 1904 | Alpha-1,6-mannosylglycoprotein 6-beta-N-acetylglucosaminyltransferase A OS=Homo sapiens GN=MGAT5 PE=1 SV=1 | MGT5A | 85 | 0.053 | 0 | 0 | 0 | 95% | 98% | 95% | 0 | 0 | 95% | 95% | 0 | 0 |
| 1905 | Mitochondrial carrier homolog 1 OS=Homo sapiens GN=MTCH1 PE=1 SV=1 | MTCH1 | 42 | 0.013 | 0 | 0 | 8% | 95% | 73% | 0 | 0 | 0 | 0 | 98% | 95% | 95% |
| 1906 | Serine/threonine-protein kinase VRK2 OS=Homo sapiens GN=VRK2 PE=1 SV=3 | VRK2 | 58 | 0.29 | 0 | 0 | 95% | 98% | 0 | 95% | 0 | 0 | 0 | 95% | 95% | 0 |
| 1907 | Putative 60S ribosomal protein L39-like 5 OS=Homo sapiens GN=RPL39P5 PE=5 SV=2 | R39L5 (+1) | 6 | 0.0031 | 0 | 95% | 98% | 0 | 0 | 0 | 0 | 0 | 0 | 95% | 95% | 95% |
| 1908 | Chromatin assembly factor 1 subunit B OS=Homo sapiens GN=CHAF1B PE=1 SV=1 | CAF1B | 61 | 0.2 | 0 | 0 | 0 | 0 | 96% | 95% | 95% | 0 | 0 | 0 | 0 | 0 |
| 1909 | U6 snRNA-associated Sm-like protein LSm2 OS=Homo sapiens GN=LSM2 PE=1 SV=1 | LSM2 | 11 | 0.12 | 95% | 95% | 95% | 0 | 95% | 0 | 95% | 95% | 0 | 0 | 0 | 0 |
| 1910 | AP-1 complex subunit mu-1 OS=Homo sapiens GN=AP1M1 PE=1 SV=3 | AP1M1 | 49 | 0.12 | 0 | 95% | 95% | 0 | 49% | 63% | 0 | 0 | 0 | 0 | 95% | 95% |
| 1911 | AP-3 complex subunit delta-1 OS=Homo sapiens GN=AP3D1 PE=1 SV=1 | AP3D1 | 130 | 0.59 | 0 | 0 | 95% | 0 | 0 | 46% | 0 | 95% | 22% | 0 | 0 | 0 |
| 1912 | Cullin-associated NEDD8-dissociated protein 1 OS=Homo sapiens GN=CAND1 PE=1 SV=2 | CAND1 | 136 | 0.22 | 0 | 0 | 0 | 0 | 95% | 95% | 0 | 95% | 0 | 0 | 37% | 0 |
| 1913 | Membrane protein FAM174A OS=Homo sapiens GN=FAM174A PE=2 SV=1 | F174A | 20 | 0.38 | 0 | 0 | 0 | 45% | 0 | 95% | 95% | 95% | 0 | 70% | 0 | 95% |
| 1914 | Rho GDP-dissociation inhibitor 1 OS=Homo sapiens GN=ARHGDIA PE=1 SV=3 | GDIR1 | 23 | 0.93 | 95% | 0 | 0 | 0 | 95% | 0 | 0 | 95% | 0 | 0 | 0 | 95% |
| 1915 | 1,4-alpha-glucan-branching enzyme OS=Homo sapiens GN=GBE1 PE=1 SV=3 | GLGB | 80 | 0.02 | 95% | 95% | 95% | 0 | 0 | 0 | 0 | 15% | 95% | 0 | 0 | 0 |
| 1916 | LIM and senescent cell antigen-like-containing domain protein 3-like OS=Homo sapiens GN=LIMS3L PE=4 SV=1 | LIM3L (+1) | 13 | 0.48 | 0 | 0 | 0 | 0 | 0 | 95% | 0 | 95% | 95% | 42% | 9% | 95% |
| 1917 | NADH dehydrogenase [ubiquinone] 1 alpha subcomplex subunit 2 OS=Homo sapiens GN=NDUFA2 PE=1 SV=3 | NDUA2 | 11 | 0.0088 | 0 | 0 | 0 | 0 | 95% | 95% | 0 | 0 | 0 | 95% | 95% | 95% |
| 1918 | NADH dehydrogenase [ubiquinone] 1 beta subcomplex subunit 11, mitochondrial OS=Homo sapiens GN=NDUFB11 PE=1 SV=1 | NDUBB | 17 | 0.24 | 0 | 0 | 0 | 0 | 95% | 0 | 0 | 0 | 0 | 95% | 0 | 89% |
| 1919 | NEDD4 family-interacting protein 2 OS=Homo sapiens GN=NDFIP2 PE=1 SV=2 | NFIP2 | 36 | 0.33 | 95% | 95% | 0 | 0 | 95% | 95% | 95% | 95% | 0 | 0 | 0 | 0 |
| 1920 | RNA-binding protein 45 OS=Homo sapiens GN=RBM45 PE=2 SV=1 | RBM45 | 54 | 0.16 | 0 | 0 | 0 | 0 | 0 | 0 | 95% | 0 | 95% | 95% | 95% | 0 |
| 1921 | Sickle tail protein homolog OS=Homo sapiens GN=KIAA1217 PE=1 SV=2 | SKT | 214 | 0.59 | 0 | 6% | 95% | 0 | 69% | 0 | 0 | 0 | 0 | 12% | 84% | 0 |
| 1922 | Signal recognition particle 54 kDa protein OS=Homo sapiens GN=SRP54 PE=1 SV=1 | SRP54 | 56 | 0.59 | 0 | 0 | 0 | 0 | 0 | 0 | 0 | 70% | 79% | 95% | 0 | 0 |
| 1923 | Transmembrane protein 181 OS=Homo sapiens GN=TMEM181 PE=1 SV=2 | TM181 | 69 | 0.44 | 0 | 18% | 0 | 0 | 94% | 0 | 0 | 0 | 0 | 30% | 0 | 95% |
| 1924 | Transmembrane protein 237 OS=Homo sapiens GN=TMEM237 PE=1 SV=2 | TM237 | 46 | 0.58 | 0 | 95% | 0 | 85% | 10% | 0 | 0 | 0 | 0 | 0 | 0 | 0 |
| 1925 | Transmembrane protein 19 OS=Homo sapiens GN=TMEM19 PE=2 SV=1 | TMM19 | 36 | 0.44 | 0 | 0 | 64% | 0 | 0 | 9% | 0 | 0 | 0 | 95% | 0 | 0 |
| 1926 | DNA topoisomerase 2-binding protein 1 OS=Homo sapiens GN=TOPBP1 PE=1 SV=3 | TOPB1 | 171 | 0.44 | 0 | 30% | 95% | 0 | 0 | 0 | 0 | 66% | 0 | 0 | 0 | 0 |
| 1927 | Trafficking protein particle complex subunit 11 OS=Homo sapiens GN=TRAPPC11 PE=1 SV=2 | TPC11 | 129 | 0.59 | 0 | 0 | 11% | 86% | 11% | 65% | 45% | 0 | 0 | 95% | 0 | 0 |
| 1928 | Cluster of Serpin B6 OS=Homo sapiens GN=SERPINB6 PE=1 SV=3 (SPB6_HUMAN) | SPB6 [2] | 43 | 0.44 | 0 | 63% | 0 | 0 | 0 | 0 | 0 | 0 | 0 | 7% | 100% | 10% |
| 1929 | Acyl-CoA desaturase OS=Homo sapiens GN=SCD PE=1 SV=2 | ACOD | 42 | 0.17 | 0 | 0 | 95% | 0 | 0 | 0 | 0 | 0 | 0 | 100% | 0 | 100% |
| 1930 | Serine beta-lactamase-like protein LACTB, mitochondrial OS=Homo sapiens GN=LACTB PE=1 SV=2 | LACTB | 61 | 0.64 | 95% | 0 | 95% | 0 | 0 | 0 | 95% | 0 | 0 | 100% | 0 | 0 |
| 1931 | 26S protease regulatory subunit 4 OS=Homo sapiens GN=PSMC1 PE=1 SV=1 | PRS4 | 49 | 0.2 | 0 | 59% | 0 | 0 | 95% | 0 | 95% | 100% | 0 | 0 | 0 | 0 |
| 1932 | Methionine--tRNA ligase, cytoplasmic OS=Homo sapiens GN=MARS PE=1 SV=2 | SYMC | 101 | 0.18 | 0 | 0 | 0 | 0 | 100% | 95% | 0 | 0 | 0 | 0 | 0 | 94% |
| 1933 | TBC1 domain family member 20 OS=Homo sapiens GN=TBC1D20 PE=1 SV=1 | TBC20 | 46 | 0.028 | 100% | 95% | 95% | 0 | 0 | 0 | 0 | 0 | 0 | 0 | 95% | 0 |
| 1934 | Tumor suppressor p53-binding protein 1 OS=Homo sapiens GN=TP53BP1 PE=1 SV=2 | TP53B | 214 | 0.097 | 0 | 100% | 95% | 0 | 0 | 98% | 0 | 0 | 0 | 0 | 0 | 0 |
| 1935 | Hemoglobin subunit alpha OS=Homo sapiens GN=HBA1 PE=1 SV=2 | HBA | 15 | 0.25 | 0 | 100% | 95% | 0 | 0 | 0 | 0 | 0 | 0 | 92% | 18% | 0 |
| 1936 | Lysyl oxidase homolog 2 OS=Homo sapiens GN=LOXL2 PE=1 SV=1 | LOXL2 | 87 | 0.44 | 0 | 0 | 0 | 0 | 0 | 0 | 0 | 100% | 73% | 0 | 0 | 0 |
| 1937 | Ragulator complex protein LAMTOR4 OS=Homo sapiens GN=LAMTOR4 PE=1 SV=1 | LTOR4 | 11 | 0.44 | 13% | 0 | 0 | 0 | 0 | 0 | 14% | 100% | 14% | 0 | 0 | 0 |
| 1938 | 26S proteasome non-ATPase regulatory subunit 11 OS=Homo sapiens GN=PSMD11 PE=1 SV=3 | PSD11 | 47 | 0.44 | 0 | 0 | 0 | 0 | 0 | 0 | 23% | 100% | 0 | 0 | 0 | 0 |
| 1939 | Short-chain dehydrogenase/reductase 3 OS=Homo sapiens GN=DHRS3 PE=1 SV=2 | DHRS3 | 34 | 0.44 | 100% | 77% | 12% | 0 | 0 | 0 | 0 | 0 | 0 | 0 | 0 | 0 |
| 1940 | SWI/SNF-related matrix-associated actin-dependent regulator of chromatin subfamily D member 2 OS=Homo sapiens GN=SMARCD2 PE=1 SV=3 | SMRD2 | 59 | 0.44 | 0 | 0 | 0 | 0 | 0 | 10% | 0 | 0 | 0 | 12% | 0 | 100% |
| 1941 | Pleiotropic regulator 1 OS=Homo sapiens GN=PLRG1 PE=1 SV=1 | PLRG1 | 57 | 0.23 | 0 | 100% | 95% | 0 | 88% | 0 | 0 | 0 | 0 | 0 | 0 | 0 |
| 1942 | ORM1-like protein 3 OS=Homo sapiens GN=ORMDL3 PE=1 SV=1 | ORML3 | 17 | 0.59 | 0 | 0 | 100% | 0 | 98% | 0 | 0 | 98% | 0 | 0 | 0 | 62% |
| 1943 | Proteasome subunit alpha type-7 OS=Homo sapiens GN=PSMA7 PE=1 SV=1 | PSA7 | 28 | 0.12 | 95% | 97% | 0 | 0 | 0 | 0 | 0 | 95% | 100% | 0 | 0 | 0 |
| 1944 | Sorting nexin-3 OS=Homo sapiens GN=SNX3 PE=1 SV=3 | SNX3 | 19 | 0.6 | 12% | 0 | 0 | 0 | 95% | 0 | 9% | 100% | 0 | 0 | 0 | 0 |
| 1945 | Methyltransferase-like protein 7A OS=Homo sapiens GN=METTL7A PE=1 SV=1 | MET7A | 28 | 0.6 | 95% | 45% | 43% | 0 | 0 | 0 | 0 | 0 | 0 | 100% | 0 | 0 |
| 1946 | 26S protease regulatory subunit 8 OS=Homo sapiens GN=PSMC5 PE=1 SV=1 | PRS8 | 46 | 0.6 | 0 | 0 | 0 | 0 | 0 | 0 | 0 | 100% | 0 | 0 | 95% | 0 |
| 1947 | Syntaxin-10 OS=Homo sapiens GN=STX10 PE=1 SV=1 | STX10 | 28 | 0.0028 | 0 | 95% | 0 | 0 | 0 | 0 | 100% | 95% | 95% | 0 | 0 | 0 |
| 1948 | Treacle protein OS=Homo sapiens GN=TCOF1 PE=1 SV=3 | TCOF | 152 | 0.052 | 0 | 0 | 0 | 0 | 0 | 27% | 0 | 0 | 0 | 95% | 28% | 100% |
| 1949 | Double-stranded RNA-binding protein Staufen homolog 2 OS=Homo sapiens GN=STAU2 PE=1 SV=1 | STAU2 | 63 | 0.44 | 0 | 0 | 0 | 0 | 0 | 0 | 0 | 0 | 0 | 0 | 44% | 100% |
| 1950 | A-kinase anchor protein 13 OS=Homo sapiens GN=AKAP13 PE=1 SV=2 | AKP13 | 308 | 0.59 | 13% | 95% | 0 | 0 | 0 | 0 | 0 | 0 | 100% | 0 | 0 | 0 |
| 1951 | Protein polybromo-1 OS=Homo sapiens GN=PBRM1 PE=1 SV=1 | PB1 | 193 | 0.44 | 0 | 0 | 0 | 0 | 0 | 0 | 0 | 0 | 0 | 84% | 0 | 100% |
| 1952 | WW domain-binding protein 11 OS=Homo sapiens GN=WBP11 PE=1 SV=1 | WBP11 | 70 | 0.6 | 0 | 0 | 0 | 0 | 14% | 10% | 95% | 14% | 0 | 0 | 100% | 0 |
| 1953 | G-rich sequence factor 1 OS=Homo sapiens GN=GRSF1 PE=1 SV=3 | GRSF1 | 53 | 0.052 | 0 | 0 | 0 | 0 | 0 | 0 | 0 | 97% | 100% | 0 | 0 | 0 |
| 1954 | Protein YIPF6 OS=Homo sapiens GN=YIPF6 PE=1 SV=2 | YIPF6 | 26 | 0.6 | 0 | 0 | 0 | 0 | 95% | 0 | 0 | 0 | 0 | 7% | 99% | 95% |
| 1955 | Eukaryotic translation initiation factor 5B OS=Homo sapiens GN=EIF5B PE=1 SV=4 | IF2P | 139 | < 0.00010 | 0 | 0 | 0 | 0 | 0 | 0 | 0 | 0 | 0 | 95% | 95% | 99% |
| 1956 | Alpha-L-iduronidase OS=Homo sapiens GN=IDUA PE=1 SV=2 | IDUA | 73 | 0.12 | 0 | 95% | 95% | 0 | 0 | 0 | 0 | 0 | 0 | 0 | 95% | 99% |
| 1957 | Eukaryotic translation initiation factor 4 gamma 3 OS=Homo sapiens GN=EIF4G3 PE=1 SV=2 | IF4G3 | 177 | 0.15 | 0 | 0 | 0 | 0 | 55% | 80% | 47% | 99% | 48% | 47% | 0 | 0 |
| 1958 | 26S proteasome non-ATPase regulatory subunit 1 OS=Homo sapiens GN=PSMD1 PE=1 SV=2 | PSMD1 | 106 | < 0.00010 | 0 | 0 | 0 | 0 | 0 | 0 | 95% | 99% | 95% | 0 | 0 | 0 |
| 1959 | Etoposide-induced protein 2.4 homolog OS=Homo sapiens GN=EI24 PE=1 SV=4 | EI24 | 39 | 0.44 | 0 | 63% | 98% | 0 | 14% | 95% | 0 | 0 | 0 | 0 | 0 | 0 |
| 1960 | Polyprenol reductase OS=Homo sapiens GN=SRD5A3 PE=1 SV=1 | PORED | 37 | 0.44 | 63% | 95% | 66% | 0 | 0 | 0 | 98% | 0 | 0 | 0 | 0 | 0 |
| 1961 | ER lumen protein retaining receptor 1 OS=Homo sapiens GN=KDELR1 PE=1 SV=1 | ERD21 | 25 | 0.15 | 0 | 95% | 95% | 0 | 0 | 0 | 95% | 98% | 0 | 0 | 0 | 0 |
| 1962 | DnaJ homolog subfamily C member 8 OS=Homo sapiens GN=DNAJC8 PE=1 SV=2 | DNJC8 | 30 | 0.44 | 0 | 0 | 0 | 0 | 0 | 0 | 0 | 0 | 0 | 95% | 91% | 97% |
| 1963 | CUGBP Elav-like family member 1 OS=Homo sapiens GN=CELF1 PE=1 SV=2 | CELF1 (+1) | 52 | 0.44 | 10% | 0 | 0 | 0 | 0 | 0 | 0 | 0 | 0 | 96% | 95% | 0 |
| 1964 | Golgi reassembly-stacking protein 1 OS=Homo sapiens GN=GORASP1 PE=1 SV=3 | GORS1 | 46 | 0.011 | 0 | 0 | 0 | 95% | 0 | 95% | 95% | 95% | 95% | 0 | 0 | 0 |
| 1965 | General transcription factor II-I OS=Homo sapiens GN=GTF2I PE=1 SV=2 | GTF2I | 112 | 0.014 | 0 | 0 | 0 | 95% | 0 | 95% | 0 | 0 | 0 | 95% | 95% | 95% |
| 1966 | Hippocalcin-like protein 1 OS=Homo sapiens GN=HPCAL1 PE=1 SV=3 | HPCL1 | 22 | 0.3 | 0 | 0 | 0 | 95% | 0 | 0 | 0 | 0 | 0 | 81% | 95% | 0 |
| 1967 | WD repeat-containing protein 36 OS=Homo sapiens GN=WDR36 PE=1 SV=1 | WDR36 | 105 | 0.44 | 0 | 5% | 14% | 95% | 0 | 0 | 0 | 0 | 0 | 0 | 5% | 0 |
| 1968 | Aldehyde dehydrogenase X, mitochondrial OS=Homo sapiens GN=ALDH1B1 PE=1 SV=3 | AL1B1 | 57 | < 0.00010 | 0 | 0 | 0 | 0 | 0 | 0 | 0 | 0 | 0 | 95% | 95% | 95% |
| 1969 | ATP synthase subunit a OS=Homo sapiens GN=MT-ATP6 PE=1 SV=1 | ATP6 | 25 | 0.8 | 0 | 0 | 95% | 0 | 0 | 0 | 0 | 93% | 0 | 24% | 91% | 0 |
| 1970 | C-1-tetrahydrofolate synthase, cytoplasmic OS=Homo sapiens GN=MTHFD1 PE=1 SV=3 | C1TC | 102 | 0.59 | 0 | 0 | 17% | 92% | 0 | 0 | 95% | 0 | 35% | 60% | 0 | 0 |
| 1971 | Caspase recruitment domain-containing protein 10 OS=Homo sapiens GN=CARD10 PE=2 SV=2 | CAR10 | 116 | 0.44 | 20% | 0 | 0 | 0 | 0 | 0 | 0 | 95% | 0 | 0 | 93% | 0 |
| 1972 | Cell division cycle and apoptosis regulator protein 1 OS=Homo sapiens GN=CCAR1 PE=1 SV=2 | CCAR1 | 133 | 0.44 | 33% | 0 | 0 | 0 | 0 | 0 | 94% | 95% | 0 | 0 | 26% | 0 |
| 1973 | Uncharacterized protein C6orf118 OS=Homo sapiens GN=C6orf118 PE=2 SV=1 | CF118 | 54 | 0.44 | 6% | 0 | 0 | 0 | 11% | 0 | 95% | 0 | 0 | 0 | 0 | 0 |
| 1974 | Destrin OS=Homo sapiens GN=DSTN PE=1 SV=3 | DEST | 19 | 0.24 | 0 | 0 | 26% | 0 | 0 | 0 | 0 | 95% | 0 | 14% | 95% | 95% |
| 1975 | Eukaryotic translation initiation factor 3 subunit G OS=Homo sapiens GN=EIF3G PE=1 SV=2 | EIF3G | 36 | 0.57 | 0 | 95% | 68% | 0 | 0 | 0 | 0 | 81% | 0 | 84% | 0 | 0 |
| 1976 | Exosome complex component RRP46 OS=Homo sapiens GN=EXOSC5 PE=1 SV=1 | EXOS5 | 25 | 0.052 | 0 | 0 | 0 | 0 | 0 | 0 | 95% | 95% | 0 | 0 | 74% | 0 |
| 1977 | N-acetylgalactosamine-6-sulfatase OS=Homo sapiens GN=GALNS PE=1 SV=1 | GALNS | 58 | 0.0039 | 0 | 95% | 57% | 0 | 0 | 0 | 0 | 0 | 0 | 90% | 95% | 95% |
| 1978 | Glioma tumor suppressor candidate region gene 2 protein OS=Homo sapiens GN=GLTSCR2 PE=1 SV=2 | GSCR2 | 54 | 0.6 | 0 | 0 | 0 | 0 | 0 | 9% | 15% | 93% | 55% | 0 | 0 | 95% |
| 1979 | sp|Q9BYK8|HELZ2_HUMAN-DECOY Helicase with zinc finger domain 2 OS=Homo sapiens GN=HELZ2... | HELZ2-DECOY (+1) | ? | 0.54 | 0 | 0 | 95% | 0 | 0 | 9% | 0 | 0 | 95% | 0 | 0 | 0 |
| 1980 | Homocysteine-responsive endoplasmic reticulum-resident ubiquitin-like domain member 1 protein OS=Homo sapiens GN=HERPUD1 PE=1 SV=1 | HERP1 | 44 | 0.2 | 6% | 0 | 0 | 0 | 0 | 0 | 0 | 95% | 95% | 95% | 0 | 0 |
| 1981 | Keratinocyte-associated protein 2 OS=Homo sapiens GN=KRTCAP2 PE=1 SV=2 | KTAP2 | 15 | 0.36 | 95% | 0 | 95% | 0 | 0 | 0 | 0 | 95% | 95% | 0 | 95% | 0 |
| 1982 | Lysophospholipid acyltransferase 5 OS=Homo sapiens GN=LPCAT3 PE=1 SV=1 | MBOA5 | 56 | 0.59 | 0 | 95% | 0 | 0 | 0 | 0 | 83% | 0 | 30% | 0 | 0 | 53% |
| 1983 | Nck-associated protein 5 OS=Homo sapiens GN=NCKAP5 PE=1 SV=2 | NCKP5 | 209 | 0.44 | 0 | 0 | 0 | 0 | 95% | 0 | 0 | 0 | 0 | 0 | 0 | 0 |
| 1984 | NADH dehydrogenase [ubiquinone] 1 beta subcomplex subunit 1 OS=Homo sapiens GN=NDUFB1 PE=1 SV=1 | NDUB1 | 7 | 0.0088 | 0 | 0 | 0 | 0 | 76% | 95% | 0 | 0 | 0 | 95% | 95% | 95% |
| 1985 | Pyruvate dehydrogenase protein X component, mitochondrial OS=Homo sapiens GN=PDHX PE=1 SV=3 | ODPX | 54 | 0.014 | 0 | 0 | 0 | 0 | 8% | 95% | 0 | 0 | 0 | 95% | 95% | 95% |
| 1986 | GDP-fucose protein O-fucosyltransferase 1 OS=Homo sapiens GN=POFUT1 PE=1 SV=1 | OFUT1 | 44 | 0.052 | 0 | 0 | 0 | 0 | 0 | 0 | 0 | 0 | 0 | 95% | 95% | 7% |
| 1987 | GDP-fucose protein O-fucosyltransferase 2 OS=Homo sapiens GN=POFUT2 PE=1 SV=3 | OFUT2 | 50 | 0.068 | 0 | 84% | 0 | 0 | 0 | 0 | 95% | 0 | 0 | 95% | 95% | 95% |
| 1988 | P13639-DECOY | P13639-DECOY | ? | 0.44 | 0 | 0 | 95% | 0 | 0 | 0 | 0 | 0 | 0 | 0 | 0 | 0 |
| 1989 | P43681-DECOY | P43681-DECOY | ? | 0.0028 | 0 | 95% | 0 | 0 | 0 | 0 | 95% | 95% | 95% | 0 | 0 | 0 |
| 1990 | Group XV phospholipase A2 OS=Homo sapiens GN=PLA2G15 PE=1 SV=2 | PAG15 | 47 | 0.19 | 9% | 95% | 16% | 0 | 0 | 0 | 0 | 0 | 0 | 95% | 0 | 95% |
| 1991 | Proteasome subunit beta type-3 OS=Homo sapiens GN=PSMB3 PE=1 SV=2 | PSB3 | 23 | 0.44 | 0 | 0 | 65% | 0 | 0 | 0 | 0 | 95% | 45% | 0 | 0 | 0 |
| 1992 | Periodic tryptophan protein 2 homolog OS=Homo sapiens GN=PWP2 PE=1 SV=2 | PWP2 | 102 | 0.87 | 0 | 95% | 0 | 0 | 95% | 0 | 0 | 0 | 95% | 95% | 95% | 0 |
| 1993 | Ribosomal RNA processing protein 1 homolog B OS=Homo sapiens GN=RRP1B PE=1 SV=3 | RRP1B | 84 | < 0.00010 | 0 | 0 | 0 | 0 | 0 | 35% | 0 | 0 | 0 | 95% | 95% | 95% |
| 1994 | Vesicle transport protein SFT2B OS=Homo sapiens GN=SFT2D2 PE=1 SV=1 | SFT2B | 18 | 0.2 | 0 | 0 | 26% | 0 | 87% | 95% | 95% | 0 | 47% | 0 | 0 | 0 |
| 1995 | WASH complex subunit strumpellin OS=Homo sapiens GN=KIAA0196 PE=1 SV=1 | STRUM | 134 | 0.052 | 0 | 0 | 0 | 0 | 0 | 0 | 0 | 0 | 0 | 95% | 0 | 95% |
| 1996 | Asparagine--tRNA ligase, cytoplasmic OS=Homo sapiens GN=NARS PE=1 SV=1 | SYNC | 63 | 0.16 | 0 | 0 | 0 | 0 | 0 | 0 | 95% | 0 | 95% | 0 | 95% | 95% |
| 1997 | General transcription factor IIH subunit 4 OS=Homo sapiens GN=GTF2H4 PE=2 SV=1 | TF2H4 | 52 | 0.44 | 0 | 0 | 0 | 0 | 0 | 0 | 0 | 0 | 0 | 85% | 0 | 95% |
| 1998 | THO complex subunit 3 OS=Homo sapiens GN=THOC3 PE=1 SV=1 | THOC3 | 39 | 0.0036 | 0 | 0 | 95% | 0 | 0 | 0 | 0 | 53% | 0 | 95% | 95% | 95% |
| 1999 | Transmembrane emp24 domain-containing protein 3 OS=Homo sapiens GN=TMED3 PE=1 SV=1 | TMED3 | 25 | 0.18 | 12% | 95% | 0 | 0 | 0 | 0 | 95% | 0 | 95% | 0 | 0 | 0 |
| 2000 | Thioredoxin reductase 1, cytoplasmic OS=Homo sapiens GN=TXNRD1 PE=1 SV=3 | TRXR1 | 71 | 0.43 | 0 | 81% | 0 | 0 | 0 | 0 | 95% | 0 | 95% | 32% | 0 | 95% |
| 2001 | Proteasome subunit alpha type-6 OS=Homo sapiens GN=PSMA6 PE=1 SV=1 | PSA6 | 27 | 0.44 | 0 | 0 | 0 | 0 | 8% | 0 | 33% | 19% | 95% | 5% | 0 | 0 |
| 2002 | Sorting nexin-19 OS=Homo sapiens GN=SNX19 PE=1 SV=2 | SNX19 | 109 | 0.44 | 0 | 0 | 7% | 0 | 80% | 0 | 0 | 0 | 0 | 14% | 27% | 93% |
| 2003 | Spliceosome-associated protein CWC15 homolog OS=Homo sapiens GN=CWC15 PE=1 SV=2 | CWC15 | 27 | 0.44 | 0 | 23% | 91% | 0 | 0 | 0 | 0 | 0 | 43% | 18% | 0 | 15% |
| 2004 | Cluster of Proteasome subunit alpha type-4 OS=Homo sapiens GN=PSMA4 PE=1 SV=1 (PSA4_HUMAN) | PSA4 [2] | 29 | < 0.00010 | 0 | 0 | 0 | 0 | 0 | 0 | 100% | 95% | 95% | 0 | 0 | 0 |
| 2005 | Collagen alpha-1(IV) chain OS=Homo sapiens GN=COL4A1 PE=1 SV=3 | CO4A1 (+1) | 161 | 0.0012 | 0 | 0 | 0 | 0 | 0 | 0 | 95% | 100% | 95% | 0 | 0 | 0 |
| 2006 | Muscleblind-like protein 1 OS=Homo sapiens GN=MBNL1 PE=1 SV=2 | MBNL1 | 42 | 0.0012 | 0 | 0 | 0 | 0 | 0 | 0 | 95% | 100% | 95% | 0 | 0 | 0 |
| 2007 | BCL2/adenovirus E1B 19 kDa protein-interacting protein 3 OS=Homo sapiens GN=BNIP3 PE=1 SV=2 | BNIP3 | 22 | 0.18 | 0 | 95% | 0 | 0 | 0 | 0 | 95% | 100% | 0 | 0 | 0 | 0 |
| 2008 | Mitochondrial inner membrane protein OXA1L OS=Homo sapiens GN=OXA1L PE=1 SV=3 | OXA1L | 49 | 0.59 | 0 | 0 | 0 | 100% | 0 | 0 | 0 | 0 | 0 | 95% | 0 | 0 |
| 2009 | Proteasome subunit alpha type-5 OS=Homo sapiens GN=PSMA5 PE=1 SV=3 | PSA5 | 26 | < 0.00010 | 0 | 0 | 0 | 0 | 0 | 0 | 100% | 95% | 95% | 0 | 0 | 0 |
| 2010 | Piwi-like protein 4 OS=Homo sapiens GN=PIWIL4 PE=2 SV=2 | PIWL4 | 97 | 0.44 | 0 | 0 | 0 | 0 | 0 | 0 | 0 | 0 | 0 | 0 | 0 | 100% |
| 2011 | Platelet receptor Gi24 OS=Homo sapiens GN=C10orf54 PE=1 SV=3 | GI24 | 34 | 0.59 | 95% | 0 | 0 | 100% | 43% | 0 | 0 | 0 | 0 | 0 | 0 | 0 |
| 2012 | Golgin subfamily A member 7 OS=Homo sapiens GN=GOLGA7 PE=1 SV=2 | GOGA7 | 16 | 0.19 | 0 | 0 | 0 | 95% | 100% | 0 | 0 | 0 | 0 | 0 | 0 | 95% |
| 2013 | SURP and G-patch domain-containing protein 2 OS=Homo sapiens GN=SUGP2 PE=1 SV=2 | SUGP2 | 120 | 0.44 | 0 | 0 | 0 | 0 | 0 | 0 | 0 | 100% | 0 | 16% | 21% | 0 |
| 2014 | Proteasome subunit alpha type-2 OS=Homo sapiens GN=PSMA2 PE=1 SV=2 | PSA2 | 26 | 0.052 | 0 | 0 | 0 | 0 | 0 | 0 | 95% | 0 | 100% | 0 | 0 | 0 |
| 2015 | Peptidyl-prolyl cis-trans isomerase-like 1 OS=Homo sapiens GN=PPIL1 PE=1 SV=1 | PPIL1 | 18 | 0.24 | 100% | 0 | 0 | 0 | 0 | 0 | 0 | 0 | 0 | 95% | 95% | 0 |
| 2016 | Cell growth-regulating nucleolar protein OS=Homo sapiens GN=LYAR PE=1 SV=2 | LYAR | 44 | 0.052 | 0 | 0 | 0 | 0 | 0 | 0 | 100% | 95% | 0 | 0 | 0 | 0 |
| 2017 | Fatty acid desaturase 1 OS=Homo sapiens GN=FADS1 PE=1 SV=3 | FADS1 | 52 | 0.24 | 0 | 0 | 0 | 0 | 0 | 0 | 0 | 0 | 95% | 100% | 97% | 0 |
| 2018 | Pre-mRNA-splicing factor ATP-dependent RNA helicase PRP16 OS=Homo sapiens GN=DHX38 PE=1 SV=2 | PRP16 | 141 | 0.052 | 0 | 0 | 0 | 0 | 0 | 0 | 0 | 0 | 0 | 99% | 100% | 0 |
| 2019 | Nesprin-3 OS=Homo sapiens GN=SYNE3 PE=1 SV=2 | SYNE3 | 112 | 0.24 | 0 | 0 | 0 | 0 | 0 | 95% | 0 | 0 | 0 | 71% | 0 | 100% |
| 2020 | WD repeat-containing protein 60 OS=Homo sapiens GN=WDR60 PE=1 SV=3 | WDR60 | 123 | 0.44 | 0 | 0 | 6% | 0 | 0 | 100% | 0 | 0 | 0 | 0 | 0 | 0 |
| 2021 | AP-3 complex subunit beta-2 OS=Homo sapiens GN=AP3B2 PE=1 SV=2 | AP3B2 | 119 | 0.44 | 0 | 0 | 0 | 0 | 7% | 0 | 0 | 0 | 0 | 100% | 0 | 0 |
| 2022 | E3 ubiquitin-protein ligase Midline-1 OS=Homo sapiens GN=MID1 PE=1 SV=1 | TRI18 | 75 | 0.052 | 0 | 95% | 100% | 0 | 0 | 0 | 0 | 0 | 0 | 0 | 0 | 0 |
| 2023 | Spermatid perinuclear RNA-binding protein OS=Homo sapiens GN=STRBP PE=1 SV=1 | STRBP | 74 | 0.32 | 27% | 0 | 99% | 0 | 0 | 0 | 0 | 0 | 81% | 0 | 0 | 0 |
| 2024 | Biorientation of chromosomes in cell division protein 1-like 1 OS=Homo sapiens GN=BOD1L1 PE=1 SV=2 | BD1L1 | 330 | 0.44 | 0 | 0 | 0 | 0 | 0 | 0 | 0 | 99% | 0 | 0 | 0 | 0 |
| 2025 | Dedicator of cytokinesis protein 10 OS=Homo sapiens GN=DOCK10 PE=1 SV=3 | DOC10 | 250 | 0.052 | 0 | 0 | 0 | 0 | 0 | 0 | 99% | 0 | 95% | 0 | 0 | 0 |
| 2026 | CMP-N-acetylneuraminate-beta-galactosamide-alpha-2,3-sialyltransferase 4 OS=Homo sapiens GN=ST3GAL4 PE=2 SV=1 | SIA4C | 38 | 0.44 | 99% | 0 | 0 | 0 | 0 | 0 | 0 | 0 | 0 | 0 | 0 | 0 |
| 2027 | Protein Hook homolog 3 OS=Homo sapiens GN=HOOK3 PE=1 SV=2 | HOOK3 | 83 | 0.052 | 0 | 0 | 0 | 0 | 0 | 0 | 30% | 99% | 95% | 0 | 0 | 0 |
| 2028 | Q96BY6-DECOY | Q96BY6-DECOY | ? | 0.53 | 99% | 0 | 95% | 0 | 0 | 95% | 0 | 0 | 0 | 95% | 0 | 0 |
| 2029 | Protein FAM179B OS=Homo sapiens GN=FAM179B PE=1 SV=4 | F179B | 189 | 0.44 | 0 | 0 | 0 | 0 | 0 | 0 | 0 | 0 | 0 | 99% | 13% | 15% |
| 2030 | Bystin OS=Homo sapiens GN=BYSL PE=1 SV=3 | BYST | 50 | 0.6 | 0 | 0 | 0 | 0 | 95% | 0 | 0 | 99% | 56% | 0 | 0 | 0 |
| 2031 | RNA-binding protein 28 OS=Homo sapiens GN=RBM28 PE=1 SV=3 | RBM28 | 86 | 0.44 | 0 | 0 | 0 | 0 | 0 | 0 | 0 | 0 | 95% | 0 | 98% | 0 |
| 2032 | U3 small nucleolar ribonucleoprotein protein MPP10 OS=Homo sapiens GN=MPHOSPH10 PE=1 SV=2 | MPP10 | 79 | 0.44 | 0 | 0 | 0 | 0 | 0 | 0 | 0 | 0 | 0 | 98% | 10% | 0 |
| 2033 | Eukaryotic translation initiation factor 5A-1 OS=Homo sapiens GN=EIF5A PE=1 SV=2 | IF5A1 | 17 | 0.2 | 0 | 0 | 0 | 0 | 0 | 0 | 95% | 0 | 95% | 98% | 0 | 0 |
| 2034 | Ankyrin repeat and SOCS box protein 2 OS=Homo sapiens GN=ASB2 PE=1 SV=1 | ASB2 | 65 | 0.44 | 98% | 38% | 28% | 0 | 0 | 0 | 0 | 0 | 0 | 0 | 0 | 0 |
| 2035 | Pyrroline-5-carboxylate reductase 1, mitochondrial OS=Homo sapiens GN=PYCR1 PE=1 SV=2 | P5CR1 | 33 | 0.6 | 0 | 0 | 0 | 0 | 0 | 0 | 0 | 0 | 97% | 0 | 95% | 0 |
| 2036 | Enoyl-CoA delta isomerase 2, mitochondrial OS=Homo sapiens GN=ECI2 PE=1 SV=4 | ECI2 | 44 | 0.052 | 0 | 0 | 0 | 0 | 0 | 0 | 0 | 0 | 0 | 95% | 97% | 21% |
| 2037 | Serine/threonine-protein kinase LATS2 OS=Homo sapiens GN=LATS2 PE=1 SV=2 | LATS2 | 120 | 0.44 | 96% | 0 | 0 | 0 | 0 | 0 | 0 | 0 | 0 | 0 | 95% | 0 |
| 2038 | Barrier-to-autointegration factor OS=Homo sapiens GN=BANF1 PE=1 SV=1 | BAF | 10 | 0.12 | 95% | 95% | 0 | 95% | 0 | 95% | 0 | 0 | 0 | 0 | 0 | 0 |
| 2039 | Q9NPP4-DECOY | Q9NPP4-DECOY | ? | 0.28 | 0 | 76% | 95% | 0 | 0 | 0 | 0 | 0 | 95% | 0 | 0 | 0 |
| 2040 | AFG3-like protein 2 OS=Homo sapiens GN=AFG3L2 PE=1 SV=2 | AFG32 | 89 | 0.59 | 0 | 0 | 0 | 0 | 16% | 95% | 0 | 0 | 0 | 95% | 0 | 0 |
| 2041 | Armadillo repeat-containing X-linked protein 3 OS=Homo sapiens GN=ARMCX3 PE=1 SV=1 | ARMX3 | 43 | 0.44 | 0 | 40% | 79% | 0 | 0 | 0 | 0 | 0 | 0 | 0 | 0 | 95% |
| 2042 | N(4)-(beta-N-acetylglucosaminyl)-L-asparaginase OS=Homo sapiens GN=AGA PE=1 SV=2 | ASPG | 37 | 0.12 | 0 | 95% | 95% | 0 | 0 | 0 | 0 | 0 | 0 | 95% | 95% | 0 |
| 2043 | Chromobox protein homolog 8 OS=Homo sapiens GN=CBX8 PE=1 SV=3 | CBX8 | 43 | 0.052 | 0 | 0 | 0 | 0 | 0 | 0 | 16% | 0 | 0 | 0 | 95% | 95% |
| 2044 | Cholinephosphotransferase 1 OS=Homo sapiens GN=CHPT1 PE=1 SV=1 | CHPT1 | 45 | < 0.00010 | 0 | 0 | 0 | 0 | 0 | 0 | 95% | 95% | 95% | 0 | 0 | 0 |
| 2045 | Chondroitin sulfate synthase 1 OS=Homo sapiens GN=CHSY1 PE=1 SV=3 | CHSS1 | 92 | 0.44 | 0 | 0 | 0 | 0 | 0 | 0 | 0 | 0 | 0 | 0 | 95% | 0 |
| 2046 | UPF0420 protein C16orf58 OS=Homo sapiens GN=C16orf58 PE=1 SV=2 | CP058 | 51 | 0.44 | 0 | 0 | 95% | 0 | 0 | 0 | 0 | 0 | 0 | 18% | 0 | 0 |
| 2047 | Pre-mRNA-splicing factor CWC22 homolog OS=Homo sapiens GN=CWC22 PE=1 SV=3 | CWC22 | 105 | 0.052 | 0 | 0 | 0 | 0 | 0 | 46% | 0 | 0 | 0 | 95% | 95% | 0 |
| 2048 | Cytochrome c oxidase subunit 6B1 OS=Homo sapiens GN=COX6B1 PE=1 SV=2 | CX6B1 | 10 | 0.097 | 0 | 0 | 0 | 0 | 0 | 0 | 0 | 0 | 0 | 0 | 95% | 95% |
| 2049 | Probable ATP-dependent RNA helicase DDX47 OS=Homo sapiens GN=DDX47 PE=1 SV=1 | DDX47 | 51 | < 0.00010 | 0 | 0 | 0 | 0 | 0 | 12% | 0 | 0 | 0 | 95% | 95% | 95% |
| 2050 | Deoxyribonuclease-2-alpha OS=Homo sapiens GN=DNASE2 PE=1 SV=2 | DNS2A | 40 | 0.59 | 0 | 0 | 81% | 0 | 0 | 0 | 0 | 0 | 0 | 95% | 10% | 14% |
| 2051 | Exonuclease 3'-5' domain-containing protein 2 OS=Homo sapiens GN=EXD2 PE=1 SV=2 | EXD2 | 70 | < 0.00010 | 18% | 0 | 0 | 0 | 0 | 0 | 0 | 0 | 0 | 95% | 95% | 95% |
| 2052 | Exosome complex component MTR3 OS=Homo sapiens GN=EXOSC6 PE=1 SV=1 | EXOS6 | 28 | < 0.00010 | 0 | 0 | 0 | 0 | 0 | 0 | 0 | 63% | 0 | 95% | 95% | 95% |
| 2053 | Exosome complex component RRP42 OS=Homo sapiens GN=EXOSC7 PE=1 SV=3 | EXOS7 | 32 | 0.052 | 0 | 0 | 0 | 0 | 0 | 5% | 0 | 95% | 95% | 0 | 0 | 0 |
| 2054 | Protein FAM8A1 OS=Homo sapiens GN=FAM8A1 PE=1 SV=1 | FA8A1 | 44 | 0.59 | 0 | 0 | 95% | 0 | 0 | 0 | 0 | 0 | 0 | 95% | 17% | 47% |
| 2055 | sp|P11362|FGFR1_HUMAN-DECOY Fibroblast growth factor receptor 1 OS=Homo sapiens GN=FGFR1... | FGFR1-DECOY (+2) | ? | 0.17 | 62% | 95% | 0 | 0 | 0 | 0 | 0 | 95% | 92% | 0 | 0 | 0 |
| 2056 | H/ACA ribonucleoprotein complex subunit 1 OS=Homo sapiens GN=GAR1 PE=1 SV=1 | GAR1 | 22 | 0.59 | 0 | 14% | 95% | 0 | 0 | 0 | 9% | 95% | 0 | 0 | 0 | 0 |
| 2057 | Kinesin-like protein KIF2A OS=Homo sapiens GN=KIF2A PE=1 SV=3 | KIF2A | 80 | 0.44 | 0 | 0 | 0 | 0 | 0 | 0 | 0 | 0 | 82% | 0 | 0 | 95% |
| 2058 | Keratinocyte proline-rich protein OS=Homo sapiens GN=KPRP PE=1 SV=1 | KPRP | 64 | 0.18 | 0 | 95% | 0 | 0 | 0 | 0 | 83% | 13% | 95% | 0 | 0 | 0 |
| 2059 | Leucine-rich repeat and coiled-coil domain-containing protein 1 OS=Homo sapiens GN=LRRCC1 PE=1 SV=2 | LRCC1 | 120 | 0.44 | 0 | 0 | 0 | 0 | 0 | 48% | 7% | 0 | 0 | 95% | 0 | 0 |
| 2060 | Lysophospholipid acyltransferase 2 OS=Homo sapiens GN=MBOAT2 PE=2 SV=2 | MBOA2 | 60 | < 0.00010 | 0 | 0 | 0 | 0 | 0 | 0 | 0 | 0 | 0 | 95% | 95% | 95% |
| 2061 | Mitochondrial carnitine/acylcarnitine carrier protein OS=Homo sapiens GN=SLC25A20 PE=1 SV=1 | MCAT | 33 | 0.12 | 0 | 0 | 0 | 0 | 95% | 95% | 0 | 0 | 0 | 95% | 95% | 0 |
| 2062 | sp|Q9UBU8|MO4L1_HUMAN-DECOY Mortality factor 4-like protein 1 OS=Homo sapiens GN=MORF4L1... | MO4L1-DECOY (+1) | ? | 0.6 | 95% | 0 | 0 | 0 | 0 | 0 | 0 | 7% | 0 | 0 | 95% | 0 |
| 2063 | Nuclear cap-binding protein subunit 1 OS=Homo sapiens GN=NCBP1 PE=1 SV=1 | NCBP1 | 92 | 0.6 | 0 | 0 | 0 | 0 | 0 | 0 | 0 | 0 | 95% | 0 | 95% | 0 |
| 2064 | Neuroguidin OS=Homo sapiens GN=NGDN PE=1 SV=1 | NGDN | 36 | 0.013 | 0 | 0 | 0 | 0 | 95% | 0 | 0 | 0 | 0 | 70% | 95% | 95% |
| 2065 | PAS domain-containing protein 1 OS=Homo sapiens GN=PASD1 PE=2 SV=1 | PASD1 | 87 | 0.44 | 0 | 0 | 0 | 0 | 0 | 0 | 10% | 95% | 0 | 0 | 0 | 0 |
| 2066 | Periphilin-1 OS=Homo sapiens GN=PPHLN1 PE=1 SV=2 | PPHLN | 53 | 0.44 | 0 | 0 | 0 | 0 | 0 | 95% | 0 | 0 | 0 | 12% | 13% | 14% |
| 2067 | Protein phosphatase 1 regulatory subunit 3A OS=Homo sapiens GN=PPP1R3A PE=1 SV=3 | PPR3A | 126 | 0.44 | 0 | 0 | 0 | 0 | 0 | 0 | 0 | 0 | 0 | 95% | 0 | 0 |
| 2068 | Q9BY44-DECOY | Q9BY44-DECOY (+1) | ? | 0.44 | 95% | 0 | 0 | 0 | 0 | 22% | 0 | 20% | 0 | 0 | 0 | 0 |
| 2069 | Putative RNA-binding protein 15 OS=Homo sapiens GN=RBM15 PE=1 SV=2 | RBM15 | 107 | 0.59 | 0 | 0 | 0 | 0 | 95% | 59% | 0 | 0 | 0 | 95% | 0 | 0 |
| 2070 | 60 kDa SS-A/Ro ribonucleoprotein OS=Homo sapiens GN=TROVE2 PE=1 SV=2 | RO60 | 61 | 0.052 | 0 | 0 | 0 | 0 | 0 | 0 | 95% | 0 | 95% | 0 | 0 | 0 |
| 2071 | RRP12-like protein OS=Homo sapiens GN=RRP12 PE=1 SV=2 | RRP12 | 144 | 0.12 | 0 | 0 | 0 | 0 | 0 | 0 | 0 | 95% | 95% | 0 | 95% | 95% |
| 2072 | Protein O-mannose kinase OS=Homo sapiens GN=POMK PE=1 SV=1 | SG196 | 40 | 0.47 | 0 | 0 | 92% | 0 | 0 | 0 | 0 | 95% | 0 | 95% | 0 | 95% |
| 2073 | Tryptophan--tRNA ligase, cytoplasmic OS=Homo sapiens GN=WARS PE=1 SV=2 | SYWC | 53 | 0.12 | 95% | 0 | 95% | 0 | 0 | 0 | 95% | 95% | 0 | 0 | 0 | 0 |
| 2074 | Target of Myb protein 1 OS=Homo sapiens GN=TOM1 PE=1 SV=2 | TOM1 | 54 | 0.46 | 0 | 95% | 0 | 0 | 0 | 0 | 95% | 0 | 0 | 95% | 95% | 0 |
| 2075 | Translocating chain-associated membrane protein 2 OS=Homo sapiens GN=TRAM2 PE=1 SV=1 | TRAM2 | 43 | 0.25 | 0 | 95% | 95% | 0 | 0 | 0 | 0 | 0 | 0 | 95% | 0 | 0 |
| 2076 | Ubiquitin-conjugating enzyme E2 J1 OS=Homo sapiens GN=UBE2J1 PE=1 SV=2 | UB2J1 | 35 | 0.45 | 0 | 0 | 0 | 0 | 95% | 95% | 95% | 0 | 0 | 95% | 0 | 0 |
| 2077 | WD repeat-containing protein 11 OS=Homo sapiens GN=WDR11 PE=1 SV=1 | WDR11 | 137 | 0.6 | 0 | 0 | 0 | 0 | 0 | 9% | 0 | 95% | 0 | 0 | 0 | 95% |
| 2078 | Uncharacterized protein C18orf63 OS=Homo sapiens GN=C18orf63 PE=2 SV=2 | CR063 | 77 | 0.44 | 0 | 0 | 0 | 0 | 0 | 0 | 0 | 0 | 0 | 0 | 94% | 0 |
| 2079 | Q9Y490-DECOY | Q9Y490-DECOY | ? | 0.44 | 0 | 0 | 45% | 0 | 88% | 0 | 0 | 0 | 0 | 0 | 0 | 0 |
| 2080 | Cleavage stimulation factor subunit 1 OS=Homo sapiens GN=CSTF1 PE=1 SV=1 | CSTF1 | 48 | 0.093 | 0 | 0 | 0 | 0 | 0 | 0 | 0 | 0 | 0 | 0 | 100% | 95% |
| 2081 | 26S protease regulatory subunit 7 OS=Homo sapiens GN=PSMC2 PE=1 SV=3 | PRS7 | 49 | 0.44 | 0 | 0 | 0 | 0 | 0 | 0 | 100% | 0 | 0 | 0 | 0 | 0 |
| 2082 | Glycoprotein-N-acetylgalactosamine 3-beta-galactosyltransferase 1 OS=Homo sapiens GN=C1GALT1 PE=1 SV=1 | C1GLT | 42 | 0.57 | 0 | 0 | 0 | 0 | 95% | 0 | 0 | 0 | 0 | 0 | 0 | 100% |
| 2083 | RNA-binding protein 8A OS=Homo sapiens GN=RBM8A PE=1 SV=1 | RBM8A | 20 | 0.44 | 0 | 0 | 0 | 0 | 0 | 0 | 0 | 0 | 0 | 100% | 0 | 6% |
| 2084 | Cytoplasmic aconitate hydratase OS=Homo sapiens GN=ACO1 PE=1 SV=3 | ACOC | 98 | 0.44 | 0 | 0 | 0 | 0 | 0 | 0 | 100% | 0 | 0 | 0 | 0 | 0 |
| 2085 | OCIA domain-containing protein 1 OS=Homo sapiens GN=OCIAD1 PE=1 SV=1 | OCAD1 | 28 | 0.44 | 0 | 0 | 0 | 0 | 0 | 0 | 0 | 0 | 0 | 0 | 0 | 100% |
| 2086 | U2 small nuclear ribonucleoprotein B'' OS=Homo sapiens GN=SNRPB2 PE=1 SV=1 | RU2B | 25 | 0.44 | 0 | 97% | 0 | 0 | 0 | 0 | 0 | 0 | 0 | 100% | 0 | 0 |
| 2087 | TBC1 domain family member 8B OS=Homo sapiens GN=TBC1D8B PE=1 SV=2 | TBC8B | 129 | 0.44 | 0 | 0 | 0 | 0 | 0 | 0 | 100% | 0 | 0 | 0 | 0 | 0 |
| 2088 | Eukaryotic translation initiation factor 3 subunit K OS=Homo sapiens GN=EIF3K PE=1 SV=1 | EIF3K | 25 | 0.6 | 0 | 0 | 0 | 0 | 0 | 0 | 100% | 0 | 0 | 0 | 95% | 0 |
| 2089 | Beta-1,3-glucosyltransferase OS=Homo sapiens GN=B3GALTL PE=1 SV=2 | B3GLT | 57 | < 0.00010 | 0 | 0 | 0 | 100% | 95% | 95% | 0 | 0 | 0 | 0 | 0 | 0 |
| 2090 | DNA polymerase zeta catalytic subunit OS=Homo sapiens GN=REV3L PE=1 SV=2 | DPOLZ | 353 | 0.44 | 100% | 0 | 7% | 0 | 0 | 0 | 0 | 0 | 0 | 0 | 0 | 0 |
| 2091 | Estradiol 17-beta-dehydrogenase 11 OS=Homo sapiens GN=HSD17B11 PE=1 SV=3 | DHB11 | 33 | 0.44 | 0 | 95% | 100% | 0 | 0 | 0 | 0 | 0 | 0 | 0 | 0 | 0 |
| 2092 | Solute carrier family 43 member 3 OS=Homo sapiens GN=SLC43A3 PE=1 SV=2 | S43A3 | 55 | 0.44 | 0 | 0 | 0 | 0 | 12% | 100% | 0 | 0 | 0 | 0 | 0 | 0 |
| 2093 | Oxidoreductase HTATIP2 OS=Homo sapiens GN=HTATIP2 PE=1 SV=2 | HTAI2 | 27 | 0.6 | 0 | 0 | 0 | 0 | 95% | 0 | 0 | 0 | 0 | 0 | 0 | 99% |
| 2094 | Uncharacterized protein C9orf84 OS=Homo sapiens GN=C9orf84 PE=2 SV=1 | CI084 | 165 | 0.44 | 0 | 11% | 0 | 0 | 99% | 0 | 0 | 0 | 0 | 0 | 0 | 0 |
| 2095 | Mitochondrial dicarboxylate carrier OS=Homo sapiens GN=SLC25A10 PE=1 SV=2 | DIC | 31 | 0.6 | 0 | 0 | 0 | 0 | 0 | 98% | 0 | 0 | 0 | 0 | 0 | 95% |
| 2096 | Notchless protein homolog 1 OS=Homo sapiens GN=NLE1 PE=1 SV=4 | NLE1 | 53 | 0.44 | 0 | 0 | 0 | 0 | 0 | 0 | 0 | 98% | 0 | 0 | 0 | 0 |
| 2097 | 39S ribosomal protein L28, mitochondrial OS=Homo sapiens GN=MRPL28 PE=1 SV=4 | RM28 | 30 | 0.052 | 0 | 0 | 0 | 0 | 0 | 0 | 0 | 0 | 0 | 98% | 0 | 95% |
| 2098 | PDZ domain-containing protein 8 OS=Homo sapiens GN=PDZD8 PE=1 SV=1 | PDZD8 | 129 | 0.055 | 0 | 0 | 0 | 95% | 95% | 0 | 0 | 0 | 0 | 0 | 0 | 0 |
| 2099 | Uncharacterized protein C2orf73 OS=Homo sapiens GN=C2orf73 PE=2 SV=3 | CB073 | 32 | 0.44 | 0 | 0 | 0 | 0 | 0 | 95% | 0 | 0 | 0 | 0 | 0 | 0 |
| 2100 | Cytochrome c-type heme lyase OS=Homo sapiens GN=HCCS PE=1 SV=1 | CCHL | 31 | 0.056 | 95% | 0 | 95% | 0 | 0 | 0 | 0 | 0 | 0 | 32% | 0 | 0 |
| 2101 | Hsp90 co-chaperone Cdc37 OS=Homo sapiens GN=CDC37 PE=1 SV=1 | CDC37 | 44 | 0.44 | 0 | 95% | 0 | 0 | 0 | 0 | 0 | 0 | 0 | 31% | 0 | 0 |
| 2102 | Cholesterol 24-hydroxylase OS=Homo sapiens GN=CYP46A1 PE=1 SV=1 | CP46A | 57 | 0.44 | 0 | 13% | 0 | 0 | 0 | 0 | 0 | 6% | 0 | 0 | 95% | 0 |
| 2103 | Copine-8 OS=Homo sapiens GN=CPNE8 PE=1 SV=2 | CPNE8 | 63 | 0.44 | 0 | 0 | 0 | 0 | 0 | 95% | 0 | 0 | 0 | 12% | 0 | 14% |
| 2104 | Crooked neck-like protein 1 OS=Homo sapiens GN=CRNKL1 PE=1 SV=4 | CRNL1 | 100 | 0.052 | 0 | 95% | 95% | 0 | 0 | 0 | 0 | 0 | 0 | 0 | 0 | 0 |
| 2105 | Carboxy-terminal domain RNA polymerase II polypeptide A small phosphatase 1 OS=Homo sapiens GN=CTDSP1 PE=1 SV=1 | CTDS1 | 29 | < 0.00010 | 95% | 95% | 95% | 0 | 0 | 0 | 0 | 0 | 0 | 0 | 0 | 0 |
| 2106 | sp|P39880|CUX1_HUMAN-DECOY Homeobox protein cut-like 1 OS=Homo sapiens GN=CUX1 PE=1... | CUX1-DECOY | ? | 0.8 | 0 | 0 | 0 | 0 | 95% | 0 | 0 | 95% | 0 | 93% | 0 | 0 |
| 2107 | Cystatin-C OS=Homo sapiens GN=CST3 PE=1 SV=1 | CYTC | 16 | 0.59 | 0 | 0 | 0 | 0 | 0 | 95% | 0 | 0 | 0 | 95% | 0 | 0 |
| 2108 | Probable ATP-dependent RNA helicase DDX52 OS=Homo sapiens GN=DDX52 PE=1 SV=3 | DDX52 | 68 | 0.052 | 0 | 0 | 0 | 0 | 0 | 0 | 0 | 0 | 72% | 0 | 95% | 95% |
| 2109 | Probable ATP-dependent RNA helicase DDX56 OS=Homo sapiens GN=DDX56 PE=1 SV=1 | DDX56 | 62 | 0.6 | 0 | 0 | 0 | 0 | 0 | 0 | 0 | 0 | 95% | 0 | 95% | 7% |
| 2110 | ATP-dependent RNA helicase DHX8 OS=Homo sapiens GN=DHX8 PE=1 SV=1 | DHX8 | 139 | 0.44 | 0 | 0 | 0 | 0 | 0 | 0 | 0 | 0 | 0 | 13% | 95% | 0 |
| 2111 | DnaJ homolog subfamily B member 6 OS=Homo sapiens GN=DNAJB6 PE=1 SV=2 | DNJB6 | 36 | 0.22 | 0 | 0 | 0 | 0 | 95% | 95% | 0 | 0 | 95% | 0 | 0 | 0 |
| 2112 | Delta(3,5)-Delta(2,4)-dienoyl-CoA isomerase, mitochondrial OS=Homo sapiens GN=ECH1 PE=1 SV=2 | ECH1 | 36 | 0.44 | 0 | 0 | 12% | 0 | 0 | 0 | 0 | 0 | 0 | 95% | 0 | 0 |
| 2113 | ER membrane protein complex subunit 6 OS=Homo sapiens GN=EMC6 PE=1 SV=1 | EMC6 | 12 | 0.052 | 0 | 0 | 0 | 0 | 0 | 0 | 95% | 95% | 0 | 0 | 0 | 5% |
| 2114 | ELM2 and SANT domain-containing protein 1 OS=Homo sapiens GN=ELMSAN1 PE=1 SV=2 | EMSA1 | 115 | 0.44 | 0 | 0 | 0 | 0 | 0 | 0 | 0 | 0 | 0 | 0 | 0 | 95% |
| 2115 | Ectopic P granules protein 5 homolog OS=Homo sapiens GN=EPG5 PE=2 SV=2 | EPG5 | 292 | 0.6 | 95% | 0 | 6% | 0 | 0 | 0 | 0 | 0 | 0 | 0 | 95% | 0 |
| 2116 | Exosome complex component RRP45 OS=Homo sapiens GN=EXOSC9 PE=1 SV=3 | EXOS9 | 49 | 0.6 | 0 | 0 | 0 | 0 | 0 | 95% | 95% | 60% | 0 | 0 | 0 | 0 |
| 2117 | HIRA-interacting protein 3 OS=Homo sapiens GN=HIRIP3 PE=1 SV=3 | HIRP3 | 62 | 0.44 | 95% | 0 | 0 | 0 | 0 | 0 | 0 | 0 | 0 | 0 | 0 | 0 |
| 2118 | Eukaryotic translation initiation factor 1A, X-chromosomal OS=Homo sapiens GN=EIF1AX PE=1 SV=2 | IF1AX (+1) | 16 | 0.052 | 0 | 0 | 0 | 0 | 0 | 0 | 0 | 0 | 0 | 95% | 95% | 0 |
| 2119 | Pre-mRNA-splicing factor ISY1 homolog OS=Homo sapiens GN=ISY1 PE=1 SV=3 | ISY1 | 33 | 0.59 | 0 | 0 | 0 | 12% | 0 | 95% | 0 | 0 | 0 | 91% | 0 | 0 |
| 2120 | Keratinocyte-associated transmembrane protein 2 OS=Homo sapiens GN=KCT2 PE=2 SV=2 | KCT2 | 29 | 0.6 | 0 | 0 | 0 | 0 | 95% | 0 | 95% | 0 | 0 | 0 | 0 | 0 |
| 2121 | Luc7-like protein 3 OS=Homo sapiens GN=LUC7L3 PE=1 SV=2 | LC7L3 | 51 | 0.24 | 0 | 0 | 0 | 0 | 0 | 0 | 0 | 95% | 0 | 95% | 95% | 0 |
| 2122 | U6 snRNA-associated Sm-like protein LSm4 OS=Homo sapiens GN=LSM4 PE=1 SV=1 | LSM4 | 15 | 0.6 | 0 | 0 | 0 | 0 | 0 | 95% | 0 | 0 | 13% | 0 | 95% | 0 |
| 2123 | MANSC domain-containing protein 1 OS=Homo sapiens GN=MANSC1 PE=2 SV=1 | MANS1 | 47 | 0.052 | 0 | 0 | 0 | 0 | 0 | 8% | 0 | 0 | 0 | 95% | 95% | 0 |
| 2124 | DBH-like monooxygenase protein 1 OS=Homo sapiens GN=MOXD1 PE=2 SV=1 | MOXD1 | 70 | 0.052 | 13% | 95% | 95% | 0 | 0 | 0 | 0 | 0 | 0 | 0 | 0 | 0 |
| 2125 | Mitochondrial pyruvate carrier 2 OS=Homo sapiens GN=MPC2 PE=1 SV=1 | MPC2 | 14 | 0.052 | 0 | 0 | 0 | 0 | 0 | 0 | 0 | 0 | 0 | 95% | 58% | 95% |
| 2126 | NADH dehydrogenase [ubiquinone] 1 alpha subcomplex subunit 6 OS=Homo sapiens GN=NDUFA6 PE=1 SV=3 | NDUA6 | 18 | 0.44 | 0 | 0 | 0 | 0 | 0 | 0 | 0 | 0 | 0 | 0 | 29% | 95% |
| 2127 | H/ACA ribonucleoprotein complex subunit 2 OS=Homo sapiens GN=NHP2 PE=1 SV=1 | NHP2 | 17 | 0.21 | 0 | 0 | 0 | 0 | 0 | 0 | 95% | 95% | 0 | 0 | 0 | 95% |
| 2128 | Poly [ADP-ribose] polymerase 9 OS=Homo sapiens GN=PARP9 PE=1 SV=2 | PARP9 | 96 | 0.44 | 0 | 0 | 0 | 0 | 0 | 0 | 0 | 0 | 0 | 0 | 95% | 0 |
| 2129 | PDZ and LIM domain protein 4 OS=Homo sapiens GN=PDLIM4 PE=1 SV=2 | PDLI4 | 35 | 0.59 | 0 | 95% | 0 | 0 | 0 | 0 | 0 | 95% | 0 | 0 | 0 | 0 |
| 2130 | Prostaglandin E synthase 2 OS=Homo sapiens GN=PTGES2 PE=1 SV=1 | PGES2 | 42 | 0.052 | 0 | 95% | 95% | 0 | 0 | 0 | 0 | 0 | 0 | 0 | 0 | 0 |
| 2131 | PC4 and SFRS1-interacting protein OS=Homo sapiens GN=PSIP1 PE=1 SV=1 | PSIP1 | 60 | 0.59 | 0 | 0 | 95% | 0 | 95% | 0 | 0 | 0 | 0 | 0 | 0 | 0 |
| 2132 | 26S proteasome non-ATPase regulatory subunit 3 OS=Homo sapiens GN=PSMD3 PE=1 SV=2 | PSMD3 | 61 | 0.44 | 0 | 95% | 0 | 0 | 0 | 0 | 0 | 0 | 0 | 0 | 0 | 0 |
| 2133 | Q15319-DECOY | Q15319-DECOY | ? | < 0.00010 | 0 | 0 | 0 | 82% | 95% | 95% | 0 | 0 | 0 | 0 | 0 | 0 |
| 2134 | Q5UIP0-DECOY | Q5UIP0-DECOY | ? | 0.6 | 0 | 0 | 0 | 0 | 12% | 95% | 0 | 0 | 0 | 0 | 95% | 0 |
| 2135 | Ankycorbin OS=Homo sapiens GN=RAI14 PE=1 SV=2 | RAI14 | 110 | < 0.00010 | 0 | 0 | 0 | 0 | 0 | 0 | 0 | 0 | 0 | 95% | 95% | 95% |
| 2136 | Receptor expression-enhancing protein 4 OS=Homo sapiens GN=REEP4 PE=1 SV=1 | REEP4 | 29 | 0.096 | 0 | 0 | 0 | 0 | 0 | 0 | 0 | 95% | 95% | 0 | 0 | 0 |
| 2137 | Replication factor C subunit 2 OS=Homo sapiens GN=RFC2 PE=1 SV=3 | RFC2 | 39 | 0.44 | 0 | 0 | 0 | 0 | 0 | 0 | 0 | 0 | 0 | 95% | 0 | 0 |
| 2138 | E3 ubiquitin-protein ligase RNF220 OS=Homo sapiens GN=RNF220 PE=1 SV=1 | RN220 | 63 | 0.44 | 0 | 0 | 0 | 0 | 0 | 0 | 95% | 0 | 0 | 0 | 0 | 0 |
| 2139 | Solute carrier family 35 member C2 OS=Homo sapiens GN=SLC35C2 PE=1 SV=2 | S35C2 | 40 | 0.59 | 0 | 95% | 13% | 0 | 95% | 0 | 0 | 0 | 0 | 0 | 0 | 0 |
| 2140 | CMP-N-acetylneuraminate-beta-galactosamide-alpha-2,3-sialyltransferase 1 OS=Homo sapiens GN=ST3GAL1 PE=2 SV=1 | SIA4A | 39 | < 0.00010 | 0 | 0 | 0 | 0 | 0 | 0 | 95% | 95% | 95% | 0 | 0 | 0 |
| 2141 | Spermatogenesis-associated protein 1 OS=Homo sapiens GN=SPATA1 PE=2 SV=3 | SPAT1 | 50 | 0.44 | 95% | 0 | 0 | 11% | 0 | 0 | 0 | 0 | 0 | 0 | 0 | 0 |
| 2142 | FACT complex subunit SSRP1 OS=Homo sapiens GN=SSRP1 PE=1 SV=1 | SSRP1 | 81 | 0.59 | 0 | 95% | 5% | 0 | 95% | 0 | 0 | 0 | 0 | 0 | 0 | 0 |
| 2143 | Three-prime repair exonuclease 1 OS=Homo sapiens GN=TREX1 PE=1 SV=1 | TREX1 | 39 | 0.44 | 0 | 0 | 0 | 0 | 0 | 0 | 0 | 0 | 0 | 58% | 95% | 51% |
| 2144 | Ubiquitin-conjugating enzyme E2 N OS=Homo sapiens GN=UBE2N PE=1 SV=1 | UBE2N (+1) | 17 | 0.59 | 0 | 0 | 95% | 0 | 95% | 0 | 18% | 0 | 0 | 0 | 0 | 0 |
| 2145 | Vacuolar protein sorting-associated protein 18 homolog OS=Homo sapiens GN=VPS18 PE=1 SV=2 | VPS18 | 110 | 0.44 | 0 | 0 | 0 | 19% | 0 | 0 | 95% | 0 | 0 | 0 | 0 | 0 |
| 2146 | Protein PML OS=Homo sapiens GN=PML PE=1 SV=3 | PML | 98 | 0.24 | 0 | 0 | 0 | 0 | 0 | 0 | 0 | 0 | 95% | 0 | 81% | 75% |
| 2147 | Q6PIF2-DECOY | Q6PIF2-DECOY | ? | 0.44 | 0 | 95% | 0 | 0 | 0 | 51% | 0 | 0 | 0 | 0 | 0 | 0 |
| 2148 | sp|Q8WXH0|SYNE2_HUMAN-DECOY Nesprin-2 OS=Homo sapiens GN=SYNE2 PE=1 SV=3 | SYNE2-DECOY | ? | 0.8 | 0 | 0 | 81% | 0 | 0 | 94% | 0 | 0 | 0 | 0 | 84% | 0 |
| 2149 | Programmed cell death protein 6 OS=Homo sapiens GN=PDCD6 PE=1 SV=1 | PDCD6 | 22 | 0.44 | 0 | 0 | 0 | 0 | 0 | 0 | 0 | 0 | 0 | 92% | 0 | 0 |
| 2150 | P15313-DECOY | P15313-DECOY | ? | 0.44 | 0 | 0 | 0 | 0 | 0 | 91% | 0 | 0 | 0 | 0 | 0 | 25% |
| 2151 | Methyltransferase-like protein 17, mitochondrial OS=Homo sapiens GN=METTL17 PE=1 SV=1 | MET17 | 51 | 0.44 | 0 | 0 | 0 | 0 | 0 | 0 | 0 | 0 | 0 | 0 | 0 | 90% |
| 2152 | PAX3- and PAX7-binding protein 1 OS=Homo sapiens GN=PAXBP1 PE=1 SV=2 | PAXB1 | 105 | 0.44 | 0 | 0 | 0 | 0 | 0 | 0 | 0 | 0 | 0 | 100% | 0 | 0 |
| 2153 | DNA replication licensing factor MCM5 OS=Homo sapiens GN=MCM5 PE=1 SV=5 | MCM5 | 82 | 0.44 | 0 | 0 | 0 | 0 | 0 | 0 | 100% | 0 | 0 | 0 | 0 | 0 |
| 2154 | 26S proteasome non-ATPase regulatory subunit 14 OS=Homo sapiens GN=PSMD14 PE=1 SV=1 | PSDE | 35 | 0.44 | 0 | 0 | 0 | 0 | 0 | 0 | 100% | 0 | 0 | 0 | 0 | 0 |
| 2155 | Centrosomal protein of 104 kDa OS=Homo sapiens GN=CEP104 PE=1 SV=1 | CE104 | 104 | 0.44 | 0 | 0 | 0 | 0 | 0 | 0 | 0 | 0 | 0 | 0 | 0 | 100% |
| 2156 | Collagen alpha-5(VI) chain OS=Homo sapiens GN=COL6A5 PE=1 SV=1 | CO6A5 | 290 | 0.44 | 0 | 0 | 0 | 15% | 100% | 0 | 0 | 0 | 0 | 0 | 0 | 0 |
| 2157 | Replication factor C subunit 4 OS=Homo sapiens GN=RFC4 PE=1 SV=2 | RFC4 | 40 | 0.44 | 0 | 0 | 0 | 0 | 0 | 0 | 0 | 0 | 0 | 0 | 0 | 100% |
| 2158 | ATM interactor OS=Homo sapiens GN=ATMIN PE=1 SV=2 | ATMIN | 88 | 0.44 | 0 | 0 | 0 | 0 | 0 | 0 | 0 | 0 | 0 | 0 | 100% | 0 |
| 2159 | Nuclease EXOG, mitochondrial OS=Homo sapiens GN=EXOG PE=1 SV=2 | EXOG | 41 | 0.44 | 0 | 0 | 0 | 0 | 0 | 100% | 0 | 0 | 0 | 0 | 0 | 0 |
| 2160 | ADM2 OS=Homo sapiens GN=ADM2 PE=2 SV=1 | ADM2 | 16 | 0.44 | 0 | 0 | 0 | 0 | 99% | 0 | 0 | 0 | 0 | 0 | 0 | 0 |
| 2161 | Protein FAM49B OS=Homo sapiens GN=FAM49B PE=1 SV=1 | FA49B | 37 | 0.44 | 0 | 0 | 98% | 0 | 0 | 0 | 0 | 0 | 0 | 0 | 0 | 0 |
| 2162 | Heterogeneous nuclear ribonucleoprotein L-like OS=Homo sapiens GN=HNRNPLL PE=1 SV=1 | HNRLL | 60 | 0.44 | 0 | 0 | 0 | 0 | 97% | 0 | 0 | 0 | 0 | 0 | 0 | 0 |
| 2163 | Protein FAM177A1 OS=Homo sapiens GN=FAM177A1 PE=1 SV=1 | F177A | 24 | 0.44 | 0 | 0 | 0 | 0 | 0 | 0 | 0 | 97% | 0 | 0 | 0 | 0 |
| 2164 | Dipeptidyl peptidase 1 OS=Homo sapiens GN=CTSC PE=1 SV=2 | CATC | 52 | 0.052 | 0 | 0 | 0 | 0 | 0 | 0 | 0 | 0 | 0 | 96% | 95% | 0 |
| 2165 | Q8TAG9-DECOY | Q8TAG9-DECOY | ? | 0.44 | 0 | 0 | 0 | 0 | 0 | 0 | 0 | 0 | 0 | 0 | 0 | 96% |
| 2166 | ADP-ribosylation factor GTPase-activating protein 1 OS=Homo sapiens GN=ARFGAP1 PE=1 SV=2 | ARFG1 | 45 | 0.055 | 0 | 0 | 0 | 95% | 0 | 95% | 0 | 0 | 0 | 0 | 0 | 0 |
| 2167 | Protein lifeguard 3 OS=Homo sapiens GN=TMBIM1 PE=1 SV=2 | LFG3 | 35 | 0.055 | 0 | 0 | 0 | 95% | 95% | 0 | 0 | 0 | 0 | 0 | 0 | 0 |
| 2168 | Melanoma-associated antigen B2 OS=Homo sapiens GN=MAGEB2 PE=1 SV=3 | MAGB2 | 35 | 0.055 | 0 | 0 | 0 | 95% | 95% | 0 | 0 | 0 | 0 | 0 | 0 | 0 |
| 2169 | Metaxin-2 OS=Homo sapiens GN=MTX2 PE=1 SV=1 | MTX2 | 30 | 0.055 | 0 | 0 | 0 | 95% | 0 | 95% | 0 | 0 | 0 | 0 | 0 | 0 |
| 2170 | Zinc finger protein 806 OS=Homo sapiens GN=ZNF806 PE=2 SV=1 | ZN806 | 68 | 0.44 | 0 | 0 | 0 | 95% | 0 | 0 | 0 | 0 | 0 | 0 | 0 | 0 |
| 2171 | 6-phosphogluconolactonase OS=Homo sapiens GN=PGLS PE=1 SV=2 | 6PGL | 28 | 0.59 | 0 | 95% | 0 | 0 | 0 | 0 | 0 | 0 | 0 | 0 | 0 | 70% |
| 2172 | sp|P02768|ALBU_HUMAN-DECOY Serum albumin OS=Homo sapiens GN=ALB PE=1 SV=2 | ALBU-DECOY | ? | 0.6 | 0 | 0 | 0 | 0 | 95% | 0 | 72% | 0 | 0 | 0 | 0 | 0 |
| 2173 | Dol-P-Man:Man(7)GlcNAc(2)-PP-Dol alpha-1,6-mannosyltransferase OS=Homo sapiens GN=ALG12 PE=1 SV=1 | ALG12 | 55 | 0.44 | 0 | 0 | 0 | 0 | 0 | 0 | 0 | 0 | 0 | 95% | 0 | 54% |
| 2174 | Alpha-1,2-mannosyltransferase ALG9 OS=Homo sapiens GN=ALG9 PE=1 SV=2 | ALG9 | 70 | 0.59 | 0 | 95% | 0 | 0 | 95% | 0 | 0 | 0 | 0 | 0 | 0 | 0 |
| 2175 | Ankyrin repeat and LEM domain-containing protein 2 OS=Homo sapiens GN=ANKLE2 PE=1 SV=4 | ANKL2 | 104 | 0.44 | 0 | 95% | 0 | 0 | 0 | 0 | 0 | 0 | 0 | 0 | 0 | 0 |
| 2176 | BET1-like protein OS=Homo sapiens GN=BET1L PE=1 SV=1 | BET1L | 12 | 0.59 | 0 | 0 | 95% | 0 | 0 | 0 | 0 | 95% | 0 | 0 | 0 | 0 |
| 2177 | BET1 homolog OS=Homo sapiens GN=BET1 PE=1 SV=1 | BET1 | 13 | 0.44 | 0 | 95% | 0 | 0 | 0 | 0 | 0 | 0 | 0 | 0 | 0 | 52% |
| 2178 | C2 domain-containing protein 2-like OS=Homo sapiens GN=C2CD2L PE=1 SV=3 | C2C2L | 76 | 0.052 | 0 | 0 | 0 | 0 | 95% | 78% | 0 | 0 | 0 | 0 | 0 | 0 |
| 2179 | Carboxypeptidase Z OS=Homo sapiens GN=CPZ PE=1 SV=2 | CBPZ | 74 | 0.052 | 0 | 0 | 0 | 0 | 0 | 0 | 0 | 0 | 0 | 95% | 95% | 0 |
| 2180 | Coiled-coil domain-containing protein 126 OS=Homo sapiens GN=CCDC126 PE=2 SV=2 | CC126 | 16 | 0.44 | 0 | 0 | 0 | 0 | 0 | 18% | 0 | 0 | 0 | 0 | 95% | 0 |
| 2181 | Vacuolar fusion protein CCZ1 homolog B OS=Homo sapiens GN=CCZ1B PE=1 SV=1 | CCZ1B (+1) | 56 | 0.59 | 0 | 95% | 0 | 0 | 0 | 0 | 0 | 0 | 0 | 0 | 0 | 95% |
| 2182 | sp|Q08722|CD47_HUMAN-DECOY Leukocyte surface antigen CD47 OS=Homo sapiens GN=CD47... | CD47-DECOY | ? | 0.055 | 0 | 0 | 0 | 88% | 95% | 0 | 0 | 0 | 0 | 0 | 0 | 0 |
| 2183 | UPF0669 protein C6orf120 OS=Homo sapiens GN=C6orf120 PE=1 SV=1 | CF120 | 21 | 0.44 | 0 | 0 | 95% | 0 | 0 | 0 | 0 | 0 | 0 | 0 | 0 | 0 |
| 2184 | Choline/ethanolamine kinase OS=Homo sapiens GN=CHKB PE=1 SV=3 | CHKB | 45 | 0.44 | 0 | 0 | 0 | 0 | 0 | 0 | 0 | 0 | 0 | 0 | 95% | 0 |
| 2185 | CDGSH iron-sulfur domain-containing protein 1 OS=Homo sapiens GN=CISD1 PE=1 SV=1 | CISD1 | 12 | 0.44 | 0 | 0 | 0 | 0 | 0 | 95% | 0 | 0 | 0 | 0 | 0 | 0 |
| 2186 | Uncharacterized protein C17orf85 OS=Homo sapiens GN=C17orf85 PE=1 SV=2 | CQ085 | 71 | 0.44 | 0 | 0 | 0 | 0 | 0 | 0 | 0 | 0 | 0 | 0 | 95% | 0 |
| 2187 | Protein FAM111A OS=Homo sapiens GN=FAM111A PE=1 SV=2 | F111A | 70 | 0.44 | 0 | 0 | 0 | 0 | 0 | 0 | 0 | 0 | 0 | 0 | 95% | 0 |
| 2188 | Ferritin light chain OS=Homo sapiens GN=FTL PE=1 SV=2 | FRIL | 20 | 0.59 | 0 | 95% | 0 | 0 | 0 | 0 | 0 | 0 | 0 | 0 | 95% | 0 |
| 2189 | G kinase-anchoring protein 1 OS=Homo sapiens GN=GKAP1 PE=1 SV=2 | GKAP1 | 42 | 0.44 | 0 | 0 | 0 | 0 | 0 | 0 | 0 | 95% | 0 | 0 | 0 | 0 |
| 2190 | GTP-binding protein 1 OS=Homo sapiens GN=GTPBP1 PE=1 SV=3 | GTPB1 | 72 | 0.6 | 0 | 0 | 0 | 0 | 95% | 0 | 0 | 95% | 0 | 0 | 0 | 0 |
| 2191 | Hydroxyacyl-coenzyme A dehydrogenase, mitochondrial OS=Homo sapiens GN=HADH PE=1 SV=3 | HCDH | 34 | 0.44 | 0 | 95% | 0 | 0 | 0 | 0 | 0 | 0 | 0 | 0 | 0 | 75% |
| 2192 | Putative high mobility group protein B1-like 1 OS=Homo sapiens GN=HMGB1P1 PE=5 SV=1 | HGB1A | 24 | 0.44 | 0 | 0 | 0 | 0 | 0 | 0 | 0 | 0 | 0 | 0 | 0 | 95% |
| 2193 | High mobility group protein B2 OS=Homo sapiens GN=HMGB2 PE=1 SV=2 | HMGB2 | 24 | 0.052 | 0 | 0 | 0 | 0 | 0 | 0 | 0 | 0 | 0 | 95% | 95% | 0 |
| 2194 | Intraflagellar transport protein 81 homolog OS=Homo sapiens GN=IFT81 PE=1 SV=1 | IFT81 | 80 | 0.44 | 0 | 0 | 0 | 0 | 95% | 0 | 7% | 0 | 0 | 0 | 0 | 0 |
| 2195 | Inorganic pyrophosphatase OS=Homo sapiens GN=PPA1 PE=1 SV=2 | IPYR | 33 | 0.44 | 0 | 95% | 0 | 0 | 0 | 0 | 0 | 0 | 0 | 0 | 0 | 0 |
| 2196 | Kelch-like protein 14 OS=Homo sapiens GN=KLHL14 PE=1 SV=2 | KLH14 | 71 | 0.44 | 0 | 0 | 0 | 0 | 95% | 0 | 0 | 0 | 0 | 0 | 0 | 0 |
| 2197 | Laminin subunit alpha-4 OS=Homo sapiens GN=LAMA4 PE=1 SV=4 | LAMA4 | 203 | 0.44 | 0 | 0 | 95% | 0 | 0 | 0 | 0 | 0 | 0 | 0 | 0 | 0 |
| 2198 | sp|P55268|LAMB2_HUMAN-DECOY Laminin subunit beta-2 OS=Homo sapiens GN=LAMB2 PE=1... | LAMB2-DECOY | ? | 0.44 | 0 | 0 | 95% | 0 | 0 | 0 | 0 | 0 | 30% | 0 | 0 | 0 |
| 2199 | La-related protein 4 OS=Homo sapiens GN=LARP4 PE=1 SV=3 | LARP4 | 81 | 0.44 | 0 | 0 | 0 | 0 | 0 | 95% | 0 | 0 | 0 | 0 | 0 | 0 |
| 2200 | E3 ubiquitin-protein ligase MARCH5 OS=Homo sapiens GN=MARCH5 PE=1 SV=1 | MARH5 | 31 | 0.44 | 0 | 0 | 95% | 0 | 0 | 0 | 0 | 0 | 0 | 0 | 0 | 0 |
| 2201 | NADH dehydrogenase [ubiquinone] 1 alpha subcomplex subunit 4 OS=Homo sapiens GN=NDUFA4 PE=1 SV=1 | NDUA4 | 9 | 0.44 | 0 | 0 | 0 | 0 | 95% | 0 | 0 | 0 | 0 | 35% | 0 | 0 |
| 2202 | Lipoamide acyltransferase component of branched-chain alpha-keto acid dehydrogenase complex, mitochondrial OS=Homo sapiens GN=DBT PE=1 SV=3 | ODB2 | 53 | 0.44 | 0 | 0 | 0 | 0 | 0 | 0 | 0 | 0 | 0 | 0 | 95% | 13% |
| 2203 | P62750-DECOY | P62750-DECOY | ? | 0.44 | 0 | 0 | 0 | 0 | 0 | 0 | 0 | 0 | 0 | 95% | 0 | 0 |
| 2204 | sp|Q15154|PCM1_HUMAN-DECOY Pericentriolar material 1 protein OS=Homo sapiens GN=PCM1... | PCM1-DECOY | ? | 0.052 | 0 | 0 | 0 | 0 | 0 | 0 | 0 | 0 | 0 | 93% | 0 | 95% |
| 2205 | PMS1 protein homolog 1 OS=Homo sapiens GN=PMS1 PE=1 SV=1 | PMS1 | 106 | 0.44 | 95% | 0 | 0 | 0 | 0 | 0 | 0 | 0 | 0 | 0 | 0 | 0 |
| 2206 | 26S protease regulatory subunit 10B OS=Homo sapiens GN=PSMC6 PE=1 SV=1 | PRS10 | 44 | 0.44 | 0 | 0 | 0 | 0 | 0 | 0 | 95% | 57% | 0 | 0 | 0 | 0 |
| 2207 | Proteasome subunit beta type-5 OS=Homo sapiens GN=PSMB5 PE=1 SV=3 | PSB5 | 28 | 0.44 | 0 | 0 | 0 | 0 | 0 | 0 | 95% | 0 | 0 | 0 | 0 | 0 |
| 2208 | Ras-related protein Rab-30 OS=Homo sapiens GN=RAB30 PE=1 SV=2 | RAB30 | 23 | 0.052 | 0 | 0 | 0 | 0 | 0 | 0 | 0 | 0 | 0 | 95% | 95% | 0 |
| 2209 | Ras-related protein Rab-43 OS=Homo sapiens GN=RAB43 PE=1 SV=1 | RAB43 (+1) | 23 | 0.066 | 0 | 0 | 0 | 0 | 95% | 95% | 0 | 0 | 0 | 0 | 0 | 0 |
| 2210 | Double-strand-break repair protein rad21 homolog OS=Homo sapiens GN=RAD21 PE=1 SV=2 | RAD21 | 72 | 0.052 | 0 | 0 | 0 | 0 | 0 | 0 | 95% | 0 | 95% | 0 | 0 | 0 |
| 2211 | RNA-binding protein 12B OS=Homo sapiens GN=RBM12B PE=1 SV=2 | RB12B | 118 | 0.44 | 0 | 0 | 0 | 0 | 0 | 0 | 0 | 0 | 0 | 0 | 95% | 0 |
| 2212 | Rab3 GTPase-activating protein catalytic subunit OS=Homo sapiens GN=RAB3GAP1 PE=1 SV=3 | RB3GP | 111 | 0.052 | 0 | 0 | 0 | 0 | 0 | 0 | 0 | 0 | 0 | 95% | 0 | 95% |
| 2213 | Pre-mRNA-splicing factor RBM22 OS=Homo sapiens GN=RBM22 PE=1 SV=1 | RBM22 | 47 | 0.44 | 0 | 0 | 0 | 0 | 0 | 0 | 0 | 0 | 0 | 0 | 11% | 95% |
| 2214 | Rhomboid domain-containing protein 2 OS=Homo sapiens GN=RHBDD2 PE=2 SV=2 | RHBD2 | 39 | 0.44 | 0 | 0 | 0 | 0 | 0 | 0 | 95% | 0 | 0 | 0 | 0 | 0 |
| 2215 | sp|P50914|RL14_HUMAN-DECOY 60S ribosomal protein L14 OS=Homo sapiens GN=RPL14 PE=1... | RL14-DECOY | ? | 0.052 | 0 | 0 | 0 | 0 | 80% | 95% | 0 | 0 | 0 | 0 | 0 | 0 |
| 2216 | 39S ribosomal protein L12, mitochondrial OS=Homo sapiens GN=MRPL12 PE=1 SV=2 | RM12 | 21 | 0.6 | 0 | 0 | 0 | 0 | 95% | 0 | 0 | 0 | 0 | 0 | 0 | 95% |
| 2217 | 39S ribosomal protein L45, mitochondrial OS=Homo sapiens GN=MRPL45 PE=1 SV=2 | RM45 | 35 | 0.44 | 0 | 0 | 0 | 0 | 0 | 0 | 0 | 0 | 0 | 95% | 0 | 9% |
| 2218 | RING finger protein 11 OS=Homo sapiens GN=RNF11 PE=1 SV=1 | RNF11 | 17 | 0.052 | 0 | 0 | 0 | 0 | 95% | 95% | 0 | 0 | 0 | 0 | 0 | 0 |
| 2219 | RRP15-like protein OS=Homo sapiens GN=RRP15 PE=1 SV=2 | RRP15 | 31 | 0.44 | 0 | 0 | 0 | 0 | 0 | 0 | 0 | 0 | 0 | 0 | 0 | 95% |
| 2220 | Splicing factor 3B subunit 5 OS=Homo sapiens GN=SF3B5 PE=1 SV=1 | SF3B5 | 10 | 0.052 | 0 | 0 | 0 | 0 | 0 | 0 | 0 | 0 | 0 | 95% | 0 | 95% |
| 2221 | Slit homolog 3 protein OS=Homo sapiens GN=SLIT3 PE=2 SV=3 | SLIT3 | 168 | 0.44 | 0 | 0 | 95% | 0 | 0 | 0 | 0 | 0 | 0 | 0 | 0 | 0 |
| 2222 | Sperm protein associated with the nucleus on the X chromosome B/F OS=Homo sapiens GN=SPANXB1 PE=2 SV=1 | SPNXB (+2) | 12 | 0.056 | 95% | 0 | 95% | 0 | 0 | 0 | 0 | 0 | 0 | 0 | 0 | 0 |
| 2223 | General transcription factor 3C polypeptide 3 OS=Homo sapiens GN=GTF3C3 PE=1 SV=1 | TF3C3 | 101 | 0.44 | 0 | 0 | 0 | 0 | 0 | 0 | 0 | 0 | 0 | 95% | 8% | 0 |
| 2224 | Metalloproteinase inhibitor 3 OS=Homo sapiens GN=TIMP3 PE=1 SV=2 | TIMP3 | 24 | 0.44 | 0 | 0 | 0 | 0 | 0 | 0 | 95% | 0 | 0 | 0 | 0 | 0 |
| 2225 | Transmembrane protein 104 OS=Homo sapiens GN=TMEM104 PE=1 SV=2 | TM104 | 56 | 0.052 | 0 | 0 | 0 | 0 | 0 | 0 | 95% | 94% | 0 | 0 | 0 | 0 |
| 2226 | TOM1-like protein 2 OS=Homo sapiens GN=TOM1L2 PE=1 SV=1 | TM1L2 | 56 | 0.44 | 0 | 0 | 0 | 0 | 0 | 0 | 0 | 0 | 0 | 95% | 0 | 0 |
| 2227 | Transmembrane protein 258 OS=Homo sapiens GN=TMEM258 PE=1 SV=1 | TM258 | 9 | 0.44 | 0 | 27% | 95% | 0 | 0 | 0 | 0 | 0 | 0 | 0 | 0 | 0 |
| 2228 | Transmembrane protein 9 OS=Homo sapiens GN=TMEM9 PE=1 SV=1 | TMEM9 | 21 | 0.44 | 0 | 0 | 95% | 0 | 0 | 0 | 0 | 0 | 0 | 0 | 0 | 0 |
| 2229 | Transmembrane protein 68 OS=Homo sapiens GN=TMEM68 PE=2 SV=2 | TMM68 | 37 | 0.6 | 0 | 0 | 0 | 0 | 95% | 0 | 0 | 95% | 0 | 0 | 0 | 0 |
| 2230 | TRMT1-like protein OS=Homo sapiens GN=TRMT1L PE=1 SV=2 | TRM1L | 82 | 0.052 | 0 | 0 | 0 | 0 | 0 | 0 | 0 | 0 | 0 | 95% | 0 | 95% |
| 2231 | Vacuolar protein sorting-associated protein 26B OS=Homo sapiens GN=VPS26B PE=1 SV=2 | VP26B | 39 | 0.59 | 0 | 95% | 0 | 0 | 95% | 0 | 0 | 0 | 0 | 0 | 0 | 0 |
| 2232 | Zinc finger MYM-type protein 1 OS=Homo sapiens GN=ZMYM1 PE=2 SV=1 | ZMYM1 | 129 | 0.44 | 0 | 0 | 0 | 0 | 0 | 0 | 0 | 95% | 0 | 0 | 0 | 0 |
| 2233 | BUB3-interacting and GLEBS motif-containing protein ZNF207 OS=Homo sapiens GN=ZNF207 PE=1 SV=1 | ZN207 | 51 | 0.44 | 0 | 0 | 95% | 0 | 0 | 52% | 0 | 0 | 0 | 0 | 0 | 0 |
| 2234 | U6 snRNA-associated Sm-like protein LSm8 OS=Homo sapiens GN=LSM8 PE=1 SV=3 | LSM8 | 10 | 0.44 | 0 | 0 | 0 | 0 | 0 | 0 | 0 | 0 | 95% | 6% | 0 | 0 |
| 2235 | Peflin OS=Homo sapiens GN=PEF1 PE=1 SV=1 | PEF1 | 30 | 0.44 | 0 | 0 | 37% | 0 | 0 | 0 | 0 | 0 | 95% | 0 | 0 | 0 |
| 2236 | CTP synthase 1 OS=Homo sapiens GN=CTPS1 PE=1 SV=2 | PYRG1 | 67 | 0.44 | 0 | 0 | 0 | 0 | 0 | 0 | 0 | 0 | 95% | 0 | 0 | 0 |
| 2237 | Glutamine--tRNA ligase OS=Homo sapiens GN=QARS PE=1 SV=1 | SYQ | 88 | 0.44 | 0 | 0 | 0 | 0 | 0 | 0 | 0 | 0 | 95% | 0 | 0 | 0 |
| 2238 | Carbohydrate sulfotransferase 9 OS=Homo sapiens GN=CHST9 PE=2 SV=2 | CHST9 | 52 | 0.44 | 86% | 0 | 95% | 0 | 0 | 0 | 0 | 0 | 0 | 0 | 0 | 0 |
| 2239 | Heat shock 70 kDa protein 4 OS=Homo sapiens GN=HSPA4 PE=1 SV=4 | HSP74 | 94 | 0.44 | 0 | 91% | 0 | 0 | 0 | 0 | 0 | 0 | 0 | 0 | 0 | 0 |
| 2240 | Ral GTPase-activating protein subunit alpha-1 OS=Homo sapiens GN=RALGAPA1 PE=1 SV=1 | RGPA1 | 230 | 0.44 | 0 | 91% | 0 | 0 | 0 | 0 | 0 | 0 | 0 | 0 | 0 | 0 |
| 2241 | P50454-DECOY | P50454-DECOY | ? | 0.59 | 0 | 88% | 0 | 0 | 0 | 0 | 0 | 0 | 0 | 0 | 90% | 0 |
| 2242 | Q6ZNE9-DECOY | Q6ZNE9-DECOY | ? | 0.44 | 0 | 0 | 0 | 0 | 0 | 0 | 0 | 90% | 0 | 0 | 0 | 0 |
| 2243 | Serine-threonine kinase receptor-associated protein OS=Homo sapiens GN=STRAP PE=1 SV=1 | STRAP | 38 | 0.44 | 0 | 0 | 0 | 0 | 0 | 0 | 0 | 0 | 0 | 0 | 90% | 30% |
| 2244 | Q6ZW33-DECOY | Q6ZW33-DECOY | ? | 0.44 | 0 | 0 | 0 | 0 | 0 | 0 | 0 | 0 | 0 | 0 | 88% | 0 |
| 2245 | ER lumen protein retaining receptor 2 OS=Homo sapiens GN=KDELR2 PE=1 SV=1 | ERD22 | 24 | 0.29 | 0 | 44% | 0 | 0 | 0 | 0 | 100% | 34% | 0 | 0 | 0 | 0 |
| 2246 | Deoxyribonuclease-1-like 1 OS=Homo sapiens GN=DNASE1L1 PE=1 SV=1 | DNSL1 | 34 | 0.44 | 0 | 0 | 0 | 95% | 0 | 0 | 0 | 0 | 0 | 0 | 0 | 0 |
| 2247 | EF-hand calcium-binding domain-containing protein 14 OS=Homo sapiens GN=EFCAB14 PE=2 SV=1 | EFC14 | 55 | 0.44 | 0 | 0 | 0 | 95% | 0 | 0 | 0 | 0 | 0 | 0 | 0 | 0 |
| 2248 | Transmembrane protein 132E OS=Homo sapiens GN=TMEM132E PE=2 SV=1 | T132E | 107 | 0.44 | 0 | 0 | 0 | 95% | 0 | 0 | 0 | 0 | 0 | 0 | 0 | 0 |
| 2249 | sp|Q96SB8|SMC6_HUMAN-DECOY Structural maintenance of chromosomes protein 6 OS=Homo... | SMC6-DECOY | ? | 0.44 | 0 | 0 | 0 | 95% | 0 | 0 | 0 | 0 | 0 | 0 | 0 | 0 |
| 2250 | ATP-binding cassette sub-family D member 1 OS=Homo sapiens GN=ABCD1 PE=1 SV=2 | ABCD1 | 83 | 0.44 | 0 | 0 | 0 | 0 | 95% | 0 | 0 | 0 | 0 | 0 | 0 | 0 |
| 2251 | sp|Q16352|AINX_HUMAN-DECOY Alpha-internexin OS=Homo sapiens GN=INA PE=1 SV=2 | AINX-DECOY | ? | 0.44 | 0 | 0 | 0 | 0 | 0 | 0 | 0 | 95% | 0 | 0 | 0 | 0 |
| 2252 | sp|O75179|ANR17_HUMAN-DECOY Ankyrin repeat domain-containing protein 17 OS=Homo sapiens... | ANR17-DECOY | ? | 0.44 | 0 | 95% | 0 | 0 | 0 | 0 | 0 | 0 | 0 | 0 | 0 | 0 |
| 2253 | sp|Q9UIF8|BAZ2B_HUMAN-DECOY Bromodomain adjacent to zinc finger domain protein 2B... | BAZ2B-DECOY | ? | 0.44 | 0 | 0 | 0 | 0 | 0 | 0 | 0 | 95% | 0 | 0 | 0 | 0 |
| 2254 | sp|Q96G01|BICD1_HUMAN-DECOY Protein bicaudal D homolog 1 OS=Homo sapiens GN=BICD1... | BICD1-DECOY | ? | 0.44 | 0 | 95% | 0 | 0 | 0 | 0 | 0 | 0 | 0 | 0 | 0 | 0 |
| 2255 | Cytochrome b561 domain-containing protein 2 OS=Homo sapiens GN=CYB561D2 PE=1 SV=1 | C56D2 | 24 | 0.44 | 0 | 0 | 0 | 0 | 0 | 0 | 0 | 0 | 0 | 0 | 0 | 95% |
| 2256 | Carboxypeptidase A4 OS=Homo sapiens GN=CPA4 PE=1 SV=2 | CBPA4 | 47 | 0.44 | 0 | 0 | 0 | 0 | 0 | 0 | 0 | 0 | 0 | 0 | 95% | 0 |
| 2257 | Coiled-coil domain-containing protein 115 OS=Homo sapiens GN=CCDC115 PE=1 SV=1 | CC115 | 20 | 0.44 | 0 | 0 | 0 | 0 | 0 | 95% | 0 | 0 | 0 | 0 | 0 | 0 |
| 2258 | CCAAT/enhancer-binding protein zeta OS=Homo sapiens GN=CEBPZ PE=1 SV=3 | CEBPZ | 121 | 0.44 | 0 | 0 | 0 | 0 | 0 | 0 | 0 | 0 | 0 | 95% | 0 | 0 |
| 2259 | Uncharacterized protein C7orf50 OS=Homo sapiens GN=C7orf50 PE=1 SV=1 | CG050 | 22 | 0.44 | 0 | 95% | 0 | 0 | 0 | 0 | 0 | 0 | 0 | 0 | 0 | 0 |
| 2260 | Charged multivesicular body protein 1a OS=Homo sapiens GN=CHMP1A PE=1 SV=1 | CHM1A | 22 | 0.44 | 0 | 0 | 0 | 0 | 0 | 0 | 95% | 0 | 0 | 0 | 0 | 0 |
| 2261 | Cold-inducible RNA-binding protein OS=Homo sapiens GN=CIRBP PE=1 SV=1 | CIRBP | 19 | 0.44 | 0 | 0 | 0 | 0 | 0 | 0 | 0 | 0 | 0 | 0 | 95% | 0 |
| 2262 | Collagen alpha-1(VIII) chain OS=Homo sapiens GN=COL8A1 PE=1 SV=2 | CO8A1 | 73 | 0.44 | 0 | 0 | 0 | 0 | 0 | 0 | 0 | 0 | 0 | 0 | 95% | 0 |
| 2263 | sp|P13611|CSPG2_HUMAN-DECOY Versican core protein OS=Homo sapiens GN=VCAN PE=1 SV=3 | CSPG2-DECOY | ? | 0.44 | 0 | 0 | 0 | 0 | 0 | 95% | 0 | 0 | 0 | 0 | 0 | 0 |
| 2264 | sp|Q13616|CUL1_HUMAN-DECOY Cullin-1 OS=Homo sapiens GN=CUL1 PE=1 SV=2 | CUL1-DECOY | ? | 0.44 | 0 | 0 | 0 | 0 | 0 | 0 | 0 | 0 | 0 | 0 | 95% | 0 |
| 2265 | DAZ-associated protein 1 OS=Homo sapiens GN=DAZAP1 PE=1 SV=1 | DAZP1 | 43 | 0.44 | 0 | 0 | 0 | 0 | 0 | 0 | 0 | 0 | 0 | 95% | 0 | 0 |
| 2266 | Peroxisomal 2,4-dienoyl-CoA reductase OS=Homo sapiens GN=DECR2 PE=1 SV=1 | DECR2 | 31 | 0.44 | 0 | 95% | 0 | 0 | 0 | 0 | 0 | 0 | 0 | 0 | 0 | 0 |
| 2267 | Dehydrogenase/reductase SDR family member on chromosome X OS=Homo sapiens GN=DHRSX PE=2 SV=2 | DHRSX | 36 | 0.44 | 0 | 0 | 0 | 0 | 0 | 0 | 0 | 0 | 0 | 95% | 0 | 0 |
| 2268 | DnaJ homolog subfamily A member 2 OS=Homo sapiens GN=DNAJA2 PE=1 SV=1 | DNJA2 | 46 | 0.44 | 0 | 0 | 0 | 0 | 0 | 0 | 0 | 0 | 0 | 95% | 0 | 0 |
| 2269 | Eukaryotic peptide chain release factor subunit 1 OS=Homo sapiens GN=ETF1 PE=1 SV=3 | ERF1 | 49 | 0.44 | 0 | 0 | 0 | 0 | 0 | 0 | 0 | 0 | 0 | 95% | 0 | 0 |
| 2270 | Exosome complex component RRP41 OS=Homo sapiens GN=EXOSC4 PE=1 SV=3 | EXOS4 | 26 | 0.44 | 0 | 0 | 0 | 0 | 0 | 0 | 0 | 95% | 0 | 0 | 0 | 0 |
| 2271 | Exosome complex component RRP43 OS=Homo sapiens GN=EXOSC8 PE=1 SV=1 | EXOS8 | 30 | 0.44 | 0 | 0 | 0 | 0 | 0 | 0 | 0 | 95% | 0 | 0 | 0 | 0 |
| 2272 | Forkhead box protein N1 OS=Homo sapiens GN=FOXN1 PE=2 SV=1 | FOXN1 | 69 | 0.44 | 0 | 0 | 0 | 0 | 95% | 0 | 0 | 0 | 0 | 0 | 0 | 0 |
| 2273 | 4-aminobutyrate aminotransferase, mitochondrial OS=Homo sapiens GN=ABAT PE=1 SV=3 | GABT | 56 | 0.44 | 0 | 0 | 0 | 0 | 0 | 0 | 0 | 95% | 0 | 0 | 0 | 0 |
| 2274 | Protein GPR108 OS=Homo sapiens GN=GPR108 PE=2 SV=3 | GP108 | 61 | 0.44 | 0 | 0 | 0 | 0 | 0 | 0 | 95% | 0 | 0 | 0 | 0 | 0 |
| 2275 | Putative GTP-binding protein 6 OS=Homo sapiens GN=GTPBP6 PE=2 SV=3 | GTPB6 | 57 | 0.44 | 0 | 0 | 95% | 0 | 0 | 0 | 0 | 0 | 0 | 0 | 0 | 0 |
| 2276 | DNA-binding protein Ikaros OS=Homo sapiens GN=IKZF1 PE=1 SV=1 | IKZF1 | 58 | 0.44 | 0 | 0 | 0 | 0 | 0 | 0 | 0 | 0 | 0 | 95% | 0 | 0 |
| 2277 | Kelch repeat and BTB domain-containing protein 12 OS=Homo sapiens GN=KBTBD12 PE=2 SV=2 | KBTBC | 71 | 0.44 | 0 | 0 | 0 | 0 | 0 | 0 | 0 | 0 | 0 | 0 | 0 | 95% |
| 2278 | Kynurenine 3-monooxygenase OS=Homo sapiens GN=KMO PE=1 SV=2 | KMO | 56 | 0.44 | 95% | 0 | 0 | 0 | 0 | 0 | 0 | 0 | 0 | 0 | 0 | 0 |
| 2279 | Leucine-rich repeat and calponin homology domain-containing protein 4 OS=Homo sapiens GN=LRCH4 PE=1 SV=2 | LRCH4 | 73 | 0.44 | 0 | 0 | 0 | 0 | 0 | 95% | 0 | 0 | 0 | 0 | 0 | 0 |
| 2280 | Lumican OS=Homo sapiens GN=LUM PE=1 SV=2 | LUM | 38 | 0.44 | 0 | 0 | 0 | 0 | 0 | 0 | 0 | 0 | 0 | 95% | 0 | 0 |
| 2281 | Matrilin-2 OS=Homo sapiens GN=MATN2 PE=1 SV=4 | MATN2 | 107 | 0.44 | 0 | 0 | 0 | 0 | 95% | 0 | 0 | 0 | 0 | 0 | 0 | 0 |
| 2282 | Calcium uptake protein 1, mitochondrial OS=Homo sapiens GN=MICU1 PE=1 SV=1 | MICU1 | 54 | 0.44 | 0 | 0 | 0 | 0 | 0 | 0 | 0 | 0 | 0 | 0 | 95% | 0 |
| 2283 | Multiple myeloma tumor-associated protein 2 OS=Homo sapiens GN=MMTAG2 PE=1 SV=1 | MMTA2 | 29 | 0.44 | 0 | 0 | 0 | 0 | 0 | 0 | 0 | 95% | 0 | 0 | 0 | 0 |
| 2284 | sp|Q9Y2K3|MYH15_HUMAN-DECOY Myosin-15 OS=Homo sapiens GN=MYH15 PE=1 SV=5 | MYH15-DECOY | ? | 0.44 | 0 | 0 | 0 | 0 | 0 | 95% | 0 | 0 | 0 | 0 | 0 | 0 |
| 2285 | sp|P52179|MYOM1_HUMAN-DECOY Myomesin-1 OS=Homo sapiens GN=MYOM1 PE=1 SV=2 | MYOM1-DECOY | ? | 0.44 | 0 | 0 | 0 | 0 | 95% | 0 | 0 | 0 | 0 | 0 | 0 | 0 |
| 2286 | NADH dehydrogenase [ubiquinone] 1 alpha subcomplex subunit 7 OS=Homo sapiens GN=NDUFA7 PE=1 SV=3 | NDUA7 | 13 | 0.44 | 0 | 0 | 0 | 0 | 0 | 0 | 0 | 0 | 0 | 95% | 0 | 0 |
| 2287 | sp|Q96BD5|PF21A_HUMAN-DECOY PHD finger protein 21A OS=Homo sapiens GN=PHF21A PE=1... | PF21A-DECOY | ? | 0.44 | 0 | 0 | 0 | 0 | 95% | 0 | 0 | 0 | 0 | 0 | 0 | 0 |
| 2288 | sp|Q9Y2H5|PKHA6_HUMAN-DECOY Pleckstrin homology domain-containing family A member... | PKHA6-DECOY | ? | 0.44 | 95% | 0 | 0 | 0 | 0 | 0 | 0 | 0 | 0 | 0 | 0 | 0 |
| 2289 | Pleckstrin homology domain-containing family O member 2 OS=Homo sapiens GN=PLEKHO2 PE=2 SV=1 | PKHO2 | 53 | 0.44 | 0 | 0 | 95% | 0 | 0 | 0 | 0 | 0 | 0 | 0 | 0 | 0 |
| 2290 | sp|O15031|PLXB2_HUMAN-DECOY Plexin-B2 OS=Homo sapiens GN=PLXNB2 PE=1 SV=3 | PLXB2-DECOY | ? | 0.44 | 0 | 0 | 0 | 0 | 0 | 0 | 0 | 95% | 0 | 0 | 0 | 0 |
| 2291 | sp|Q9BZL4|PP12C_HUMAN-DECOY Protein phosphatase 1 regulatory subunit 12C OS=Homo... | PP12C-DECOY | ? | 0.44 | 0 | 0 | 0 | 0 | 0 | 0 | 0 | 0 | 0 | 95% | 0 | 0 |
| 2292 | sp|Q5JSZ5|PRC2B_HUMAN-DECOY Protein PRRC2B OS=Homo sapiens GN=PRRC2B PE=1 SV=2 | PRC2B-DECOY | ? | 0.44 | 0 | 0 | 0 | 0 | 95% | 0 | 0 | 0 | 0 | 0 | 0 | 0 |
| 2293 | Ras-related protein Rab-24 OS=Homo sapiens GN=RAB24 PE=1 SV=1 | RAB24 | 23 | 0.44 | 0 | 95% | 0 | 0 | 0 | 0 | 0 | 0 | 0 | 0 | 0 | 0 |
[truncated: 24,369 more chars]
